# Population modeling with machine learning can enhance measures of mental health

**DOI:** 10.1093/gigascience/giab071

**Published:** 2021-10-15

**Authors:** Kamalaker Dadi, Gaël Varoquaux, Josselin Houenou, Danilo Bzdok, Bertrand Thirion, Denis Engemann

**Affiliations:** Inria, CEA, Neurospin, Parietal team, Université Paris Saclay, 91120 Palaiseau, France; Inria, CEA, Neurospin, Parietal team, Université Paris Saclay, 91120 Palaiseau, France; Montréal Neurological Institute, McGill University, Montreal, QC, Canada; Mila - Quebec Artificial Intelligence Institute, Montreal, QC, Canada; CEA, NeuroSpin, Psychiatry Team, UNIACT Lab, Université Paris Saclay, France; APHP, Mondor University Hospitals, Psychiatry Department, INSERM U955 Team 15 “Translational Psychiatry,” Créteil, France; Inria, CEA, Neurospin, Parietal team, Université Paris Saclay, 91120 Palaiseau, France; Mila - Quebec Artificial Intelligence Institute, Montreal, QC, Canada; Department of Biomedical Engineering, Montreal Neurological Institute, Faculty of Medicine, McGill University, Montreal, QC, Canada; Inria, CEA, Neurospin, Parietal team, Université Paris Saclay, 91120 Palaiseau, France; Inria, CEA, Neurospin, Parietal team, Université Paris Saclay, 91120 Palaiseau, France; Department of Neurology, Max Planck Institute for Human Cognitive and Brain Sciences, Germany

**Keywords:** mental health, proxy measures, machine learning, sociodemographic factors, brain imaging

## Abstract

**Background:**

Biological aging is revealed by physical measures, e.g., DNA probes or brain scans. In contrast, individual differences in mental function are explained by psychological constructs, e.g., intelligence or neuroticism. These constructs are typically assessed by tailored neuropsychological tests that build on expert judgement and require careful interpretation. Could machine learning on large samples from the general population be used to build proxy measures of these constructs that do not require human intervention?

**Results:**

Here, we built proxy measures by applying machine learning on multimodal MR images and rich sociodemographic information from the largest biomedical cohort to date: the UK Biobank. Objective model comparisons revealed that all proxies captured the target constructs and were as useful, and sometimes more useful, than the original measures for characterizing real-world health behavior (sleep, exercise, tobacco, alcohol consumption). We observed this complementarity of proxy measures and original measures at capturing multiple health-related constructs when modeling from, both, brain signals and sociodemographic data.

**Conclusion:**

Population modeling with machine learning can derive measures of mental health from heterogeneous inputs including brain signals and questionnaire data. This may complement or even substitute for psychometric assessments in clinical populations.

## Background

Quantitative measures of mental health remain challenging despite substantial efforts [1]. The field has struggled with unstable diagnostic systems [2], small sample sizes [3], and reliance on case-control studies [4]. Perhaps most importantly, mental health cannot be measured the same way diabetes can be assessed through plasma levels of insulin or glucose. Psychological constructs, e.g., intelligence or anxiety, can only be probed indirectly through lengthy expert-built questionnaires or structured examinations by a specialist. Although questionnaires often remain the best accessible option, their capacity to measure a construct is limited [5]. In practice, a full neuropsychological evaluation is not an automated process but relies on expert judgement to confront multiple answers and interpret them in the context of the broader picture, such as the cultural background of the participant. While the field of psychometrics has thoroughly studied the validity of psychological constructs and their measurement [6–8], the advent of new biophysical measurements of the brain brings new promises [9–11]. The growth of biobanks and advances in machine learning open the door to large-scale validation of psychological measures for mental health research [12], and the hope to develop more generalizable models [13]. Yet, to be reliable, machine learning needs large labeled datasets [14]. Its application to learning imaging biomarkers of mental disorders is limited by the availability of large cohorts with high-quality neuropsychiatric diagnoses [15].

By comparison, it is easier to collect data on the general population without information on clinical conditions. For brain health, such data have led to the development of proxy measures that quantify biological aging [11, 16–22]. One counterintuitive aspect of the methodology is that measures of biological aging can be obtained by focusing on the age of a person, which is known in advance and is in itself not interesting. Yet, by (imperfectly) predicting the age from brain data, machine learning can capture the relevant signal. On the basis of a population of brain images, it extracts the best guess for the age of a person, indirectly positioning that person within the population. Individual-specific prediction errors therefore reflect deviations from what is statistically expected [23]. The brain of a person can look similar to the brains commonly seen in older (or younger) people. The resulting brain-predicted age reflects physical and cognitive impairment in adults [16, 17, 24] and reveals neurodegenerative processes [22, 25]. Can this strategy of biomarker-like proxy measures be extended to other targets beyond the construct of aging? Extrapolating from these successes, we propose to build upon large datasets to extend the collection of health-related proxy measures that probe mental traits. For this end, we focused on constructs fundamentally different in terms of content and methodology.

One high-stake target is intelligence, which is measured through socially administered tests and is one of the most extensively studied constructs in psychology. Fluid intelligence refers to the putatively culture-free, heritable, and physiological component of intelligence [26, 27] and is a latent construct designed to capture individual differences in cognitive capacity. It has been robustly associated with neuronal maturation and is typically reflected in cognitive-processing speed and working-memory capacity [28]. Applied to psychiatric disorders, it may help characterize psychosis, bipolar disorder, and substance abuse [29, 30].

Neuroticism is a second promising target. As a key representative of the extensively studied Big Five personality inventory, neuroticism has a long-standing tradition in the psychology of individual differences [31, 32]. Neuroticism is measured using self-assessment questionnaires and conceptualized as capturing dispositional negative emotionality including anxiety and depressiveness [33]. It has been interculturally validated [26, 34], and population genetics studies have repeatedly linked neuroticism to shared genes [35–37]. Neuroticism has been shown useful in psychometric screening and supports predicting real-world behavior [38, 39].

Despite strong population-level heritability [40, 41], the link between psychological constructs, brain function, and genetics is still being actively researched [33, 42, 43]. Empowered by emerging large-scale datasets, current attempts to predict fluid intelligence or neuroticism from thousands of magnetic resonance imaging (MRI) scans argue in favor of heterogeneity and weakly generalizing effects [44, 45]. This stands in contrast to the remarkable performance obtained when predicting psychometric data from language-based inputs captured by Twitter and Facebook user data [46, 47]. Because MRI acquisitions can be difficult to come by in certain populations, the promises of social media data are appealing. However, such data may lead to measurement and selection biases that are difficult to control. Instead, background sociodemographic data may provide an easily accessible alternative for contextualizing the heterogeneity of psychological traits [48].

Another challenge is that psychological traits are often measured using arbitrary non-physical units, e.g., education degree or monthly income. In fact, society treats individual differences as categorical or continuous, depending on the practical context. While personality has been proposed to span a continuum [49], psychiatrists treat certain people as patients and not others [50]. Therefore, a measure that performs globally poorly at a continuous scale can be sufficient to distinguish subgroups because it may be informative around the boundary region between certain classes, e.g., pilots who should fly and who should not. Choosing the granularity with which to gauge psychological constructs is difficult.

Confronting the promises of population phenotyping with the challenges of measuring psychological traits raises the following questions: (i) Can the success of brain age at characterizing health be extended to other proxy measures directly targeting mental constructs? (ii) How well can various constructs related to mental health be approximated from general-purpose inputs not designed to measure specific latent constructs? (iii) What is the relative merit of brain imaging and sociodemographic characteristics? We tackled these questions by using machine learning to craft proxy measures in order to approximate well-characterized target measures from brain-imaging and sociodemographic data. We studied age, fluid intelligence, and neuroticism. These targets have been, traditionally, considered as proxies for mental health and are fundamentally different in terms of scope and nature. Our results suggest that, the same way brain age can enrich age as a predictor of neurological complications, the additional proxy measures proposed in this work can bring value for the study of mental health by enriching the mental asessments they were constructed from.

**Figure 1:**
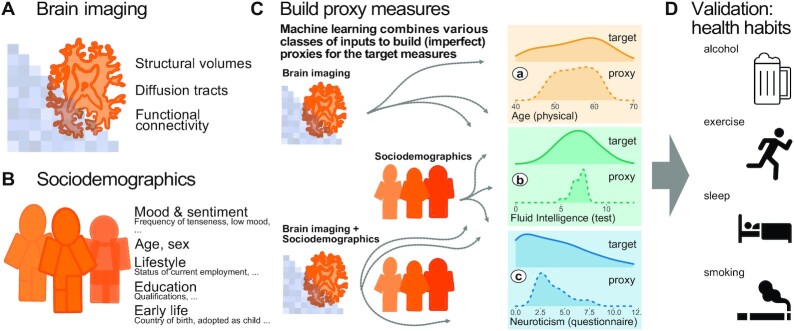
Methods workflow: building and evaluating proxy measures. We combined multiple brain-imaging modalities (**A**) with sociodemographic data (**B**) to approximate health-related biomedical and psychological constructs (**C**), i.e., brain age (assessed through prediction of chronological age), cognitive capacity (assessed through a fluid-intelligence test), and the tendency to report negative emotions (assessed through a neuroticism questionnaire). We included the imaging data from the 10,000-subjects release of the UK Biobank. Among imaging data (**A**) we considered features related to cortical and subcortical volumes, functional connectivity from rfMRI based on ICA networks, and white matter molecular tracts from diffusive directions (see Table 1 for an overview of the multiple brain-imaging modalities). We then grouped the sociodemographic data (**B**) into 5 different blocks of variables related to self-reported mood and sentiment, primary demographic characteristics, lifestyle, education, and early-life events (Table 2 lists the number of variables in each block). We systematically compared the approximations of all 3 targets based on either brain images or sociodemographic characteristics in isolation or combined (**C**) to evaluate the relative contribution of these distinct inputs. Note that proxy measures can only add to the target measures if they are not identical, i.e., if the approximation of the target from the given inputs is imperfect (guaranteed in our context because the exact data-generating mechanism is unknown and causally important variables remain unobserved). Using the full model (brain imaging + sociodemographic characteristics), we benchmarked complementarity of the proxy measures and the target measures with regard to real-world patterns of health behavior (**D**), i.e., the number of alcoholic beverages, exercise (metabolic equivalent task), sleep duration, and the number of cigarettes smoked. Potentially additive effects between proxies and targets were gauged using multiple linear regression. Models were developed on 50% of the data (randomly drawn) based on random forest regression guided by Monte Carlo cross-validation with 100 splits (see section “Model development and generalization testing"). We assessed generalization and health implications using the other 50% of the data as fully independent out-of-sample evaluations (see section “Statistical analysis"). Learning curves suggested that this split-half approach provided sufficient data for model construction (Fig. 1 – Fig. supplement 1).

The article is organized as follows: We first present a summary of the methodology and the workflow of building distinct proxy measures for age, fluid intelligence, and neuroticism using machine learning (Fig. 1). We then benchmark the proxy and the original target measures against real-world patterns of health-relevant behavior. Subsequently, through systematic model comparisons, we assess the relative contributions of brain imaging and sociodemographic data for prediction performance in the regression and classification settings. The complementarity between the proxy measures is, finally, discussed in the light of statistical considerations, potential data-generating mechanisms, and applications for public health and clinical research.

## Results: Validity of Proxy Measures

### Complementing the original measures at characterizing real-life health-related habits

To approximate age, fluid intelligence, and neuroticism, we applied random forest regression on sociodemographic data and brain images. The data were split into validation data for model construction (see section “Model development and generalization testing") and generalization data for statistical inference on out-of-sample predictions with independent data (see section “Statistical analysis"). Our findings suggested that some information on psychological constructs can be assembled from general inputs not specifically tailored to measure these constructs, such as brain images and sociodemographic variables. The resulting proxy measures can be regarded as crude approximations of the psychological measures, but they can nonetheless capture essential aspects of the target constructs. To probe the external validity of the proxy measures, we used left-out data to investigate their link with real-world behavior, e.g., sleep, physical exercise, and alcohol and tobacco consumption. To relate such health behaviors to our proxy measures, we modeled them separately as weighted sums of predicted brain age Δ, fluid intelligence, and neuroticism using multiple linear regression (section “Statistical analysis"). To avoid circularity, we used the out-of-sample predictions for all proxy measures (section “Model development and generalization testing").

**Figure 2:**
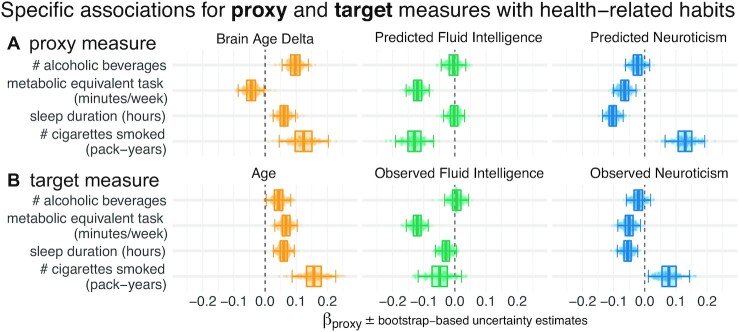
Proxy measures show systematic and complementary out-of-sample associations with health-related habits. We probed the external validity of all 3 proxy measures (brain age, fluid intelligence, neuroticism) based on a combination of brain images and all sociodemographic factors (see  Fig. 1 for details). We investigated their out-of-sample associations with ecological indicators of mental health (sleep duration, time spent with physical exercise, number of alcoholic beverages and cigarettes consumed). To tease apart complementary and redundant effects, we constructed multiple linear regression models on out-of-sample predictions combining all 3 proxy measures **(A)**. For comparison, we repeated the analysis using the actual target measures **(B)** observed on the held-out data. Regression models are depicted row-wise. Box plots summarize the uncertainty distribution of target-specific (color) regression coefficients, with whiskers indicating 2-sided 95% uncertainty intervals (parametric bootstrap). Dots illustrate a random subset of 200 out of 10,000 coefficient draws. At least 2 distinct patterns emerged: either the health outcome was specifically associated with 1 proxy measure (brain age Δ and number of alcoholic beverages) or multiple measures showed additive associations with the outcome (e.g., number of pack years smoked). For target measures **(B)**, associations with health habits were often noisier or less pronounced compared to the target measures **(A)** and even a change in direction was observed for brain age and metabolic activity. Figure 2 - Fig. supplement 1 shows highly similar trends with marginal associations between proxy measures and health-related habits. Our results suggest that the proxy measures capture health-related habits well, potentially better than the original target measures, and in a complementary way across the 3 measures. The same patterns emerged as brain-predicted age rather than the brain age Δ is used as a proxy measure (Fig. 2 - Fig. supplement 2). As proxy-specific deconfounding is applied, this pattern is preserved (Fig. 2 - Fig. supplement 3). Modeling of health-related habits jointly from proxy and target measures simultaneously revealed specific complementarity between proxy and target measures across multiple domains, i.e., age, fluid intelligence, and neuroticism (Fig. 2 - Fig. supplement 4).

The estimated regression coefficients (partial correlations) revealed complementary associations between the proxy measures and health-related behavior (Fig. 2). Similar patterns arise when proxy measures are considered in isolation  (Fig. 2 - Fig. supplement 1). Compared with other proxy measures, elevated brain age Δ was associated with increased alcohol consumption (Fig. 2, first row). Levels of physical exercise were consistently associated with all 3 predicted targets, suggesting additive effects (Fig. 2, second row). For fluid intelligence, this result, counterintuitive from the health standpoint, could imply that higher test scores reveal a more sedentary lifestyle. Increased sleep duration consistently went along with elevated brain age Δ but lower levels of predicted neuroticism (Fig. 2, third row). This may seem counterintuitive but is conditional on neuroticism showing a negative link with sleep duration. No consistent effect emerged for fluid intelligence. Numbers of cigarettes smoked was independently associated with all predicted targets (Fig. 2, last row): Intensified smoking went along with elevated brain age Δ and neuroticism but lower fluid intelligence.

The 3 proxy measures are difficult to compare on an equal footing because a Δ was considered for brain age only (the difference between predicted and actual age) and aging-specific deconfounding was applied. The brain age Δ is indeed the standard practice, theoretically justified because age is on a metric scale [51] for which the difference between the predicted and the measured value has a clear meaning. Such a difference is less obvious for variables with ordinal scales as implied by psychometric measures. Second, age has a pervasive influence on virtually any biomedical entity, which motivates controlling for its effect on proxy measures. To rule out the possibility that differences in proxy measures’ associations with health-related behavior are driven by this methodological asymmetry, we repeated the main analysis from Fig. 2, first, using the predicted age without computing the Δ (Fig. 2 - Fig. supplement 2) and, second, introducing additional deconfounders for fluid intelligence and neuroticism (Fig. 2 - Fig. supplement 3). The resulting patterns were virtually unchanged, confirming that interpretations are robust.

A question that remains is whether the proxy measures bring additional value compared to the original target measures from which they were derived. These original target measures showed similar associations with health behavior, with the same signs in most cases (Fig. 2B). At the same time, the ensuing patterns were noisier, suggesting that empirically derived proxy measures yielded enhanced associations with health behavior. This inference may be difficult to make because differences between targets and proxies were not always easy to pinpoint visually. To implement a more rigorous statistical approach, we built comprehensive models of each respective health-related habit in which we used all proxies (predicted age, predicted fluid intelligence, predicted neuroticism) and all targets (age, fluid intelligence, neuroticism) simultaneously as predictors (Fig. 2 - Fig. supplement 4). The results show systematic additive effects of proxies and targets across the 3 target domains and the 4 health habits. These trends are well captured by the hypothesis tests of the respective linear models (Supplementary Table S3). Because targets and proxies may be systematically intercorrelated, multicollinearity may corrupt these inferences. Inspection of variance inflation factors (VIF)—a measure that reveals how well a given predictor can be approximated by a linear combination of the other predictors—argued in favor of low to moderate levels of multicollinearity (Supplementary Table S4). Indeed, all VIF values fell between 3 and 1, whereas, classically, values >5 or 10 are considered as thresholds [52] for pathological collinearity. This suggests that the model inferences are statistically sound.

### The relative importance of brain and sociodemographic data depends on the target

**Figure 3:**
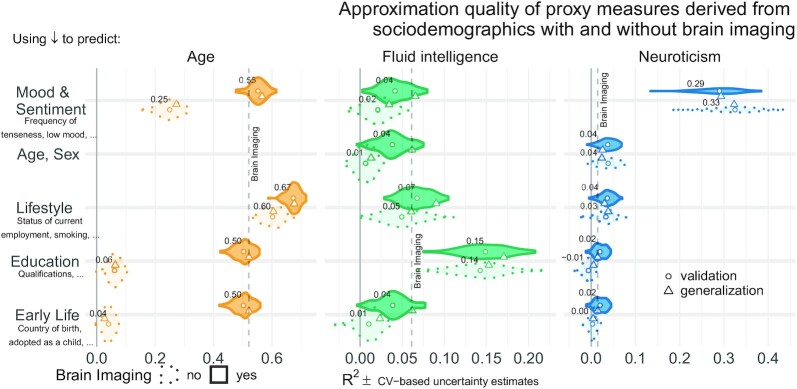
Approximation performance of proxy measures derived from sociodemographic data and MRI. We report the *R*^2^ metric to facilitate comparisons across prediction targets. The cross-validation (CV) distribution (100 Monte Carlo splits) on the validation dataset is depicted by violins. Drawing style indicates whether brain imaging (solid outlines of violins) was included or excluded (dotted outlines of violins). Dots depict the average performance on the validation data across CV-splits. Triangles depict the performance of the average prediction (CV-bagging) on held-out generalization datasets. For convenience, the mean performance on the validation set is annotated for each plot. Vertical dotted lines indicate the average performance of the full MRI model. The validation and held-out datasets gave a similar picture of approximation performance with no evidence for cross-validation bias [53]. For the averaged out-of-sample predictions, the probability of the observed performance under the null distribution and the uncertainty of effect sizes was formally probed using permutation tests and bootstrap-based confidence intervals (Supplementary Table S1). Corresponding statistics for the baseline performance of models solely based on brain imaging (vertical dotted lines) are presented in Supplementary Table S5. Figure 3 - Fig. supplement 1 shows approximation results based on MRI. Figure 3 - Fig. supplement 2 presents results based on all sociodemographic factors.

In a second step, we investigated the relative performance of proxy measures built from brain signals and distinct sociodemographic factors for the 3 targets: age, fluid intelligence, and neuroticism. Among the sociodemographic variables there was 1 block for each target explaining most of the prediction performance (Fig. 3, dotted outlines). Combining all sociodemographic variables did not lead to obvious enhancements (Fig. 3 - Fig. supplement 2). For age prediction, variables related to current lifestyle showed by far the highest performance. For fluid intelligence, education performed by far best. For neuroticism, mood and sentiment clearly showed the strongest performance.

Combining MRI and sociodemographic characteristics enhanced age prediction systematically across all 4 blocks of variables  (Fig. 3 solid outlines and Supplementary Table S1). The benefit of brain-imaging features was less marked for prediction of fluid intelligence or neuroticism. For fluid intelligence, brain-imaging data led to statistically significant improvements of performance, however, with small effect sizes (Supplementary Table S1). For neuroticism, no systematic benefit of including brain images alongside sociodemographic characteristics emerged (Supplementary Table S1, bottom row). Nevertheless, brain data were sufficient for statistically significant approximation of the target measures in all 3 targets (Supplementary Table S5).

**Figure 4:**
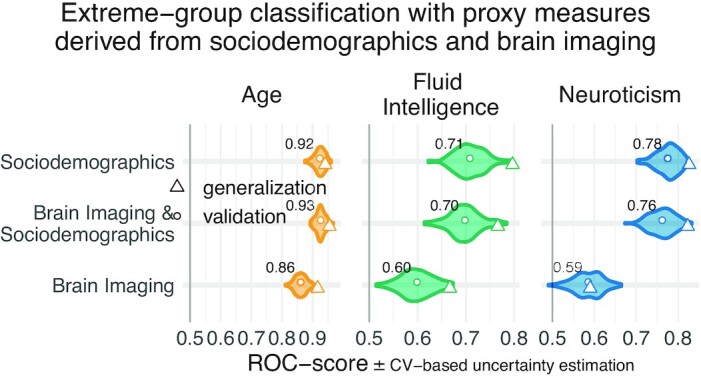
Classification analysis from imaging, sociodemographic characteristics, and combination of both data types. For classification of extreme groups instead of continuous regression, we split the data into low vs high groups based on 33rd and 66th percentiles. Visual conventions follow Fig. 3. We report the accuracy in AUC. Models including sociodemographic characteristics performed visibly better than models purely based on brain imaging. Differences between brain-imaging and sociodemographic characteristics appeared less pronounced as compared to the fully fledged regression analysis. For the average out-of-sample predictions, the probability of the observed performance under the null distribution and the uncertainty of effect sizes were formally probed using permutation tests and bootstrap-based confidence intervals (Supplementary Table S2). Corresponding statistics for the baseline performance of models solely based on brain imaging (vertical dotted lines) are presented in Supplementary Table S6. Overall, when moving from the more difficult full-scale regression problem to the extreme-group classification problem with purely ranking-based scores, the relative differences between brain-based and sociodemographic characteristics-based prediction gradually faded away.

Psychological measures often come without physical scales and units [51]. In practice, clinicians and educators use them with specific thresholds for decision making. To investigate empirically defined proxy measures beyond continuous regression, we performed binary classification of extreme groups obtained from discretizing the targets using the 33rd and 66th percentiles, following the recommendations by Gelman and Hill [54] regarding discrete variable encoding strategies. Furthermore, we measured accuracy with the area under the classification accuracy curve (AUC), which is only sensitive to ranking, ignoring the scale of the error. Classification performance visibly exceeded the chance level (AUC >0.5) for all models (Fig. 4) and approached or exceeded levels considered practically useful (AUC >0.8) [50]. Across proxy measures, models including sociodemographic characteristics performed best but the difference between purely sociodemographic and brain-based models was comparably weak, at the order of 0.01–0.02 AUC points (Supplementary Table S2). Using brain-imaging data alone led to degraded performance that was, nevertheless, better than chance, as revealed by permutation testing (Supplementary Table S6).

## Discussion

Guided by machine learning, we empirically derived proxy measures that combine multiple sources of information to capture extensively validated target measures from psychology. These proxy measures all showed complementary associations with real-world health indicators beyond the original targets. The combination of brain imaging and target-specific sociodemographic inputs often improved approximation performance.

### Empirically derived proxy measures: validity and practical utility

In our study, construct validity [6, 7, 55] of the corresponding proxy measures was supported by the gain in prediction performance brought by specific sociodemographic factors (Fig. 3). Association with health-relevant habits added external validity to the proxy measures (Fig. 2). The complementary patterns related to traditional construct semantics: High consumption of cigarettes is associated with neuroticism [56]; excessive drinking may lead to brain atrophy and cognitive decline [57]—both common correlates of increased brain age [22, 58].

Can our empirically derived proxy measures thus substitute for specific psychometric instruments? A mental health professional may still prefer an established routine for clinical assessment, relying on interviews and personality questionnaires with implicit experience-based thresholds. Inclusion of brain imaging may even seem to yield diminishing returns when approximating high-level psychological traits. Yet, it could simply be a matter of time until more effective acquisition techniques will be discovered alongside more powerful signal representations. Including brain imaging rather seems a “safe bet” because machine learning is often capable of selecting relevant inputs [11, 59] and costs of MRI acquisition can be amortized by clinical usage. Empirically derived proxy measures may open new doors where tailored assessment of latent constructs is not applicable due to lack of specialized mental health workforce or sheer cost.

### Constructs of mental health can be accessed from general-purpose data

Brain age has served as landmark in this study. It has been arguably the most discussed candidate for a surrogate biomarker in the brain-imaging literature [16, 17, 24]. With mean absolute errors ∼4 years, ≤67% variance explained, and AUC scores ≤0.93 in the classification setting, our results compare favorably to the recent brain age literature within the UK Biobank (UKBB) [19, 60] and in other datasets [11, 22], although we relied on off-the-shelf methods and not custom deep learning methods [61]. Applying the same approach to psychological constructs (fluid intelligence, neuroticism), we found that approximation from brain-imaging data or sociodemographic descriptors was generally harder.

It is important to recapitulate that approximation quality on these differently measured targets has a different meaning. Age is measured with meaningful physical units (years) on a ratio scale [51] (Selma is twice as old as Bob). Psychometric scores are unit-free, which may provoke ambiguity regarding the level of measurement [55]. Their implied scales may be considered as interval (the difference between Bob’s and Selma’s intelligence is −0.1 standard deviations) if not ordinal (Bob’s intelligence was ranked below Selma’s)  [51]. In day-to-day psychological practice, these scores are often used via practically defined thresholds, e.g., school admission or pilot candidate selection [62, 63]. In the classification setting, all proxy measures approached or exceeded a performance of 0.80 deemed relevant in biomarker development [50], although to be fair, they approximated established psychometric targets (proxy measures themselves) and not a medical condition. Different proxy measures should, thus, be subjected to different standards, depending on the granularity of the implied measurement scale.

A more complete view on how the proxy measures capture mental health constructs emerges from their associations with real-world behavior (Fig. 2). Indeed, the associations with proxy measures (Fig. 2B) were less noisy and more consistent than with the target measures (Fig. 2A), regardless of their approximation quality. This may seem surprising at first, but the target measures are themselves noisy and of imperfect validity. These measures correspond to traditional tests, which, in practice, must be interpreted by an expert, actively confronting their output with broader information on the individual. For instance, IQ scores are typically normalized across age groups. However, extending such a normalization approach to many factors (socio-economic status, culture, sex) poses fundamental high-dimensional statistics challenges. Conversely, using machine learning to assemble proxy measures by mapping the targets to rich sociodemographic and brain data implicitly contextualizes them. In this respect, the resulting measures capture more general signal than the original tests. Here, machine learning could be seen as mimicking the work of a mental health expert who carefully compares psychometric results with other facts known about an individual and its reference population.

### The benefits offered by brain data depend on the target construct

All brain-derived approximations were statistically meaningful. Yet, only for age prediction, imaging data by itself led to convincing performance. For fluid intelligence and neuroticism, sociodemographic factors were the most important determinants of prediction success. The best-performing sociodemographic models were based on inputs semantically close to these targets, i.e., education details or mood and sentiment. While those results support construct validity, they may come with a certain risk of circularity. The causal role of those predictors is not necessarily clear because better educational attainment is heritable itself [64] and may reinforce existing cognitive abilities. Similarly, stressful life events may exacerbate existing dispositions to experience negative emotions. Such dispositions can develop into traits captured by neuroticism [65] and can, in turn, lead to accumulating further stressful life events [38]. Nevertheless, for fluid intelligence but not neuroticism, brain imaging added incremental value when combined with various sociodemographic predictors. This may suggest that the cues for neuroticism conveyed by brain imaging were already present in sociodemographic predictors, hinting at common causes. Of note, in the specific context of aging, the empirical distinction between brain age and cognitive age (age predicted from cognitive and behavioral data) is reflecting a similar intuition that different inputs can yield complementary proxies of the same target [66].

### Limitations

Additional constructs and psychometric tools could have been evaluated. The broader construct of intelligence is often estimated using a general factor model with multiple correlated tests. While this is obviously useful for normative assessments, measures of fluid intelligence can also serve as a situational fitness signal [30]. There is a wealth of questionnaires for measuring negative emotionality and neuroticism, specifically. Yet, we could only study the EPQ scale provided by the UKBB. A complementary approach would be to estimate latent factors by pooling all non-imaging data semantically related to neuroticism [67]. Here, we considered established target measures “as is,” instead of derivatives.

It terms of mental health research, this study falls short of directly testing the clinical relevance of estimated proxy measures. Even in a very large general-population cohort such as the UKBB, there are only a few hundred diagnosed cases of mental disorders (ICD-10 mental health diagnoses from the F chapter) with brain-imaging data available. As a result, we could not directly assess the performance of proxy measures in clinical populations. The low number of diagnosed mental disorders in the UKBB highlights the practical importance of studying mental health as a continuous variable, in addition to diagnosed conditions. Indeed, a public health perspective calls for targeting individual differences in health, not only pathology. Psychological constructs such as IQ and neuroticism are important factors of the epidemiology of psychiatric disorders [29, 30, 38, 68], and accelerated brain aging is associated with various neurological conditions [17, 18, 25]. Yet, few cohorts come with extensive neuropsychological testing. Validated proxies of these constructs open the door to including them in epidemiological studies as secondary outcomes or additional explanatory variables.

## Conclusion

In population studies of mental health, individual traits are captured via lengthy assessments, tailored to specific brain and psychological constructs. We have shown that proxy measures built empirically from general-purpose data can capture these constructs and can improve upon traditional measures when studying real-world health patterns. Proxy measures can make psychological constructs available to broader, more ecological studies building on large epidemiological cohorts or real-world evidence. This can make the difference where psychological constructs are central to developing treatment and prevention strategies but direct measures have not been collected.

## Methods

To facilitate reproduction, understanding, and reuse, we have made all data analysis and visualization source code available on GitHub [69].

### Dataset

The UKBB database is to date the most extensive large-scale cohort aimed at studying the determinants of the health outcomes in the general adult population. The UKBB is openly accessible and has extensive data acquired on 500,000 individuals aged 40–70 years covering rich phenotypes, health-related information, brain-imaging, and genetic data [12]. Participants were invited for repeated assessments, some of which included MRI. For instance, cognitive tests that were administered during an initial assessment were also assessed during the follow-up visits. This has enabled finding for many participants ≥1 visit containing all heterogeneous input data needed to develop the proposed proxy measures. The study was conducted using the UKBB Resource Application 23827.

### Participants

All participants gave informed consent. The UKBB study was examined and approved by the North West Multi-centre Research Ethics Committee. We considered participants who have responded to cognitive tests and questionnaires and provide access to their primary demographic characteristics and brain images [70]. Out of the total size of UKBB populations, we found 11,175 participants who had repeated assessments overlapping with the first brain-imaging release [71]. Note that the features (sociodemographic variables) that we included in the analysis are measures that are self-reported during a follow-up imaging visit. The demographic characteristics are $51.6\%$ female (5,572) and $48.3\%$ male (5,403), and an age range of 40–70 years (mean [SD], 55 [7.5] years). The data for model training were selected using a randomized split-half procedure yielding 5,587 individuals. The remaining participants were set aside as a held-out set for generalization testing (see section “Model development and generalization testing"). We made sure that the participants used for model training and generalization testing were strictly non-overlapping.

Learning curves documented that the training split was sufficiently large for constructing stable prediction models (Fig. 1 - Fig. supplement 1) with profiles of performance similar to the latest benchmarks on model complexity in the UKBB [72]. Moreover, simulations and empirical findings suggest that larger testing sets are more effective at mitigating optimistic performance estimates [53, 73]. Together, this provided a pragmatic solution to the inference-prediction dilemma [59, 74] given the 2 objectives of the present investigation to obtain reasonably good predictive models while at the same time performing parameter inference of statistical models developed on the left-out data.

To establish specific comparisons between models based on sociodemographic characteristics, brain data, or their combinations, we exclusively considered the cases for which MRI scans were available. The final sample sizes used for model construction and generalization testing then depended on the availability of MRI: For age and fluid intelligence, our randomized split-half procedure (see section “Model development and generalization testing") yielded 4,203 cases for model building and 4,157 for generalization. For cases with valid neuroticism assessment, fewer brain images were available, which yielded 3,550 cases for model building and 3,509 for generalization.

### Data acquisition

Sociodemographic data (non-imaging) were collected with self-report measures administered through touchscreen questionnaires, complemented by verbal interviews, physical measures, biological sampling, and imaging data. MRI data were acquired with the Siemens Skyra 3T using a standard Siemens 32-channel RF receiver head coil [75]. We considered 3 MRI modalities because each of them potentially captures unique neurobiological details: structural MRI (sMRI/T1), resting-state functional MRI (rs-fMRI), and diffusion MRI (dMRI). For technical details about the MR acquisition parameters see [71]. We used image-derived phenotypes of those distinct brain-imaging modalities because they provide actionable summaries of the brain measurements and encourage comparability across studies.

#### Target measures

As our target measures for brain age modeling, we use an individual’s age at baseline recruitment (UKBB code “21022-0.0”). Fluid intelligence was assessed using a cognitive battery designed to measure an individual’s capacity to solve novel problems that require logic and abstract reasoning. In the UKBB, the fluid intelligence test (UKBB code “20016-2.0”) comprises 13 logic and reasoning questions that were administered via the touchscreen to record a response within 2 minutes for each question. Therefore, each correct answer is scored as 1 point, with 13 points in total (see the user manual [76] for an overview of the 13 items). Neuroticism (UKBB code “20127-0.0”) was measured using a shorter version of the revised Eysenck Personality Questionnaire (EPQ-N) comprising 12 items [32]. Neuroticism was assessed during the UKBB baseline visit. A score in the range of 0–12 summarizes dispositional tendency to experience negative emotions (a complete list of neuroticism questionnaires is provided by the dedicated field descriptions and derivation for variables related to bipolar disorder, major depression status, and neuroticism score  [77]).

In the course of this work, a question that emerged concerned the size of the gap between age at baseline recruitment and MRI scan time and its potential effect on the analysis. Supplementary checks indicated that the age gap was ≥5 years for most participants. Yet, from a statistical perspective, the 2 age measures turned out to be interchangeable (Supplementary Fig. S2) and global conclusions remained unchanged (Supplementary Fig. S3).

### Sociodemographic data

In this work, we refer to non-imaging variables broadly as sociodemographic characteristics excluding the candidate targets fluid intelligence and neuroticism. To approximate latent constructs from sociodemographic characteristics, we included 86 non-imaging inputs (Supplementary Table S7), which are the collection of variables reflecting each participant’s demographic and social factors i.e., sex, age, date and month of birth, body mass index, ethnicity, exposures at early life (e.g., breast feeding, maternal smoking around birth, adopted as a child), education, lifestyle-related variables (e.g., occupation, household family income, number of people in household, smoking habits), and mental health variables. All these data were self-reported. We then assigned these 86 variables to 5 groups based on their relationships. On the basis of our conceptual understanding of the variables, we assigned them to 1 of 5 groups: **(1)** mood and sentiment, (**2)** primary demographic characteristics such as age and sex, **(3)** lifestyle, **(4)** education, **and (5)** early life. We then investigated the intercorrelation between all 86 variables to ensure that the proposed grouping is compatible with their empirical correlation structure (Supplementary Fig. S1).

The sociodemographic groups had varying amounts of missing data, with a portion of the missingness related to the participants' lifestyle habits such as smoking and mental health issues [78]. To deal with this missingness in the data using imputation [79], we used column-wise replacement of missing information with the median value calculated from the known part of the variable. We subsequently included an indicator for the presence of imputed values for downstream analysis. Such imputation is well suited to predictive models [80].

### Image processing to derive phenotypes for machine learning

MRI data preprocessing was carried out by the UKBB imaging team. The full technical details are described elsewhere [71, 75]. Below, we describe briefly the custom processing steps that we used on top of the already preprocessed inputs.

#### Structural MRI

This type of data analysis on T1-weighted brain images is concerned with morphometry of the gray matter areas, i.e., the quantification of size, volume of brain structures and tissue types, and their variations under brain disease conditions or behavior [81]. For example, volume changes in gray matter areas over lifetime are associated with brain aging [82], general intelligence [83], and brain disease [84]. Such volumes are calculated within pre-defined regions of interest composed of cortical and sub-cortical structures [85] and cerebellar regions [86]. We included 157 sMRI features consisting of volume of total brain and gray matter along with brain subcortical structures [87, 88]. All these features are pre-extracted by the UKBB brain imaging team [71] and are part of the data download. We concatenated all inputs alongside custom-built fMRI features for predictive analysis (feature union).

#### Diffusion-weighted MRI

dMRI enables the identification of white matter tracts along the principal diffusive direction of water molecules, as well as the connections between different gray matter areas [89, 90]. The study of these local anatomical connections through white matter is relevant to the understanding of brain diseases and functional organization [91]. We included 432 dMRI skeleton features of FA (fractional anisotropy), MO (tensor mode), MD (mean diffusivity), ICVF (intra-cellular volume fraction), ISOVF (isotropic volume fraction), and OD (orientation dispersion index) modeled on many brain white matter structures extracted from neuroanatomy (dMRI skeleton measurements [92]; for technical details see [93]). The skeleton features we included were from Category 134 shipped by the UKBB brain-imaging team, and we used them without modification.

#### Functional MRI

Resting-state functional MRI captures low-frequency fluctuations in blood oxygenation that can reveal ongoing neuronal interactions in time forming distinct brain networks [94]. Functional connectivity within these brain networks can be linked to clinical status [95], to behavior [71], or to psychological traits [45]. We also included resting-state connectivity features based on the time series extracted from independent component analysis (ICA), with 55 components representing various brain networks extracted on UKBB rfMRI data [71]. These included the default mode network, extended default mode network, and cingulo-opercular network, executive control and attention network, visual network, and sensorimotor network. We measured functional connectivity in terms of the between-network covariance. We estimated the covariance matrices using Ledoit-Wolf shrinkage [96]. To account for the fact that covariance matrices live on a particular manifold, i.e., a curved non-Euclidean space, we used tangent-space embedding to transform the matrices into a Euclidean space [97, 98] following recent recommendations [99, 100]. For predictive modeling, we then vectorized the covariance matrices to 1, 485 features by taking the lower triangular part. These steps were performed with NiLearn [101].

### Comparing predictive models to approximate target measures

#### Imaging-based models

First, we focused on purely imaging-based models based on exhaustive combinations of the 3 types of MRI modalities (see Table 1 for an overview). This allowed us to study potential overlap and complementarity between the MRI modalities. Preliminary analyses revealed that combining all MRI data gave reasonable results with no evident disadvantage for particular combinations of MRI modalities (Fig. 3 - Fig. supplement 1); hence, for simplicity, we only focused on the full MRI model in subsequent analyses.

**Table 1.** Imaging-based models

Index	Name	No. variables	No. groups
1	Brain volumes (sMRI)	157	1
2	White matter (dMRI)	432	1
3	Functional connectivity (fMRI)	1,485	1
4	sMRI, dMRI	589	2
5	sMRI, fMRI	1,642	2
6	dMRI, fMRI	1,917	2
7	sMRI, dMRI, fMRI (full MRI)	2,074	3

#### Sociodemographic models

We composed predictive models based on non-exhaustive combinations of different types of sociodemographic variables. To investigate the relative importance of each class of sociodemographic inputs, we performed systematic model comparisons. We were particularly interested in studying the relative contributions of early-life factors as compared to factors related to more recent life events such as education as well as factors related to current circumstances such as mood and sentiment and lifestyle. The resulting models based on distinct groups of predictors are listed in Table 2 (for additional details see Supplementary Table S7 and  Supplementary Fig. S1).

**Table 2.** Non-imaging baseline models or sociodemographic models based on a single group

Index	Name	No. variables
1	Mood and Sentiment (MS)	25
2	Age, Sex (AS)	5
3	Lifestyle (LS)	45
4	Education (EDU)	2
5	Early Life (EL)	9

Variables in each group are described in section “Sociodemographic data.”

#### Combined imaging and sociodemographic models

In the next step, we were interested in how brain-related information would interact within each of these sociodemographic models. For example, information such as the age of an individual or the level of education may add important contextual information to brain images. We therefore considered an alternative variant for each of the models in Table 2 that included all MRI-related features (2,074 additional features) as described in section “Image processing to derive phenotypes for machine learning.”

#### Predictive model

Linear models are recommended as the default choice in neuroimaging research [99, 102] especially when datasets include <1,000 data points. This study approximated targets generated by distinct underlying mechanisms based on multiple classes of heterogenous input data with several thousands of data points. We hence chose the non-parametric random forest algorithm, which can be readily applied on data of different units for non-linear regression and classification [103] with mean squared error as impurity criterion. To improve computation time we fixed tree depth to 250 trees, a hyperparameter that is not usually tuned but set to a generous number because performance plateaus beyond a certain number of trees ([104], ch. 15). Preliminary analyses suggested that additional trees would not have led to substantial improvements in performance. We used nested cross-validation (5-fold grid search) to tune the depth of the trees as well as the number of variables considered for splitting (see Table 3 for a full list of hyperparameters considered).

**Table 3.** Random forest hyperparameters and tuning with grid search (5-fold cross-validation)

Hyperparameter	Values
Impurity criterion	Mean squared error
Maximum tree depth	5, 10, 20, 40, full depth
Fraction of features for split	1, 5, “log2,” “sqrt,” “complete”
No. of trees	250

##### Classification analysis

We also performed classification analysis on the continuous targets. Adapting recommendations from Gelman and Hill [54], we performed discrete variable encoding of the targets leading to extreme groups based on the 33rd and 66th percentiles (see Table 4 for the number of classification samples per group). This choice avoids including samples near the average outcome, for which the input data may be indistinct. We were particularly interested in understanding whether model performance would increase when moving toward classifying extreme groups. For this analysis, we considered all 3 types of models (full MRI 2,074 features from imaging-based models; all sociodemographic characteristics variables, total 86 variables see section, combination of full MRI and all sociodemographic characteristics, a total of 2,160 variables; see section “Comparing predictive models to approximate target measures”). When predicting age, we excluded the age and sex sociodemographic block from all sociodemographic variables, which then yielded a total of 81 variables. To assess the performance for classification analysis, we used the area under the curve (AUC) of the receiver operating characteristic (ROC) curve as an evaluation metric [102].

**Table 4.** Number of samples for classification analysis (N)

No. groups	Age	Fluid intelligence	Neuroticism
1	1,335	1,108	1,054
2	1,200	898	1,020

### Model development and generalization testing

Before any empirical work, we generated 2 random partitions of the data, 1 validation dataset for model construction and 1 held-out generalization dataset for studying out-of-sample associations using classical statistical analyses.

For cross-validation, we then subdivided the validation set into 100 training and testing splits following the Monte Carlo resampling scheme (also referred to as shuffle-split) with 10% of the data used for testing. To compare model performance based on paired tests, we used the same splits across all models. Split-wise testing performance was summarized for informal inference using violin plots (Figs 3 and 4). For generalization testing, predictions on the held-out data were generated from all 100 models from each cross-validation split.

On the held-out set, unique subject-wise predictions were obtained by averaging across folds and occasional duplicate predictions due to Monte Carlo sampling, which could produce multiple predictions per participant (we ensured prior to computation that with 100 CV-splits, predictions were available for all participants). Such a strategy is known as CV-bagging [105, 106] and can improve both performance and stability of results (the use of CV-bagging can explain why in Figs 3 and 4 and Fig. 3 - Figure supplement 1 the performance was sometimes slightly better on the held-out set compared to the cross-validation on the validation test). The resulting average predictions yielded the final proxy measures for the analysis of health-related behaviors in Fig. 2 and were reported in Fig. 3 and Fig. 4.

### Statistical analysis

#### Resampling statistics for model comparisons on the held-out data

To assess the statistical significance of the observed model performance and the differences in performance between the models, we computed resampling statistics of the performance metrics on the held-out generalization data not used for model construction [107]. Once unique subject-wise predictions were obtained on the held-out generalization data by averaging the predictions emanating from each fold of the validation set (CV-bagging), we computed null and bootstrap distributions of the observed test statistic on the held-out data, i.e., *R*^2^ score for regression and AUC score for classification.

##### Baseline comparisons

To obtain a *P*-value for baseline comparisons (“could the prediction performance of a given model be explained by chance?") on the held-out data, we permuted targets 10,000 times and then recomputed the test statistic in each iteration. *P*-values were then defined as the probability of the test statistic under null distribution being larger than the observed test statistic. To compute uncertainty intervals, we used the non-parametric bootstrap method, recomputing the test statistic after resampling 10,000 times with replacement and reporting the 2.5 and 97.5 percentiles of the resulting distribution. Note that this procedure is unrelated to the parametric bootstrap used for the analyses presented in Fig. 2 and supplements (see section “Health-related habits regression").

##### Pairwise comparisons between models

For model comparisons, we considered the out-of-sample difference in *R*^2^ or AUC between any 2 models. To obtain a *P*-value for model comparisons (“could the difference in prediction performance between 2 given models be explained chance?") on the held-out data, for every testing-data point, we randomly swapped the predictions from Model A and Model B 10,000 times and then recomputed the test statistic in each iteration. We omitted all cases for which only predictions from 1 of the models under comparison was present. *P*-values were then defined as the probability of the absolute value of the test statistic under null distribution being larger than the absolute value of the observed test statistic. The absolute value was considered to account for differences in both directions. Uncertainty intervals were obtained from computing the 2.5 and 97.5 percentiles of the non-parametric bootstrap distribution based on 10,000 iterations. Here, predictions from Model A and Model B were resampled using identical resampling indices to ensure a meaningful paired difference. Again, note that this procedure is unrelated to the parametric bootstrap used for the analyses presented in Fig. 2 and supplements (see section “Health-related habits regression").

#### Out-of-sample association between proxy measures and health-related habits

##### Computation of brain age Δ and de-confounding

For association with health-contributing habits (Table 5), we computed the brain age Δ as the difference between predicted age and actual age:

(1) \begin{eqnarray*}
\mathrm{Brain}\ \mathrm{Age}\ \Delta = \mathrm{Age}_{\mathrm{predicted}} - \mathrm{Age}. \end{eqnarray*}

Because age prediction is rarely perfect, the residuals will still contain age-related variance, which commonly leads to brain age bias when relating the brain age to an outcome of interest, e.g., sleep duration [108]. To mitigate leakage of age-related information into the statistical models, we used a de-confounding procedure in line with [109] and [eqs. 6–8] consisting in residualizing a measure of interest (e.g., sleep duration) with regard to age through multiple regression with quadratic terms for age. To minimize computation on the held-out data we first trained a model relating the score of interest to age on the validation set to then derive a dedconfounding predictor for the held-out generalization data. The resulting de-confounding procedure for variables in the held-out data amounts to computing an age-residualized predictor measure_resid_ from the measure of interest (e.g., sleep duration) by applying the following quadratic fit on the validation data:

(2) \begin{eqnarray*} \begin{split} \mathrm{measure}_{\mathrm{validation}} = \mathrm{age}_{\mathrm{validation}} \times \beta _{\mathrm{val}1} + \\ \mathrm{age}_{\mathrm{validation}}^2 \times \beta _{\mathrm{val}2} + \epsilon. \end{split}
\end{eqnarray*}

The deconfounding predictor was then obtained by evaluating the weights β_val1_ and β_val2_ obtained from Equation 2 on the generalization data:

(3) \begin{eqnarray*} \begin{split} \mathrm{deconfounder} = \mathrm{age}_{\mathrm{generalization}} \times \beta _{\mathrm{val}1} \\ + \mathrm{age}_{\mathrm{generalization}} ^ 2 \times \beta _{\mathrm{val}2}. \end{split}
\end{eqnarray*}

We performed this procedure for all target measures to study associations not driven by the effect of age. For supplementary analyses presented in Fig. 2 - Figure supplement 3, the same procedure was applied, substituting age for fluid intelligence and neuroticism, respectively.

**Table 5.** Extra health variables used for correlation analysis with participant-specific predicted scores

Family	EID	Variable
Alcohol*	1568-0.0	Average weekly red wine intake
	1578-0.0	Average weekly champagne plus white wine intake
	1588-0.0	Average weekly beer plus cider intake
	1598-0.0	Average weekly spirits intake
	1608-0.0	Average weekly fortified wine intake
	5364-0.0	Average weekly intake of other alcoholic drinks
Physical activity	22040-0.0	Summed MET minutes per week for all activity
Smoking	20161-0.0	Pack-years of smoking
Sleep	1160-0.0	Sleep duration

*We computed a compound drinking score by summing up all variables from the alcohol family. MET: metabolic equivalent task.

##### Health-related habits regression

We then investigated the joint association between proxy measures of interest and health-related habits (Table 5) using multiple linear regression. For simplicity, we combined all brain imaging and all sociodemographic variables (Fig. 3, Figure 3 - Figure supplement 1, Figure supplement 2, Figure 3). The ensuing model can be denoted as

(4) \begin{eqnarray*}
\mathrm{measure} &=& \mathrm{deconfounder} \times \beta _1 + \mathrm{Brain\ Age}\ \Delta \times \beta _2\nonumber\\ &&\quad +\, \mathrm{PredFluidInt} \times \beta _3 + \mathrm{PredNeurot} \times \beta _4 + \epsilon, \end{eqnarray*}

where “deconfounder" is given by Equation 2. Prior to model fitting, rows with missing inputs were omitted. For comparability, we then applied standard scaling on all outcomes and all predictors.

The parametric bootstrap was a natural choice for uncertainty estimation because we used standard multiple linear regression, which provides a well-defined procedure for mathematically quantifying its implied probabilistic model. Computation was carried out using the “sim" function from the arm package as described in [11, 54] (Ch. 7, pp.142–3). This procedure can be intuitively regarded as yielding draws from the posterior distribution of the multiple linear regression model under the assumption of a uniform prior. For consistency with previous analyses, we computed 10,000 draws.

For supplementary analysis in Fig. 2 - Figure supplement 2, the brain-predicted age instead of the Δ was used:

(5) \begin{eqnarray*}
\mathrm{measure} &=& \mathrm{deconfounder} \times \beta _1 + \mathrm{Brain\ Age} \times \beta _2+\nonumber \\ &&\quad \mathrm{PredFluidInt} \times \beta _3 + \mathrm{PredNeurot} \times \beta _4 + \epsilon , \end{eqnarray*}

For supplementary analysis in Fig. 2 - Figure supplement 3, additional deconfounders were introduced.

(6) \begin{eqnarray*}
\mathrm{measure} &=& \mathrm{deconfounder}_{\mathrm{age}} \times \beta _1\\ && + \mathrm{Brain\ Age} \times \beta _2+ \mathrm{deconfounder}_{\mathrm{FI}}\nonumber\\ && \times \beta_3 + \mathrm{PredFluidInt} \times \beta _4 + \mathrm{deconfounder}_{\mathrm{N}}\nonumber\\ && + \beta _5 + \mathrm{PredNeurot} \times \beta _6 + \epsilon , \end{eqnarray*}

where deconfounder_FI_ is the deconfounder for fluid intelligence and deconfounder_N_ the deconfounder for neuroticism following the procedure described in Equations 2 and 3.

For supplementary analysis in Fig. 2 - Figure supplement 4, proxies and targets were analyzed simultaneously.

(7) \begin{eqnarray*}
\mathrm{measure} &=& \mathrm{Age} \times \beta _1 + \mathrm{Brain\ Age} \times \beta _2+ \mathrm{Fluid\ Intelligence} \times \beta_3 +\nonumber\\ &&\quad \mathrm{PredFluidInt} \times \beta _4 + \nonumber\\ &&\quad \mathrm{Neuroticism} + \beta _5 + \mathrm{PredNeurot} \times \beta _6 + \epsilon . \end{eqnarray*}

### Software

Preprocessing and model building were carried out using Python 3.7. The NiLearn library was used for processing MRI inputs [101]. We used the scikit-learn library for machine learning [110]. For statistical modeling and visualization we used the R language [111] (version 3.5.3) and its ecosystem: data.table for high-performance manipulation of tabular data, ggplot [112, 113] for visualization, and the arm package for parametric bootstrapping [114]. All data analysis code is shared on GitHub [69].

## Availability of Source Code and Requirements

- Project name: empirical_proxy_measures
- Project home page: https://github.com/KamalakerDadi/empirical_proxy_measures
- Operating system(s): Platform independent
- Programming language: Python and R
- Other requirements: Python 3.6.8 or higher, R 3.4.3 or higher
- License: BSD-3

## Data Availability

Aggregated data supporting the results and figures of this article are available through the GigaScience Database [115] and the “empirical_proxy_measures" code repository [69]. In the future, the individual-level proxy measures obtained from the prediction models in this work will be shared in agreement with the UK Biobank regulations; see [69] for details. The input data are available for other researchers via UKBB’s controlled access scheme [116]. The procedure to apply for access [117] requires registering with the UK Biobank and compiling an application form detailing:

- A summary of the planned research
- The UK Biobank data fields required for the project
- A description of derivatives (data, variables) generated by the project

## Additional Files

**Figure 1 – Figure supplement 1**: Learning curves on the random split-half validation used for model building. To facilitate comparisons, we evaluated predictions of age, fluid intelligence and neuroticism from a complete set of socio-demographic variables without brain imaging using the coefficient of determination *R^2^* metric (y-axis) to compare results obtained from 100 to 3000 training samples (x-axis). The cross-validation (CV) distribution was obtained from 100 Monte Carlo splits. Across targets, performance started to plateau after around 1000 training samples with scores virtually identical to the final model used in subsequent analyses. These benchmarks suggest that inclusion of additional training samples would not have led to substantial improvements in performance.

**Figure 2 – Figure supplement 1**: Marginal associations between proxy measures and health-related habits. Marginal (instead of conditional) estimates using univariate regression. Same visual conventions as in Fig. 2.

**Figure 2 – Figure supplement 2:** Conditional associations between proxy measures and health-related habits without explicit brain age delta. Conditional estimates using multivariate regression. Instead of the brain age delta, the brain-predicted age is included alongside an age-deconfounder as used in the main analysis. Same visual conventions as in Fig. 2.

**Figure 2 – Figure supplement 3:** Conditional associations between proxy measures and health-related habits with-proxy-specific deconfounding. Conditional estimates using multivariate regression. Instead of the brain age delta, the brain-predicted age is included alongside an age-deconfounder as used in the main analysis. Moreover, predicted fluid intelligence and neuroticism are deconfounded for the target values at training time, analogous to the brain age predictions. Same visual conventions as in Fig. 2.

**Figure 2 – Figure supplement 4**: Joint modeling of health-related habits from proxy and target measures. Conditional estimates using multivariate regression. Every health-related habit (double rows) is modeled simultaneously from multiple proxies and targets. Same visual conventions as in Fig. 2. Across health- habits, additive effects emerged not only for proxies and targets within the same measure (e.g. age) but also across measures (e.g. age and fluid intelligence). For illustration, we shall consider two examples. Regarding alcohol consumption, age was the most important measure and opposite conditional effects were observed for the proxy and the target: Across the age range, people with higher brain age tended to drink more and across the brain-age range, older people tended to drink less. For smoking, the proxy measures were the most important variables with clear non-zero coefficients, pointing in different directions across target domains. Holding fluid intelligence and neuroticism constant (targets and proxies), people with higher brain age tended to have been smoking for a longer time. At the same time, those who scored lower on predicted fluid intelligence across the entire range of age, predicted age, measured fluid intelligence, predicted neuroticism and neuroticism, have been smoking for a longer time. Finally, those who scored higher on predicted neuroticism tended to smoke more across the ranges of all other measures.

**Figure 3 – Figure supplement 1:** Prediction of individual differences in proxy measures from MRI. Approximation performance using multiple MR modalities on the validation dataset: sMRI, dMRI, rfMRI and their combinations (see Table 1). Visual conventions as in Fig. 3. One can see that prediction of age was markedly stronger than prediction of fluid intelligence or prediction of neuroticism. As a general trend, models based on multiple MRI modalities tended to yield better prediction. For simplicity, we based subsequent analyses on the full model based on all MRI data.

**Figure 3 – Figure supplement 2:** Approximation performance using all sociodemographic data. Approximation performance using all sociodemographic variables with or without brain imaging included on the validation dataset. Visual conventions as in Fig. 3. The performance was highly related to the best performing models within each target Figure 3, i.e., life style for age, education for fluid intelligence and mood & sentiment for neuroticism. This suggests that for each target those specific blocks of predictors were sufficiently explaining the performance. For simplicity, we based subsequent analyses on all sociodemographic variables in Fig. 2, Fig. 3 and Fig. 4.

**Supplementary Figure S1:**Intercorrelations between sociodemographic inputs. To check the plausibility of the proposed grouping of variables into blocks, we investigated the intercorrelations among the sociodemographic inputs (Supplementary Table S7). We first applied Yeo-Johnson power transform to the variables, yielding approximately symmetrical distributions. Then we computed Pearson correlations. One can see that most variables show low if any intercorrelations. Strongly intercorrelated blocks emerged, in particular for Mood and Sentiment and Lifestyle. Note that within the Lifestyle category many smaller blocks with strong intercorrelation occurred, some of which were obviously related to the circumstances of living, such as household or employment status.

**Supplementary Figure S2**: Investigating the age gap between the first visit and the MRI visit time point. (**A**) Individual gap between age at first visit and MRI scan time. MRI scans never happened at the first visit, leading to a strictly positive gap of >5 years for most participants. Pearson correlation coefficient indicates high rank stability, suggesting that, from a statistical perspective, age at first visit and age at scan time are, essentially, interchangeable. (**B**) Direct comparison of individual-specific age predictions from brain images and sociodemographic data. Same model as in the main analysis (Fig. 2). The emerging pattern of association summarized by Pearson correlation coefficient suggests that predictions from models trained on age either at the first visit or at MRI-scan time are equivalent.

**Supplementary Figure S3:**Proxy measures show systematic and complementary out-of-sample associations with health-related habits using age at MRI scan time. The patterns observed in Fig. 2 and global conclusions remain unchanged.

**Supplementary Table S1**: Paired difference between purely sociodemographic and models including brain imaging on held-out data.

**Supplementary Table S2**: Difference statistics for classification on the held-out set for sociodemographic vs combined approximation.

**Supplementary Table S3**: Inferential statistics for joint proxy-target models of health-related habits.

**Supplementary Table S4**: Variance inflation factors (VIF) for joint proxy-target models of health-related habits.

**Supplementary Table S5**: Regression statistics on the held-out set for purely MRI-based approximation.

**Supplementary Table S6**: Classification difference statistics on the held-out set for MRI-based approximation.

**Supplementary Table S7**: List of variables contained in each block of sociodemographic models: Mood and Sentiment (MS), Age, Sex (AS), Education (EDU), Early Life (EL).

## Abbreviations

AUC: area under the classification accuracy curve; ICA: independent component analysis; ICD-10: International Statistical Classification of Diseases and Related Health Problems, 10th Revision; MRI: magnetic resonance imaging; UKBB: UK Biobank; VIF: variance inflation factors.

## Competing Interests

The authors declare that they have no competing interests.

## Funding

D. B. acknowledges funding by the Canadian Institutes of Health Research (438531).

G. V. acknowledges funding by the Canada First Research Excellence Fund.

## Authors' Contributions

- **Conceptualization**: B.T., D.B., D.E., G.V., J.H.
- **Data curation**: D.B., K.D.
- **Software**: B.T., D.E., G.V., K.D.
- **Formal analysis**: D.E., G.V., K.D.
- **Supervision**: B.T., D.E., G.V.
- **Funding acquisition**: G.V., J.H.
- **Validation**: D.E., K.D.
- **Investigation**: D.E., K.D.
- **Visualization**: D.E., G.V., K.D.
- **Methodology**: B.T., D.E., G.V.
- **Project administration**: D.E., G.V.
- **Writing—original draft**: D.E., K.D.
- **Writing—review and editing**: D.B., B.T., D.E., G.V., J.H., K.D.

## Supplementary Material

giab071_GIGA-D-21-00080_Original_Submission

giab071_GIGA-D-21-00080_Revision_1

giab071_GIGA-D-21-00080_Revision_2

giab071_GIGA-D-21-00080_Revision_3

giab071_Response_to_Reviewer_Comments_Original_Submission

giab071_Response_to_Reviewer_Comments_Revision_1

giab071_Response_to_Reviewer_Comments_Revision_2

giab071_Reviewer_1_Report_Original_Submission Bo Cao -- 4/24/2021 Reviewed

giab071_Reviewer_1_Report_Revision_1 Bo Cao -- 7/28/2021 Reviewed

giab071_Reviewer_1_Report_Revision_2 Bo Cao -- 8/12/2021 Reviewed

giab071_Reviewer_2_Report_Original_Submission Hugo Schnack -- 4/27/2021 Reviewed

giab071_Supplemental_File
