# Supplementary material for: Population modeling with machine learning can enhance measures of mental health
Source: Gigascience. 2021 Oct 15;10(10):giab071. doi: 10.1093/gigascience/giab071 (PMC8559220; doi:10.1093/gigascience/giab071)
Supplement: giab071_GIGA-D-21-00080_Revision_1 [file giab071_giga-d-21-00080_revision_1.pdf]

GigaScience

Population modeling with machine learning can enhance measures of mental health

--Manuscript Draft--

**Manuscript Number:**	GIGA-D-21-00080R1
**Full Title:**	Population modeling with machine learning can enhance measures of mental health
**Article Type:**	Research
**Funding Information:**	Canada First Research Excellence Fund — Dr. Gaël Varoquaux<br>Canadian Institutes of Health Research (438531) — Dr Danilo Bzdok
**Abstract:**	Background Biological aging is revealed by physical measures, e.g., DNA probes or brain scans. Instead, individual differences in mental function are explained by psychological constructs, e.g., intelligence or neuroticism. These constructs are typically assessed by tailored neuropsychological tests that build on expert judgement and require careful interpretation. Could machine learning on large samples from the general population be used to build proxy measures of these constructs that do not require human intervention?<br>Results Here, we built proxy measures by applying machine learning on multimodal MR images and rich sociodemographic information from the largest biomedical cohort to date: the UK Biobank. Objective model comparisons revealed that all proxies captured the target constructs and were as useful, and sometimes more useful than the original measures for characterizing real-world health behavior (sleep, exercise, tobacco, alcohol consumption). We observed this complementarity of proxy measures and original measures when modeling from brain signals or sociodemographic data, capturing multiple health-related constructs.<br>Conclusions Population modeling with machine learning can derive measures of mental health from brain signals and questionnaire data, which may complement or even substitute for psychometric assessments in clinical populations.
**Corresponding Author:**	Denis-Alexander Engemann<br>Inria Saclay: Inria Centre de Recherche Saclay-Ile-de-France<br>Palaiseau, FRANCE
**Corresponding Author Secondary Information:**	
**Corresponding Author's Institution:**	Inria Saclay: Inria Centre de Recherche Saclay-Ile-de-France
**Corresponding Author's Secondary Institution:**	
**First Author:**	Kamalaker Dadi
**First Author Secondary Information:**	
**Order of Authors:**	Kamalaker Dadi<br>Gaël Varoquaux<br>Josselin Houenou<br>Danilo Bzdok<br>Bertrand Thirion<br>Denis-Alexander Engemann
**Order of Authors Secondary Information:**	
**Response to Reviewers:**	## Summary<br><br>**Please note that a fully formatted and more readable version of the response to the reviewers (including coloured highlights and the new figures and tables in context) is available as PDF among the submitted files.**

Powered by Editorial Manager® and ProduXion Manager® from Aries Systems Corporation

Please note that we could not fully control the order in which the files were included by the submission system

We would like to thank the editor and the reviewers for the thoughtful feedback and constructive criticism on our work. We have taken the revisions as an important opportunity to improve our manuscript and would like to highlight two points that both reviewers and the editor have emphasized:

1. both reviewers had concerns regarding the far-reaching conclusion: the **findings suggested that psychological constructs can be approximated from brain images and sociodemographic variables.** Considering these concerns, we added methodological and conceptual clarifications, discussed our findings more carefully and revised the general conclusions to express greater nuance.

1. both reviewers also pointed out that the phenotype **age / brain age** is different from the other measurements. We conducted multiple control analysis to investigate this point and clarify the differences, both, conceptually and statistically. The wealth of results and related discussion points are fully reported in the revised manuscript.

This required substantial reanalysis demanding several weeks of computation time and led us to including 6 new supplementary figures and 2 new supplementary tables. A detailed display of the changes in the manuscript is available in the file `diff-with-original-submission.pdf` enclosed with the resubmission.

Please note that, where appropriate, replies are organized by bundles of related concerns, sometimes involving concerns raised by both reviewers.

Reviewer 1

R1.1

The manuscript describes an application of Machine Learning (ML) models for the quantification of psychological constructs, e.g., fluid intelligence and neuroticism, using multi-model MRI data from a large population cohort, the UK biobank data. They show that the proxy measures of these psychological constructs are more useful compared to the original constructs for characterizing health behaviors. Overall, the manuscript is well written. The research questions are clearly stated and are of practical importance. However, the reviewer has following concerns.

Reply to R1.1: We thank the reviewer for the positive appreciation of our work.

R1.2

Major Concerns:

1) In page 3 (left, lines 3-6 of the main text), the author claims that Our findings suggested that psychological constructs can be approximated from brain images and sociodemographic variables - inputs not tailored to specifically measure these constructs.. The reviewer has concerns about this claim. Although Figure 3 shows the models performance in predicting age, fluid intelligence and neuroticism using neuroimaging data and different areas of sociodemographic data, the performance of the models in predicting the psychological constructs, fluid intelligences and neuroticism, may not be good enough to support such a claim.

Reply to R1.2 : We thank the reviewer for pointing out the potential ambiguity of the wording. An approximation can be understood as an almost perfect prediction but also as a crude guess. In fact, we did not intend to suggest that the approximations are perfect. We have now rephrased the above sentence accordingly and, moreover, carefully edited other parts of the main text to help avoid this misunderstanding.

Changes

In abstract (instead of advantage):

Powered by Editorial Manager® and ProduXion Manager® from Aries Systems Corporation

We observed this complementarity of proxy measures and original measures when modeling from brain signals or sociodemographic data, capturing multiple health-related constructs.

In results section title (instead of outperforming):

Complementing the original measures at characterizing real-life health-related habits

In results section:

Our findings suggested that some information on psychological constructs can be assembled from general inputs not specifically tailored to measure these constructs, such as brain images and sociodemographic variables. The resulting proxy measures may be regarded as crude approximations of the psychological measures, but they can nonetheless capture essential aspects of the target constructs. To probe the external validity of the proxy-measures, we investigated their link with real-world behavior, e.g., sleep, physical exercise, alcohol and tobacco consumption on left-out data.

R1.3

2) In Figure 2, the proxy measure and original measure show similar associations with the health phenotypes for fluid intelligence (center plot) and neuroticism (right plot), but not for the brain age delta. The main reason seems to be when doing the association analysis, the measures of the health phenotypes are de-confounded for their dependence for age (In the subsection Out-of-sample association between proxy measures and health-related habits of the statistical analysis section). However, it seems the same procedure is not applied for the association analysis of fluid intelligence and neuroticism. The estimated brain age or brain age gap depends on the age. Thus, we need to either correct the brain age or brain age gap for its dependence on the age, or de-confounded the health phenotypes dependence on age. If the author wants to derive the proxy measure of the psychological construct in the same as the brain age (or biological age), same procedure should be used to correct the proxy measures dependence on the original measure.

AND

R1.4

3) Based on Figure 2, the author claims that the proxy measures have enhanced association with health behavior compared to the original measures. If we only focus on the central and right part of the Figure 2, the difference is not that obvious. We do not know if the difference is significant or not. A better approach maybe is that correct the predicted fluid intelligence and predicted fluid intelligence for their dependence on the original measures or de-confounded the original measures effects on the health behaviors.

Reply to R1.3-R1.4: We thank the reviewer for giving us the opportunity for clarifying our conceptual view and substantially extending the analysis in response to these thoughtful concerns. Note that concerns **R2.2-2.4** by reviewer 2 were highly related. Therefore, the following reply is intended to address the same issue raised by both reviewers **.**

First of all, we believe that the three measures under study are fundamentally distinct and lead to different statistical behaviors, which is the reason for which we included them in this study. Age is a metric measure, related to physical units on which intervals of the same size express the same distance. A delta can thus be a meaningful summary, which, in the case of brain age, has a straight-forward interpretation. For the other measures, ordinal or categorical scales apply; the delta is less convincing as a proxy measure metric here and may have no meaning.

Rather, in our view, what is important is that the predicted measure contains information -- in the widest sense -- beyond the actual measure. Therefore, the popular brain age delta is not necessary here. A multivariate analysis linking both chronological age and predicted age to the outcome of interest can capture the same information

(see new figure-2 supplements 2-4). On the other hand, when inspecting the other proxy measures, we found it important to use an age deconfounder, to confirm that effects were not driven by age, as age shows pervasive correlations with nearly any biobehavioral entity. This de-confounding is important to interpret results as indeed driven by the construct of interest, but associations hold without it or when adding specific deconfounders to all proxies, as we show in new analyses below (see new figure-2 supplements 3-4).

These considerations explain why we have initially processed the proxies in different ways (age-decorrelation for all proxies, delta for brain age only). We understand, however, that rational arguments alone may feel unsatisfying from a readers perspective. We have taken the opportunity to extend our analysis. To probe the robustness of our analysis to these different options we have run several checks which we have included in the supplements (described below).

While implementing these extra checks, we realized that in figure 2B, we had kept the age-deconfounder active when modeling the link between age and the health descriptors, which was not ideal as it may destroy the effect. We have corrected this accordingly and removed the age-deconfounder subsequently from the target measure analyses (see corrected figure 2 below).

New figure 2 after removing the age deconfounder for the target measures (lower panel).

(See formatted reply to the reviewers among submitted files)

New Supplementary analyses.

Figure 2 supplement 2: We ran the same analysis for figure 2 using the brain-predicted age as such, not the delta as the proxy measure. Outputs are virtually identical.

Figure 2 supplement 3: We then added de-confounders for fluid intelligence and neuroticism and repeated the analysis for figure 2. Outputs were highly similar.

Figure 2 supplement 4: To provide a more rigorous comparison between proxies and targets as stimulated in **R1.4** and assess statistical significance of possible differences between proxies and targets, we have composed a big regression model including both the proxies and the targets as predictors. Across health outcomes, we found two frequent scenarios: 1) the proxies were found statistically significant, not the actual measures, suggesting that the proxies show improved SNR over the actual measures. 2) proxies and actual measures were found statistically significant, pointing at true additive effects. The overall picture depended on the health outcome.

Table S3: Detailed statistical inferences for the comprehensive proxy-target models are listed in this new table.

Table S4 : In this context, the intrinsic link between proxies and targets may imply multicollinearity. Too much multicollinearity could potentially render the statistical testing procedure invalid. To test for multicollinearity we computed variance inflation factors (VIF) across the different models, which intuitively quantify how well each predictor can be approximated by a linear combination of the other predictors. The VIF is defined as 1 / (1 - R^2_i), where R^2_i is the coefficient of determination for the model that approximates the predictor i from all remaining predictors. A VIFs 1-5 counts as low to moderate (see extensive review and discussion in Obrien 2007, _Quality & Quantity_ 41, 673-690). The observed VIFs were mostly around 1. The highest VIF was found for age and brain age with values around 3, which suggests that age and brain age are linked to the other proxy measures. This is implicitly justifying our previous intuition of keeping age-deconfounders for the other proxy measures. More importantly, these control analyses suggest that the significance tests are not impacted by multicollinearity, which is also in line with reasonable standard errors produced by the big models from S3.

Based on these new analyses, we have updated the results section and have

Powered by Editorial Manager® and ProduXion Manager® from Aries Systems Corporation

highlighted the theoretical considerations regarding asymmetries between the proxy measures.

Changes

In the introduction, the asymmetry has been emphasized:

Extrapolating from these successes, we propose to build upon large datasets to extend the collection of health-related proxy measures, probing mental traits.For this end, we focused on constructs fundamentally different in terms of content and methodology.

In the main text, result section, a novel paragraph has been added to address the conceptual difference of the measures and consequences for the analysis:

The three proxy measures are difficult to compare on an equal footing as a delta was considered for brain age only (the difference between predicted and actual age) and aging- specific deconfounding was applied. The brain-age delta is indeed the standard practice, theoretically justified as age is on a metric scale [50] for which the difference between the predicted and the measured value has a clear meaning. Such a difference is less obvious for variables with ordinal scales as implied by psychometric measures. Second, age has a pervasive influence on virtually any biomedical entity, which motivates controlling for its effect on proxy measures. To rule out that differences in proxy measures associations to health-related behavior are driven by this methodological asymmetry, we repeated the main analysis from Figure 2, first, using the predicted age without computing the delta (Figure 2 – Figure supplement 2) and, second, introducing additional deconfounders for fluid intelligence and neuroticism (Figure 2 – Figure supplement 3). The resulting patterns were virtually unchanged, confirming that interpretations are robust.

In the main text, result section, a paragraph on big-picture statistical differences between proxies and targets has been generously expanded:

A question that remains is whether the proxy measures bring additional value compared to the original target measures they were derived from. These original target measures showed similar associations to health behavior, with the same signs in most cases (Figure 2, B). At the same time, the ensuing patterns were more noisy, suggesting that empirically derived proxy measures yielded enhanced associations with health behavior. This inference may be difficult as differences between targets and proxies were not always easy to pinpoint visually. To implement a more rigorous statistical approach, we built comprehensive models of each respective health-related habit in which we used all proxies (predicted age, predicted fluid intelligence, predicted neuroticism) and all targets (age, fluid intelligence, neuroticism) simultaneously as predictors (Figure 2 – Figure supplement 4). The results show systematic additive effects of proxies and targets across the three target domains and the four health-habits. These trends are well-captured by the hypothesis tests of the respective linear models (Table S3). As targets and proxies may be systematically intercorrelated, multicollinearity may corrupt these inferences. Inspection of variance inflation factors (VIF)— a measure that reveals how well a given predictor can be approximated by a linear combi- nation of the other predictors— argued in favor of low to moderate levels of multicollinearity (Table S4). Indeed, all VIF values fell between 3 and 1, whereas, classically, values above 5 or 10 are considered as thresholds [51] for pathological collinearity. This suggests that the model inferences are statistically sound.

In the main text, method section, health-related habits regression, a passage has been added describing the additional models (see blue highlight in screenshot).

(See formatted reply to the reviewers among submitted files)

The following items have been added to the supplement:

(See formatted reply to the reviewers among submitted files)

Figure 2 supplement 2: Conditional associations between proxy measures and health-related habits without explicit brain age delta. Conditional estimates using

multivariate regression. Instead of the brain age delta, the brain-predicted age is included alongside an age-deconfounder as used in the main analysis. Same visual conventions as in Figure 2.

(See formatted reply to the reviewers among submitted files)

Figure 2 supplement 3 Conditional associations between proxy measures and health-related habits with-proxy-specific deconfounding. Conditional estimates using multivariate regression. Instead of the brain age delta, the brain-predicted age is included alongside an age-deconfounder as used in the main analysis. Moreover, predicted fluid intelligence and neuroticism are deconfounded for the target values at training time, analogous to the brain age predictions. Same visual conventions as in Figure 2.

(See formatted reply to the reviewers among submitted files)

Figure 2 supplement 4 Joint modeling of health-related habits from proxy and target measures. Conditional estimates using multivariate regression. Every health-related habit (double rows) is modeled simultaneously from multiple proxies and targets. Same visual conventions as in Figure 2. Across health-habits, additive effects emerged not only for proxies and targets within the same measure (e.g. age) but also across measures (e.g. age and fluid intelligence).

For illustration, we shall consider two examples. Regarding alcohol consumption, age was the most important measure and opposite conditional effects were observed for the proxy and the target: Across the age range, people with higher brain age tended to drink more and across the brain-age range, older people tended to drink less. For smoking, the proxy measures were the most important variables with clear non-zero coefficients, pointing in different directions across target domains. Holding fluid intelligence and neuroticism constant (targets and proxies), people with higher brain age tended to have been smoking for a longer time. At the same time, those who scored lower on predicted fluid intelligence across the entire range of age, predicted age, measured fluid intelligence, predicted neuroticism and neuroticism, have been smoking for a longer time. Finally, those who scored higher on predicted neuroticism tended to smoke more across the ranges of all other measures.

Supplementary table 3

(See formatted reply to the reviewers among submitted files)

Supplementary table 4

(See formatted reply to the reviewers among submitted files)

Minor concerns:

R1.5

1) In page 1 (two lines before reference 15), it seems that to learn is mis-spelled into tolearn.

Reply R1.5: We thank the reviewer for having found this typo. We have corrected it.

R1.6

2) The author stated that there are repeated measures for subjects in UK biobank data. How the author tackles this issue in their data preprocessing? Using the last one or the first one or something else?

Reply R1.6 We thank reviewers for having raised this concern whose clarification may, indeed, help the reader.

Out of 500 000 participants, only a fraction has visited for repeated assessments. For instance, 10 000 participants were included in the first brain imaging release (Miller K

et al. Nat. Neuroscience 2016; Thomas J. Littlejohns et al. Nat. Communications 2020). The visits are categorized as: initial assessment visit, repeated initial assessment, imaging visit and soon. These visits are coded as 0, 1, 2 and soon. In this paper, we relied mostly on assessments overlapping with imaging visit _i.e._ variables coded as 2.0 as can be seen from Table S5, _Appendix_ 2.

Some variables used in the analysis display code 0, _e.g._ for target variables like age (21022-0.0), neuroticism (20127-0.0) or external validity variables. The reason for this is that these variables were not assessed during the follow up visits. We, therefore, relied on primary visit information.

Moreover, we explicitly checked that there is no overlap of the subjects between the validation and the generalization set.

Changes To clarify this point, we extended the subsection _Participants_.

(...) we found 11175 participants who had repeated assessments overlapping with the first brain imaging release. Note that the features (sociodemographic variables) that we included in the analysis are measures that are self-reported during a follow-up imaging visit. (...) We made sure that the subjects used for model construction and generalization were strictly non-overlapping.

R1.7

3) The selection 5,587 out of all the 10,975 subjects for the modeling, while the left part is for the out-of-sample association analysis. The selection seems arbitrary. Can the author also show a learning curve, in which x is the sample size and y is the models performance, to justify their choice is enough to train an accurate ML model?

R1.7 We thank reviewers for allowing us to clarify how we composed the training dataset.

First, we selected a percentage of training samples based on the random split half method from the scikit-learn package. As sample size differed across three targets, e.g., fewer brain images available for neuroticism prediction (see second paragraph at the subsection _Participants_) testing the out-of-sample associations on 50% of gathered samples was a pragmatic approach for obtaining sufficient data for model training and subsequent statistical inference, two complementary and distinct objectives in this investigation. Moreover, this is in line with evidence arguing in favor of larger test sets for mitigating optimism bias in estimation of out-of-sample performance (Flint et al 2021, Nat. Neuropsychopharmacology, Varoquaux et al. 2017, NeuroImage).

To explore the implications of our training and testing splits, as suggested in, we computed learning curves (displayed below). Across all targets, the results suggest that generalization performance started saturating around 1000 training samples, well below the final training samples. Adding more samples should not substantially improve generalization performance.

Changes To clarify this point, we have included a new figure as a supplement to the concept figure 1 and extended the method section on participants.

In the main text, methods, section Participants:

The demographics are 51.6% female (5\,572) and 48.3% male (5\,403) and an age range between 40-70 years (with a mean of 55 years and standard deviation of 7.5 years). The data for model training were selected using a randomized split-half procedure yielding 5\,587 individuals. The remaining subjects were set aside as a held-out set for generalization testing (see section _Model development and generalization testing_). We made sure that the subjects used for model training and generalization testing were strictly non-overlapping. Learning curves documented that the training split was sufficiently large for constructing stable prediction models (Figure 1 - supplement 1) with profiles of performance similar to latest benchmarks on model complexity in the UK Biobank (Schulz et al 2020). Moreover, simulations and empirical

Powered by Editorial Manager® and ProduXion Manager® from Aries Systems Corporation

findings suggest that larger testing sets are more effective at mitigating optimistic performance estimates (Flint et al 2021, Varoquaux et al. 2017). Together, this provided a pragmatic solution to the inference-prediction dilemma (Bzdok et al 2020; Bzdok et al., 2018) given the two objectives of the present investigation to obtain reasonably good predictive models, while at the same time performing parameter inference of statistical models developed on the left-out data.

(See formatted reply to the reviewers among submitted files)

Figure 1 supplement 1 Learning curves on the random split-half validation used for model building. To facilitate comparisons, we evaluated predictions of age, fluid intelligence and neuroticism from a complete set of socio-demographic variables without brain imaging using the coefficient of determination R^2 metric (y-axis) to compare results obtained from 100 to 3000 training samples (x-axis). The cross-validation (CV) distribution was obtained from 100 Monte Carlo splits. Across targets, performance started to plateau after around 1000 training samples with scores virtually identical to the final model used in subsequent analyses. These benchmarks suggest that inclusion of additional training samples would not have led to substantial improvements in performance.

R1.8

4) In the first paragraph of the Methods section, there are duplications.

Reply **R1.8:** We thank the reviewer for having pointed out the duplications. We have found and removed them.

R1.9

5) In the subsection of Data acquisition part, under the target measures paragraph, the age at the baseline recruitment is used as the outcome. However, in general, there is a gap between the age at baseline and the age when the MRI images were acquired. Does this matter for the data analysis in this manuscript.

Reply R1.9: We thank the reviewer for this thoughtful remark as we had not considered this issue in the first place. To investigate this age gap, we computed a distribution of the individual differences between the age at recruitment and the age at MRI-scan time. As expected, the difference was strictly positive, meaning that the MRI scan was always acquired after the first visit. The individual differences were highly rank-stable, suggesting that the age variables at recruitment and scan-time were equivalent from a statistical perspective. We subsequently fitted the prediction models from the main analysis using age at the time of the MRI-scan and directly compared the individual-specific predictions. As hypothesized, they were virtually identical. We have now made this point explicit in the main text and added a supplementary figure.

Changes

In methods, data acquisition, target measures:

In the course of this work, a question that emerged concerned the size of the gap between age at baseline recruitment and MRI-scan time and its potential impact on the analysis. Supplementary checks indicated that the age gap was at least 5 years for most participants. Yet, from a statistical perspective, the two age measures turned out highly interchangeable (Figure S2) and global conclusions remained unchanged (Figure S3).

(See formatted reply to the reviewers among submitted files)

Figure S2 Investigating the age gap between the first visit and the MRI-visit time point. **(A)** Individual gap between age at first visit and MRI-scan time. MRI scans never happened at the first visit, leading to a strictly positive gap greater than five years for most participants. Pearsons correlation coefficient indicates high rank stability, suggesting that, from a statistical perspective, age at first visit and age at scan time are, essentially, interchangeable. **(B)** Direct comparison of individual-specific age

Powered by Editorial Manager® and ProduXion Manager® from Aries Systems Corporation

predictions from brain images and sociodemographic data. Same model as in the main analysis (Figure 2). The emerging pattern of association summarized by Pearsons correlation coefficient suggests that predictions from models either trained on age at the first visit or at MRI-scan time are equivalent.

(See formatted reply to the reviewers among submitted files)

Figure S3 Proxy measures show systematic and complementary out-of-sample associations with health-related habits using age at MRI-scan time. The patterns observed in Figure 2 and global conclusions remain unchanged.

R1.10

6) For the classification analysis (paragraph Classification analysis in the subsection of Comparing predictive models to approximate target measures, and the paragraph above the Discussion section), the thresholds selected to discretize the outcome variables are kind of arbitrary.

Reply R1.10: The division into groups follows the practical recommendations by Gelman and Hill 2007 for dichotomizing variables. The idea is to avoid having too many cases near the median boundary (where differences should be very subtle). We have now explained the idea and referenced Gelman and Hills book.

Changes

In results, the relative importance of brain and sociodemographic data depends on the target:

To investigate empirically-defined proxy measures beyond continuous regression, we performed binary classification of extreme groups obtained from discretizing the targets using the 33rd and 66th percentiles, following the recommendations by Gelman and Hill (2006) regarding discrete variable encoding strategies.

In method section, predictive model, classification analysis:

We also performed classification analysis on the continuous targets. Adapting recommendations from Gelman and Hill 2005, we performed discrete variable encoding of the targets leading to extreme groups based on the 33rd and 66th percentiles (see

Table 4) for the number of classification samples per group). This choice avoids including samples near the average outcome for which the input data may be indistinct.

Reviewer 2

All in all, this is a scientifically interesting study, but I think the presentation could be improved, by more clearly stating the aims of it, and by giving more insight in certain aspects of the proxy modeling.

We thank the reviewer for the positive appreciation of our work and the constructive criticism. In response to this concern, we have carefully edited the main text, extended the discussion and taken efforts to make our theoretical standpoint more explicit.

R2.1

This manuscript reports on the results of a study that can be split into two parts. For this, it should be noted that the authors consider three categories of quantities. The first category are the input data, or predictors: (a) variables derived from MRI scans and (b) rich sociodemographic variables. The second category, or target variables, as the authors call them, include: (a) age, (b) fluid intelligence and (c) neuroticism. In the first part of the study, using machine learning, predictive models are built to predict the target variables from the input variables. The resulting predictions are called proxy measures. For the second stage, a third category of variables is included, the real world health behaviours, such as alcohol use and physical activity. The authors now

Powered by Editorial Manager® and ProduXion Manager® from Aries Systems Corporation

set out to predict these measures of behaviour based on the measures of the second category, either the real ones or the proxies. Thus, the question is, can alcohol use be better predicted by neuroticism determined from a questionnaire, or by the neuroticism proxy derived from MRI and sociodemographics? The main results are presented in Figure 2, and the conclusion made by the authors is that the proxies perform better than the real measures.The authors carry out additional analyses, including the study of the relative importance of MRI and sociodemographics. The authors suggest that these proxies may have clinical use in the future.

At first sight it may seem surprising that proxies perform better then the real measure in capturing the associations, but, as the authors mention, the real measures suffer from (measurement) noise and non-objectivity. However, the proxies are biased (in the sense of being to simple) and are thus less capable of modeling the (true) individual variation. I would have expected a more in depth discussion about this.

Reply to R2.1: We thank the reviewer for sharing this thoughtful impression with us. We now realize that the current wording of the paper may have led to the impression that we see the proxies as a drop-in-replacement for the actual targets (see also **R1.2** and **R1.4).** Instead, we propose that the proxies can be flexibly used to complement the original measures or act as replacements when the original measures are not available. Of note, in the context of machine learning, biased models often lead to better predictions as it can reduce uncertainty as in the classical bias/variance tradeoff. We have now extended the main text to make our perspective clearer to the reader and avoid this kind of misunderstanding and extended the discussion to better explain the potential mechanism by which proxy measures function.

Changes

In the discussion:

A more complete view on how the proxy measures capture mental-health constructs emerges from their associations with real-world behavior (Figure 2). Indeed, the associations with proxy measures (Figure 2 A) were less noisy and more consistent than with the target measures (Figure 2 B), regardless of their approximation quality. This may seem surprising at first, but the target measures are themselves noisy and of imperfect validity. These measures correspond to traditional tests which, in practice, must be interpreted by an expert, actively confronting their output with broader information on the individual. For instance, IQ scores are typically normalized across age groups. However, extending such a normalization approach to many factors (socio-economic status, culture, gender) poses fundamental high-dimensional statistics challenges. Conversely, using machine learning to assemble proxy measures by mapping the targets to rich sociodemographic and brain data implicitly contextualizes them. In this respect, the resulting measures capture more general signal than the original tests. Here, machine learning could be seen as mimicking the work of a mental health expert who carefully compares psychometric results with other facts known about an individual and its reference population.

Conclusion:

In population studies of mental health, individual traits are captured via lengthy assessments, tailored to specific brain and psychological constructs. We have shown that proxy measures built empirically from general-purpose data can capture these constructs and can improve upon traditional measures when studying real-world health patterns. Proxy measures can make psychological constructs available to broader, more ecological studies building on large epidemiological cohorts or real-world evidence. This can make the difference where psychological constructs are central to developing treatment and prevention strategies, but direct measures have not been collected.

Related changes reported above in response to R 1.2

In abstract (instead of advantage):

We observed this complementarity of proxy measures and original measures when

modeling from brain signals or sociodemographic data, capturing multiple health-related constructs.

In results section title (instead of outperforming):

Complementing the original measures at characterizing real-life health-related habits

In results section:

Our findings suggested that some information on psychological constructs can be assembled from general inputs not specifically tailored to measure these constructs, such as brain images and sociodemographic variables. The resulting proxy measures may be regarded as crude approximations of the psychological measures, but they can nonetheless capture essential aspects of the target constructs. To probe the external validity of the proxy-measures, we investigated their link with real-world behavior, e.g., sleep, physical exercise, alcohol and tobacco consumption on left-out data.

R2.2

Apart from this, there is an asymmetry in the way age is treated as compared to the other two target variables, intelligence and neuroticism. Age is a very hard measure, without any measurement error, and independent of the brain.

AND

R2.3

The other two targets, intelligence and neuroticism, are softer measures, and directly related to the brain. How does this influence the analyses and the results?

AND

R2.4

Indeed, not predicted age is used as proxy, but brain age delta. I would have liked to see more explanation and discussion about this.

Reply to **R2.2-2.4:** We thank the reviewer for sharing these thoughtful concerns. The reviewer is absolutely right that we have treated the target variables differently, as they are fundamentally different in terms of data generating mechanisms and measurement scale. The difference between target and proxy, e.g., the delta, can make sense where one deals with physical units (e.g. years for time) but may make less sense for ordinal or categorical variables (the effective measurement scale for intelligence and neuroticism is less clear; it is only clear that there are no physical units).

These points have also been brought up by reviewer 1 and that the reply, the edits on the manuscript and the additional analyses in response **R1.3-1.4** were explicitly designed to also address concerns **R2.2-2.4**.

Moreover, we have revised the main text to make this asymmetry even more explicit, borrowing the proposed terminology of hard and soft measures. Finally, we have presented evidence in the main text that the proposed framework is flexible enough to handle these intrinsic differences. It turned out that very similar results are obtained even when the three measures are not treated asymmetrically but identically.

New Supplementary analyses.

Figure 2 supplement 2: We ran the same analysis for figure 2 using the brain-predicted age as such, not the delta as the proxy measure. Outputs are virtually identical.

Figure 2 supplement 3: We then added de-confounders for fluid intelligence and neuroticism and repeated the analysis for figure 2. Outputs were highly similar.

Powered by Editorial Manager® and ProduXion Manager® from Aries Systems Corporation

Figure 2 supplement 4: To provide a more rigorous comparison between proxies and targets as stimulated in **R1.4** and assess statistical significance of possible differences between proxies and targets, we have composed a big regression model including both the proxies and the targets as predictors. Across health outcomes, we found two frequent scenarios: 1) the proxies were found statistically significant, not the actual measures, suggesting that the proxies show improved SNR over the actual measures. 2) proxies and actual measures were found statistically significant, pointing at true additive effects. The overall picture depended on the health outcome.

Table S3: Detailed statistical inferences for the comprehensive proxy-target models are listed in this new table.

Table S4 : In this context, the intrinsic link between proxies and targets may imply multicollinearity. Too much multicollinearity could potentially render the statistical testing procedure invalid. To test for multicollinearity we computed variance inflation factors (VIF) across the different models, which intuitively quantify how well each predictor can be approximated by a linear combination of the other predictors. The VIF is defined as 1 / (1 - R^2_i), where R^2_i is the coefficient of determination for the model that approximates the predictor i from all remaining predictors. A VIFs 1-5 counts as low to moderate (see extensive review and discussion in Obrien 2007, _Quality & Quantity_ 41, 673-690). The observed VIFs were mostly around 1. The highest VIF was found for age and brain age with values around 3, which suggests that age and brain age are linked to the other proxy measures. This is implicitly justifying our previous intuition of keeping age-deconfounders for the other proxy measures. More importantly, these control analyses suggest that the significance tests are not impacted by multicollinearity, which is also in line with reasonable standard errors produced by the big models from S3.

Based on these new analyses, we have updated the results section and have highlighted the theoretical considerations regarding asymmetries between the proxy measures.

Related changes reported above in response to reviewer 1

In the interest of a concise reply, only related changes to the text are reprinted below. For the related figures and tables, please consider the reply to points **R1.3-1.4** above.

In the introduction, the asymmetry has been emphasized:

Extrapolating from these successes, we propose to build upon large datasets to extend the collection of health-related proxy measures, probing mental traits.For this end, we focused on constructs fundamentally different in terms of content and methodology.

In the main text, result section, a novel paragraph has been added to address the conceptual asymmetry of the measures and consequences for the analysis:

The three proxy measures are difficult to compare on an equal footing as a delta was considered for brain age only (the difference between predicted and actual age) and aging-specific deconfounding was applied. The brain-age delta is in- deed the standard practice, theoretically justified as age is on a metric scale [50] for which the difference between the predicted and the measured value has a clear meaning. Such a difference is less obvious for variables with ordinal scales as im- plied by psychometric measures. Second, age has a pervasive influence on virtually any biomedical entity, which motivates controlling for its effect on proxy measures. To rule out that differences in proxy measures associations to health-related behavior are driven by this methodological asymmetry, we repeated the main analysis from Figure 2, first, using the predicted age without computing the delta (Figure 2 – Figure supplement 2) and, second, introducing additional deconfounders for fluid intelligence and neuroticism (Figure 2 – Figure supplement 3). The resulting patterns were virtually unchanged, confirming that our interpretations are robust.

In the main text, result section, a paragraph on big-picture statistical differences

Powered by Editorial Manager® and ProduXion Manager® from Aries Systems Corporation

between proxies and targets has been generously expanded:

A question that remains is whether the proxy measures bring additional value compared to the original target measures they were derived from. These original target measures showed similar associations to health behavior, with the same signs in most cases (Figure 2, B). At the same time, the ensuing patterns were more noisy, suggesting that empirically derived proxy measures yielded enhanced associations with health behavior. This inference may be difficult as differences between targets and proxies were not always easy to pinpoint visually. To implement a more rigorous statistical approach, we built comprehensive models of each respective health-related habit in which we used all proxies (predicted age, predicted fluid intelligence, predicted neuroticism) and all targets (age, fluid intelligence, neuroticism) simultaneously as predictors (Figure 2 – Figure supplement 4). The results show systematic additive effects of proxies and targets across the three target domains and the four health-habits. These trends are well-captured by the hypothesis tests of the respective linear models (Table S3). As targets and proxies may be systematically intercorrelated, multicollinearity may corrupt these inferences. Inspection of variance inflation factors (VIF)— a measure that reveals how well a given predictor can be approximated by a linear combination of the other predictors— argued in favor of low to moderate levels of multicollinearity (Table S4). Indeed, all VIF values fell between 3 and 1, whereas, classically, values above 5 or 10 are considered as thresholds [51] for pathological collinearity. This suggests that the model inferences are statistically sound.

R2.5

Finally, the suggested clinical use of the proxies is not supported well enough in my opinion. Maybe the authors could add more this discussion to this point as well.

We thank the reviewer for this suggestion. We absolutely agree with this impression. Unfortunately, relevant clinical data have not been available in the UK-Biobank. At the same time, the focus here is a public health perspective targeting individual differences in health, not pathology. We believe that, beyond the exact measures studied here, the framework, theory and methods proposed in this work can be readily applied with other measures and applied in the clinical setting, which is something that needs to be done in forthcoming studies. We have extended the discussion acknowledging this limitation in the dedicated section.

Changes

In limitations:

In terms of mental-health research, this study falls short of directly testing the clinical relevance of estimated proxy measures. Even in a very large general-population cohort such as the UK Biobank, there are only a few hundred diagnosed cases of mental disorders (ICD-10 mental-health diagnoses from the F chapter) with brain-imaging data available. As a result, we could not directly assess the performance of proxy measures in clinical populations.

The low number of diagnosed mental disorders in UK Biobank highlights the practical importance of studying mental health as a continuous, in addition to diagnosed conditions. Indeed, a public health perspective calls for targeting individual differences in health, not only pathology. Psychological constructs such as IQ and neuroticism are important factors of the epidemiology of psychiatric disorders [38, 30, 29, 67], and accelerated brain aging is associated with various neurological conditions [18, 17, 25]. Yet, few cohorts come with extensive neuropsychological testing. Validated proxies of these constructs open the door to including them in epidemiological studies as secondary outcomes or additional explanatory variables.

Related changes reported above in earlier response to related concerns by reviewer 1

In the discussion:

A more complete view on how the proxy measures capture mental-health constructs

	emerges from their associations with real-world behavior (Figure 2). Indeed, the associations with proxy measures (Figure 2 B) were less noisy and more consistent then with the target measures (Figure 2 A), regardless of their approximation quality. This may seem surprising at first, but the target measures are themselves noisy and of imperfect validity. These measures correspond to traditional tests which, in practice, must be interpreted by an expert, actively confronting their output with broader information on the individual. For instance, IQ scores are typically normalized across age groups. Extending such normalization approach to many factors (socio-economic status, culture, gender) poses however fundamental challenges of high-dimensional statistics. Conversely, using machine learning to assemble proxy measures by mapping the targets to rich sociodemographic and brain data implicitly contextualizes them. In this respect, the resulting measure captures more general signal than the original tests. Here, machine learning could be seen as mimicking the work of a mental health expert who carefully compares psychometric results with other facts known about an individual and its reference population.<br><br>Conclusion:<br><br>In population studies of mental health, individual traits are captured via lengthy assessments, tailored to specific brain and psychological constructs. We have shown that proxy measures built empirically from general-purpose data can capture these constructs and can improve upon traditional measures when studying real-world health patterns. Proxy measures can make psychological constructs available to broader more ecological studies building on large epidemiological cohorts or real-world evidence. This can make the difference where psychological constructs are central to developing treatment and prevention strategies, but direct measures have not been collected.
**Additional Information:**	
**Question**	**Response**
Are you submitting this manuscript to a special series or article collection?	No
**Experimental design and statistics**<br><br>Full details of the experimental design and statistical methods used should be given in the Methods section, as detailed in our Minimum Standards Reporting Checklist. Information essential to interpreting the data presented should be made available in the figure legends.<br><br>Have you included all the information requested in your manuscript?	Yes
**Resources**<br><br>A description of all resources used, including antibodies, cell lines, animals and software tools, with enough information to allow them to be uniquely identified, should be included in the Methods section. Authors are strongly	Yes

Powered by Editorial Manager® and ProduXion Manager® from Aries Systems Corporation

encouraged to cite Research Resource Identifiers (RRIDs) for antibodies, model organisms and tools, where possible.<br><br>Have you included the information requested as detailed in our Minimum Standards Reporting Checklist?	
**Availability of data and materials**<br><br>All datasets and code on which the conclusions of the paper rely must be either included in your submission or deposited in publicly available repositories (where available and ethically appropriate), referencing such data using a unique identifier in the references and in the "Availability of Data and Materials" section of your manuscript.<br><br>Have you have met the above requirement as detailed in our Minimum Standards Reporting Checklist?	Yes

Manuscript Click here to access/download;Manuscript;manuscript_r1.pdf

OXFORD (GIGA)^n SCIENCE

GigaScience, 20xx, 1–24

doi: xx.xxxx/xxxx
Manuscript in Preparation
Paper

PAPER

Population modeling with machine learning can enhance measures of mental health

Kamalaker Dadi[1], Gaël Varoquaux[1,2,3], Josselin Houenou[4,5], Danilo Bzdok[1,3,6], Bertrand Thirion[1] and Denis Engemann[1,7,*]

[1]Inria, CEA, Neurospin, Parietal team, Univ. Paris Saclay, 91120 Palaiseau, France and [2]Montréal Neurological Institute, McGill University, Montreal, Canada and [3]Mila - Quebec Artificial Intelligence Institute, Canada and [4]CEA, NeuroSpin, Psychiatry Team, UNIACT Lab, Univ. Paris Saclay and [5]APHP, Mondor University Hospitals, Psychiatry Dept, INSERM U955 Team 15 "Translational Psychiatry", Créteil, France and [6]Department of Biomedical Engineering, Montreal Neurological Institute, Faculty of Medicine, McGill University, Montreal, Canada and [7]Department of Neurology, Max Planck Institute for Human Cognitive and Brain Sciences, Germany

[*] denis.engemann@gmail.com

Abstract

Background Biological aging is revealed by physical measures, *e.g.*, DNA probes or brain scans. Instead, individual differences in mental function are explained by psychological constructs, e.g., intelligence or neuroticism. These constructs are typically assessed by tailored neuropsychological tests that build on expert judgement and require careful interpretation. Could machine learning on large samples from the general population be used to build proxy measures of these constructs that do not require human intervention? **Results** Here, we built proxy measures by applying machine learning on multimodal MR images and rich sociodemographic information from the largest biomedical cohort to date: the UK Biobank. Objective model comparisons revealed that all proxies captured the target constructs and were as useful, and sometimes more useful than the original measures for characterizing real-world health behavior (sleep, exercise, tobacco, alcohol consumption). We observed this complementarity of proxy measures and original measures when modeling from brain signals or sociodemographic data, capturing multiple health-related constructs. **Conclusions** Population modeling with machine learning can derive measures of mental health from brain signals and questionnaire data, which may complement or even substitute for psychometric assessments in clinical populations.

Key words: Mental Health, Proxy Measures, Machine Learning, Sociodemographic Factors, Brain Imaging

Background

Quantitative measures of mental health remain challenging despite substantial efforts [1]. The field has struggled with unstable diagnostic systems [2], small sample sizes [3], and reliance on case-control studies [4]. Perhaps most importantly, mental health cannot be measured the same way diabetes can be assessed through plasma levels of insulin or glucose. Psychological constructs, *e.g.*, intelligence or anxiety, can only be probed indirectly through lengthy expert-built questionnaires or structured examinations by a specialist. Though questionnaires often remain the best accessible option, their capacity to measure a construct is limited [5]. In practice, as full neuropsychological evaluation is not automated process but relies on expert judgement to confront multiple answers and interpret them in the context of the broader picture, such as cul-

Compiled on: July 14, 2021.
Draft manuscript prepared by the author.

Key Points

- We applied machine learning on more than 10.000 individuals from the general population to define empirical approximations of health-related psychological measures that do not require human judgment.
- We found that machine-learning enriched the given psychological measures via approximation from brain and sociodemographic data: Resulting proxy measures related as well or better to real-world health behavior than the original measures.
- Model comparisons showed that sociodemographic information contributed most to characterizing psychological traits beyond aging.

tural background of the participant. While the field of psychometrics has thoroughly studied the validity of psychological constructs and their measure [6, 7, 8], the advent of new biophysical measurements of the brain brings new promises [9, 10, 11]. The growth of biobanks and advances in machine learning open the door to large-scale validation of psychological measures for mental health research [12], and the hope to develop more generalizable models [13]. Yet, to be reliable, machine learning needs large labeled datasets [14]. Its application to learn imaging biomarkers of mental disorders is limited by the availability of large cohorts with high-quality neuropsychiatric diagnosis [15].

By comparison, it is easier to collect data on the general population without information on clinical conditions. For brain health, such data has lead to developing proxy measures that quantify biological aging [16, 17, 18, 11, 19, 20, 21, 22]. One counterintuitive aspect of the methodology is that measures of biological aging can be obtained by focusing on the age of a person, which is known in advance and in itself not interesting. Yet, by predicting the age, machine-learning can capture the relevant signal. Based on a population of brain images, it extracts the best guess for the age of a person, indirectly positioning that person within the population. Individual-specific prediction errors therefore reflect deviations from what is statistically expected [23]. The brain of a person can look similar to the brains commonly seen in older (or younger) people. The resulting brain-predicted age reflects physical and cognitive impairment in adults [24, 17, 16] and reveals neurodegenerative processes [22, 25]. Can this strategy of biomarker-like proxy measures be extended to other targets beyond the construct of aging? Extrapolating from these successes, we propose to build upon large datasets to extend the collection of health-related *proxy measures*, probing mental traits. For this end, we focused on constructs fundamentally different in terms of content and methodology.

One high-stake target is intelligence, which is measured through socially administered tests and is one of the most extensively studied constructs in psychology. Fluid intelligence refers to the putatively culture-free, heritable and physiological component of intelligence [26, 27] and is a latent construct designed to capture individual differences in cognitive capacity. It has been robustly associated with neuronal maturation and is typically reflected in cognitive-processing speed and working-memory capacity [28]. Applied to psychiatric disorders, it may help characterize psychosis, bipolar disorder, and substance abuse [29, 30].

Neuroticism is a second promising target. As a key representative of the extensively studied Big Five personality inventory, neuroticism has a long-standing tradition in the psychology of individual differences [31, 32]. Neuroticism is measured using self-assessment questionnaires and conceptualized as capturing dispositional negative emotionality including anxiety and depressiveness [33]. It has been inter-culturally validated [26, 34] and population-genetics studies have repeatedly linked neuroticism to shared genes [35, 36, 37]. Neuroticism was shown useful in psychometric screening and supports predicting real-world behavior [38, 39].

Despite strong population-level heritability [40, 41], the link between psychological constructs, brain function and genetics is still being actively researched [42, 33]. Empowered by emerging large-scale datasets, current attempts to predict fluid intelligence or neuroticism from thousands of MRI scans argue in favor of heterogeneity and weakly generalizing effects [43, 44]. This stands in contrast to the remarkable performance obtained when predicting psychometric data from language-based inputs captured by Twitter and Facebook user data [45, 46]. As MRI acquisitions can be difficult to come by in certain populations, the promises of social-media data are appealing. However, such data may lead to measurement and selection biases difficult to control. Instead, background sociodemographic data may provide an easily accessible alternative for contextualizing the heterogeneity of psychological traits [47].

Another challenge is that psychological traits are often measured using arbitrary non-physical units, *e.g.* education degree or monthly income. In fact, society treats individual differences as categorical or continuous, depending on the practical context. While personality has been proposed to span a continuum [48], psychiatrists treat certain people as patients and not others [49]. Therefore, a measure that performs globally poorly at a continuous scale can be sufficient to distinguish subgroups as it may be informative around the boundary region between certain classes, *e.g.*, pilots who should fly and who should not. Choosing the granularity with which to gauge psychological constructs is diffcult.

Confronting the promises of population phenotyping with the challenges of measuring psychological traits raises the following questions: 1) How well can various constructs related to mental health be approximated from general-purpose inputs not designed to measure specific latent constructs? 2) Can the success of brain age be extended to other proxy measures capturing complementary facets of mental health? 3) What is the relative merit of brain imaging and sociodemographics? We tackled these questions by using machine learning to craft *proxy measures* in order to approximate well-characterized *target measures* from brain-imaging and sociodemographic data. We studied age, fluid intelligence, and neuroticism. These targets have been, traditionally, considered as proxies for mental health and are fundamentally different in terms of scope and nature. Figure 1 summarizes our approach. Results suggest that, as with brain age, proxy measures can bring value for the study of mental health that goes beyond approximating an available measure.

Results: validity of proxy measures

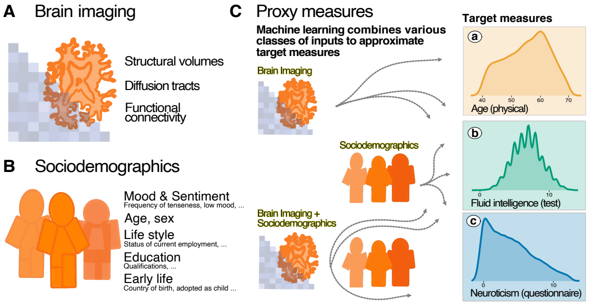

Figure 1. Methods overview: building and evaluating proxy measures We combined multiple brain-imaging modalities (**A**) with sociodemographic data (**B**) to approximate health-related biomedical and psychological constructs (**C**), *i.e.*, brain age (accessed through prediction of chronological age), cognitive capacity (accessed through a fluid-intelligence test) and the tendency to report negative emotions (accessed through a neuroticism questionnaire). We included the imaging data from the 10 000-subjects release of the UK biobank. Among imaging data (**A**) we considered features related to cortical and subcortical volumes, functional connectivity from rfMRI based on ICA networks, and white-matter molecular tracts from diffusive directions (see Table 1 for an overview about the multiple brain-imaging modalities). We then grouped the sociodemographic data (**B**) into five different blocks of variables related to self-reported mood & sentiment, primary demographics, lifestyle, education, and early-life events (Table 2 lists the number of variables in each block). Subsequently, we systematically compared the approximations of all three targets based on either brain images and sociodemographics in isolation or combined (**C**) to evaluate the relative contribution of these distinct inputs. Models were developed on 50% of the data (randomly drawn) based on random forest regression guided by Monte Carlo cross-validation with 100 splits (see section Model Development and Generalization Testing). We assessed generalization using the other 50% of the data as fully independent out-of-sample evaluations (see section Statistical Analysis). Learning curves suggested that this split-half approach provided sufficient data for model construction (Figure 1 – Figure supplement 1).

Complementing the original measures at characterizing real-life health-related habits

To approximate age, fluid intelligence and neuroticism, we applied random-forest regression on sociodemographic data and brain images. The data was split into *validation data* for model construction (see section Model Development and Generalization Testing) and *generalization data* for statistical inference on out-of-sample predictions with independent data (see section Statistical Analysis). Our findings suggested that some information on psychological constructs can be assembled from general inputs not specifically tailored to measure these constructs, such as brain images and sociodemographic variables. The resulting proxy measures may be regarded as crude approximations of the psychological measures, but they can nonetheless capture essential aspects of the target constructs. To probe the external validity of the proxy measures, we investigated their link with real-world behavior, e.g., sleep, physical exercise, alcohol and tobacco consumption on left-out data. To probe the external validity of the proxy-measures, we investigated their link with real-world behavior, *e.g.*, sleep, physical exercise, alcohol and tobacco consumption on left-out data. To relate such health behaviors to our proxy measures, we modeled them separately as weighted sums of predicted brain-age delta, fluid intelligence and neuroticism using multiple linear regression (section Statistical Analysis). To avoid circularity, we used the out-of-sample predictions for all proxy measures (section Model Development and Generalization Testing).

The estimated regression coefficients (partial correlations), revealed complementary associations between the proxy measures and health-related behavior (Figure 2). Similar patterns arise when considering proxy measures in isolation (Figure 2 – Figure supplement 1). Compared to other proxy measures, elevated brain-age delta was associated with increased alcohol consumption (Figure 2, first row). Levels of physical exercise were consistently associated with all three predicted targets, suggesting additive effects (Figure 2, second row). For fluid intelligence, this result, counter-intuitive from the health standpoint, could imply that higher test scores reveal a more sedentary life style. Increased sleep duration consistently went along with elevated brain age delta, but lower levels of predicted neuroticism (Figure 2, third row). This may seem counter-intuitive, but is conditional on neuroticism showing a negative link with sleep duration. No consistent effect emerged for fluid intelligence. Numbers of cigarettes smoked was independently associated with all predicted targets (Figure 2, last row): Intensified smoking went along with elevated brain age delta and neuroticism but lower fluid intelligence.

The three proxy measures are difficult to compare on an equal footing as a delta was considered for brain age only (the difference between predicted and actual age) and aging-specific deconfounding was applied. The brain-age delta is indeed the standard practice, theoretically justified as age is on a metric scale [50] for which the difference between the predicted and the measured value has a clear meaning. Such a difference is less obvious for variables with ordinal scales as implied by psychometric measures. Second, age has a pervasive influence on virtually any biomedical entity, which motivates controlling for its effect on proxy measures. To rule out that differences in proxy measures' associations to health-related behavior are driven by this methodological asymmetry, we repeated the main analysis from Figure 2, first, using the predicted age without computing the delta (Figure 2 – Figure supplement 2) and, second, introducing additional deconfounders for fluid intelligence and neuroticism (Figure 2 – Figure supplement 3). The resulting patterns were virtually unchanged, confirming that interpretations are robust.

A question that remains is whether the proxy measures bring additional value compared to the original target measures they were derived from. These original target measures showed similar associations to health behavior, with the same signs in most cases (Figure 2, B). At the same time, the ensuing patterns were more noisy, suggesting that empirically derived proxy measures yielded enhanced associations with health behavior. This inference may be difficult as differences between targets and proxies were not always easy to pinpoint visually. To implement a more rigorous statistical approach, we built comprehensive models of each respective health-related habit in which we used all proxies (predicted age, predicted fluid intelligence, predicted neuroticism) and all targets (age, fluid intelligence, neuroticism) simultaneously as predictors (Figure 2 – Figure supplement 4). The results show systematic additive effects of proxies and targets across the three target domains and the four health-habits. These trends are well-captured by the hypothesis tests of the respective linear models (Table S3). As targets and proxies may be systematically intercorrelated, multicollinearity may corrupt these inferences. Inspection of variance inflation factors (VIF)— a measure that reveals how well a given predictor can be approximated by a linear combination of the other predictors— argued in favor of low to moderate levels of multicollinearity (Table S4). Indeed, all VIF values fell between 3 and 1, whereas, classically, values above 5 or 10 are considered as thresholds [51] for pathological collinearity. This suggests that the model inferences are statistically sound.

The relative importance of brain and sociodemographic data depends on the target

In a second step, we investigated the relative performance of proxy measures built from brain signals and distinct sociodemographic factors for the three targets: age, fluid intelligence

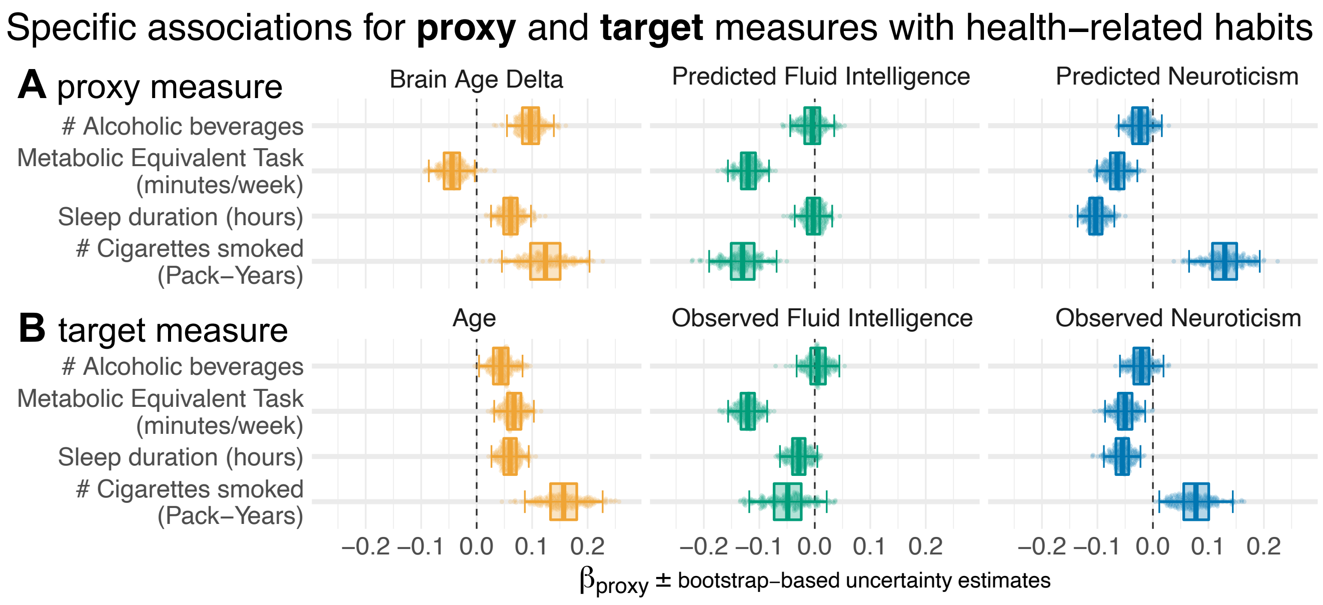

Figure 2. Proxy measures show systematic and complementary out-of-sample associations with health-related habits. We probed the external validity of all three proxy measures (brain age, fluid intelligence, neuroticism) based on a combination of brain images and all sociodemographic factors (see **Figure 1** for details). We investigated their out-of-sample associations with ecological indicators of mental health (sleep duration, time spent with physical exercise, number of alcoholic beverages and cigarettes consumed). To tease apart complementary and redundant effects, we constructed multiple linear regression models on out-of-sample predictions combining all three proxy measures **(A)**. For comparison, we repeated the analysis using the actual target measures **(B)** observed on the held-out data. Regression models are depicted rows-wise. Box plots summarize the uncertainty distribution of target-specific (color) regression coefficients with whiskers indicating two-sided 95% uncertainty intervals (parametric bootstrap). Dots illustrate a random subset of 200 out of 10 000 coefficient draws. The average coefficient estimate is annotated for convenience. At least two distinct patterns emerged: either the health outcome was specifically associated with one proxy measures (brain age delta and number of alcoholic beverages) or multiple measures showed additive associations with the outcome (*e.g.* number of pack years smoked). For target measures **(B)**, associations with health habits were often noisier or less pronounced compared to the target measures **(A)** and even a a change in direction was observed for brain age and metabolic activity. Figure 2 – Figure supplement 1 shows highly similar trends with marginal associations between proxy measures and health-related habits. Our results suggest that the proxy measures capture well health-related habits, potentially better than the original target measures, and in a complementary way across the three measures. The same patterns emerged as brain-predicted age rather than the brain age delta is used as a proxy measure (Figure 2 – Figure supplement 2). As proxy-specific deconfounding is applied, this pattern is preserved (Figure 2 – Figure supplement 3). Modeling of health-related habits jointly from proxy and target measures simultaneously revealed specific complementari. ty between proxy and target measures across multiple domains *i.e.* age, fluid intelligence, neuroticism (Figure 2 – Figure supplement 4).

and neuroticism. Among the sociodemographic variables there was one block for each target explaining most of the prediction performance (Figure 3, dotted outlines). Combining all sociodemographic variables did not lead to obvious enhancements (Figure 3 – Figure supplement 2). For age prediction, variables related to current life-style showed by far the highest performance. For fluid intelligence, education performed by far best. For neuroticism, mood & sentiment clearly showed the strongest performance.

Combining MRI and sociodemographics, enhanced age prediction systematically on all four blocks of variables (Figure 3 solid outlines, and Table S1). The benefit of brain-imaging features was less marked for prediction of fluid intelligence or neuroticism. With fluid intelligence, brain-imaging data improved the performance statistically significantly for all models, yet, with small effect sizes (Table S1). For neuroticism, no systematic benefit of including brain images alongside sociodemographics emerged (Table S1, bottom row). Nevertheless, brain data was sufficient for statistically significant approximation of the target measures in all three targets (Table S5).

Psychological measures often come without physical scales and units [50]. In practice, clinicians and educators use them with specific thresholds for decision making. To investigate empirically-defined proxy measures beyond continuous regression, we performed binary classification of extreme groups obtained from discretizing the targets using the 33_{rd} and 66_{th} percentiles, following the recommendations by Gelman and Hill 2006 regarding discrete variable encoding strategies. Furthermore, we measured accuracy with the area under the classification accuracy curve (AUC) which is only sensitive to ranking, ignoring the scale of the error. Classification performance visibly exceeded the chance level (AUC > 0.5) for all models (Figure 4) and approached or exceeded levels considered practically useful (AUC > 0.8) [49]. Across proxy measures, models including sociodemographics performed best but the difference between purely sociodemographic and brain-based models was comparably weak, at the order of 0.01-0.02 AUC points (Table S2). Using brain data only led to worse performance, yet, still better than chance as revealed by permutation testing (Table S6).

Discussion

Guided by machine learning, we empirically derived proxy measures that combine multiple sources of information to capture extensively validated target measures from psychology. These proxy measures all showed complementary associations with real-world health indicators beyond the original targets. The combination of brain imaging and target-specific sociodemographic inputs often improved approximation performance.

Empirically-derived proxy measures: validity and practical utility

In our study, construct validity [6, 54, 7] of the corresponding proxy measures was supported by the gain in prediction performance brought by specific sociodemographic factors (Fig-

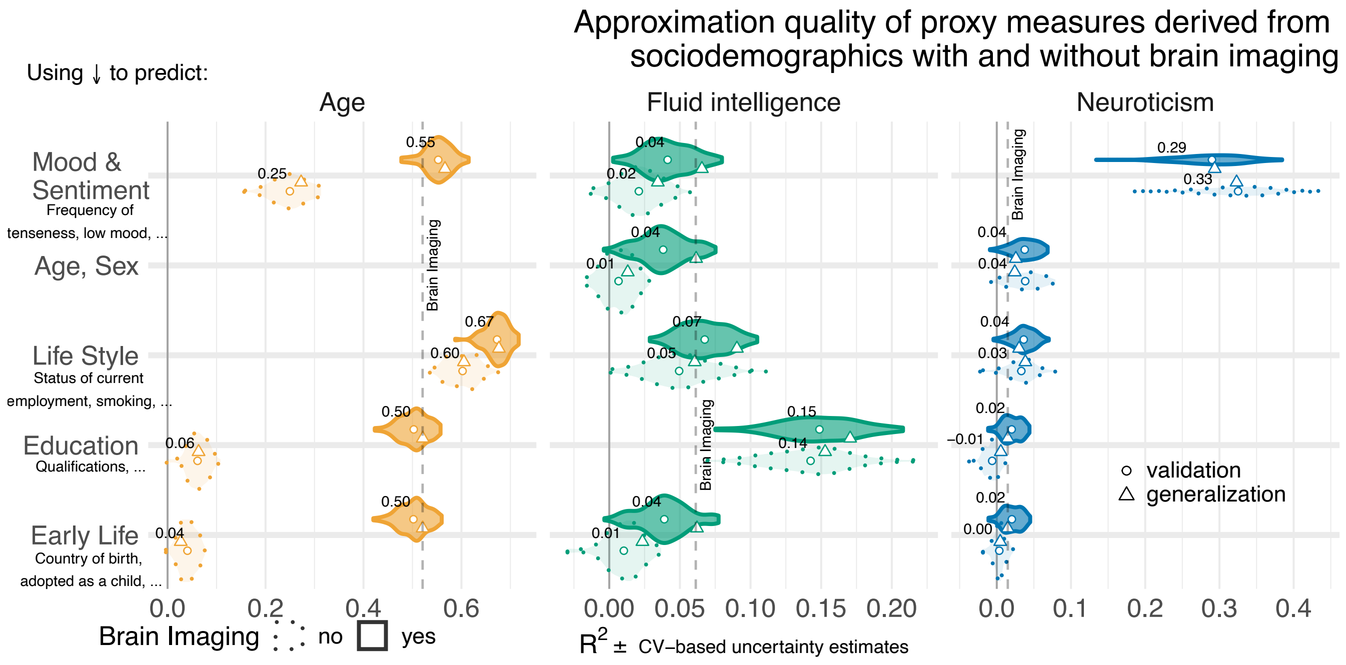

Figure 3. Approximation performance of proxy measures derived from sociodemographic data and MRI. We report the R^2 metric to facilitate comparisons across prediction targets. The cross-validation (CV) distribution (100 Monte Carlo splits) on the validation dataset is depicted by violins. Drawing style indicates whether brain imaging (solid outlines of violins) was included in addition or not (dotted outlines of violins). Dots depict the average performance on the validation data across CV-splits. Pyramids depict the performance of the average prediction (CV-bagging) on held-out generalization datasets. For convenience, the mean performance on the validation set is annotated for each plot. Vertical dotted lines indicate the average performance of the full MRI model. The validation and held-out datasets gave similar picture of approximation performance with no evidence for cross-validation bias [52]. For the averaged out-of-sample predictions, the probability of the observed performance under the null-distribution and the uncertainty of effect sizes were formally probed using permutation tests and bootstrap-based confidence intervals (Table S1). Corresponding statistics for the baseline performance of models solely based on brain imaging (vertical dotted lines) are presented in Table S5. Figure 3 – Figure supplement 1 shows approximation results based on MRI. Figure 3 – Figure supplement 2 presents results based on all sociodemographic factors.

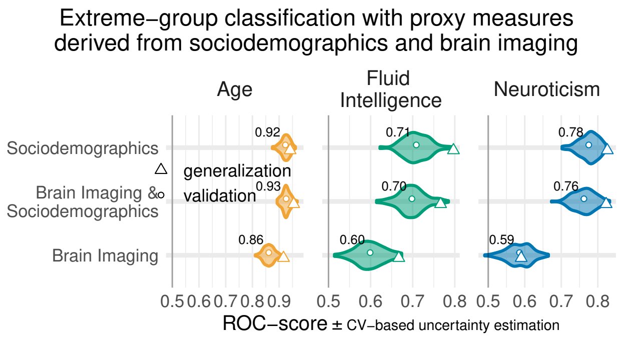

Figure 4. Classification analysis from imaging, sociodemographics and combination of both data. For classification of extreme groups instead of continuous regression, we split the data into low vs high groups based on 33_{rd} and 66_{th} percentiles. Visual conventions follow Figure 3. We report the accuracy in AUC. Models including sociodemographics performed visibly better than models purely based on brain imaging. Differences between brain-imaging and sociodemographics appeared less pronounced as compared to the fully-fledged regression analysis. For the average out-of-sample predictions, the probability of the observed performance under the null-distribution and the uncertainty of effect sizes were formally probed using permutation tests and bootstrap-based confidence intervals (Table S2). Corresponding statistics for the baseline performance of models solely based on brain imaging (vertical dotted lines) are presented in Table S6. Overall, when moving from the more difficult full-scale regression problem to extreme-group classification problem with purely ranking-based scores, the relative differences between brain-based and sociodemographics-based prediction gradually faded away.

ure 3). Association with health-relevant habits added external validity to the proxy measures (Figure 2). The complementary patterns related to traditional construct semantics: High consumption of cigarettes is associated with neuroticism [55], excessive drinking may lead to brain atrophy and cognitive decline [56] – both common correlates of elevated brain age [22, 57].

Can our empirically-derived proxy measures, thus, substitute for specific psychometric instruments? A mental-health professional may still prefer an established routine for clinical assessment, relying on interviews and personality questionnaires with implicit experience-based thresholds. Inclusion of brain imaging may even seem to yield diminishing returns when approximating high-level psychological traits. Yet, it could simply be a matter of time until more effective acquisition protocols will be discovered alongside useful signal representations. Including brain imaging, rather seems a "safe bet" as machine learning is often capable of selecting relevant inputs [11, 58] and costs of MRI-acquisition can be amortized by clinical usage. Empirically-derived proxy measures may open new doors where tailored assessment of latent constructs is not applicable due to lack of specialized mental-health workforce or sheer cost.

Constructs of mental-health can be accessed from general-purpose data

Brain age has served as landmark in this study. It has been arguably the most discussed candidate for a surrogate biomarker in the brain imaging literature [16, 17, 24]. With mean absolute errors around 4 years, up to 67% variance explained, and AUC-scores up to 0.93 in the classification setting, our results compare favorably to the recent brain-age literature within the UK Biobank [19, 59] and in other datasets [22, 11], though we relied on off-the-shelf methods and not custom deep learning methods [60]. Applying the same approach to psychologi-

cal constructs (fluid intelligence, neuroticism), we found that approximation from brain imaging data or sociodemographic descriptors was generally harder.

It is important to recapitulate that approximation quality on these differently measured targets has a different meaning. Age is measured with meaningful physical units (years) on a ratio scale [50] (Selma is *twice as old* as Bob). Psychometric scores are unit-free, which may provoke ambiguity regarding the level of measurement [54]. Their implied scales may be considered as interval (the *difference between* Bob's and Selma's intelligence is -0.1 standard deviations) if not ordinal (Bob's intelligence was *ranked below* Selma's) [50]. In day-to-day psychological practice, these scores are often used via practically-defined thresholds, *e.g.* school admission or pilot candidate selection [61, 62]. In the classification setting, all proxy measures approached or exceeded a performance of 0.80 deemed relevant in biomarker development [49], though to be fair, they approximated established psychometric targets (proxy measures themselves) and not a medical condition. Different proxy measures should, thus, be subjected to different standards, depending on the granularity of the implied measurement scale.

A more complete view on how the proxy measures capture mental-health constructs emerges from their associations with real-world behavior (Figure 2). Indeed, the associations with proxy measures (Figure 2 **B**) were less noisy and more consistent then with the target measures (Figure 2 **A**), regardless of their approximation quality. This may seem surprising at first, but the target measures are themselves noisy and of imperfect validity. These measures correspond to traditional tests which, in practice, must be interpreted by an expert, actively confronting their output with broader information on the individual. For instance, IQ scores are typically normalized across age groups. However, extending such a normalization approach to many factors (socio-economic status, culture, gender) poses fundamental high-dimensional statistics challenges. Conversely, using machine learning to assemble proxy measures by mapping the targets to rich sociodemographic and brain data implicitly contextualizes them. In this respect, the resulting measures capture more general signal than the original tests. Here, machine learning could be seen as mimicking the work of a mental health expert who carefully compares psychometric results with other facts known about an individual and its reference population.

The benefits offered by brain data depend on the target construct

All brain-derived approximations were statistically meaningful. Yet, only for age prediction, imaging data by itself led to convincing performance. For fluid intelligence and neuroticism, sociodemographic factors were the most important determinants of prediction success. The best-performing sociodemographic models were based on inputs semantically close to these targets, *i.e.*, education details or mood & sentiment. While those results support construct validity, they may come with a certain risk of circularity. The causal role of those predictors is not necessarily clear as better educational attainment is heritable itself [63] and may reinforce existing cognitive abilities. Similarly, prolonged emotional stress due to life events may exacerbate existing dispositions to experience negative emotions captured by neuroticism [64], traits which commonly help accumulate stressful life events [38]. Nevertheless, for fluid intelligence but not neuroticism, brain imaging added incremental value when combined with various sociodemographic predictors. This may suggest that the cues for neuroticism conveyed by brain imaging were already present in sociodemographic predictors, hinting at common causes. Off note, in the specific context of aging, the empirical distinction between brain age and cognitive age is reflecting a similar intuition [65].

Limitations

Additional constructs and psychometric tools could have been visited. The broader construct of intelligence is often estimated using a general factor model with multiple correlated tests. While this is obviously useful for normative assessments, measures of fluid intelligence can also serve a situational fitness signal [30]. There is a wealth of questionnaires for measuring negative emotionality and neuroticism, specifically. Yet, we could only study the EPQ scale provided by the UK Biobank. A complementary approach would be to estimate latent factors by pooling all non-imaging data semantically related to neuroticism [66]. Here, we considered established target measures "as is", instead of derivatives.

It terms of mental-health research, this study falls short of directly testing the clinical relevance of estimated proxy measures. Even in a very large general-population cohort such as the UK Biobank, there are only a few hundred diagnosed cases of mental disorders (ICD-10 mental-health diagnoses from the F chapter) with brain-imaging data available. As a result, we could not directly assess the performance of proxy measures in clinical populations. The low number of diagnosed mental disorders in UK Biobank highlights the practical importance of studying mental health as a continuous, in addition to diagnosed conditions. Indeed, a public health perspective calls for targeting individual differences in health, not only pathology. Psychological constructs such as IQ and neuroticsm are important factors of the epidemiology of psychiatric disorders [38, 30, 29, 67], and accelerated brain aging is associated with various neurological conditions [18, 17, 25]. Yet, few cohorts come with extensive neuropsychological testing. Validated proxies of these constructs open the door to including them in epidemiological studies as secondary outcomes or additional explanatory variables.

Conclusion: Proxy measures may enhance the validity of constructs gauging mental health

In population studies of mental health, individual traits are captured via lengthy assessments, tailored to specific brain and psychological constructs. We have shown that proxy measures built empirically from general-purpose data can capture these constructs and can improve upon traditional measures when studying real-world health patterns. Proxy measures can make psychological constructs available to broader, more ecological studies building on large epidemiological cohorts or real-world evidence. This can make the difference where psychological constructs are central to developing treatment and prevention strategies, but direct measures have not been collected.

Methods

To facilitate reproduction, understanding, and reuse, we have made all data analysis and visualization source code available on Github: `https://github.com/KamalakerDadi/empirical_proxy_measures`.

Dataset

The United Kingdom Biobank (UKBB) database is to date the most extensive large-scale cohort aimed at studying the deter-

minants of the health outcomes in the general adult population. The UKBB is openly accessible and has extensive data acquired on 500 000 individuals aged 40-70 years covering rich phenotypes, health-related information, brain-imaging and genetic data [12]. Participants were invited for repeated assessments, some of which included MR imaging. For instance, cognitive tests that were administered during an initial assessment were also assessed during the follow-up visits. This has enabled finding for many subjects at least one visit containing all heterogeneous input data needed to develop the proposed proxy measures. The study was conducted using the UKBB Resource Application 23827.

Participants

All participants gave informed consent. The UKBB study was examined and approved by the North West Multi-centre Research Ethics Committee. We considered participants who have responded to cognitive tests, questionnaires, and have access to their primary demographics and brain images [68]. Out of the total size of UKBB populations, we found 11 175 participants who had repeated assessments overlapping with the first brain imaging release [69]. Note that the features (sociodemographic variables) that we included in the analysis are measures that are self-reported during a follow-up imaging visit. The demographics are 51.6% female (5 572) and 48.3% male (5 403), and an age range between 40-70 years (with a mean of 55 years and standard deviation of 7.5 years). The data for model training were selected using a randomized split-half procedure yielding 5 587 individuals. The remaining subjects were set aside as a held-out set for generalization testing (see section Model development and generalization testing). We made sure that the subjects used for model training and generalization testing were strictly non-overlapping.

Learning curves documented that the training split was sufficiently large for constructing stable prediction models Figure 1 – Figure supplement 1 with profiles of performance similar to latest benchmarks on model complexity in the UK Biobank [70]. Moreover, simulations and empirical findings suggest that larger testing sets are more effective at mitigating optimistic performance estimates [71, 52]. Together, this provided a pragmatic solution to the inference-prediction dilemma [58, 72] given the two objectives of the present investigation to obtain reasonably good predictive models, while at the same time performing parameter inference of statistical models developed on the left-out data.

To establish specific comparisons between models based on sociodemographics, brain data or their combinations we exclusively considered the cases for which MRI scans were available. The final sample sizes used for model construction and generalization testing then depended on the availability of MRI: For age and fluid intelligence, our randomized split-half procedure (see section Model development and generalization testing) yielded 4203 cases for model building and 4157 for generalization. For cases with valid neuroticism assessment, fewer brain images were available, which yielded 3550 cases for model building and 3509 for generalization.

Data acquisition

Sociodemographic data (non-imaging) was collected with self-report measures administered through touchscreen questionnaires, complemented by verbal interviews, physical measures, biological sampling and imaging data. MRI data were acquired with the Siemens Skyra 3T using a standard Siemens 32-channel RF receiver head coil [73]. We considered three MR imaging modalities as each of them potentially captures unique neurobiological details: structural MRI (sMRI/T1), resting-state functional MRI (rs-fMRI) and diffusion MRI (dMRI). For technical details about the MR acquisition parameters, please refer to [69]. We used image-derived phenotypes (IDPs) of those distinct brain-imaging modalities, as they provide actionable summaries of the brain measurements and encourage comparability across studies.

Target measures

As our target measures for brain age modeling, we use an individual's age at baseline recruitment (UKBB code "21022-0.0"). Fluid intelligence, was assessed using a cognitive battery designed to measure an individual's capacity to solve novel problems that require logic and abstract reasoning. In the UK Biobank, the fluid intelligence test (UKBB code "20016-2.0") comprises thirteen logic and reasoning questions that were administered via the touchscreen to record a response within two minutes for each question. Therefore, each correct answer is scored as one point with 13 points in total[1]. Neuroticism (UKBB code "20127-0.0") was measured using a shorter version of the revised Eysenck Personality Questionnaire (EPQ-N) comprised of 12-items [32]. Neuroticism was assessed during Biobank's baseline visit. The summary of the individual's scores ranges from 0 to 12 that assess dispositional tendency to experience negative emotions [2].

In the course of this work, a question that emerged concerned the size of the gap between age at baseline recruitment and MRI-scan time and its potential impact on the analysis. Supplementary checks indicated that the age gap was at least 5 years for most participants. Yet, from a statistical perspective, the two age measures turned out highly interchangeable (Figure S2) and global conclusions remained unchanged (Figure S3).

Sociodemographic data

In this work, we refer to non-imaging variables broadly as sociodemographics excluding the candidate targets fluid intelligence and neuroticism. To approximate latent constructs from sociodemographics, we included 86 non-imaging inputs (Table S7) which are the collection of variables reflecting each participant's demographic and social factors *i.e.*, sex, age, date and month of birth, body mass index, ethnicity, exposures at early life –*e.g.* breast feeding, maternal smoking around birth, adopted as a child– education, lifestyle-related variables –*e.g.* occupation, household family income, household people living at the same place, smoking habits–, and mental-health variables. All these data were self-reported. We then assigned these 86 variables to five groups based on their relationships. Based on our conceptual understanding of the variables, we name assigned them to one out of five groups: **1)** mood & sentiment, **2)** primary demographics as age, sex, **3)** lifestyle, **4)** education, **5)** early life. We then investigated the intercorrelation between all 86 variables to ensure that the proposed grouping is compatible with their empirical correlation structure Figure S1.

The sociodemographic groups had varying amounts of missing data. For *e.g.* the source of missingness is concerned with the participants lifestyle habits such as smoking and mental health issues [74]. To deal with this missingness in the data

1 A complete overview of the 13 individual fluid intelligence items can be seen from this manual `https://biobank.ctsu.ox.ac.uk/crystal/crystal/docs/Fluidintelligence.pdf`

2 For a complete list of Neuroticism questionnaires can be seen from this manual `https://biobank.ctsu.ox.ac.uk/crystal/crystal/docs/MentalStatesDerivation.pdf`

using imputation [75], we used column-wise replacement of missing information with the median value calculated from the known part of the variable. We subsequently included an indicator for the presence of imputed for down-stream analysis. Such imputation is well suited to predictive models [76].

Image processing to derive phenotypes for machine learning

MRI data preprocessing were carried out by UKBB imaging team. The full technical details are described elsewhere [69, 73]. Below, we describe briefly the custom processing steps that we used on top of the already preprocessed inputs.

Structural MRI

This type of data analysis on T1-weighted brain images are concerned with morphometry of the gray matter areas *i.e.* the quantification of size, volume of brain structures and tissue types and their variations under neuropathologies or behavior [77]. For example, volume changes in gray matter areas over lifetime are associated with: brain aging [78], general intelligence [79] and brain disease [80]. Such volumes are calculated within pre-defined ROIs composed of cortical and sub-cortical structures [81] and cerebellar regions [82]. We included 157 sMRI features consisting of volume of total brain and grey matter along with brain subcortical structures[3]. All these features are pre-extracted by UKBB brain imaging team [69] and are part of data download. We concatenated all inputs alongside custom-built fMRI features for predictive analysis (feature union).

Diffusion weighted MRI

Diffusion MRI enables to identify white matter tracts along principal diffusive direction of water molecules, as well as the connections between different gray matter areas [83, 84]. The study of these local anatomical connections through white matter are relevant to the understanding of neuropathologies and functional organization [85]. We included 432 dMRI skeleton features of FA (fractional anisotropy), MO (tensor mode) and MD (mean diffusivity), ICVF (intra-cellular volume fraction), ISOVF (isotropic volume fraction) and OD (orientation dispersion index) modeled on many brain white matter structures extracted from neuroanatomy[4]. For extensive technical details, please refer to [86]. The skeleton features we included were from category134 shipped by the UKBB brain-imaging team and we used them without modification.

Functional MRI

Resting-state functional MR images capture low-frequency fluctuations in blood oxygenation that can reveal ongoing neuronal interactions in time forming distinct brain networks [87]. Functional connectivity within these brain network can be linked to clinical status [88], to behavior [69], or to psychological traits [44]. We also included resting-state connectivity features based on the time-series extracted from Independent Component Analysis (ICA) with 55 components representing various brain networks extracted on UKBB rfMRI data [69]. These included the default mode network, extended default mode network and cingulo-opercular network, executive control and attention network, visual network, and sensorimotor network. We measured functional connectivity in terms

3 Regional grey matter volumes `http://biobank.ctsu.ox.ac.uk/crystal/label.cgi?id=1101` Subcortical volumes `http://biobank.ctsu.ox.ac.uk/crystal/label.cgi?id=1102`

4 Diffusion-MRI skeleton measurements `http://biobank.ctsu.ox.ac.uk/crystal/label.cgi?id=134`

Table 1. Imaging-based models.

Index	Name	# variables	# groups
1	brain volumes (sMRI)	157	1
2	white matter (dMRI)	432	1
3	functional connectivity (fMRI)	1485	1
4	sMRI, dMRI	589	2
5	sMRI, fMRI	1642	2
6	dMRI, fMRI	1917	2
7	sMRI, dMRI, fMRI (full MRI)	2074	3

Table 2. Non-imaging baseline models or sociodemographic models based on single group. Variables in each group are described at corresponding section sociodemographic data

Index	Name	# variables
1	Mood & Sentiment (MS)	25
2	Age, Sex (AS)	5
3	Life style (LS)	45
4	Education (EDU)	2
5	Early Life (EL)	9

of the between-network covariance. We estimated the covariance matrices using Ledoit-Wolf shrinkage [89]. To account for the fact that covariance matrices live on a particular manifold, *i.e.*, a curved non-Euclidean space, we used the tangent-space embedding to transform the matrices into a Euclidean space [90, 91] following recent recommendations [92, 93]. For predictive modeling, we then vectorized the covariance matrices to 1485 features by taking the lower triangular part. These steps were performed with `NiLearn` [94].

Comparing predictive models to approximate target measures

Imaging-based models

First, we focused on purely imaging-based models based on exhaustive combinations of the three types of MRI modalities (see Table 1 for an overview). This allowed us to study potential overlap and complementarity between the MRI-modalities. Preliminary analyses revealed that combining all MRI data gave reasonable results with no evident disadvantage over particular combinations of MRI modalities (Figure 3 – Figure supplement 1), hence, for simplicity, we only focused on the full MRI model in subsequent analyses.

Sociodemographic models

We composed predictive models based on non-exhaustive combinations of different types of sociodemographic variables. To investigate the relative importance of each class of sociodemographic inputs, we performed systematic model comparisons. We were particularly interested in studying the relative contributions of early-life factors as compared to factors related to more recent life events such as education as well as factors related to current circumstances such as mood & sentiment and life-style. The resulting models based on distinct groups of predictors are listed in Table 2 (for additional details see Table S7 and Figure S1).

Combined imaging and sociodemographic models

In the next step, we were interested in how brain-related information would interact within each of these sociodemographic models. For example, information such as the age of an individual, or the level of education, may add important contextual information to brain images. We therefore considered an alter-

Table 3. Random forest hyperparameters and tuning with grid search (5 fold cross-validation).

Hyperparameter	Values
Impurity criterion	Mean squared error
Maximum tree depth	5, 10, 20, 40, full depth
Fraction of features for split	1, 5, “log2”, “sqrt”, “complete”
Number of trees	250

Table 4. Number of samples for classification analysis (N).

# groups	Age	Fluid intelligence	Neuroticism
1	1335	1108	1054
2	1200	898	1020

native variant for each of the models in Table 2 that included all MRI-related features (2 074 additional features) as described at section image processing to derive phenotypes for machine learning.

Predictive model

Linear models are recommended as default choice in neuroimaging research [92, 95] especially when datasets include fewer than 1000 data points. In this study approximated targets generated by distinct underlying mechanisms based on multiple classes of heterogenous input data with several thousands of data points. We hence chose the non-parametric random forest algorithm that can be readily applied on data of different units for non-linear regression and classification [96] with mean squared error as impurity criterion. To improve computation time we fixed tree-depth to 250 trees, a hyperparameter that is usually not tuned but set to a generous number as performance plateaus beyond a certain number of trees [97, ch. 15]. Preliminary analyses suggested that additional trees would not have led to substantial improvements in performance. We used nested cross-validation (5-fold grid search) to tune the depth of the trees as well as the number of variables considered for splitting (see Table 3 for a full list of hyper-parameters considered).

Classification analysis. We also performed classification analysis on the continuous targets. Adapting recommendations from Gelman and Hill [53], we performed discrete variable encoding of the targets leading to extreme groups based on the 33_{rd} and 66_{th} percentiles (see Table 4 for the number of classification samples per group). This choice avoids including samples near the average outcome for which the input data may be indistinct. We were particularly interested in understanding whether model performance would increase when moving toward classifying extreme groups. For this analysis, we considered all three types of models (full MRI 2074 features from imaging-based models, all sociodemographics variables, total 86 variables see section, combination of full MRI and all sociodemographics, a total 2160 variables see section (See section Comparing predictive models to approximate target measures). When predicting age, we excluded the age & sex sociodemographic block from all sociodemographic variables which then yielded a total of 81 variables. To assess the performance for classification analysis, we used the area under the curve (AUC) of the receiver operator characteristic (ROC) as an evaluation metric [95].

Model development and generalization testing

Before any empirical work, we generated two random partitions of the data, one validation dataset for model construction and one held-out generalization dataset for studying out-of-sample associations using classical statistical analyses.

For cross-validation, we then subdivided the validation set into 100 training- and testing splits following the Monte Carlo resampling scheme (also referred to as shuffle-split) with 10% of the data used for testing. To compare model performances based on paired tests, we used the same splits across all models. Split-wise testing performance was extracted and carried forward for informal inference using violin plots (Figure 3,Figure 4). For generalization testing, predictions on the held-out data were generated from all 100 models from each cross-validation split.

On the held-out set, unique subject-wise predictions were obtained by averaging across folds and occasional duplicate predictions due to Monte Carlo sampling which could produce multiple predictions per subject[5]. Such strategy is known as CV-bagging [98, 99] and can improve both performance and stability of results[6]. The resulting averages were reported as point estimates in Figures 3,4, and 3 – Figure supplement 1 and used as proxy measures in the analysis of health-related behaviors Figure 2.

Statistical analysis

Resampling statistics for model comparisons on the held-out data

To assess the statistical significance of the observed model performance and the differences in performance between the models, we computed resampling statistics of the performance metrics on the held-out generalization data not used for model construction [100]. Once unique subject-wise predictions were obtained on the held-out generalization data by averaging the predictions emanating from each fold of the validation set (cv-bagging), we computed null- and bootstrap-distributions of the observed test statistic on the held-out data, i.e., R^2 score for regression and *AUC* score for classification.

Baseline comparisons. To obtain a p-value for baseline comparisons (*could the prediction performance of a given model be explained by chance?*) on the held-out data, we permuted targets 10 000 times and then recomputed the test statistic in each iteration. P-values were then defined as the probability of the test statistic under null distribution being larger than the observed test statistic. To compute uncertainty intervals, we used bootstrap, recomputing the test statistic after resampling 10 000 times with replacement and reporting the 2.5 and 97.5 percentiles of the resulting distribution.

Pairwise comparisons between models. For model comparisons, we considered the out-of-sample difference in R^2 or *AUC* between any two models. To obtain a p-value for model comparisons (*could the difference in prediction performance between two given models be explained chance?*) on the held-out data, we permuted the scores predicted by model A and model B for every single prediction 10 000 times and then recomputed the test statistic in each iteration. We omitted all cases for which only predictions from one of the models under comparison was present. P-values were then defined as the probability of the absolute of the test statistic under null distribution being larger than the absolute observed test statistic. The absolute was considered to account for differences in both directions. Uncertainty intervals were obtained from computing the 2.5 and 97.5 percentiles of the bootstrap distribution based on 10 000 iterations. Here, predictions from model A and model B were

5 We ensured prior to computation that with 100 CV-splits, predictions were available for all subjects.

6 The use of CV-bagging can explain why on figures 3,4, and 3 – Figure supplement 1 the performance was sometimes slightly better on the held-out set compared to the cross-validation on the validation test.

Table 5. Extra health variables used for correlation analysis with subject-specific predicted scores.

Family	eid	Variables
Alcohol*	1568-0.0	Average weekly red wine intake
	1578-0.0	Average weekly champagne plus white wine intake
	1588-0.0	Average weekly beer plus cider intake
	1598-0.0	Average weekly spirits intake
	1608-0.0	Average weekly fortified wine intake
	5364-0.0	Average weekly intake of other alcoholic drinks
Physical activity	22040-0.0	Summed MET minutes per week for all activity
Smoking	20161-0.0	Pack years of smoking
Sleep	1160-0.0	Sleep duration

*We computed a compound drinking score by summing up all variables from the alcohol family

resampled using identical resampling indices to ensure a meaningful paired difference.

Out-of-sample association between proxy measures and health-related habits

Computation of brain age delta and de-confounding. For association with health-contributing habits (Table 5), we computed the brain age delta as the difference between predicted age and actual age:

$$BrainAge\Delta = Age_{predicted} - Age \tag{1}$$

As age prediction is rarely perfect, the residuals will still contain age-related variance which commonly leads to brain age bias when relating the brain age to an outcome of interest, *e.g.*, sleep duration [101]. To mitigate leakage of age-related information into the statistical models, we employed a de-confounding procedure in line with [102] and [11, eqs. 6-8] consisting in residualizing a measure of interest (*e.g.* sleep duration) with regard to age through multiple regression with quadratic terms for age. To minimize computation on the held-out data, we first trained a model relating the score of interest to age on the validation set to then derive a de-confounding predictor for the held-out generalization data. The resulting de-confounding procedure for variables in the held-out data amounts to computing an age-residualized predictor $measure_{resid}$ from the measure of interest (*e.g.* sleep duration) by applying the following quadratic fit on the validation data:

$$\begin{aligned} measure_{validation} = age_{validation} \times \beta_{val1} + \\ age^2_{validation} \times \beta_{val2} + \epsilon \end{aligned} \tag{2}$$

The de-confounding predictor was then obtained by evaluating the weights β_{val1} and β_{val2} obtained from Equation 2 on the generalization data:

$$\begin{aligned} deconfounder = age_{generalization} \times \beta_{val1} \\ + age^2_{generalization} \times \beta_{val2} \end{aligned} \tag{3}$$

We performed this procedure for all target measures, to study associations not driven by the effect of age. For supplementary analyses presented in figure Figure 2 – Figure supplement 3, the same procedure was applied, substituting age for fluid intelligence and neuroticism, respectively.

Health-related habits regression. We then investigated the joint association between proxy measures of interest and health-related habits (Table 5) using multiple linear regression. For simplicity, we combined all brain imaging and all sociodemographics variables (Figure 3, Figure 3 – Figure supplement 1, Figure 3 – Figure supplement 2). The ensuing model can be denoted as

$$\begin{aligned} measure = deconfounder \times \beta_1 + BrainAge\Delta \times \beta_2 \\ + PredFluidInt \times \beta_3 + PredNeurot \times \beta_4 + \epsilon, \end{aligned} \tag{4}$$

where *deconfounder* is given by Equation 2. Prior to model fitting, rows with missing inputs were omitted. For comparability, we then applied standard scaling on all outcomes and all predictors.

The parametric bootstrap was a natural choice for uncertainty estimation, as we used standard multiple linear regression which provides a well defined procedure for mathematically quantifying its implied probabilistic model. Computation was carried out using `sim` function from the `arm` package as described in [53, Ch.7,pp.142-143]. This procedure can be intuitively regarded as yielding draws from the posterior distribution of the multiple linear regression model under the assumption of a uniform prior. For consistency with previous analyses, we computed 10000 draws.

For supplementary analysis in Figure 2 – Figure supplement 2, the brain-predicted age instead of the delta was used:

$$\begin{aligned} measure = deconfounder \times \beta_1 + BrainAge \times \beta_2 + \\ PredFluidInt \times \beta_3 + PredNeurot \times \beta_4 + \epsilon, \end{aligned} \tag{5}$$

For supplementary analysis in Figure 2 – Figure supplement 3, additional deconfounders were introduced.

$$\begin{aligned} measure = deconfounder_{age} \times \beta_1 + \\ BrainAge \times \beta_2 + deconfounder_{FI} \times beta_3 + PredFluidInt \times \beta_4 + \\ deconfounder_N + \beta_5 + PredNeurot \times \beta_6 + \epsilon, \end{aligned} \tag{6}$$

where $deconfounder_{FI}$ is the deconfounder for fluid intelligence and $deconfounder_N$ the deconfounder for neuroticsm following the procedure described in Equation 2 and Equation 3.

For supplementary analysis in Figure 2 – Figure supplement 4, proxies and targets were analyzed simultaneously.

$$\begin{aligned} measure = Age \times \beta_1 + BrainAge \times \beta_2 + FluidIntelligence \times beta_3 + \\ PredFluidInt \times \beta_4 + \\ Neuroticism + \beta_5 + PredNeurot \times \beta_6 + \epsilon, \end{aligned} \tag{7}$$

Software

Preprocessing and model building were carried out using Python 3.7. The `NiLearn` library was used for processing MRI inputs [94]. We used the *scikit-learn* library for ma-

chine learning [103]. For statistical modeling and visualization we used the R-language [104] (version 3.5.3) and its ecosystem: `data.table` for high-performance manipulation of tabular data, `ggplot` [105, 106] for visualization and the `arm` package for parametric bootstrapping [107]. All data analysis code is shared on GitHub: https://github.com/KamalakerDadi/empirical_proxy_measures.

Availability of source code and requirements

- Project name: "empirical_proxy_measures"
- Project home page: e.g. https://github.com/KamalakerDadi/empirical_proxy_measures
- Operating system(s): e.g. Platform independent
- Programming language: e.g. Python and R
- Other requirements: e.g. Python 3.6.8 or higher, R 3.4.3 or higher
- License: BSD-3

Availability of supporting data and materials

The data supporting the results and figures of this article is available in the "empirical_proxy_measures" repository, https://github.com/KamalakerDadi/empirical_proxy_measures. The input data is publicly available via the UK Biobank http://www.ukbiobank.ac.uk.

Declarations

Author's Contributions (alphabetic order)

- **Conceptualization**: BT, DB, DE, GV, JH
- **Data curation**: DB, KD
- **Software**: BT, DE, GV, KD
- **Formal analysis**: DE, GV, KD
- **Supervision**: BT, DE, GV
- **Funding acquisition**: GV, JH
- **Validation**: DE, KD
- **Investigation**: DE, KD
- **Visualization**: DE, GV, KD
- **Methodology**: BT, DE, GV
- **Project administration**: DE, GV
- **Writing – original draft**: DE, KD
- **Writing – review and editing**: DB, BT, DE, GV, JH, KD

Acknowledgements

We would like to thank Dr. Stefania de Vito and Dr. Benjamin de Haas for the critical review and helpful discussion of previous versions of the manuscript. We would like to thank Dr. Julien Dubois and Prof. Ralph Adolphs for helpful discussions in the course of this research project.

References

1. Kapur S, Phillips AG, Insel TR. Why has it taken so long for biological psychiatry to develop clinical tests and what to do about it? Molecular Psychiatry 2012;17(12):1174–1179.
2. Insel T, Cuthbert B, Garvey M, Heinssen R, Pine DS, Quinn K, et al. Research Domain Criteria (RDoC): Toward a New Classification Framework for Research on Mental Disorders. American Journal of Psychiatry 2010;167(7):748–751.
3. Szucs D, Ioannidis JP. Empirical assessment of published effect sizes and power in the recent cognitive neuroscience and psychology literature. PLoS biology 2017;15(3):e2000797.
4. Hozer F, Houenou J. Can neuroimaging disentangle bipolar disorder? Journal of affective disorders 2016;195:199–214.
5. Enkavi AZ, Eisenberg IW, Bissett PG, Mazza GL, MacKinnon DP, Marsch LA, et al. Large-scale analysis of test–retest reliabilities of self-regulation measures. Proceedings of the National Academy of Sciences 2019;116(12):5472–5477.
6. Cronbach LJ, Meehl PE. Construct validity in psychological tests. Psychological Bulletin 1955;52(4):281–302.
7. Borsboom D, Mellenbergh GJ, van Heerden J. The Concept of Validity. Psychological Review 2004;111(4):1061–1071.
8. Eisenberg IW, Bissett PG, Enkavi AZ, Li J, MacKinnon DP, Marsch LA, et al. Uncovering the structure of self-regulation through data-driven ontology discovery. Nature Communications 2019;10(1):1–13.
9. Nave G, Jung WH, Linnér RK, Kable JW, Koellinger PD. Are Bigger Brains Smarter? Evidence From a Large-Scale Preregistered Study:. Psychological Science 2018;.
10. Kievit RA, Fuhrmann D, Borgeest GS, Simpson-Kent IL, Henson RNA. The neural determinants of age-related changes in fluid intelligence: a pre-registered, longitudinal analysis in UK Biobank. Wellcome Open Research 2018;3.
11. Engemann DA, Kozynets O, Sabbagh D, Lemaître G, Varoquaux G, Liem F, et al. Combining magnetoencephalography with magnetic resonance imaging enhances learning of surrogate-biomarkers. eLife 2020 may;9:e54055. https://doi.org/10.7554/eLife.54055.
12. Collins R. What makes UK Biobank special? The Lancet 2012;379(9822):1173–1174.
13. Woo CW, Chang LJ, Lindquist MA, Wager TD. Building better biomarkers: brain models in translational neuroimaging. Nature Neuroscience 2017;20(3):365–377.
14. Varoquaux G. Cross-validation failure: Small sample sizes lead to large error bars. NeuroImage 2018;180:68–77.
15. Bzdok D, Meyer-Lindenberg A. Machine learning for precision psychiatry: opportunities and challenges. Biological Psychiatry: Cognitive Neuroscience and Neuroimaging 2018;3(3):223–230.
16. Dosenbach NU, Nardos B, Cohen AL, Fair DA, Power JD, Church JA, et al. Prediction of individual brain maturity using fMRI. Science 2010;329(5997):1358–1361.
17. Cole JH, Leech R, Sharp DJ, Initiative ADN. Prediction of brain age suggests accelerated atrophy after traumatic brain injury. Annals of neurology 2015;77(4):571–581.
18. Cole JH, Ritchie SJ, Bastin ME, Hernández MV, Maniega SM, Royle N, et al. Brain age predicts mortality. Molecular psychiatry 2018;23(5):1385.
19. Smith SM, Elliott LT, Alfaro-Almagro F, McCarthy P, Nichols TE, Douaud G, et al. Brain aging comprises many modes of structural and functional change with distinct genetic and biophysical associations. Elife 2020;9:e52677.
20. Koutsouleris N, Davatzikos C, Borgwardt S, Gaser C, Bottlender R, Frodl T, et al. Accelerated brain aging in schizophrenia and beyond: a neuroanatomical marker of psychiatric disorders. Schizophrenia bulletin 2014;40(5):1140–1153.
21. He T, An L, Feng J, Bzdok D, Holmes AJ, Eickhoff SB, et al. Meta-matching: a simple framework to translate phenotypic predictive models from big to small data. bioRxiv 2020;.
22. Liem F, Varoquaux G, Kynast J, Beyer F, Masouleh SK,

Huntenburg JM, et al. Predicting brain-age from multimodal imaging data captures cognitive impairment. NeuroImage 2017;148:179–188.
23. Spiegelhalter D. How old are you, really? Communicating chronic risk through 'effective age'of your body and organs. BMC medical informatics and decision making 2016;16(1):1–6.
24. Smith SM, Vidaurre D, Alfaro-Almagro F, Nichols TE, Miller KL. Estimation of brain age delta from brain imaging. NeuroImage 2019;.
25. Gonneaud J, Baria AT, Binette AP, Gordon BA, Chhatwal JP, Cruchaga C, et al. Functional brain age prediction suggests accelerated aging in preclinical familial Alzheimer's disease, irrespective of fibrillar amyloid-beta pathology. bioRxiv 2020;.
26. Cattell RB, Scheier IH. The meaning and measurement of neuroticism and anxiety. 1961;.
27. Cattell RB. Theory of fluid and crystallized intelligence: A critical experiment. Journal of educational psychology 1963;54(1):1.
28. Shelton JT, Elliott EM, Matthews RA, Hill B, Gouvier W, others. The relationships of working memory, secondary memory, and general fluid intelligence: working memory is special. Journal of Experimental Psychology: Learning, Memory, and Cognition 2010;36(3):813.
29. Keyes KM, Platt J, Kaufman AS, McLaughlin KA. Association of Fluid Intelligence and Psychiatric Disorders in a Population-Representative Sample of US Adolescents. JAMA psychiatry 2017;74(2):179–188.
30. Khandaker GM, Dalman C, Kappelmann N, Stochl J, Dal H, Kosidou K, et al. Association of Childhood Infection With IQ and Adult Nonaffective Psychosis in Swedish Men: A Population-Based Longitudinal Cohort and Co-relative Study. JAMA Psychiatry 2018;75(4):356–362.
31. Costa PT, McCrae RR. Neo Pi-R. Psychological Assessment Resources Odessa, FL; 1992.
32. Eysenck SB, Eysenck HJ, Barrett P. A Revised Version of the Psychoticism Scale. Personality and Individual Differences 1985;6:21–29.
33. Shackman AJ, Tromp DP, Stockbridge MD, Kaplan CM, Tillman RM, Fox AS. Dispositional negativity: An integrative psychological and neurobiological perspective. Psychological bulletin 2016;142(12):1275.
34. Lynn R, Martin T. Gender differences in extraversion, neuroticism, and psychoticism in 37 nations. The Journal of social psychology 1997;137(3):369–373.
35. Hettema JM, Neale MC, Myers JM, Prescott CA, Kendler KS. A population-based twin study of the relationship between neuroticism and internalizing disorders. American journal of Psychiatry 2006;163(5):857–864.
36. Birley AJ, Gillespie NA, Heath AC, Sullivan PF, Boomsma DI, Martin NG. Heritability and nineteen-year stability of long and short EPQ-R Neuroticism scales. Personality and individual differences 2006;40(4):737–747.
37. Pedersen NL, Plomin R, McClearn GE, Friberg L. Neuroticism, extraversion, and related traits in adult twins reared apart and reared together. Journal of personality and social psychology 1988;55(6):950.
38. Lahey BB. Public health significance of neuroticism. American Psychologist 2009;64(4):241.
39. Tyrer P, Reed GM, Crawford MJ. Classification, assessment, prevalence, and effect of personality disorder. The Lancet 2015;385(9969):717–726.
40. Power RA, Pluess M. Heritability estimates of the Big Five personality traits based on common genetic variants. Translational psychiatry 2015;5(7):e604.
41. Vukasović T, Bratko D. Heritability of personality: a meta-analysis of behavior genetic studies. Psychological bulletin 2015;141(4):769.
42. Yarkoni T. Neurobiological substrates of personality: A critical overview. APA handbook of personality and social psychology 2015;4:61–83.
43. Dubois J, Galdi P, Han Y, Paul LK, Adolphs R. Resting-State Functional Brain Connectivity Best Predicts the Personality Dimension of Openness to Experience. Personality Neuroscience 2018;1.
44. Dubois J, Galdi P, Paul LK, Adolphs R. A distributed brain network predicts general intelligence from resting-state human neuroimaging data. Philosophical Transactions of the Royal Society B: Biological Sciences 2018;373(1756):20170284.
45. Quercia D, Kosinski M, Stillwell D, Crowcroft J. Our twitter profiles, our selves: Predicting personality with twitter; 2011. p. 180–185.
46. Youyou W, Kosinski M, Stillwell D. Computer-based personality judgments are more accurate than those made by humans. Proceedings of the National Academy of Sciences 2015;112(4):1036–1040.
47. Horn JL, Donaldson G, Engstrom R. Apprehension, Memory, and Fluid Intelligence Decline in Adulthood. Research on Aging 1981;3(1):33–84. https://doi.org/10.1177/016402758131002.
48. Eysenck HJ. The continuity of abnormal and normal behavior. Psychological Bulletin 1958;55(6):429–432.
49. Perlis RH. Translating biomarkers to clinical practice. Molecular Psychiatry 2011;16(11):1076–1087.
50. Stevens SS, et al. On the theory of scales of measurement 1946;.
51. O'brien RM. A caution regarding rules of thumb for variance inflation factors. Quality & quantity 2007;41(5):673–690.
52. Varoquaux G, Raamana PR, Engemann DA, Hoyos-Idrobo A, Schwartz Y, Thirion B. Assessing and tuning brain decoders: Cross-validation, caveats, and guidelines. NeuroImage 2017;145:166 – 179. http://www.sciencedirect.com/science/article/pii/S105381191630595X, individual Subject Prediction.
53. Gelman A, Hill J. Data analysis using regression and multilevel/hierarchical models. Cambridge university press; 2006.
54. Borsboom D. Measuring the mind: Conceptual issues in contemporary psychometrics. Cambridge University Press; 2005.
55. Terracciano A, Costa Jr PT. Smoking and the Five-Factor Model of personality. Addiction 2004;99(4):472–481.
56. Topiwala A, Allan CL, Valkanova V, Zsoldos E, Filippini N, Sexton C, et al. Moderate alcohol consumption as risk factor for adverse brain outcomes and cognitive decline: longitudinal cohort study. bmj 2017;357:j2353.
57. Wang J, Knol MJ, Tiulpin A, Dubost F, de Bruijne M, Vernooij MW, et al. Gray matter age prediction as a biomarker for risk of dementia. Proceedings of the National Academy of Sciences 2019;116(42):21213–21218.
58. Bzdok D, Engemann D, Thirion B. Inference and Prediction Diverge in Biomedicine. Cell Press Patterns 2020 2020/10/13;https://doi.org/10.1016/j.patter.2020.100119.
59. Cole JH, Poudel RP, Tsagkrasoulis D, Caan MW, Steves C, Spector TD, et al. Predicting brain age with deep learning from raw imaging data results in a reliable and heritable biomarker. NeuroImage 2017;163:115–124.
60. He T, Kong R, Holmes AJ, Sabuncu MR, Eickhoff SB, Bzdok D, et al. Is deep learning better than kernel regression for functional connectivity prediction of fluid intelligence? In: 2018 International Workshop on Pattern Recognition in Neuroimaging (PRNI) IEEE; 2018. p. 1–4.

61. Carretta TR. Pilot candidate selection method. Aviation Psychology and Applied Human Factors 2011;.
62. Carretta TR, Ree MJ. Pilot-candidate selection method: Sources of validity. The International Journal of Aviation Psychology 1994;4(2):103–117.
63. Krapohl E, Rimfeld K, Shakeshaft NG, Trzaskowski M, McMillan A, Pingault JB, et al. The high heritability of educational achievement reflects many genetically influenced traits, not just intelligence. Proceedings of the national academy of sciences 2014;111(42):15273–15278.
64. Colodro-Conde L, Couvy-Duchesne B, Zhu G, Coventry WL, Byrne EM, Gordon S, et al. A direct test of the diathesis–stress model for depression. Molecular psychiatry 2018;23(7):1590–1596.
65. Anatürk M, Kaufmann T, Cole JH, Suri S, Griffanti L, Zsoldos E, et al. Prediction of brain age and cognitive age: Quantifying brain and cognitive maintenance in aging. Human brain mapping 2021;42(6):1626–1640.
66. Maglanoc LA, Kaufmann T, Meer Dvd, Marquand AF, Wolfers T, Jonassen R, et al. Brain Connectome Mapping of Complex Human Traits and Their Polygenic Architecture Using Machine Learning. Biological Psychiatry 2020;87(8):717–726.
67. Khandaker GM, Barnett JH, White IR, Jones PB. A quantitative meta-analysis of population-based studies of premorbid intelligence and schizophrenia. Schizophrenia research 2011;132(2-3):220–227.
68. Sudlow C, Gallacher J, Allen N, Beral V, Burton P, Danesh J, et al. UK Biobank: An Open Access Resource for Identifying the Causes of a Wide Range of Complex Diseases of Middle and Old Age. PLOS Medicine 2015;12(3):1–10.
69. Miller KL, Alfaro-Almagro F, Bangerter NK, Thomas DL, Yacoub E, Xu J, et al. Multimodal population brain imaging in the UK Biobank prospective epidemiological study. Nature neuroscience 2016;19(11):1523–1536.
70. Schulz MA, Yeo BT, Vogelstein JT, Mourao-Miranada J, Kather JN, Kording K, et al. Different scaling of linear models and deep learning in UKBiobank brain images versus machine-learning datasets. Nature communications 2020;11(1):1–15.
71. Flint C, Cearns M, Opel N, Redlich R, Mehler DM, Emden D, et al. Systematic misestimation of machine learning performance in neuroimaging studies of depression. Neuropsychopharmacology 2021;46(8):1510–1517.
72. IJ H. Statistics versus machine learning. Nature methods 2018;15(4):233.
73. Alfaro-Almagro F, Jenkinson M, Bangerter NK, Andersson JLR, Griffanti L, Douaud G, et al. Image processing and Quality Control for the first 10,000 brain imaging datasets from UK Biobank. NeuroImage 2018;166:400–424.
74. Fry A, Littlejohns TJ, Sudlow C, Doherty N, Adamska L, Sprosen T, et al. Comparison of Sociodemographic and Health-Related Characteristics of UK Biobank Participants With Those of the General Population. American Journal of Epidemiology 2017 06;186(9):1026–1034. https://doi.org/10.1093/aje/kwx246.
75. Little RJA, Rubin DB. Statistical Analysis with Missing Data. New York, NY, USA: John Wiley & Sons, Inc.; 1986.
76. Josse J, Prost N, Scornet E, Varoquaux G. On the consistency of supervised learning with missing values; 2019, https://hal.archives-ouvertes.fr/hal-02024202, working paper or preprint.
77. Lerch JP, van der Kouwe AJ, Raznahan A, Paus T, Johansen-Berg H, Miller KL, et al. Studying neuroanatomy using MRI. Nature neuroscience 2017;20:314 – 326.
78. Ritchie SJ, Dickie DA, Cox SR, Valdes Hernandez MdC, Corley J, Royle NA, et al. Brain volumetric changes and cognitive ageing during the eighth decade of life. Human Brain Mapping 2015;36(12):4910–4925.
79. Cox S, Ritchie S, Fawns-Ritchie C, Tucker-Drob E, Deary I. Structural brain imaging correlates of general intelligence in UK Biobank. Intelligence 2019;76:101376.
80. Thompson PM, Hayashi KM, Dutton RA, Chiang MC, Leow AD, Sowell ER, et al. Tracking Alzheimer's Disease. Annals of the New York Academy of Sciences 2007;1097:183–214.
81. Desikan RS, Ségonne F, Fischl B, Quinn BT, Dickerson BC, Blacker D, et al. An automated labeling system for subdividing the human cerebral cortex on MRI scans into gyral based regions of interest. Neuroimage 2006;31(3):968–980.
82. Diedrichsen J, Balsters JH, Flavell J, Cussans E, Ramnani N. A probabilistic MR atlas of the human cerebellum. NeuroImage 2009;46(1):39 – 46.
83. Behrens TEJ, Woolrich MW, Jenkinson M, Johansen-Berg H, Nunes RG, Clare S, et al. Characterization and propagation of uncertainty in diffusion-weighted MR imaging. Magnetic Resonance in Medicine 2003;50:1077–1088.
84. Conturo TE, Lori NF, Cull TS, Akbudak E, Snyder AZ, Shimony JS, et al. Tracking neuronal fiber pathways in the living human brain. Proceedings of the National Academy of Sciences 1999;96:10422–10427.
85. Saygin ZM, Osher DE, Norton ES, Youssoufian DA, Beach SD, Feather J, et al. Connectivity precedes function in the development of the visual word form area. Nature neuroscience 2016;19.
86. de Groot M, Vernooij MW, Klein S, Ikram MA, Vos FM, Smith SM, et al. Improving alignment in Tract-based spatial statistics: Evaluation and optimization of image registration. NeuroImage 2013;76:400–411.
87. Biswal B, Yetkin FZ, Haughton VM, Hyde JS. Functional connectivity in the motor cortex of resting human brain using echo-planar mri. Magnetic Resonance in Medicine 1995;34(4):537–541.
88. Greicius MD, Srivastava G, Reiss AL, Menon V. Default-mode network activity distinguishes Alzheimer's disease from healthy aging: evidence from functional MRI. Proceedings of the National Academy of Sciences 2004;101:4637.
89. Ledoit O, Wolf M. Honey, I shrunk the sample covariance matrix. The Journal of Portfolio Management 2004;30(4):110–119.
90. Varoquaux G, Baronnet F, Kleinschmidt A, Fillard P, Thirion B. Detection of brain functional-connectivity difference in post-stroke patients using group-level covariance modeling. Medical image computing and computer-assisted intervention: MICCAI International Conference on Medical Image Computing and Computer-Assisted Intervention 2010;13(Pt 1):200–208.
91. Sabbagh D, Ablin P, Varoquaux G, Gramfort A, Engeman DA. Manifold-regression to predict from MEG/EEG brain signals without source modeling. In: Advances in Neural Information Processing Systems (NeurIPS); 2019. .
92. Dadi K, Rahim M, Abraham A, Chyzhyk D, Milham M, Thirion B, et al. Benchmarking functional connectome-based predictive models for resting-state fMRI. NeuroImage 2019;192:115–134.
93. Pervaiz U, Vidaurre D, Woolrich MW, Smith SM. Optimising network modelling methods for fMRI. NeuroImage 2020;211:116604.
94. Abraham A, Pedregosa F, Eickenberg M, Gervais P, Mueller A, Kossaifi J, et al. Machine learning for neuroimaging with scikit-learn. Frontiers in Neuroinformatics 2014;8.
95. Poldrack RA, Huckins G, Varoquaux G. Establishment of

best practices for evidence for prediction: a review. JAMA psychiatry 2020;77(5):534–540.
96. Breiman L. Random Forests. Machine Learning 2001;45(1):5–32.
97. Hastie T, Tibshirani R, Friedman J, Franklin J. The elements of statistical learning: data mining, inference and prediction. The Mathematical Intelligencer 2005;27(2):83–85.
98. Varoquaux G, Raamana PR, Engemann DA, Hoyos-Idrobo A, Schwartz Y, Thirion B. Assessing and tuning brain decoders: Cross-validation, caveats, and guidelines. NeuroImage 2017;145(August 2015):166–179.
99. O'Connor D, Lake EM, Scheinost D, Constable RT. Resample aggregating improves the generalizability of connectome predictive modeling. NeuroImage 2021;236:118044.
100. Gemein LAW, Schirrmeister RT, Chrabąszcz P, Wilson D, Boedecker J, Schulze-Bonhage A, et al. Machine-learning-based diagnostics of EEG pathology. NeuroImage 2020;220:117021. http://www.sciencedirect.com/science/article/pii/S1053811920305073.
101. Le TT, Kuplicki RT, McKinney BA, Yeh HW, Thompson WK, Paulus MP, et al. A Nonlinear Simulation Framework Supports Adjusting for Age When Analyzing BrainAGE. Frontiers in Aging Neuroscience 2018;10:317. https://www.frontiersin.org/article/10.3389/fnagi.2018.00317.
102. Smith SM, Vidaurre D, Alfaro-Almagro F, Nichols TE, Miller KL. Estimation of brain age delta from brain imaging. NeuroImage 2019;200:528 – 539.
103. Pedregosa F, Varoquaux G, Gramfort A, Michel V, Thirion B, Grisel O, et al. Scikit-learn: Machine Learning in Python. J Mach Learn Res 2011 nov;12:2825–2830.
104. R Core Team. R: A Language and Environment for Statistical Computing. R Foundation for Statistical Computing, Vienna, Austria; 2019, https://www.R-project.org/.
105. Wickham H. ggplot2: Elegant Graphics for Data Analysis. Springer-Verlag New York; 2016. http://ggplot2.org.
106. Clarke E, Sherrill-Mix S. ggbeeswarm: Categorical Scatter (Violin Point) Plots; 2017, https://CRAN.R-project.org/package=ggbeeswarm, r package version 0.6.0.
107. Gelman A, Su YS. arm: Data Analysis Using Regression and Multilevel/Hierarchical Models; 2020, https://CRAN.R-project.org/package=arm, r package version 1.11-1.

Click here to access/download;Manuscript;reply-to-the-reviewers.pdf

Summary

We would like to thank the editor and the reviewers for the thoughtful feedback and constructive criticism on our work. We have taken the revisions as an important opportunity to improve our manuscript and would like to highlight two points that both reviewers and the editor have emphasized:

1. both reviewers had concerns regarding the far-reaching conclusion: the **“findings suggested that psychological constructs can be approximated from brain images and sociodemographic variables."** Considering these concerns, we added methodological and conceptual clarifications, discussed our findings more carefully and revised the general conclusions to express greater nuance.

2. both reviewers also pointed out that the phenotype **“age / brain age"** is different from the other measurements. We conducted multiple control analysis to investigate this point and clarify the differences, both, conceptually and statistically. The wealth of results and related discussion points are fully reported in the revised manuscript.

This required substantial reanalysis demanding several weeks of computation time and led us to including 6 new supplementary figures and 2 new supplementary tables. A detailed display of the changes in the manuscript is available in the file `diff-with-original-submission.pdf` enclosed with the resubmission.

Please note that, where appropriate, replies are organized by bundles of related concerns, sometimes involving concerns raised by both reviewers.

Reviewer 1

R1.1

The manuscript describes an application of Machine Learning (ML) models for the quantification of psychological constructs, e.g., fluid intelligence and neuroticism, using multi-model MRI data from a large population cohort, the UK biobank data. They show that the proxy measures of these psychological constructs are more useful compared to the original constructs for characterizing health behaviors. Overall, the manuscript is well written. The research questions are clearly stated and are of practical importance. However, the reviewer has following concerns.

Reply to R1.1: We thank the reviewer for the positive appreciation of our work.

R1.2

Major Concerns:
1) In page 3 (left, lines 3-6 of the main text), the author claims that "Our findings suggested that psychological constructs can be approximated from brain images and sociodemographic variables - inputs not tailored to specifically measure these constructs.". The reviewer has concerns about this claim. Although Figure 3 shows the model's performance in predicting age, fluid intelligence and neuroticism using

neuroimaging data and different areas of sociodemographic data, the performance of the models in predicting the psychological constructs, fluid intelligences and neuroticism, may not be good enough to support such a claim.

Reply to R1.2: We thank the reviewer for pointing out the potential ambiguity of the wording. An approximation can be understood as an “almost perfect prediction” but also as a “crude guess”. In fact, we did not intend to suggest that the approximations are perfect. We have now rephrased the above sentence accordingly and, moreover, carefully edited other parts of the main text to help avoid this misunderstanding.

Changes

In abstract (instead of advantage):

> We observed this complementarity of proxy measures and original measures when modeling from brain signals or sociodemographic data, capturing multiple health-related constructs.

In results section title (instead of outperforming):

> Complementing the original measures at characterizing real-life health-related habits

In results section:

> Our findings suggested that some information on psychological constructs can be assembled from general inputs not specifically tailored to measure these constructs, such as brain images and sociodemographic variables. The resulting proxy measures may be regarded as crude approximations of the psychological measures, but they can nonetheless capture essential aspects of the target constructs. To probe the external validity of the proxy-measures, we investigated their link with real-world behavior, e.g., sleep, physical exercise, alcohol and tobacco consumption on left-out data.

R1.3

2) In Figure 2, the proxy measure and original measure show similar associations with the health phenotypes for fluid intelligence (center plot) and neuroticism (right plot), but not for the brain age delta. The main reason seems to be when doing the association analysis, the measures of the health phenotypes are de-confounded for their dependence for age (In the subsection "Out-of-sample association between proxy measures and health-related habits" of the "statistical analysis" section). However, it seems the same procedure is not applied for the association analysis of fluid intelligence and neuroticism. The estimated brain age or brain age gap depends on the age. Thus, we need to either correct the brain age or brain age gap for its dependence on the age, or de-confounded the health phenotype's dependence on age. If the author wants to derive the proxy measure of the psychological construct in the same as the brain age (or biological age), same procedure should be used to correct the proxy measure's dependence on the original measure.

AND

R1.4

3) Based on Figure 2, the author claims that the proxy measures have enhanced association with health behavior compared to the original measures. If we only focus on the central and right part of the Figure 2, the difference is not that obvious. We do not know if the difference is significant or not. A better approach maybe is that correct the predicted fluid intelligence and predicted fluid intelligence for their dependence on the original measures or de-confounded the original measures' effects on the health behaviors.

Reply to R1.3-R1.4: We thank the reviewer for giving us the opportunity for clarifying our conceptual view and substantially extending the analysis in response to these thoughtful concerns. Note that concerns **R2.2-2.4** by reviewer 2 were highly related. Therefore, the following reply is intended to address the same issue raised by both reviewers**.**

First of all, we believe that the three measures under study are fundamentally distinct and lead to different statistical behaviors, which is the reason for which we included them in this study. Age is a metric measure, related to physical units on which intervals of the same size express the same distance. A delta can thus be a meaningful summary, which, in the case of brain age, has a straight-forward interpretation. For the other measures, ordinal or categorical scales apply; the delta is less convincing as a proxy measure metric here and may have no meaning.

Rather, in our view, what is important is that the predicted measure contains information -- in the widest sense -- beyond the actual measure. Therefore, the popular brain age delta is not necessary here. A multivariate analysis linking both chronological age and predicted age to the outcome of interest can capture the same information (see new figure-2 supplements 2-4). On the other hand, when inspecting the other proxy measures, we found it important to use an age deconfounder, to confirm that effects were not driven by age, as age shows pervasive correlations with nearly any biobehavioral entity. This de-confounding is important to interpret results as indeed driven by the construct of interest, but associations hold without it or when adding specific deconfounders to all proxies, as we show in new analyses below (see new figure-2 supplements 3-4).

These considerations explain why we have initially processed the proxies in different ways (age-decorrelation for all proxies, delta for brain age only). We understand, however, that rational arguments alone may feel unsatisfying from a reader's perspective. We have taken the opportunity to extend our analysis. To probe the robustness of our analysis to these different options we have run several checks which we have included in the supplements (described below).

While implementing these extra checks, we realized that in figure 2B, we had kept the age-deconfounder active when modeling the link between age and the health descriptors, which was not ideal as it may destroy the effect. We have corrected this accordingly and removed the age-deconfounder subsequently from the target measure analyses (see corrected figure 2 below).

New figure 2 after removing the age deconfounder for the target measures (lower panel).

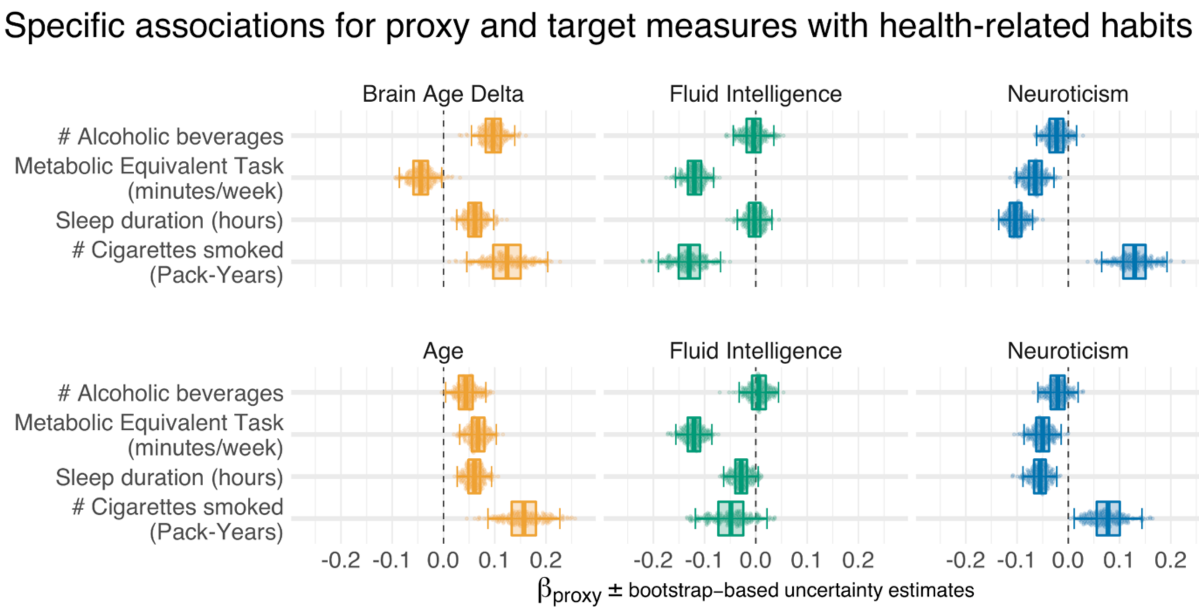

New Supplementary analyses.

Figure 2 supplement 2: We ran the same analysis for figure 2 using the brain-predicted age as such, not the delta as the proxy measure. Outputs are virtually identical.

Figure 2 supplement 3: We then added de-confounders for fluid intelligence and neuroticism and repeated the analysis for figure 2. Outputs were highly similar.

Figure 2 supplement 4: To provide a more rigorous comparison between proxies and targets as stimulated in **R1.4** and assess statistical significance of possible differences between proxies and targets, we have composed a big regression model including both the proxies and the targets as predictors. Across health outcomes, we found two frequent scenarios: 1) the proxies were found statistically significant, not the actual measures, suggesting that the proxies show improved SNR over the actual measures. 2) proxies and actual measures were found statistically significant, pointing at true additive effects. The overall picture depended on the health outcome.

Table S3: Detailed statistical inferences for the comprehensive proxy-target models are listed in this new table.

Table S4: In this context, the intrinsic link between proxies and targets may imply multicollinearity. Too much multicollinearity could potentially render the statistical testing procedure invalid. To test for multicollinearity we computed variance inflation factors (VIF) across the different models, which intuitively quantify how well each predictor can be approximated by a linear combination of the other predictors. The VIF is defined as 1 / (1 - R^2_i), where R^2_i is the coefficient of determination for the model that approximates the predictor i from all remaining predictors. A VIFs 1-5 counts as low to moderate (see extensive review and discussion in O'brien 2007, *Quality & Quantity* 41, 673-690). The observed VIFs were mostly around 1. The highest VIF was found for age and brain age with values around 3, which suggests

that age and brain age are linked to the other proxy measures. This is implicitly justifying our previous intuition of keeping age-deconfounders for the other proxy measures. More importantly, these control analyses suggest that the significance tests are not impacted by multicollinearity, which is also in line with reasonable standard errors produced by the big models from S3.

Based on these new analyses, we have updated the results section and have highlighted the theoretical considerations regarding asymmetries between the proxy measures.

Changes

In the introduction, the asymmetry has been emphasized:

> Extrapolating from these successes, we propose to build upon large datasets to extend the collection of health-related proxy measures, probing mental traits. For this end, we focused on constructs fundamentally different in terms of content and methodology.

In the main text, result section, a novel paragraph has been added to address the conceptual difference of the measures and consequences for the analysis:

> The three proxy measures are difficult to compare on an equal footing as a delta was considered for brain age only (the difference between predicted and actual age) and aging- specific deconfounding was applied. The brain-age delta is indeed the standard practice, theoretically justified as age is on a metric scale [50] for which the difference between the predicted and the measured value has a clear meaning. Such a difference is less obvious for variables with ordinal scales as implied by psychometric measures. Second, age has a pervasive influence on virtually any biomedical entity, which motivates controlling for its effect on proxy measures. To rule out that differences in proxy measures' associations to health-related behavior are driven by this methodological asymmetry, we repeated the main analysis from Figure 2, first, using the predicted age without computing the delta (Figure 2 – Figure supplement 2) and, second, introducing additional deconfounders for fluid intelligence and neuroticism (Figure 2 – Figure supplement 3). The resulting patterns were virtually unchanged, confirming that interpretations are robust.

In the main text, result section, a paragraph on big-picture statistical differences between proxies and targets has been generously expanded:

> A question that remains is whether the proxy measures bring additional value compared to the original target measures they were derived from. These original target measures showed similar associations to health behavior, with the same signs in most cases (Figure 2, B). At the same time, the ensuing patterns were more noisy, suggesting that empirically derived proxy measures yielded enhanced associations with health behavior. This inference may be difficult as differences between targets and proxies were not always easy to pinpoint visually. To implement a more rigorous statistical approach, we built comprehensive models of each respective health-related habit in which we used all proxies (predicted age, predicted fluid intelligence, predicted neuroticism) and all targets (age, fluid intelligence, neuroticism) simultaneously as predictors (Figure 2 – Figure supplement 4). The results show systematic additive effects of proxies and targets across the three target domains and the four health-habits. These trends are well-captured by the hypothesis tests

of the respective linear models (Table S3). As targets and proxies may be systematically intercorrelated, multicollinearity may corrupt these inferences. Inspection of variance inflation factors (VIF)— a measure that reveals how well a given predictor can be approximated by a linear combi- nation of the other predictors— argued in favor of low to moderate levels of multicollinearity (Table S4). Indeed, all VIF values fell between 3 and 1, whereas, classically, values above 5 or 10 are considered as thresholds [51] for pathological collinearity. This suggests that the model inferences are statistically sound.

In the main text, method section, health-related habits regression, a passage has been added describing the additional models (see blue highlight in screenshot).

consisting in residualizing a measure of interest (*e.g.* sleep duration) with regard to age through multiple regression with quadratic terms for age. To minimize computation on the held-out data, we first trained a model relating the score of interest to age on the validation set to then derive a de-confounding predictor for the held-out generalization data. The resulting de-confounding procedure for variables in the held-out data amounts to computing an age-residualized predictor $measure_{resid}$ from the measure of interest (*e.g.* sleep duration) by applying the following quadratic fit on the validation data:

$$measure_{validation} = age_{validation} \times \beta_{val1} + age^2_{validation} \times \beta_{val2} + \epsilon \quad (2)$$

The de-confounding predictor was then obtained by evaluating the weights β_{val1} and β_{val2} obtained from Equation 2 on the generalization data:

$$deconfounder = age_{generalization} \times \beta_{val1} + age^2_{generalization} \times \beta_{val2} \quad (3)$$

We performed this procedure for all target measures, to study associations not driven by the effect of age. For supplementary analyses presented in figure Figure 2 – Figure supplement 3, the same procedure was applied, substituting age for fluid intelligence and neuroticism, respectively.

Health-related habits regression. We then investigated the joint association between proxy measures of interest and health-[illegible]

we computed 10000 draws.

For supplementary analysis in Figure 2 – Figure supplement 2, the brain-predicted age instead of the delta was used:

$$measure = deconfounder \times \beta_1 + BrainAge \times \beta_2 + PredFluidInt \times \beta_3 + PredNeurot \times \beta_4 + \epsilon, \quad (5)$$

For supplementary analysis in Figure 2 – Figure supplement 3, additional deconfounders were introduced.

$$measure = deconfounder_{age} \times \beta_1 + BrainAge \times \beta_2 + deconfounder_{FI} \times beta_3 + PredFluidInt \times \beta_4 + deconfounder_N + \beta_5 + PredNeurot \times \beta_6 + \epsilon, \quad (6)$$

where $deconfounder_{FI}$ is the deconfounder for fluid intelligence and $deconfounder_N$ the deconfounder for neuroticsm following the procedure described in Equation 2 and Equation 3.

For supplementary analysis in Figure 2 – Figure supplement 4, proxies and targets were analyzed simultaneously.

$$measure = Age \times \beta_1 + BrainAge \times \beta_2 + FluidIntelligence \times beta_3 + PredFluidInt \times \beta_4 + Neuroticism + \beta_5 + PredNeurot \times \beta_6 + \epsilon, \quad (7)$$

The following items have been added to the supplement:

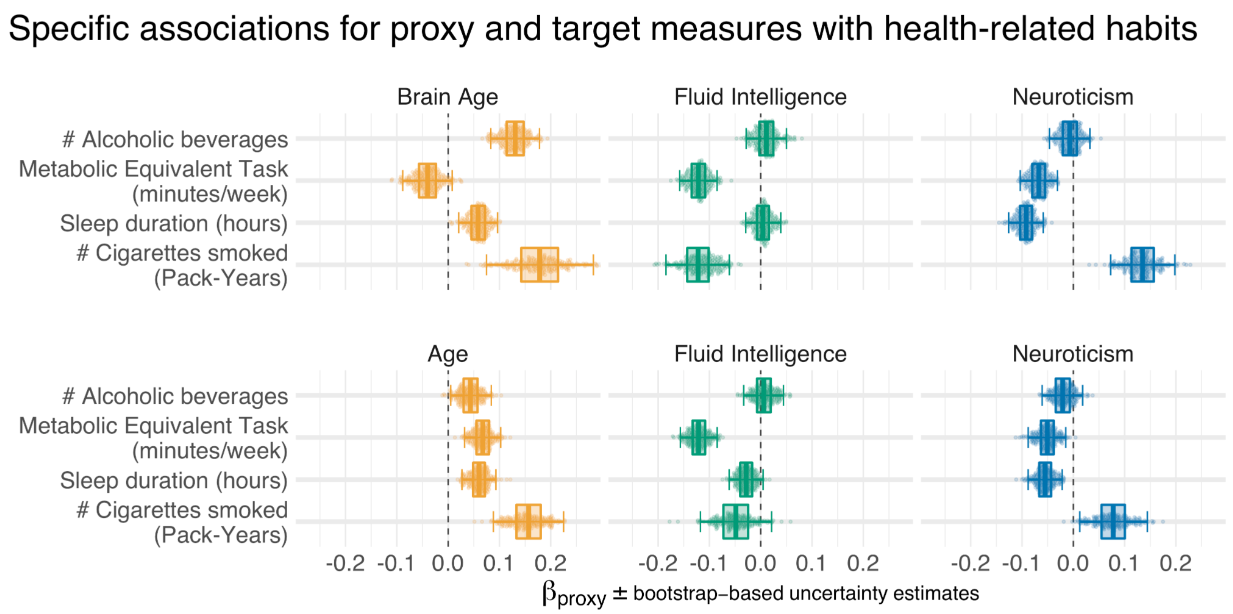

Figure 2 supplement 2: Conditional associations between proxy measures and health-related habits without explicit brain age delta. Conditional estimates using multivariate regression. Instead of the brain age delta, the brain-predicted age is included alongside an age-deconfounder as used in the main analysis. Same visual conventions as in Figure 2.

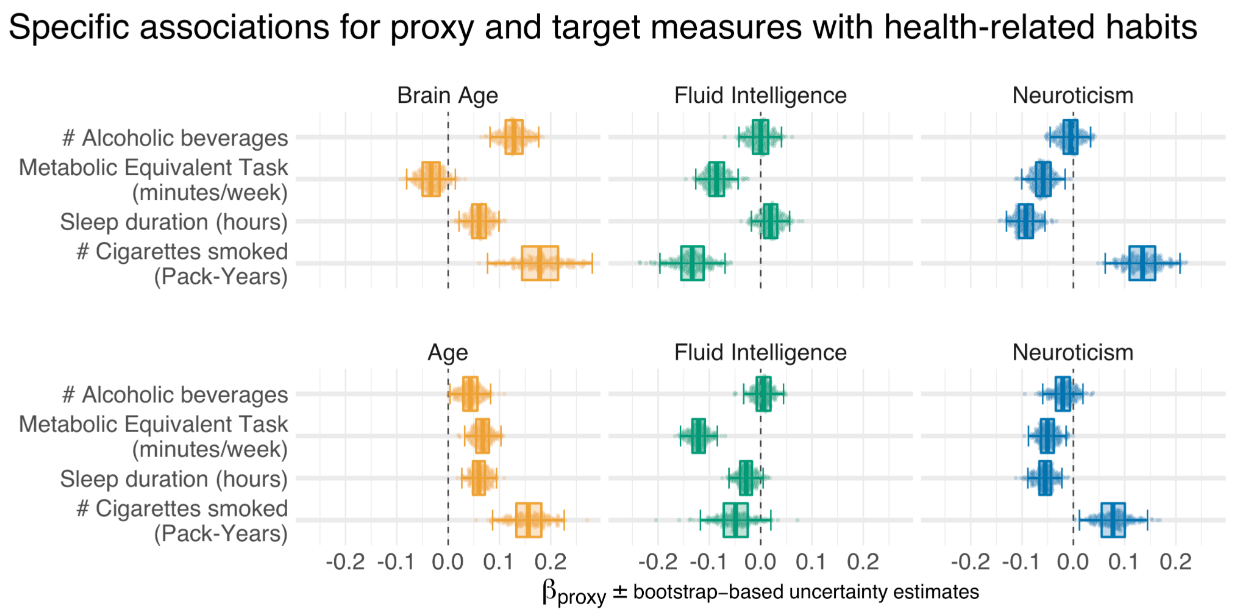

Figure 2 supplement 3 Conditional associations between proxy measures and health-related habits with-proxy-specific deconfounding. Conditional estimates using multivariate regression. Instead of the brain age delta, the brain-predicted age is included alongside an age-deconfounder as used in the main analysis. Moreover, predicted fluid intelligence and neuroticism are deconfounded for the target values at training time, analogous to the brain age predictions. Same visual conventions as in Figure 2.

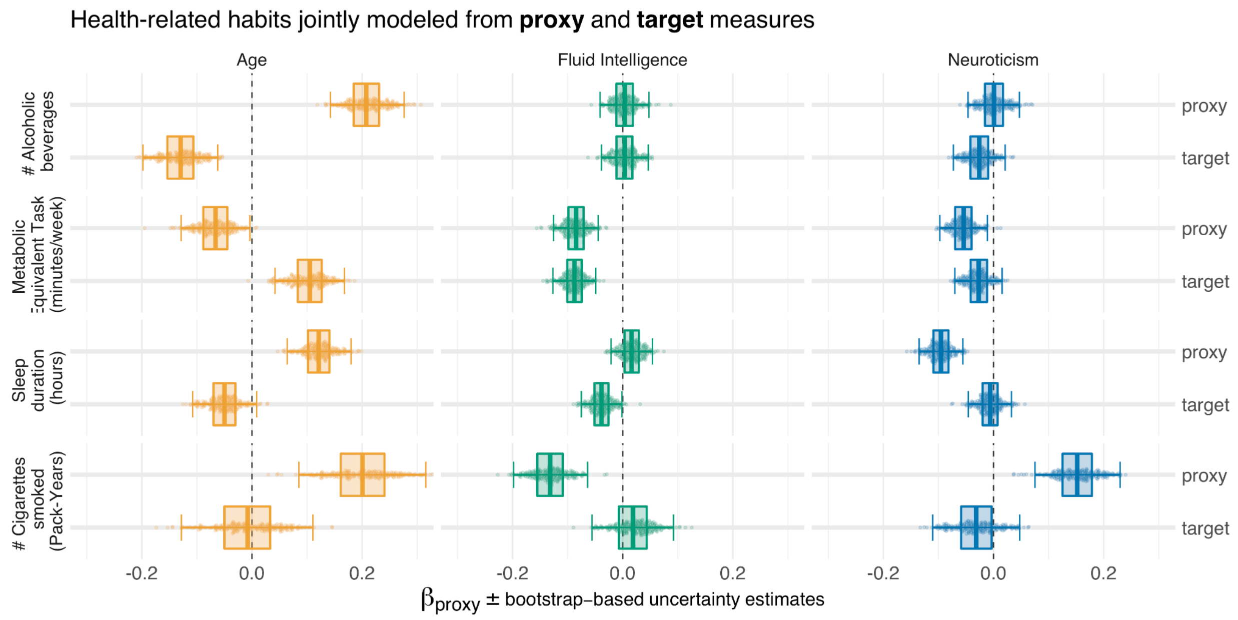

Figure 2 supplement 4 Joint modeling of health-related habits from proxy and target measures. Conditional estimates using multivariate regression. Every health-related habit (double rows) is modeled simultaneously from multiple proxies and targets. Same visual conventions as in Figure 2. Across health-habits, additive effects emerged not only for proxies and targets within the same measure (e.g. age) but also across measures (e.g. age and fluid intelligence).

For illustration, we shall consider two examples. Regarding alcohol consumption, age was the most important measure and opposite conditional effects were observed for the proxy and the target: Across the age range, people with higher brain age tended to drink more and across the brain-age range, older people tended to drink less. For smoking, the proxy measures were the most important variables with clear non-zero coefficients, pointing in different directions across target domains. Holding fluid intelligence and neuroticism constant (targets and proxies), people with higher brain age tended to have been smoking for a longer time. At the same time, those who scored lower on predicted fluid intelligence across the entire range of age, predicted age, measured fluid intelligence, predicted neuroticism and neuroticism, have been smoking for a longer time. Finally, those who scored higher on predicted neuroticism tended to smoke more across the ranges of all other measures.

Supplementary table 3

Table S3. Inferential statistics for joint proxy-target models of health-related habits

	Outcome			
	Alcohol	Activity	Sleep	Smoking
predicted Age	0.208***	−0.066**	0.121***	0.200***
	(0.034)	(0.032)	(0.029)	(0.058)
Age	−0.129***	0.105***	−0.050*	−0.008
	(0.035)	(0.032)	(0.030)	(0.060)
predicted Fluid Intelligence	0.004	−0.085***	0.016	−0.132***
	(0.022)	(0.021)	(0.019)	(0.035)
Fluid Intelligrence	0.003	−0.088***	−0.038**	0.018
	(0.022)	(0.020)	(0.019)	(0.038)
predicted Neuroticism	0.001	−0.054**	−0.095***	0.151***
	(0.024)	(0.022)	(0.020)	(0.040)
Neuroticism	−0.026	−0.027	−0.006	−0.031
	(0.024)	(0.022)	(0.020)	(0.041)
Constant	−0.001	0.018	0.017	−0.052
	(0.019)	(0.018)	(0.017)	(0.034)
Observations	2,687	3,022	3,504	896
R^2	0.016	0.031	0.020	0.071
Adjusted R^2	0.014	0.029	0.018	0.064
Residual Std. Error	1.004 (df = 2680)	0.997 (df = 3015)	0.992 (df = 3497)	0.992 (df = 889)
F Statistic	7.334*** (df = 6; 2680)	15.854*** (df = 6; 3015)	11.733*** (df = 6; 3497)	11.256*** (df = 6; 889)

Note: *p<0.1; **p<0.05; ***p<0.01

Supplementary table 4

Table S4. Variance Inflation Factors (VIF) for joint proxy-target models of health-related habits

	Alcohol	Activity	Sleep	Smoking
predicted Age	3.063	3.149	3.076	3.000
Age	3.108	3.181	3.123	3.070
predicted Fluid Intelligence	1.259	1.254	1.266	1.254
Fluid Intelligrence	1.220	1.223	1.229	1.229
predicted Neuroticism	1.451	1.457	1.460	1.590
Neuroticism	1.434	1.435	1.439	1.552

Minor concerns:

R1.5

1) In page 1 (two lines before reference 15), it seems that "to learn" is mis-spelled into "tolearn".

Reply R1.5: We thank the reviewer for having found this typo. We have corrected it.

R1.6

2) The author stated that there are repeated measures for subjects in UK biobank data. How the author tackles this issue in their data preprocessing? Using the last one or the first one or something else?

Reply R1.6 We thank reviewers for having raised this concern whose clarification may, indeed, help the reader.

Out of 500 000 participants, only a fraction has visited for repeated assessments. For instance, 10 000 participants were included in the first brain imaging release (Miller K et al. Nat. Neuroscience 2016; Thomas J. Littlejohns et al. Nat. Communications 2020). The visits are categorized as: initial assessment visit, repeated initial assessment, imaging visit and soon. These visits are coded as 0, 1, 2 and soon. In this paper, we relied mostly on assessments overlapping with imaging visit *i.e.* variables coded as 2.0 as can be seen from Table S5, *Appendix* 2.

Some variables used in the analysis display code 0, *e.g.* for target variables like age (21022-0.0), neuroticism (20127-0.0) or external validity variables. The reason for this is that these variables were not assessed during the follow up visits. We, therefore, relied on primary visit information.

Moreover, we explicitly checked that there is no overlap of the subjects between the validation and the generalization set.

Changes To clarify this point, we extended the subsection *Participants*.

> (...) we found 11175 participants who had repeated assessments overlapping with the first brain imaging release. Note that the features (sociodemographic variables) that we included in the analysis are measures that are self-reported during a follow-up imaging visit. (...) We made sure that the subjects used for model construction and generalization were strictly non-overlapping.

R1.7

3) The selection 5,587 out of all the 10,975 subjects for the modeling, while the left part is for the out-of-sample association analysis. The selection seems arbitrary. Can the author also show a learning curve, in which x is the sample size and y is the model's performance, to justify their choice is enough to train an accurate ML model?

R1.7 We thank reviewers for allowing us to clarify how we composed the training dataset.

First, we selected a percentage of training samples based on the random split half method from the scikit-learn package. As sample size differed across three targets, e.g., fewer brain images available for neuroticism prediction (see second paragraph at the subsection *Participants*) testing the out-of-sample associations on 50% of gathered samples was a pragmatic approach for obtaining sufficient data for model training and subsequent statistical inference, two complementary and distinct objectives in this investigation. Moreover, this is in line with evidence arguing in favor of larger test sets for mitigating optimism bias in estimation of out-of-sample performance (Flint et al 2021, Nat. Neuropsychopharmacology, Varoquaux et al. 2017, NeuroImage).

To explore the implications of our training and testing splits, as suggested in, we computed learning curves (displayed below). Across all targets, the results suggest that generalization performance started saturating around 1000 training samples, well below the final training samples. Adding more samples should not substantially improve generalization performance.

Changes To clarify this point, we have included a new figure as a supplement to the concept figure 1 and extended the method section on participants.

In the main text, methods, section Participants:

> The demographics are 51.6% female (5\,572) and 48.3% male (5\,403) and an age range between 40-70 years (with a mean of 55 years and standard deviation of 7.5 years). The data for model training were selected using a randomized split-half procedure yielding 5\,587 individuals. The remaining subjects were set aside as a held-out set for generalization testing (see section *Model development and generalization testing*). We made sure that the subjects used for model training and generalization testing were strictly non-overlapping. Learning curves documented that the training split was sufficiently large for constructing stable prediction models (Figure 1 - supplement 1) with profiles of performance similar to latest benchmarks on model complexity in the UK Biobank (Schulz et al 2020). Moreover, simulations and empirical findings suggest that larger testing sets are more effective at mitigating optimistic performance estimates (Flint et al 2021, Varoquaux et al. 2017). Together, this provided a pragmatic solution to the inference-prediction dilemma (Bzdok et al 2020; Bzdok et al., 2018) given the two objectives of the present investigation to obtain reasonably good predictive models, while at the same time performing parameter inference of statistical models developed on the left-out data.

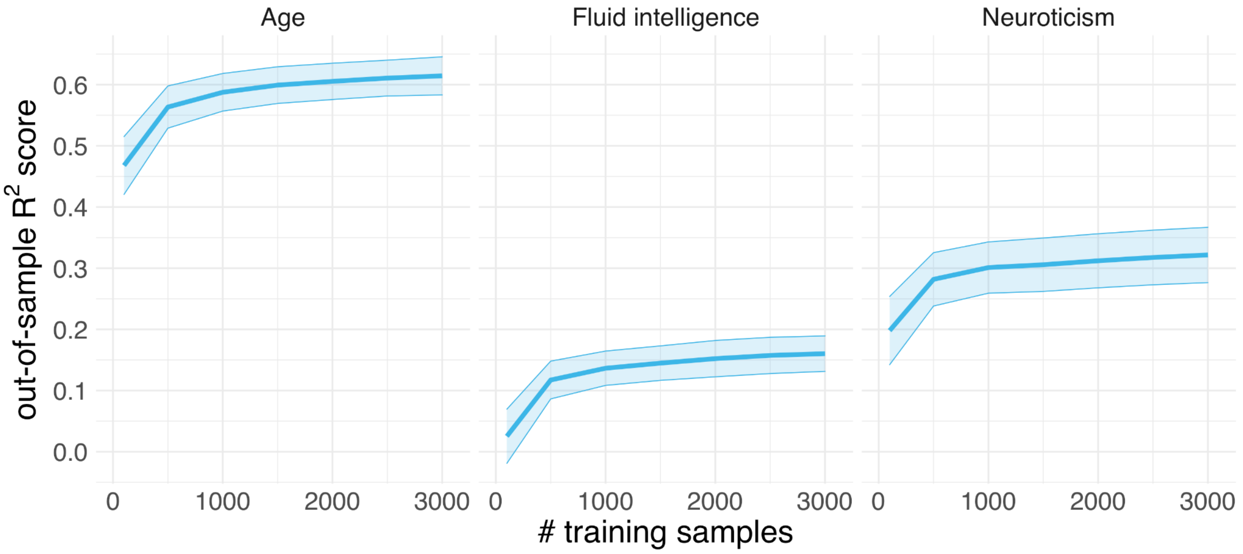

Figure 1 supplement 1 Learning curves on the random split-half validation used for model building. To facilitate comparisons, we evaluated predictions of age, fluid intelligence and neuroticism from a complete set of socio-demographic variables without brain imaging using the coefficient of determination R^2 metric (y-axis) to compare results obtained from 100 to 3000 training samples (x-axis). The cross-validation (CV) distribution was obtained from 100 Monte Carlo splits. Across targets, performance started to plateau after around 1000 training samples with scores virtually identical to the final model used in subsequent

analyses. These benchmarks suggest that inclusion of additional training samples would not have led to substantial improvements in performance.

R1.8

4) In the first paragraph of the "Methods" section, there are duplications.

Reply **R1.8:** We thank the reviewer for having pointed out the duplications. We have found and removed them.

R1.9

5) In the subsection of "Data acquisition" part, under the "target measures" paragraph, the age at the baseline recruitment is used as the outcome. However, in general, there is a gap between the age at baseline and the age when the MRI images were acquired. Does this matter for the data analysis in this manuscript.

Reply R1.9: We thank the reviewer for this thoughtful remark as we had not considered this issue in the first place. To investigate this age gap, we computed a distribution of the individual differences between the age at recruitment and the age at MRI-scan time. As expected, the difference was strictly positive, meaning that the MRI scan was always acquired after the first visit. The individual differences were highly rank-stable, suggesting that the age variables at recruitment and scan-time were equivalent from a statistical perspective. We subsequently fitted the prediction models from the main analysis using age at the time of the MRI-scan and directly compared the individual-specific predictions. As hypothesized, they were virtually identical. We have now made this point explicit in the main text and added a supplementary figure.

Changes

In methods, data acquisition, target measures:

> In the course of this work, a question that emerged concerned the size of the gap between age at baseline recruitment and MRI-scan time and its potential impact on the analysis. Supplementary checks indicated that the age gap was at least 5 years for most participants. Yet, from a statistical perspective, the two age measures turned out highly interchangeable (Figure S2) and global conclusions remained unchanged (Figure S3).

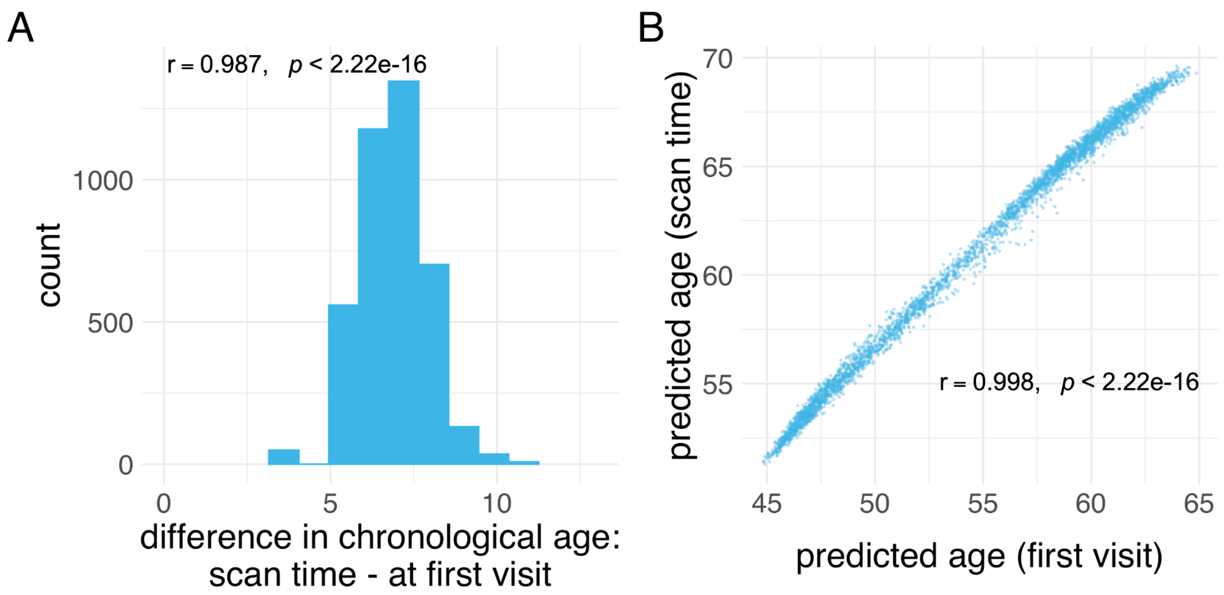

Figure S2 Investigating the age gap between the first visit and the MRI-visit time point. **(A)** Individual gap between age at first visit and MRI-scan time. MRI scans never happened at the first visit, leading to a strictly positive gap greater than five years for most participants. Pearson's correlation coefficient indicates high rank stability, suggesting that, from a statistical perspective, age at first visit and age at scan time are, essentially, interchangeable. **(B)** Direct comparison of individual-specific age predictions from brain images and sociodemographic data. Same model as in the main analysis (Figure 2). The emerging pattern of association summarized by Pearson's correlation coefficient suggests that predictions from models either trained on age at the first visit or at MRI-scan time are equivalent.

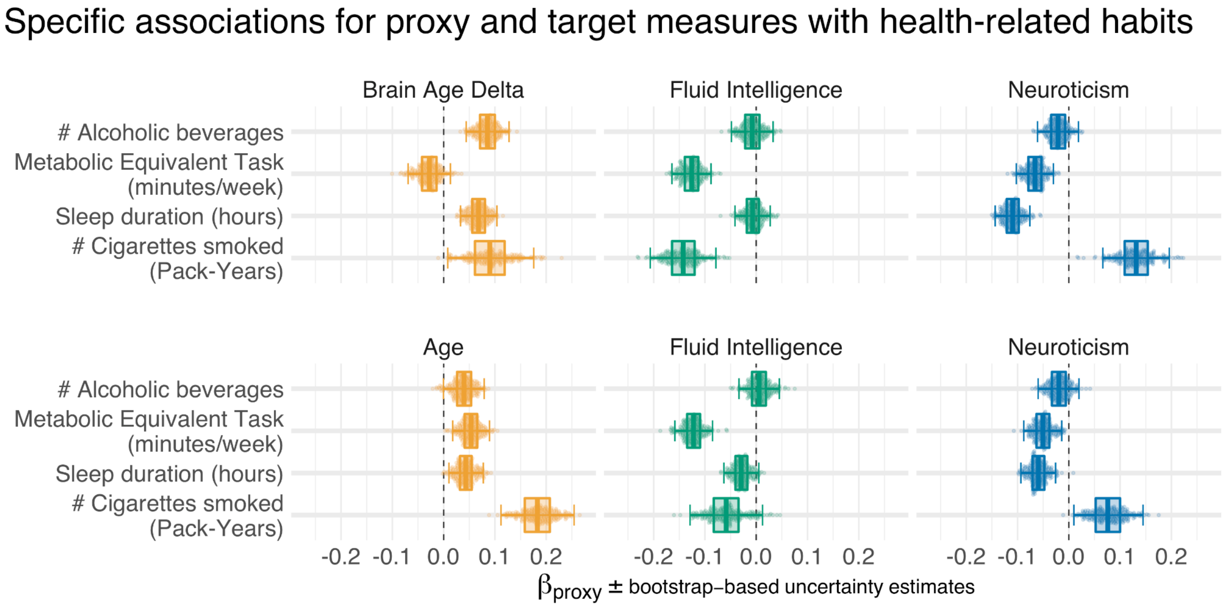

Figure S3 Proxy measures show systematic and complementary out-of-sample associations with health-related habits using age at MRI-scan time. The patterns observed in Figure 2 and global conclusions remain unchanged.

R1.10

6) For the classification analysis (paragraph "Classification analysis" in the subsection of "Comparing predictive models to approximate target measures", and the paragraph above the "Discussion" section), the thresholds selected to discretize the outcome variables are kind of arbitrary.

Reply R1.10: The division into groups follows the practical recommendations by Gelman and Hill 2007 for dichotomizing variables. The idea is to avoid having too many cases near the median boundary (where differences should be very subtle). We have now explained the idea and referenced Gelman and Hill's book.

Changes

In results, the relative importance of brain and sociodemographic data depends on the target:

> To investigate empirically-defined proxy measures beyond continuous regression, we performed binary classification of extreme groups obtained from discretizing the targets using the 33rd and 66th percentiles, following the recommendations by Gelman and Hill (2006) regarding discrete variable encoding strategies.

In method section, predictive model, classification analysis:

> We also performed classification analysis on the continuous targets. Adapting recommendations from Gelman and Hill 2005, we performed discrete variable encoding of the targets leading to extreme groups based on the 33rd and 66th percentiles (see
> Table 4) for the number of classification samples per group). This choice avoids including samples near the average outcome for which the input data may be indistinct.

Reviewer 2

All in all, this is a scientifically interesting study, but I think the presentation could be improved, by more clearly stating the aims of it, and by giving more insight in certain aspects of the 'proxy modeling'.

We thank the reviewer for the positive appreciation of our work and the constructive criticism. In response to this concern, we have carefully edited the main text, extended the discussion and taken efforts to make our theoretical standpoint more explicit.

R2.1

This manuscript reports on the results of a study that can be split into two parts. For this, it should be noted that the authors consider three categories of quantities. The first category are the input data, or 'predictors': (a) variables derived from MRI scans and (b) rich sociodemographic variables. The second category, or 'target variables', as the authors call them, include: (a) age, (b) fluid intelligence and (c) neuroticism. In the first part of the study, using machine learning, predictive models are built to predict the target variables from the input variables. The resulting predictions are called 'proxy measures'. For the second stage, a third category of variables is included, the 'real world health behaviours', such as alcohol use and physical activity. The authors now set out to predict these measures of behaviour based on the measures of the second category, either the 'real ones' or the 'proxies'. Thus, the question is, can alcohol use be better predicted by neuroticism determined from a questionnaire, or by the neuroticism proxy derived from MRI and sociodemographics? The main results are presented in Figure 2, and the conclusion made by the authors is that the proxies perform better than the real measures.The authors carry out additional analyses, including the study of the relative importance of MRI and sociodemographics. The authors suggest that these proxies may have clinical use in the future.
At first sight it may seem surprising that proxies perform better then the real measure in capturing the associations, but, as the authors mention, the real measures suffer from (measurement) noise and non-objectivity. However, the proxies are biased (in the sense of being to simple) and are thus less capable of modeling the (true) individual variation. I would have expected a more in depth discussion about this.

Reply to R2.1: We thank the reviewer for sharing this thoughtful impression with us. We now realize that the current wording of the paper may have led to the impression that we see the proxies as a drop-in-replacement for the actual targets (see also **R1.2** and **R1.4).** Instead, we propose that the proxies can be flexibly used to complement the original measures or act as replacements when the original measures are not available. Of note, in the context of machine learning, biased models often lead to better predictions as it can reduce uncertainty as in the classical bias/variance tradeoff. We have now extended the main text to make our perspective clearer to the reader and avoid this kind of misunderstanding and extended the discussion to better explain the potential mechanism by which proxy measures function.

Changes

In the discussion:

> A more complete view on how the proxy measures capture mental-health constructs emerges from their associations with real-world behavior (Figure 2). Indeed, the associations with proxy measures (Figure 2 A) were less noisy and more consistent than with the target measures (Figure 2 B), regardless of their approximation quality. This may seem surprising at first, but the target measures are themselves noisy and of imperfect validity. These measures correspond to traditional tests which, in practice, must be interpreted by an expert, actively confronting their output with broader information on the individual. For instance, IQ scores are typically normalized across age groups. However, extending such a normalization approach to many factors (socio-economic status, culture, gender) poses fundamental high-dimensional statistics challenges. Conversely, using machine learning to assemble proxy measures by mapping the targets to rich sociodemographic and brain data implicitly contextualizes them. In this respect, the resulting measures capture more general signal than the original tests. Here, machine learning could be seen as mimicking the work of a mental health expert who carefully compares psychometric results with other facts known about an individual and its reference population.

Conclusion:

> In population studies of mental health, individual traits are captured via lengthy assessments, tailored to specific brain and psychological constructs. We have shown that proxy measures built empirically from general-purpose data can capture these constructs and can improve upon traditional measures when studying real-world health patterns. Proxy measures can make psychological constructs available to broader, more ecological studies building on large epidemiological cohorts or real-world evidence. This can make the difference where psychological constructs are central to developing treatment and prevention strategies, but direct measures have not been collected.

Related changes reported above in response to R 1.2

In abstract (instead of advantage):

> We observed this complementarity of proxy measures and original measures when modeling from brain signals or sociodemographic data, capturing multiple health-related constructs.

In results section title (instead of outperforming):

> Complementing the original measures at characterizing real-life health-related habits

In results section:

> Our findings suggested that some information on psychological constructs can be assembled from general inputs not specifically tailored to measure these constructs, such as brain images and sociodemographic variables. The resulting proxy measures may be regarded as crude approximations of the psychological measures, but they can nonetheless capture essential aspects of the target constructs. To probe the external validity of the proxy-measures, we investigated their link with real-world behavior, e.g., sleep, physical exercise, alcohol and tobacco consumption on left-out data.

R2.2

Apart from this, there is an asymmetry in the way age is treated as compared to the other two target variables, intelligence and neuroticism. Age is a very hard measure, without any measurement error, and independent of the brain.

AND

R2.3

The other two targets, intelligence and neuroticism, are softer measures, and directly related to the brain. How does this influence the analyses and the results?

AND

R2.4

Indeed, not 'predicted age' is used as proxy, but 'brain age delta'. I would have liked to see more explanation and discussion about this.

Reply to **R2.2-2.4:** We thank the reviewer for sharing these thoughtful concerns. The reviewer is absolutely right that we have treated the target variables differently, as they are fundamentally different in terms of data generating mechanisms and measurement scale. The difference between target and proxy, e.g., the delta, can make sense where one deals with physical units (e.g. years for time) but may make less sense for ordinal or categorical variables (the effective measurement scale for intelligence and neuroticism is less clear; it is only clear that there are no physical units).

These points have also been brought up by reviewer 1 and that the reply, the edits on the manuscript and the additional analyses in response **R1.3-1.4** were explicitly designed to also address concerns **R2.2-2.4**. Moreover, we have revised the main text to make this asymmetry even more explicit, borrowing the proposed terminology of hard and soft measures. Finally, we have presented evidence in the main text that the proposed framework is flexible enough to handle these intrinsic differences. It turned out that very similar results are obtained even when the three measures are not treated asymmetrically but identically.

New Supplementary analyses.

Figure 2 supplement 2: We ran the same analysis for figure 2 using the brain-predicted age as such, not the delta as the proxy measure. Outputs are virtually identical.

Figure 2 supplement 3: We then added de-confounders for fluid intelligence and neuroticism and repeated the analysis for figure 2. Outputs were highly similar.

Figure 2 supplement 4: To provide a more rigorous comparison between proxies and targets as stimulated in **R1.4** and assess statistical significance of possible differences between proxies and targets, we have composed a big regression model including both the proxies and the targets as predictors. Across health outcomes, we found two frequent scenarios: 1) the proxies were found statistically significant, not the actual measures, suggesting that the proxies show improved SNR over the actual measures. 2) proxies and actual measures were found statistically significant, pointing at true additive effects. The overall picture depended on the health outcome.

Table S3: Detailed statistical inferences for the comprehensive proxy-target models are listed in this new table.

Table S4: In this context, the intrinsic link between proxies and targets may imply multicollinearity. Too much multicollinearity could potentially render the statistical testing procedure invalid. To test for

multicollinearity we computed variance inflation factors (VIF) across the different models, which intuitively quantify how well each predictor can be approximated by a linear combination of the other predictors. The VIF is defined as 1 / (1 - R^2_i), where R^2_i is the coefficient of determination for the model that approximates the predictor i from all remaining predictors. A VIFs 1-5 counts as low to moderate (see extensive review and discussion in O'brien 2007, *Quality & Quantity* 41, 673-690). The observed VIFs were mostly around 1. The highest VIF was found for age and brain age with values around 3, which suggests that age and brain age are linked to the other proxy measures. This is implicitly justifying our previous intuition of keeping age-deconfounders for the other proxy measures. More importantly, these control analyses suggest that the significance tests are not impacted by multicollinearity, which is also in line with reasonable standard errors produced by the big models from S3.

Based on these new analyses, we have updated the results section and have highlighted the theoretical considerations regarding asymmetries between the proxy measures.

Related changes reported above in response to reviewer 1

In the interest of a concise reply, only related changes to the text are reprinted below. For the related figures and tables, please consider the reply to points **R1.3-1.4** above.

In the introduction, the asymmetry has been emphasized:

> Extrapolating from these successes, we propose to build upon large datasets to extend the collection of health-related proxy measures, probing mental traits. For this end, we focused on constructs fundamentally different in terms of content and methodology.

In the main text, result section, a novel paragraph has been added to address the conceptual asymmetry of the measures and consequences for the analysis:

> The three proxy measures are difficult to compare on an equal footing as a delta was considered for brain age only (the difference between predicted and actual age) and aging-specific deconfounding was applied. The brain-age delta is in- deed the standard practice, theoretically justified as age is on a metric scale [50] for which the difference between the predicted and the measured value has a clear meaning. Such a difference is less obvious for variables with ordinal scales as im- plied by psychometric measures. Second, age has a pervasive influence on virtually any biomedical entity, which motivates controlling for its effect on proxy measures. To rule out that differences in proxy measures' associations to health-related behavior are driven by this methodological asymmetry, we repeated the main analysis from Figure 2, first, using the predicted age without computing the delta (Figure 2 – Figure supplement 2) and, second, introducing additional deconfounders for fluid intelligence and neuroticism (Figure 2 – Figure supplement 3). The resulting patterns were virtually unchanged, confirming that our interpretations are robust.

In the main text, result section, a paragraph on big-picture statistical differences between proxies and targets has been generously expanded:

> A question that remains is whether the proxy measures bring additional value compared to the original target measures they were derived from. These original target measures showed similar associations to health behavior, with the same signs in most cases (Figure 2, B). At the same time,

the ensuing patterns were more noisy, suggesting that empirically derived proxy measures yielded enhanced associations with health behavior. This inference may be difficult as differences between targets and proxies were not always easy to pinpoint visually. To implement a more rigorous statistical approach, we built comprehensive models of each respective health-related habit in which we used all proxies (predicted age, predicted fluid intelligence, predicted neuroticism) and all targets (age, fluid intelligence, neuroticism) simultaneously as predictors (Figure 2 – Figure supplement 4). The results show systematic additive effects of proxies and targets across the three target domains and the four health-habits. These trends are well-captured by the hypothesis tests of the respective linear models (Table S3). As targets and proxies may be systematically intercorrelated, multicollinearity may corrupt these inferences. Inspection of variance inflation factors (VIF)— a measure that reveals how well a given predictor can be approximated by a linear combination of the other predictors— argued in favor of low to moderate levels of multicollinearity (Table S4). Indeed, all VIF values fell between 3 and 1, whereas, classically, values above 5 or 10 are considered as thresholds [51] for pathological collinearity. This suggests that the model inferences are statistically sound.

R2.5

Finally, the suggested clinical use of the proxies is not supported well enough in my opinion. Maybe the authors could add more this discussion to this point as well.

We thank the reviewer for this suggestion. We absolutely agree with this impression. Unfortunately, relevant clinical data have not been available in the UK-Biobank. At the same time, the focus here is a public health perspective targeting individual differences in health, not pathology. We believe that, beyond the exact measures studied here, the framework, theory and methods proposed in this work can be readily applied with other measures and applied in the clinical setting, which is something that needs to be done in forthcoming studies. We have extended the discussion acknowledging this limitation in the dedicated section.

Changes

In limitations:

In terms of mental-health research, this study falls short of directly testing the clinical relevance of estimated proxy measures. Even in a very large general-population cohort such as the UK Biobank, there are only a few hundred diagnosed cases of mental disorders (ICD-10 mental-health diagnoses from the F chapter) with brain-imaging data available. As a result, we could not directly assess the performance of proxy measures in clinical populations.

The low number of diagnosed mental disorders in UK Biobank highlights the practical importance of studying mental health as a continuous, in addition to diagnosed conditions. Indeed, a public health perspective calls for targeting individual differences in health, not only pathology. Psychological constructs such as IQ and neuroticism are important factors of the epidemiology of psychiatric disorders [38, 30, 29, 67], and accelerated brain aging is associated with various neurological conditions [18, 17, 25]. Yet, few cohorts come with extensive neuropsychological

testing. Validated proxies of these constructs open the door to including them in epidemiological studies as secondary outcomes or additional explanatory variables.”

Related changes reported above in earlier response to related concerns by reviewer 1

In the discussion:

A more complete view on how the proxy measures capture mental-health constructs emerges from their associations with real-world behavior (Figure 2). Indeed, the associations with proxy measures (Figure 2 B) were less noisy and more consistent then with the target measures (Figure 2 A), regardless of their approximation quality. This may seem surprising at first, but the target measures are themselves noisy and of imperfect validity. These measures correspond to traditional tests which, in practice, must be interpreted by an expert, actively confronting their output with broader information on the individual. For instance, IQ scores are typically normalized across age groups. Extending such normalization approach to many factors (socio-economic status, culture, gender) poses however fundamental challenges of high-dimensional statistics. Conversely, using machine learning to assemble proxy measures by mapping the targets to rich sociodemographic and brain data implicitly contextualizes them. In this respect, the resulting measure captures more general signal than the original tests. Here, machine learning could be seen as mimicking the work of a mental health expert who carefully compares psychometric results with other facts known about an individual and its reference population.

Conclusion:

In population studies of mental health, individual traits are captured via lengthy assessments, tailored to specific brain and psychological constructs. We have shown that proxy measures built empirically from general-purpose data can capture these constructs and can improve upon traditional measures when studying real-world health patterns. Proxy measures can make psychological constructs available to broader more ecological studies building on large epidemiological cohorts or real-world evidence. This can make the difference where psychological constructs are central to developing treatment and prevention strategies, but direct measures have not been collected.

Click here to access/download;Figure;figure_zero_concept.pdf

Figure 1

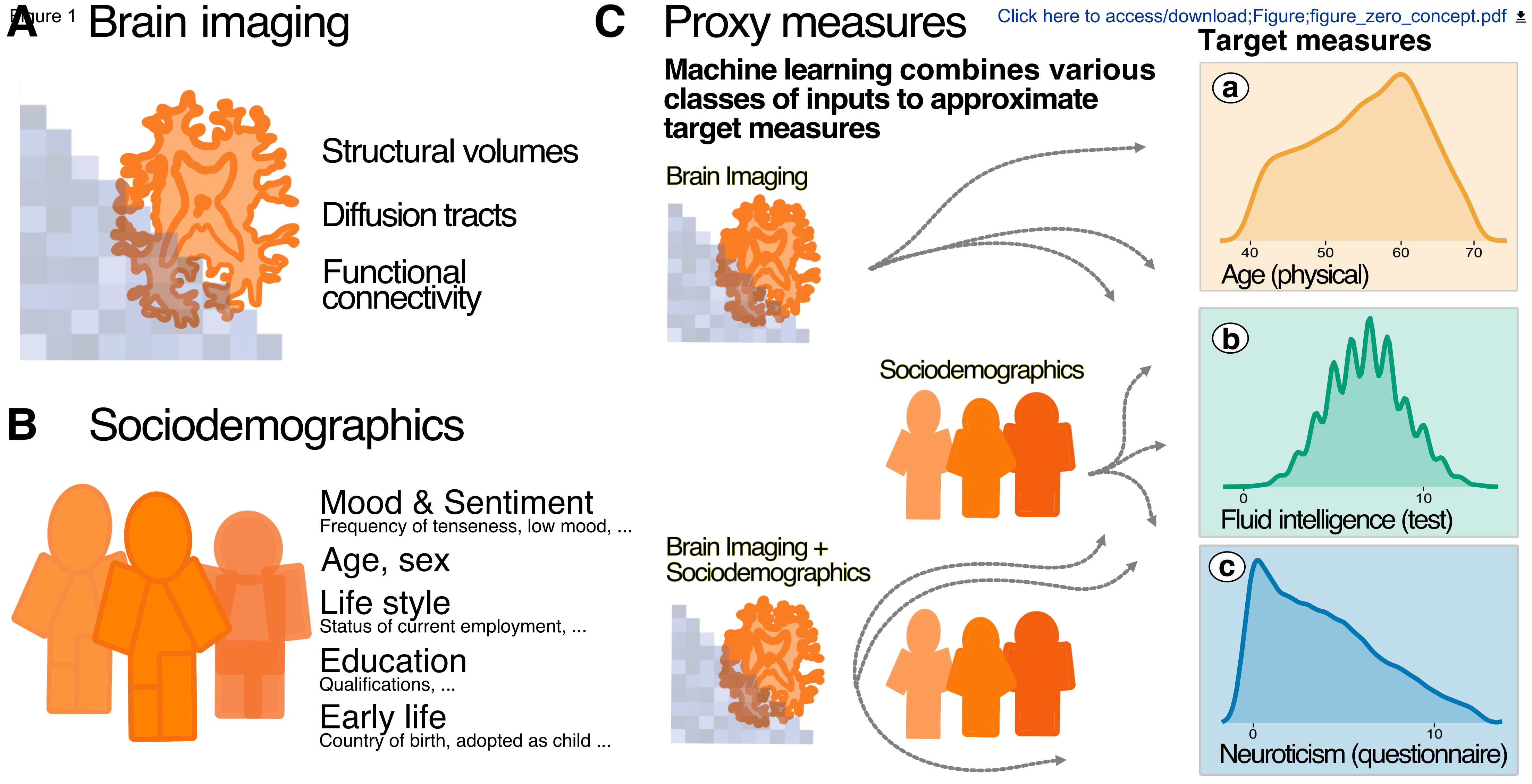

Figure 3

Click here to access/download;Figure;figure_summary_validation.pdf

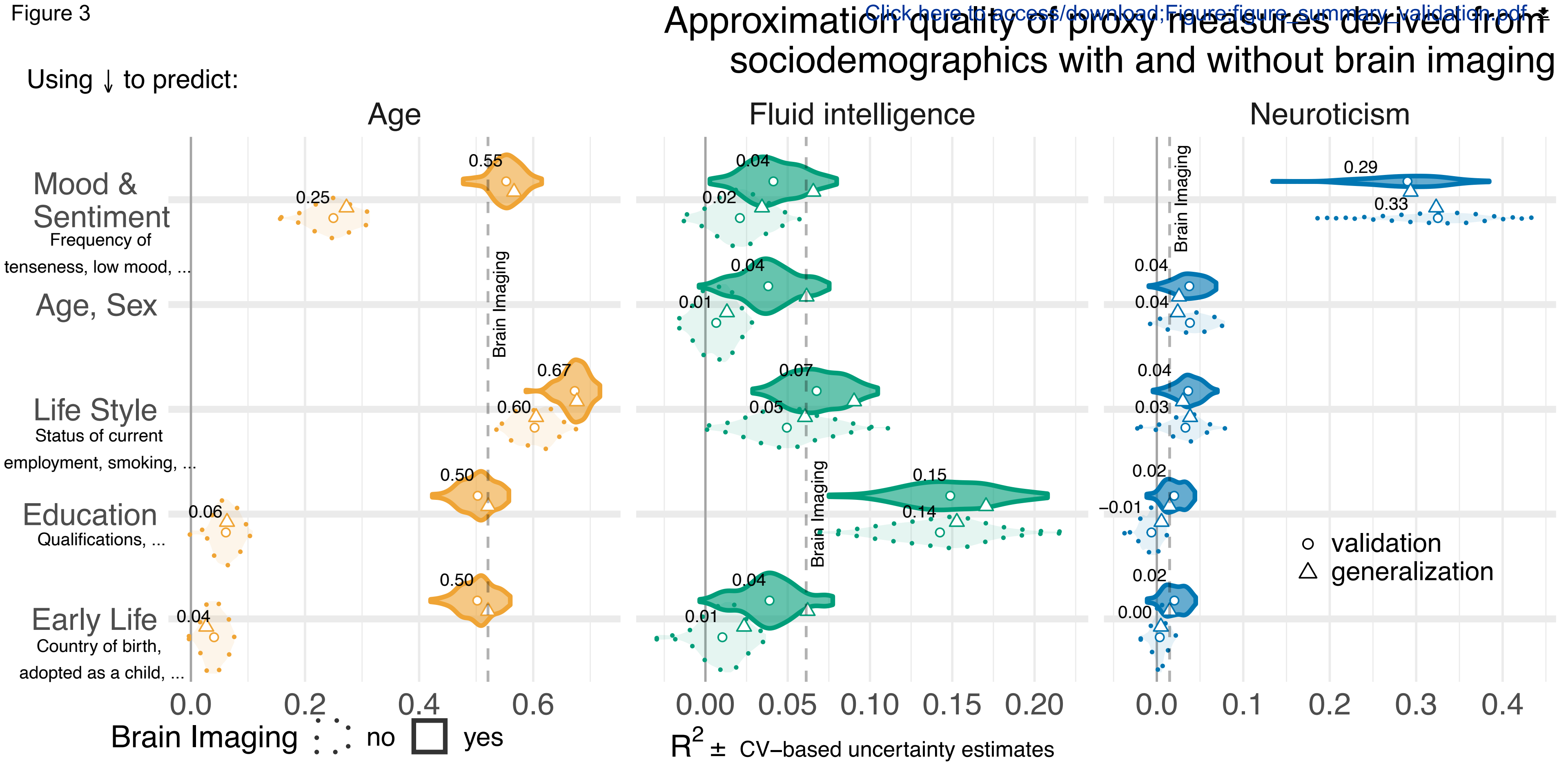

Figure 4

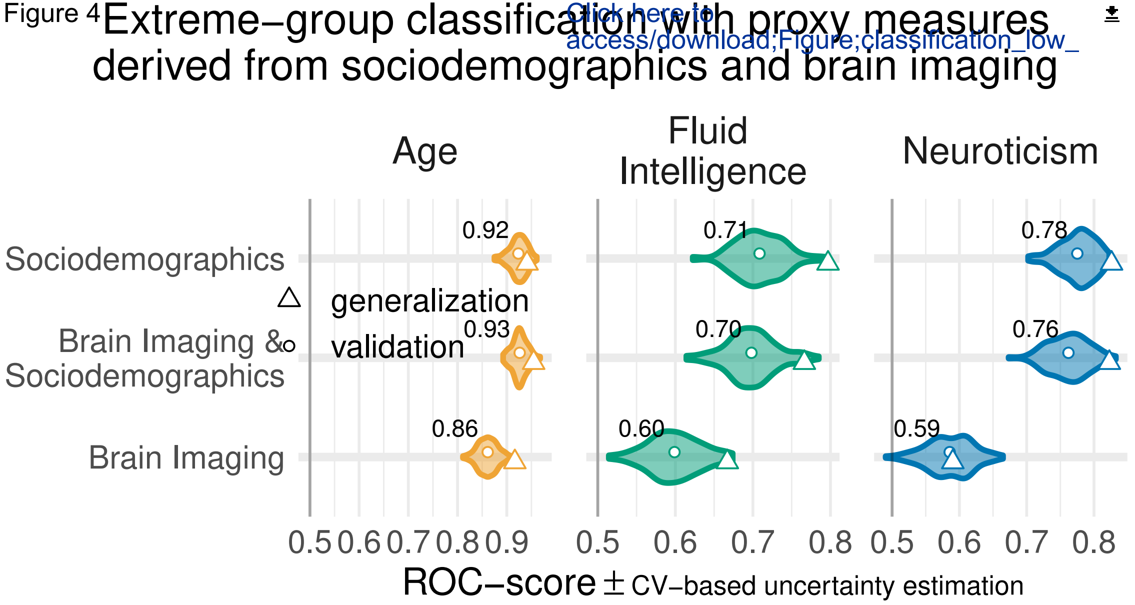

Click here to access/download;Figure;classification_low_

Figure 2 Click here to access/download;Figure;figure_2.pdf

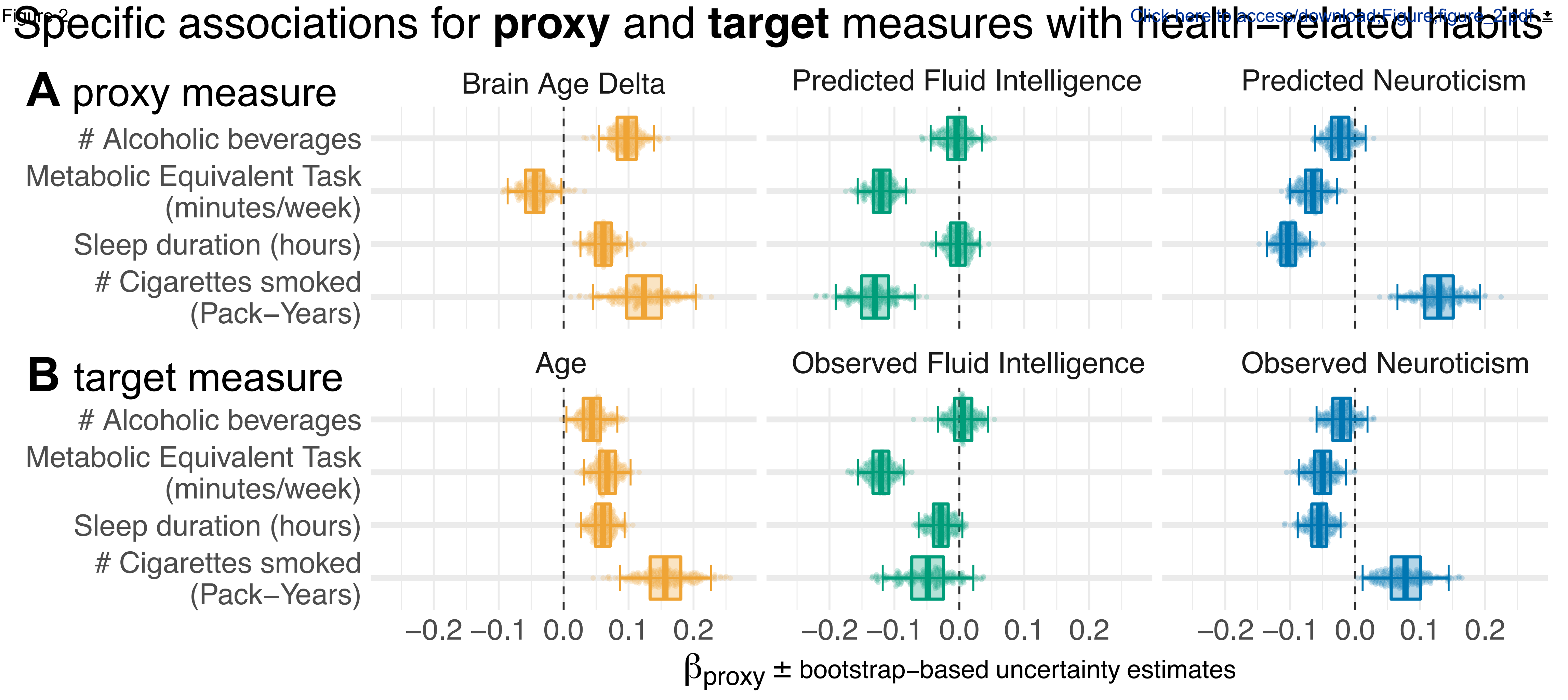

Click here to access/download
Supplementary Material
supplement_r1.pdf

Cover Letter

Click here to access/download;Personal Cover;dadi_cover_letter-signed.pdf

Paris, le 13 juillet 2021

Dr. Denis-Alexander Engemann
research scientist, INRIA-Saclay
www.denis-engemann.de
denis-alexander.engemann@inria.fr

Dear Dr. Zauner, Dear Editors of *GigaScience*

Please, find for your consideration the revision of ourmanuscript entitled "Population modeling with machine learning can enhance measures of mental health".

We would like thank the editor and the reviewers for the thoughtful feedback and constructive criticism on our work. We have taken the revisions as an important opportunity to improve our manuscript and would like to highlight two points that both reviewers and the editor have emphasized:

1. both reviewers had concerns regarding the far-reaching conclusion: the **"findings suggested that psychological constructs can be approximated from brain images and sociodemographic variables."** Considering these concerns, we added methodological and conceptual clarifications, discussed our findings more carefully and revised the general conclusions to express greater nuance.

2. both reviewers also pointed out that the phenotype **"age / brain age"** is different from the other measurements. We conducted multiple control analysis to investigate this point and clarify the differences, both, conceptually and statistically. The wealth of results and related discussion points are fully reported in the revised manuscript.

This required substantial reanalysis demanding several weeks of computation time and led us to including 6 new supplementary figures and 2 new supplementary tables. A detailed display of the changes in the manuscript is available in the file 'diff-with-original-submission.pdf' enclosed with the resubmission.

This work is not under consideration elsewhere and that we believe that GigaScience with its interdisciplinary and innovation-driven culture would be our preferred outlet to disseminate this work.

Sincerely yours,
Kamalaker Dadi, Gaël Varoquaux, Josselin Houenou, Danilo Bzdok, Bertrand Thirion & Denis A. Engemann

RESEARCH CENTRE
SACLAY ÎLE-DE-FRANCE
1 Rue Honoré
d'Estienne d'Orves
91120 Palaiseau
Phone: +33 (0)1 72 92 59 00
Fax: +33 (0)1 74 85 42 42

www.inria.fr

PDF of LaTeX diff with Original Submission
Click here to access/download;Personal Cover;diff-with-original-submission.pdf

GigaScience, 20xx, 1–24

doi: xx.xxxx/xxxx
Manuscript in Preparation
Paper

PAPER

Population modeling with machine learning can enhance measures of mental health

Kamalaker Dadi[1], Gaël Varoquaux[1,2,3], Josselin Houenou[4,5], Danilo Bzdok[1,3,6], Bertrand Thirion[1] and Denis Engemann[1,7,*]

[1]Inria, CEA, Neurospin, Parietal team, Univ. Paris Saclay, 91120 Palaiseau, France and [2]Montréal Neurological Institute, McGill University, Montreal, Canada and [3]Mila - Quebec Artificial Intelligence Institute, Canada and [4]CEA, NeuroSpin, Psychiatry Team, UNIACT Lab, Univ. Paris Saclay and [5]APHP, Mondor University Hospitals, Psychiatry Dept, INSERM U955 Team 15 "Translational Psychiatry", Créteil, France and [6]Department of Biomedical Engineering, Montreal Neurological Institute, Faculty of Medicine, McGill University, Montreal, Canada and [7]Department of Neurology, Max Planck Institute for Human Cognitive and Brain Sciences, Germany

[*] denis.engemann@gmail.com

Abstract

Background Biological aging is revealed by physical measures, *e.g.*, DNA probes or brain scans. Instead, individual differences in mental function are explained by psychological constructs, e.g., intelligence or neuroticism. These constructs are typically assessed by tailored neuropsychological tests that build on expert judgement and require careful interpretation. Could machine learning on large samples from the general population be used to build proxy measures of these constructs that do not require human intervention? **Results** Here, we built proxy measures by applying machine learning on multimodal MR images and rich sociodemographic information from the largest biomedical cohort to date: the UK Biobank. Objective model comparisons revealed that all proxies captured the target constructs and were as useful, and sometimes more useful than the original measures for characterizing real-world health behavior (sleep, exercise, tobacco, alcohol consumption). We observed this ~~advantage~~ complementarity of proxy measures ~~over the~~ and original measures when modeling from brain signals or sociodemographic data, capturing multiple health-related constructs. **Conclusions** Population modeling with machine learning can derive measures of mental health from brain signals and questionnaire data, which may ~~replace or complement~~ complement or even substitute for psychometric assessments in clinical populations.

Key words: Mental Health, Proxy Measures, Machine Learning, Sociodemographic ~~factors~~Factors, Brain Imaging

Background

Quantitative measures of mental health remain challenging despite substantial efforts [1]. The field has struggled with unstable diagnostic systems [2], small sample sizes [3], and reliance on case-control studies [4]. Perhaps most importantly, mental health cannot be measured the same way diabetes can be assessed through plasma levels of insulin or glucose. Psychological constructs, *e.g.*, intelligence or anxiety, can only be probed indirectly through lengthy expert-built questionnaires or structured examinations by a specialist. Though questionnaires often remain the best accessible option, their capacity to measure a construct is limited [5]. In practice, as full neuropsychological evaluation is not automated process but relies

Compiled on: July 14, 2021.
Draft manuscript prepared by the author.

Key Points

- We applied machine learning on more than 10.000 individuals from the general population to define empirical approximations of health-related psychological measures that do not require human judgment.
- We found that machine-learning enriched the given psychological measures via approximation from brain and sociodemographic data: Resulting proxy measures related as well or better to real-world health behavior than the original measures.
- Model comparisons showed that sociodemographic information contributed most to characterizing psychological traits beyond aging.

on expert judgement to confront multiple answers and interpret them in the context of the broader picture, such as cultural background of the participant. While the field of psychometrics has thoroughly studied the validity of psychological constructs and their measure [6, 7, 8], the advent of new biophysical measurements of the brain brings new promises [9, 10, 11]. The growth of biobanks and advances in machine learning open the door to large-scale validation of psychological measures for mental health research [12], and the hope to develop more generalizable models [13]. Yet, to be reliable, machine learning needs large labeled datasets [14]. Its application ~~tolearn~~ to learn imaging biomarkers of mental disorders is limited by the availability of large cohorts with high-quality neuropsychiatric diagnosis [15].

By comparison, it is easier to collect data on the general population without information on clinical conditions. For brain health, such data has lead to developing proxy measures that ~~quantifying~~ quantify biological aging [16, 17, 18, 11, 19, 20, 21, 22]. One counterintuitive aspect of the methodology is that measures of biological aging can be obtained by focusing on the age of a person, which is known in advance and in itself not interesting. Yet, by predicting the age, machine-learning can capture the relevant signal. Based on a population of brain images, it extracts the best guess for the age of a person, indirectly positioning that person within the population. Individual-specific prediction errors therefore reflect deviations from what is statistically expected [23]. The brain of a person can look similar to the brains commonly seen in older (or younger) people. The resulting brain-predicted age reflects physical and cognitive impairment in adults [24, 17, 16] and reveals neurodegenerative processes [22, 25]. Can this strategy of biomarker-like proxy measures be extended to other targets beyond the construct of aging? Extrapolating from these successes, we propose to build upon large datasets to extend the collection of health-related *proxy measures*, probing mental traits. For this end, we focused on constructs fundamentally different in terms of content and methodology.

One high-stake target is intelligence, which is measured through socially administered tests and is one of the most extensively studied constructs in psychology. Fluid intelligence refers to the putatively culture-free, heritable and physiological component of intelligence [26, 27] and is a latent construct designed to capture individual differences in cognitive capacity. It has been robustly associated with neuronal maturation and is typically reflected in cognitive-processing speed and working-memory capacity [28]. Applied to psychiatric disorders, it may help characterize psychosis, bipolar disorder, and substance abuse [29, 30].

Neuroticism is a second promising target. As a key representative of the extensively studied Big Five personality inventory, neuroticism has a long-standing tradition in the psychology of individual differences [31, 32]. Neuroticism is measured using self-assessment questionnaires and conceptualized as capturing dispositional negative emotionality including anxiety and depressiveness [33]. It has been inter-culturally validated [26, 34] and population-genetics studies have repeatedly linked neuroticism to shared genes [35, 36, 37]. Neuroticism was shown useful in psychometric screening and supports predicting real-world behavior [38, 39].

Despite strong population-level heritability [40, 41], the link between psychological constructs, brain function and genetics is still being actively researched [42, 33]. Empowered by emerging large-scale datasets, current attempts to predict fluid intelligence or neuroticism from thousands of MRI scans argue in favor of heterogeneity and weakly generalizing effects [43, 44]. This stands in contrast to the remarkable performance obtained when predicting psychometric data from language-based inputs captured by Twitter and Facebook user data [45, 46]. As MRI acquisitions can be difficult to come by in certain populations, the promises of social-media data are appealing. However, such data may lead to measurement and selection biases difficult to control. Instead, background sociodemographic data may provide an easily accessible alternative for contextualizing the heterogeneity of psychological traits [47].

Another challenge is that psychological traits are often measured using arbitrary non-physical units, *e.g.* education degree or monthly income. In fact, society treats individual differences as categorical or continuous, depending on the practical context. While personality has been proposed to span a continuum [48], psychiatrists treat certain people as patients and not others [49]. Therefore, a measure that performs globally poorly at a continuous scale can be sufficient to distinguish subgroups as it may be informative around the boundary region between certain classes, *e.g.*, pilots who should fly and who should not. Choosing the granularity with which to gauge psychological constructs is diffcult.

Confronting the promises of population phenotyping with the challenges of measuring psychological traits raises the following questions: 1) How well can various constructs related to mental health be approximated from general-purpose inputs not designed to measure specific latent constructs? 2) Can the success of brain age be extended to other proxy measures capturing complementary facets of mental health? 3) What is the relative merit of brain imaging and sociodemographics? We tackled these questions by using machine learning to craft *proxy measures* in order to approximate well-characterized *target measures* from brain-imaging and sociodemographic data. We studied age, fluid intelligence, and neuroticism~~– classical targets which have beenserving as proxy measures~~. These targets have been, traditionally, considered as proxies for mental health ~~in the first place~~and are fundamentally different in terms of scope and nature. Figure 1 summarizes our approach. Results suggest that, as with brain age, proxy measures can bring value for the study of mental health that goes beyond approximating an available measure.

Results: validity of proxy measures

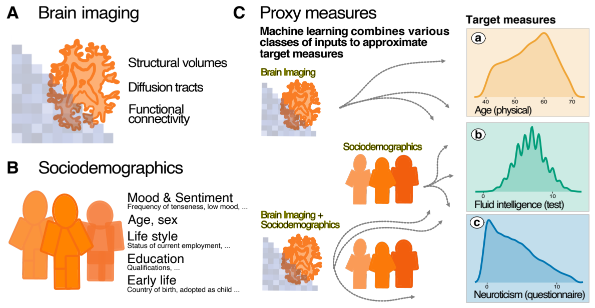

Figure 1. Methods overview: building and evaluating proxy measures We combined multiple brain-imaging modalities (**A**) with sociodemographic data (**B**) to approximate health-related biomedical and psychological constructs (**C**), *i.e.*, brain age (accessed through prediction of chronological age), cognitive capacity (accessed through a fluid-intelligence test) and the tendency to report negative emotions (accessed through a neuroticism questionnaire). We included the imaging data from the 10 000-subjects release of the UK biobank. Among imaging data (**A**) we considered features related to cortical and subcortical volumes, functional connectivity from rfMRI based on ICA networks, and white-matter molecular tracts from diffusive directions (see Table 1 for an overview about the multiple brain-imaging modalities). We then grouped the sociodemographic data (**B**) into five different blocks of variables related to self-reported mood & sentiment, primary demographics, lifestyle, education, and early-life events (Table 2 lists the number of variables in each block). Subsequently, we systematically compared the approximations of all three targets based on either brain images and sociodemographics in isolation or combined (**C**) to evaluate the relative contribution of these distinct inputs. Models were developed on 50% of the data (randomly drawn) based on random forest regression guided by Monte Carlo cross-validation with 100 splits (see section Model Development and Generalization Testing). We assessed generalization using the other 50% of the data as fully independent out-of-sample evaluations (see section Statistical Analysis). Learning curves suggested that this split-half approach provided sufficient data for model construction (Figure 1 – Figure supplement 1).

~~Outperforming~~ Complementing the original measures at characterizing real-life health-related habits

To approximate age, fluid intelligence and neuroticism, we applied random-forest regression on sociodemographic data and brain images. The data was split into *validation data* for model construction (see section Model Development and Generalization Testing) and *generalization data* for statistical inference on out-of-sample predictions with independent data (see section Statistical Analysis). Our findings suggested that some information on psychological constructs can be ~~approximated from brain images and sociodemographic variables – inputs not tailored to specifically~~ assembled from general inputs not specifically tailored to measure these constructs~~. We then applied the derived proxy measureson the~~ , such as brain images and sociodemographic variables. The resulting proxy measures may be regarded as crude approximations of the psychological measures, but they can nonetheless capture essential aspects of the target constructs. To probe the external validity of the proxy measures, we investigated their link with real-world behavior, e.g., sleep, physical exercise, alcohol and tobacco consumption on left-out data~~to gauge how well they capture~~ . To probe the external validity of the proxy-measures, we investigated their link with real-world behavior, *e.g.*, sleep, physical exercise, alcohol and tobacco consumption on left-out data. To relate such health behaviors to our proxy measures, we modeled them separately as weighted sums of predicted brain-age delta, fluid intelligence and neuroticism using multiple linear regression (section Statistical Analysis). To avoid circularity, we used the out-of-sample predictions for all proxy measures (section Model Development and Generalization Testing).

The estimated regression coefficients (partial correlations), revealed complementary associations between the proxy measures and health-related behavior (Figure 2). Similar patterns arise when considering proxy measures in isolation (Figure 2 – Figure supplement 1). Compared to other proxy measures, elevated brain-age delta was associated with increased alcohol consumption (Figure 2, first row). Levels of physical exercise were consistently associated with all three predicted targets, suggesting additive effects (Figure 2, second row). For fluid intelligence, this result, counter-intuitive from the health standpoint, could imply that higher test scores reveal a more sedentary life style. Increased sleep duration consistently went along with elevated brain age delta, but lower levels of predicted neuroticism (Figure 2, third row). This may seem counter-intuitive, but is conditional on neuroticism showing a negative link with sleep duration. No consistent effect emerged for fluid intelligence. Numbers of cigarettes smoked was independently associated with all predicted targets (Figure 2, last row): Intensified smoking went along with elevated brain age delta and neuroticism but lower fluid intelligence.

The ~~question remains~~ three proxy measures are difficult to compare on an equal footing as a delta was considered for brain age only (the difference between predicted and actual age) and aging-specific deconfounding was applied. The brain-age delta is indeed the standard practice, theoretically justified as age is on a metric scale [50] for which the difference between the predicted and the measured value has a clear meaning. Such a difference is less obvious for variables with ordinal scales as implied by psychometric measures. Second, age has a pervasive influence on virtually any biomedical entity, which motivates controlling for its effect on proxy measures. To rule out that differences in proxy measures' associations to health-related behavior are driven by this methodological asymmetry, we repeated the main analysis from Figure 2, first, using the predicted age without computing the delta (Figure 2 – Figure supplement 2) and, second, introducing additional deconfounders for fluid intelligence and neuroticism (Figure 2 – Figure supplement 3). The resulting patterns were virtually unchanged, confirming that interpretations are robust.

A question that remains is whether the proxy measures bring additional value compared to the original target measures they were derived from. These original target measures showed similar associations to health behavior, ~~often,~~ with the same signs in most cases (Figure 2, B). ~~However~~At the same time, the ensuing patterns were more noisy, suggesting that empirically derived proxy measures ~~indeed yield~~ yielded enhanced associations with health behavior. This inference may be difficult as differences between targets and proxies were not always easy to pinpoint visually. To implement a more rigorous statistical approach, we built comprehensive models of each respective health-related habit in which we used all proxies (predicted age, predicted fluid intelligence, predicted neuroticism) and all targets (age, fluid intelligence, neuroticism) simultaneously as predictors (Figure 2 – Figure supplement 4). The results show systematic additive effects of proxies and targets across the three target domains and the four health-habits. These trends are well-captured by the hypothesis tests of the respective linear models (Table S3). As targets and proxies may be systematically intercorrelated, multicollinearity may corrupt these inferences. Inspection of variance inflation factors (VIF)— a measure that reveals how well a given predictor can be approximated by a linear combination of the other predictors— argued in favor of low to moderate levels of multicollinearity (Table S4). Indeed, all VIF values fell between 3 and 1, whereas, classically, values above 5 or 10 are considered as thresholds [51] for pathological collinearity. This suggests that the model inferences are statistically sound.

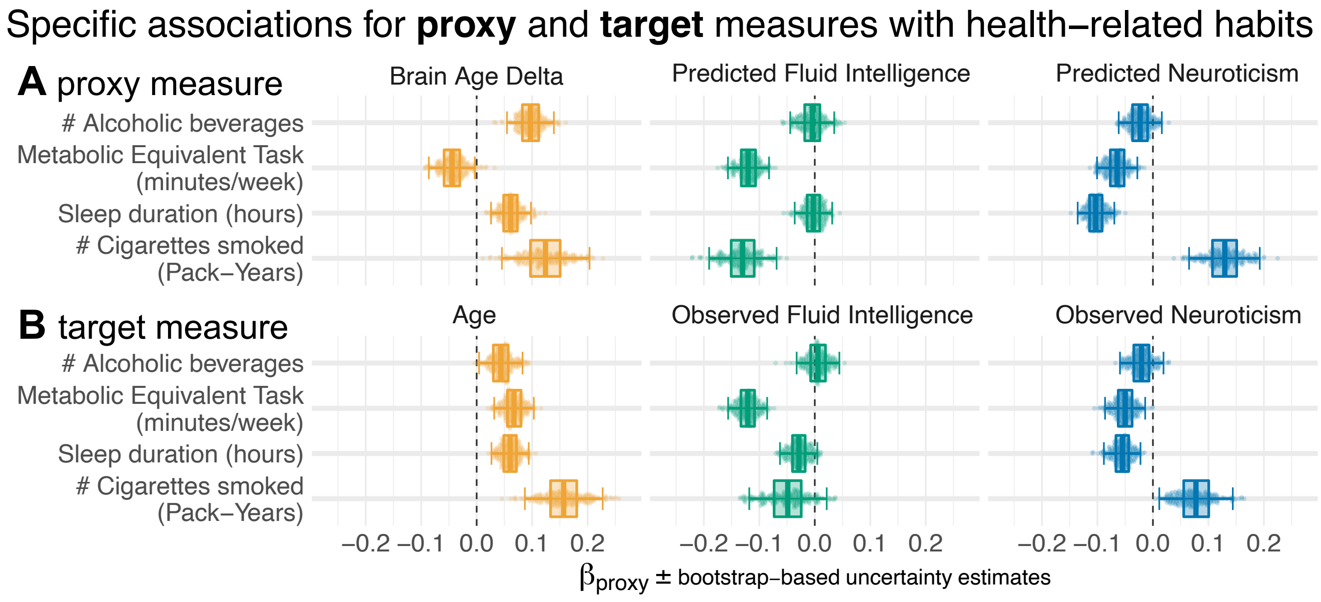

Figure 2. Proxy measures show systematic and complementary out-of-sample associations with health-related habits. We probed the external validity of all three proxy measures (brain age, fluid intelligence, neuroticism) based on a combination of brain images and all sociodemographic factors (see **Figure 1** ~~and~~ for details). We investigated their out-of-sample associations with ecological indicators of mental health (sleep duration, time spent with physical exercise, number of alcoholic beverages and cigarettes consumed). To tease apart complementary and redundant effects, we constructed multiple linear regression models on out-of-sample predictions combining all three proxy measures **(A)**. For comparison, we repeated the analysis using the actual target measures **(B)** observed on the held-out data. Regression models are depicted rows-wise. Box plots summarize the uncertainty distribution of target-specific (color) regression coefficients with whiskers indicating two-sided 95% uncertainty intervals (parametric bootstrap). Dots illustrate a random subset of 200 out of 10 000 coefficient draws. The average coefficient estimate is annotated for convenience. At least two distinct patterns emerged: either the health outcome was specifically associated with one proxy measures (brain age delta and number of alcoholic beverages) or multiple measures showed additive associations with the outcome (*e.g.* number of pack years smoked). ~~Finally,~~ For target measures **(B)**~~show noisier~~, associations ~~than proxy~~ with health habits were often noisier or less pronounced compared to the target measures **(A)** ~~, though none of the significant associations changed~~ and even a a change in direction was observed for brain age and metabolic activity. Figure 2 – Figure supplement 1 shows highly similar trends with marginal associations between proxy measures and health-related habits. Our results suggest that the proxy measures capture well health-related habits, potentially better than the original target measures, and in a complementary way across the three measures. The same patterns emerged as brain-predicted age rather than the brain age delta is used as a proxy measure (Figure 2 – Figure supplement 2). As proxy-specific deconfounding is applied, this pattern is preserved (Figure 2 – Figure supplement 3). Modeling of health-related habits jointly from proxy and target measures simultaneously revealed specific complementari. ty between proxy and target measures across multiple domains *i.e.* age, fluid intelligence, neuroticism (Figure 2 – Figure supplement 4).

The relative importance of brain and sociodemographic data depends on the target

In a second step, we investigated the relative performance of proxy measures built from brain signals and distinct sociodemographic factors for the three targets: age, fluid intelligence and neuroticism. Among the sociodemographic variables there was one block for each target explaining most of the prediction performance (Figure 3, dotted outlines). Combining all sociodemographic variables did not lead to obvious enhancements (Figure 3 – Figure supplement 2). For age prediction, variables related to current life-style showed by far the highest performance. For fluid intelligence, education performed by far best. For neuroticism, mood & sentiment clearly showed the strongest performance.

Combining MRI and sociodemographics, enhanced age prediction systematically on all four blocks of variables (Figure 3 solid outlines, and Table S1). The benefit of brain-imaging features was less marked for prediction of fluid intelligence or neuroticism. With fluid intelligence, brain-imaging data improved the performance statistically significantly for all models, yet, with small effect sizes (Table S1). For neuroticism, no systematic benefit of including brain images alongside sociodemographics emerged (Table S1, bottom row). Nevertheless, brain data was sufficient for statistically significant approximation of the target measures in all three targets (Table S5).

Psychological measures often come without physical scales and units [50]. In practice, clinicians and educators use them with specific thresholds for decision making. To investigate empirically-defined proxy measures beyond continuous regression, we performed binary classification of extreme groups obtained from discretizing the targets using the 33_{rd} and 66_{th} percentiles, following the recommendations by Gelman and Hill 2006 regarding discrete variable encoding strategies. Furthermore, we measured accuracy with the ~~AUC~~ area under the classification accuracy curve (AUC) which is only sensitive to ranking, ignoring the scale of the error. Classification performance visibly exceeded the chance level (AUC > 0.5) for all models (Figure 4) and approached or exceeded levels considered practically useful (AUC > 0.8) [49]. Across proxy measures, models including sociodemographics performed best but the difference between purely sociodemographic and brain-based models was comparably weak, at the order of 0.01-0.02 AUC points (Table S2). Using brain data only led to worse performance, yet, still better than chance as revealed by permutation testing (Table S6).

Discussion

Guided by machine learning, we empirically derived proxy measures that combine multiple sources of information to capture extensively validated target measures from psychology. These proxy measures all showed complementary associations with real-world health indicators beyond the original targets. The combination of brain imaging and target-specific sociodemographic inputs often improved approximation performance.

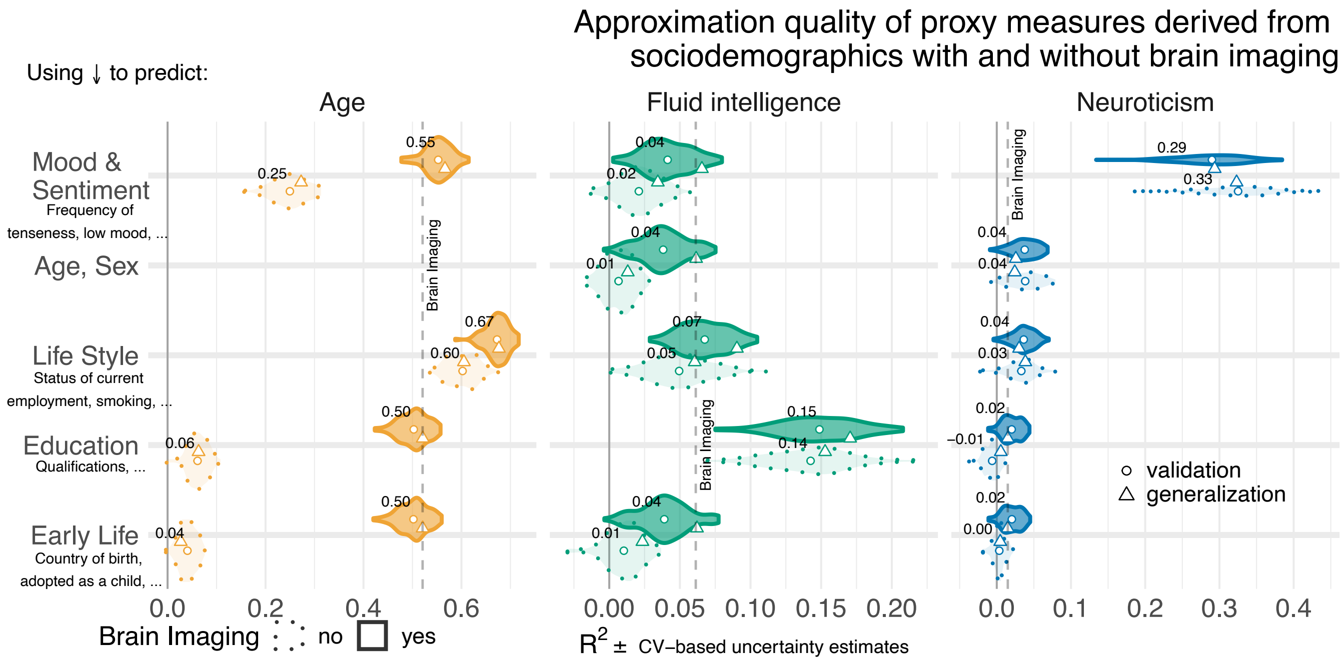

Figure 3. Approximation performance of proxy measures derived from sociodemographic data and MRI. We report the R^2 metric to facilitate comparisons across prediction targets. The cross-validation (CV) distribution (100 Monte Carlo splits) on the validation dataset is depicted by violins. Drawing style indicates whether brain imaging (solid outlines of violins) was included in addition or not (dotted outlines of violins). Dots depict the average performance on the validation data across CV-splits. Pyramids depict the performance of the average prediction (CV-bagging) on held-out generalization datasets. For convenience, the mean performance on the validation set is annotated for each plot. Vertical dotted lines indicate the average performance of the full MRI model. The validation and held-out datasets gave similar picture of approximation performance with no evidence for cross-validation bias [52]. For the averaged out-of-sample predictions, the probability of the observed performance under the null-distribution and the uncertainty of effect sizes were formally probed using permutation tests and bootstrap-based confidence intervals (Table S1). Corresponding statistics for the baseline performance of models solely based on brain imaging (vertical dotted lines) are presented in Table S5. Figure 3 – Figure supplement 1 shows approximation results based on MRI. Figure 3 – Figure supplement 2 presents results based on all sociodemographic factors.

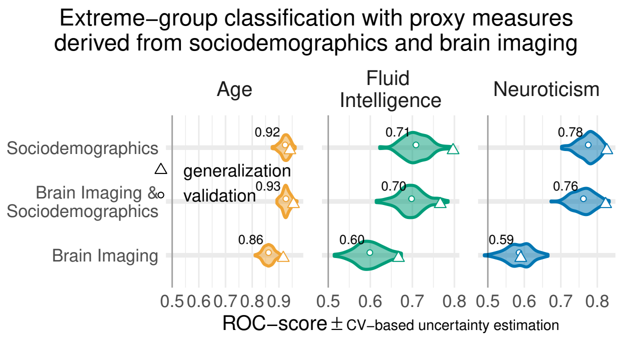

Figure 4. Classification analysis from imaging, sociodemographics and combination of both data. For classification of extreme groups instead of continuous regression, we split the data into low vs high groups based on 33_{rd} and 66_{th} percentiles. Visual conventions follow Figure 3. We report the accuracy in AUC. Models including sociodemographics performed visibly better than models purely based on brain imaging. Differences between brain-imaging and sociodemographics appeared less pronounced as compared to the fully-fledged regression analysis. For the average out-of-sample predictions, the probability of the observed performance under the null-distribution and the uncertainty of effect sizes were formally probed using permutation tests and bootstrap-based confidence intervals (Table S2). Corresponding statistics for the baseline performance of models solely based on brain imaging (vertical dotted lines) are presented in Table S6. Overall, when moving from the more difficult full-scale regression problem to extreme-group classification problem with purely ranking-based scores, the relative differences between brain-based and sociodemographics-based prediction gradually faded away.

Empirically-derived proxy measures: validity and practical utility

In our study, construct validity [6, 54, 7] of the corresponding proxy measures was supported by the gain in prediction performance brought by specific sociodemographic factors (Figure 3). Association with health-relevant habits added external validity to the proxy measures (Figure 2). The complementary patterns related to traditional construct semantics: High consumption of cigarettes is associated with neuroticism [55], excessive drinking may lead to brain atrophy and cognitive decline [56] – both common correlates of elevated brain age [22, 57].

Can our empirically-derived proxy measures, thus, substitute for specific psychometric instruments? A mental-health professional may still prefer an established routine for clinical assessment, relying on interviews and personality questionnaires ~~while still applying~~ with implicit experience-based thresholds. Inclusion of brain imaging may even seem to yield diminishing returns when approximating high-level psychological traits. Yet, it could simply be a matter of time until more effective acquisition protocols will be discovered alongside useful signal representations. Including brain imaging, rather seems a "safe bet" as machine learning is often capable of selecting relevant inputs [11, 58] and costs of MRI-acquisition can be amortized by clinical usage. Empirically-derived proxy measures may open new doors where tailored assessment of latent constructs is not applicable due to lack of specialized mental-health workforce or sheer cost.

Constructs of mental-health can be accessed from general-purpose data

Brain age has served as landmark in this study. It has been arguably the most discussed candidate for a surrogate biomarker in the brain imaging literature [16, 17, 24]. With mean absolute errors around 4 years, up to 67% variance explained, and AUC-scores up to 0.93 in the classification setting, our results compare favorably to the recent brain-age literature within the UK Biobank [19, 59] and in other datasets [22, 11], though we relied on ~~classical~~ off-the-shelf methods and not ~~deep learning~~custom deep learning methods [60]. Applying the same approach to psychological constructs (fluid intelligence, neuroticism), we found that approximation from brain imaging data or sociodemographic descriptors was generally harder.

It is important to recapitulate that approximation quality on these differently measured targets has a different meaning. Age is measured with meaningful physical units (years) on a ratio scale [50] (Selma is *twice as old* as Bob). Psychometric scores are unit-free, which may provoke ambiguity regarding the level of measurement [54]. Their implied scales may be considered as interval (the *difference between* Bob's and Selma's intelligence is -0.1 standard deviations) if not ordinal (Bob's intelligence was *ranked below* Selma's) [50]. In day-to-day psychological practice, these scores are often used via practically-defined thresholds, *e.g.* school admission or pilot candidate selection [61, 62]. In the classification setting, all proxy measures approached or exceeded a performance of 0.80 deemed relevant in biomarker development [49], though to be fair, they approximated established psychometric targets (proxy measures themselves) and not a medical condition. Different proxy measures should, thus, be subjected to different standards, depending on the granularity of the implied measurement scale.

~~The out-of-sample associations of the approximated constructs with health-related habits~~A more complete view on how the proxy measures capture mental-health constructs emerges from their associations with real-world behavior (Figure 2)~~paint a more complete picture~~. ~~Compared to the traditional measures~~ Indeed, the associations with proxy measures (Figure 2 **B**) ~~, the associations between proxy measures and real-world behavior~~ were less noisy and more consistent then with the target measures (Figure 2 **A**), regardless of their approximation quality ~~(A)~~. This may seem surprising at first, but the target measures are themselves noisy and of imperfect validity. ~~Our target~~ These measures correspond to traditional tests which, in practice, must be interpreted by an expert, actively confronting their output with broader information on the individual. For instance, IQ scores are typically normalized across age groups. However, extending such a normalization approach to many factors (socio-economic status, culture, gender) poses fundamental high-dimensional statistics challenges. Conversely, ~~the proxy measures were assembled via~~ using machine learning to assemble proxy measures by mapping the targets to rich sociodemographic and brain data ~~, implicitly contextualizing themand building a less noisy measure in the process~~implicitly contextualizes them. In this ~~sense,~~ respect, the resulting measures capture more general signal than the original tests. Here, machine learning could be seen as mimicking the work of a mental health expert who carefully compares psychometric results with other facts known about an individual and its reference population.

The benefits offered by brain data depend on the target construct

All brain-derived approximations were statistically meaningful. Yet, only for age prediction, imaging data by itself led to convincing performance. For fluid intelligence and neuroticism, sociodemographic factors were the most important determinants of prediction success. The best-performing sociodemographic models were based on inputs semantically close to these targets, *i.e.*, education details or mood & sentiment. While those results support construct validity, they may come with a certain risk of circularity. The causal role of those predictors is not necessarily clear as better educational attainment is heritable itself [63] and may reinforce existing cognitive abilities. Similarly, prolonged emotional stress due to life events may exacerbate existing dispositions to experience negative emotions captured by neuroticism [64], traits which commonly help accumulate stressful life events [38]. Nevertheless, for fluid intelligence but not neuroticism, brain imaging added incremental value when combined with various sociodemographic predictors. This may suggest that the cues for neuroticism conveyed by brain imaging were already present in sociodemographic predictors, hinting at common causes. Off note, in the specific context of aging, the empirical distinction between brain age and cognitive age is reflecting a similar intuition [65].

Limitations

Additional constructs and psychometric tools could have been visited. The broader construct of intelligence is often estimated using a general factor model with multiple correlated tests. While this is obviously useful for normative assessments, measures of fluid intelligence can also serve a situational fitness signal [30]. There is a wealth of questionnaires for measuring negative emotionality and neuroticism, specifically. Yet, we could only study the EPQ scale provided by the UK Biobank. A complementary approach would be to estimate latent factors by pooling all non-imaging data semantically related to neuroticism [66]. Here, we considered established target measures "as is", instead of derivatives.

It terms of mental-health research, this study falls short of directly testing the clinical relevance of estimated proxy measures. Even in a very large general-population cohort such as the UK Biobank, there are only a few hundred diagnosed cases of mental disorders (ICD-10 mental-health diagnoses from the F chapter) with brain-imaging data available. ~~This challenge~~ As a result, we could not directly assess the performance of proxy measures in clinical populations. The low number of diagnosed mental disorders in UK Biobank highlights the practical importance of studying mental health as a continuous, in addition to diagnosed conditions. Indeed, a public health perspective calls for targeting individual differences in health, not only pathology. Psychological constructs such as IQ and neuroticsm are important factors of the epidemiology of psychiatric disorders [38, 30, 29, 67], and accelerated brain aging is associated with various neurological conditions [18, 17, 25]. Yet, few cohorts come with extensive neuropsychological testing. Validated proxies of these constructs open the door to including them in epidemiological studies as secondary outcomes or additional explanatory variables.

Conclusion: Proxy measures may enhance the validity of constructs gauging mental health

~~Empirical proxy measures of brain and mental health~~In population studies of mental health, individual traits are captured via lengthy assessments, tailored to specific brain and psychological constructs. We have shown that proxy measures built empirically from general-purpose data can capture these constructs and can improve upon traditional measures

when studying real-world health patterns. Understanding and improving mental health of populations call for studying not only patients but also the continuity of psychological traits in the general population. In addition, Proxy measures can make psychological constructs available to broader, more ecological studies building on large epidemiological cohorts or real-world evidenceis increasingly seen as . This can make the difference where psychological constructs are central to developing treatment and prevention strategies [68] . This is particularly important in mental health, whose proper assessments demands considering the full context of individuals. We believe that developing proxy measures for constructs that are difficult to assess brings promises to this mental health research agenda: Proxy measures can provide enriched outcomes facilitating the study of mental health and its underlying factors in a broader and more ecological way, but direct measures have not been collected.

Methods

To facilitate reproduction, understanding, and reuse, we have made all data analysis and visualization source code available on Github: https://github.com/KamalakerDadi/empirical_proxy_measures.

To facilitate reproduction, understanding, and reuse, we have made all data analysis and visualization source code available on Github: .

Dataset

The United Kingdom Biobank (UKBB) database is to date the most extensive large-scale cohort aimed at studying the determinants of the health outcomes in the general adult population. The UKBB is openly accessible and has extensive data acquired on 500 000 individuals aged 40-70 years covering rich phenotypes, health-related information, brain-imaging and genetic data [12]. Participants were invited for repeated assessments, some of which included MR imaging. For instance, cognitive tests that were administered during an initial assessment were also assessed during the follow-up visits. This has enabled finding for many subjects at least one visit containing all heterogeneous input data needed to develop the proposed proxy measures. The study was conducted using the UKBB Resource Application 23827.

Participants

All participants gave informed consent. The UKBB study was examined and approved by the North West Multi-centre Research Ethics Committee. We considered participants who have responded to cognitive tests, questionnaires, and have access to their primary demographics and brain images [69]. Out of the total size of UKBB populations, we found 11 175 participants who had repeated assessments overlapping with the first brain imaging release [70]. Note that the features (sociodemographic variables) that we included in the analysis are measures that are self-reported during a follow-up imaging visit. The demographics are 51.6% female (5 572) and 48.3% male (5 403), and an age range between 40-70 years (with a mean of 55 years and standard deviation of 7.5 years). Out of the complete analysis set, The data for model training were selected using a randomized split-half procedure yielding 5 587 individualswere used in the study to train the model and . The remaining subjects were set aside as a held-out set for generalization testing (see section). Model development and generalization testing). We made sure that the subjects used for model training and generalization testing were strictly non-overlapping.

Learning curves documented that the training split was sufficiently large for constructing stable prediction models Figure 1 – Figure supplement 1 with profiles of performance similar to latest benchmarks on model complexity in the UK Biobank [71]. Moreover, simulations and empirical findings suggest that larger testing sets are more effective at mitigating optimistic performance estimates [72, 52]. Together, this provided a pragmatic solution to the inference-prediction dilemma [58, 73] given the two objectives of the present investigation to obtain reasonably good predictive models, while at the same time performing parameter inference of statistical models developed on the left-out data.

To establish specific comparisons between models based on sociodemographics, brain data or their combinations we exclusively considered the cases for which MRI scans were available. The final sample sizes used for model construction and generalization testing then depended on the availability of MRI: For age and fluid intelligence, our random splitting procedure (randomized split-half procedure (see section Model development and generalization testing) yielded 4203 cases for model building and 4157 for generalization. For cases with valid neuroticism assessment, fewer brain images were available, which yielded 3550 cases for model building and 3509 for generalization.

Data acquisition

Sociodemographic data (non-imaging) was collected with self-report measures administered through touchscreen questionnaires, complemented by verbal interviews, physical measures, biological sampling and imaging data. MRI data were acquired with the Siemens Skyra 3T using a standard Siemens 32-channel RF receiver head coil [74]. We considered three MR imaging modalities as each of them potentially captures unique neurobiological details: structural MRI (sMRI/T1), resting-state functional MRI (rs-fMRI) and diffusion MRI (dMRI). For technical details about the MR acquisition parameters, please refer to [70]. We used image-derived phenotypes (IDPs) of those distinct brain-imaging modalities, as they provide actionable summaries of the brain measurements and encourage comparability across studies.

Target measures

As our target measures for brain age modeling, we use an individual's age at baseline recruitment (UKBB code "21022-0.0"). Fluid intelligence, was assessed using a cognitive battery designed to measure an individual's capacity to solve novel problems that require logic and abstract reasoning. In the UK Biobank, the fluid intelligence test (UKBB code "20016-2.0") comprises thirteen logic and reasoning questions that were administered via the touchscreen to record a response within two minutes for each question. Therefore, each correct answer is scored as one point with 13 points in total[1]. Neuroticism (UKBB code "20127-0.0") was measured using a shorter version of the revised Eysenck Personality Questionnaire (EPQ-N) comprised of 12-items [32]. Neuroticism was assessed during Biobank's baseline visit. The summary of the individual's scores ranges from 0 to 12 that assess dispositional tendency to experience negative emotions [2].

1 A complete overview of the 13 individual fluid intelligence items can be seen from this manual https://biobank.ctsu.ox.ac.uk/crystal/crystal/docs/Fluidintelligence.pdf

2 For a complete list of Neuroticism questionnaires can be seen from this manual https://biobank.ctsu.ox.ac.uk/crystal/crystal/docs/MentalStatesDerivation.pdf

In the course of this work, a question that emerged concerned the size of the gap between age at baseline recruitment and MRI-scan time and its potential impact on the analysis. Supplementary checks indicated that the age gap was at least 5 years for most participants. Yet, from a statistical perspective, the two age measures turned out highly interchangeable (Figure S2) and global conclusions remained unchanged (Figure S3).

Sociodemographic data

In this work, we refer to non-imaging variables broadly as sociodemographics excluding the candidate targets fluid intelligence and neuroticism. To approximate latent constructs from sociodemographics, we included 86 non-imaging inputs (Table S7) which are the collection of variables reflecting each participant's demographic and social factors *i.e.*, sex, age, date and month of birth, body mass index, ethnicity, exposures at early life –*e.g.* breast feeding, maternal smoking around birth, adopted as a child– education, lifestyle-related variables –*e.g.* occupation, household family income, household people living at the same place, smoking habits–, and mental-health variables. All these data were self-reported. We then assigned these 86 variables to five groups based on their relationships. Based on our conceptual understanding of the variables, we name assigned them to one out of five groups: **1)** mood & sentiment, **2)** primary demographics as age, sex, **3)** lifestyle, **4)** education, **5)** early life. We then investigated the intercorrelation between all 86 variables to ensure that the proposed grouping is compatible with their empirical correlation structure Figure S1.

The sociodemographic groups had varying amounts of missing data. For *e.g.* the source of missingness is concerned with the participants lifestyle habits such as smoking and mental health issues [75]. To deal with this missingness in the data using imputation [76], we used column-wise replacement of missing information with the median value calculated from the known part of the variable. We subsequently included an indicator for the presence of imputed for down-stream analysis. Such imputation is well suited to predictive models [77].

Image processing to derive phenotypes for machine learning

MRI data preprocessing were carried out by UKBB imaging team. The full technical details are described elsewhere [70, 74]. Below, we describe briefly the custom processing steps that we used on top of the already preprocessed inputs.

Structural MRI

This type of data analysis on T1-weighted brain images are concerned with morphometry of the gray matter areas *i.e.* the quantification of size, volume of brain structures and tissue types and their variations under neuropathologies or behavior [78]. For example, volume changes in gray matter areas over lifetime are associated with: brain aging [79], general intelligence [80] and brain disease [81]. Such volumes are calculated within pre-defined ROIs composed of cortical and sub-cortical structures [82] and cerebellar regions [83]. We included 157 sMRI features consisting of volume of total brain and grey matter along with brain subcortical structures[3]. All these features are pre-extracted by UKBB brain imaging team [70] and are part of data download. We concatenated all inputs alongside custom-built fMRI features for predictive analysis (feature union).

Diffusion weighted MRI

Diffusion MRI enables to identify white matter tracts along principal diffusive direction of water molecules, as well as the connections between different gray matter areas [84, 85]. The study of these local anatomical connections through white matter are relevant to the understanding of neuropathologies and functional organization [86]. We included 432 dMRI skeleton features of FA (fractional anisotropy), MO (tensor mode) and MD (mean diffusivity), ICVF (intra-cellular volume fraction), ISOVF (isotropic volume fraction) and OD (orientation dispersion index) modeled on many brain white matter structures extracted from neuroanatomy[4]. For extensive technical details, please refer to [87]. The skeleton features we included were from category134 shipped by the UKBB brain-imaging team and we used them without modification.

Functional MRI

Resting-state functional MR images capture low-frequency fluctuations in blood oxygenation that can reveal ongoing neuronal interactions in time forming distinct brain networks [88]. Functional connectivity within these brain network can be linked to clinical status [89], to behavior [70], or to psychological traits [44]. We also included resting-state connectivity features based on the time-series extracted from Independent Component Analysis (ICA) with 55 components representing various brain networks extracted on UKBB rfMRI data [70]. These included the default mode network, extended default mode network and cingulo-opercular network, executive control and attention network, visual network, and sensorimotor network. We measured functional connectivity in terms of the between-network covariance. We estimated the covariance matrices using Ledoit-Wolf shrinkage [90]. To account for the fact that covariance matrices live on a particular manifold, *i.e.*, a curved non-Euclidean space, we used the tangent-space embedding to transform the matrices into a Euclidean space [91, 92] following recent recommendations [93, 94]. For predictive modeling, we then vectorized the covariance matrices to 1 485 features by taking the lower triangular part. These steps were performed with `NiLearn` [95].

Comparing predictive models to approximate target measures

Imaging-based models

First, we focused on purely imaging-based models based on exhaustive combinations of the three types of MRI modalities (see Table 1 for an overview). This allowed us to study potential overlap and complementarity between the MRI-modalities. Preliminary analyses revealed that combining all MRI data gave reasonable results with no evident disadvantage over particular combinations of MRI modalities (Figure 3 – Figure supplement 1), hence, for simplicity, we only focused on the full MRI model in subsequent analyses.

Sociodemographic models

We composed predictive models based on non-exhaustive combinations of different types of sociodemographic variables. To investigate the relative importance of each class of sociodemographic inputs, we performed systematic model comparisons.

3 Regional grey matter volumes `http://biobank.ctsu.ox.ac.uk/crystal/label.cgi?id=1101` Subcortical volumes `http://biobank.ctsu.ox.ac.uk/crystal/label.cgi?id=1102`

4 Diffusion-MRI skeleton measurements `http://biobank.ctsu.ox.ac.uk/crystal/label.cgi?id=134`

Table 1. Imaging-based models.

Index	Name	# variables	# groups
1	brain volumes (sMRI)	157	1
2	white matter (dMRI)	432	1
3	functional connectivity (fMRI)	1485	1
4	sMRI, dMRI	589	2
5	sMRI, fMRI	1642	2
6	dMRI, fMRI	1917	2
7	sMRI, dMRI, fMRI (full MRI)	2074	3

Table 2. Non-imaging baseline models or sociodemographic models based on single group. Variables in each group are described at corresponding section: .sociodemographic data

Index	Name	# variables
1	Mood & Sentiment (MS)	25
2	Age, Sex (AS)	5
3	Life style (LS)	45
4	Education (EDU)	2
5	Early Life (EL)	9

Table 3. Random forest hyperparameters and tuning with grid search (5 fold cross-validation).

Hyperparameter	Values
Impurity criterion	Mean squared error
Maximum tree depth	5, 10, 20, 40, full depth
Fraction of features for split	1, 5, "log2", "sqrt", "complete"
Number of trees	250

We were particularly interested in studying the relative contributions of early-life factors as compared to factors related to more recent life events such as education as well as factors related to current circumstances such as mood & sentiment and life-style. The resulting models based on distinct groups of predictors are listed in Table 2 (for additional details see Table S7 and Figure S1).

Combined imaging and sociodemographic models

In the next step, we were interested in how brain-related information would interact within each of these sociodemographic models. For example, information such as the age of an individual, or the level of education, may add important contextual information to brain images. We therefore considered an alternative variant for each of the models in Table 2 that included all MRI-related features (2 074 additional features) as described at section image processing to derive phenotypes for machine learning.

Predictive model

Linear models are recommended as default choice in neuroimaging research [93, 96] especially when datasets include fewer than 1000 data points. In this study approximated targets generated by distinct underlying mechanisms based on multiple classes of heterogenous input data with several thousands of data points. We hence chose the non-parametric random forest algorithm that can be readily applied on data of different units for non-linear regression and classification [97] with mean squared error as impurity criterion. To improve computation time we fixed tree-depth to 250 trees, a hyper-parameter that is not usually not tuned but set to a generous number as performance plateaus beyond a certain number of trees [98, ch. 15]. Preliminary analyses suggested that additional trees would not have led to substantial improvements in performance. We used nested cross-validation (5-fold grid search) to tune the depth of the trees as well as the number of variables considered for splitting (see Table 3 for a full list of hyper-parameters considered).

Table 4. Number of samples for classification analysis (N).

# groups	Age	Fluid intelligence	Neuroticism
1	1335	1108	1054
2	1200	898	1020

Classification analysis. We also performed classification analysis on the continuous targets. ~~For this purpose, we discretized the targets into~~ Adapting recommendations from Gelman and Hill [53], we performed discrete variable encoding of the targets leading to extreme groups based on the 33_{rd} and 66_{th} percentiles (see Table 4 for the number of classification samples per group). This choice avoids including samples near the average outcome for which the input data may be indistinct. We were particularly interested in understanding whether model performance would increase when moving toward classifying extreme groups. For this analysis, we considered all three types of models (full MRI 2074 features from imaging-based models~~see section~~, all sociodemographics variables, total 86 variables see section~~)~~, combination of full MRI and all sociodemographics, a total 2160 variables see section (See section Comparing predictive models to approximate target measures). When predicting age, we excluded the age & sex sociodemographic block from all sociodemographic variables which then yielded a total of 81 variables. To assess the performance for classification analysis, we used the area under the curve (AUC) of the receiver operator characteristic (ROC) as an evaluation metric [96].

Model development and generalization testing

Before any empirical work, we generated two random partitions of the data, one validation dataset for model construction and one held-out generalization dataset for studying out-of-sample associations using classical statistical analyses.

For cross-validation, we then subdivided the validation set into 100 training- and testing splits following the Monte Carlo resampling scheme (also referred to as shuffle-split) with 10% of the data used for testing. To compare model performances based on paired tests, we used the same splits across all models. Split-wise testing performance was extracted and carried forward for informal inference using violin plots (Figure 3,Figure 4). For generalization testing, predictions on the held-out data were generated from all 100 models from each cross-validation split.

On the held-out set, unique subject-wise predictions were obtained by averaging across folds and occasional duplicate predictions due to Monte Carlo sampling which could produce multiple predictions per subject[5]. Such strategy is known as CV-bagging ~~[99]~~ [99, 100] and can improve both performance and stability of results[6]. The resulting averages were reported as point estimates in Figures 3,4, and 3 – Figure supplement 1 and used as proxy measures in the analysis of health-related behaviors Figure 2.

5 We ensured prior to computation that with 100 CV-splits, predictions were available for all subjects.

6 The use of CV-bagging can explain why on figures 3,4, and 3 – Figure supplement 1 the performance was sometimes slightly better on the held-out set compared to the cross-validation on the validation test.

Table 5. Extra health variables used for correlation analysis with subject-specific predicted scores.

Family	eid	Variables
Alcohol*	1568-0.0	Average weekly red wine intake
	1578-0.0	Average weekly champagne plus white wine intake
	1588-0.0	Average weekly beer plus cider intake
	1598-0.0	Average weekly spirits intake
	1608-0.0	Average weekly fortified wine intake
	5364-0.0	Average weekly intake of other alcoholic drinks
Physical activity	22040-0.0	Summed MET minutes per week for all activity
Smoking	20161-0.0	Pack years of smoking
Sleep	1160-0.0	Sleep duration

*We computed a compound drinking score by summing up all variables from the alcohol family

Statistical analysis

Resampling statistics for model comparisons on the held-out data

To assess the statistical significance of the observed model performance and the differences in performance between the models, we computed resampling statistics of the performance metrics on the held-out generalization data not used for model construction [101]. Once unique subject-wise predictions were obtained on the held-out generalization data by averaging the predictions emanating from each fold of the validation set (cv-bagging), we computed null- and bootstrap-distributions of the observed test statistic on the held-out data, i.e., R^2 score for regression and *AUC* score for classification.

Baseline comparisons. To obtain a p-value for baseline comparisons (*could the prediction performance of a given model be explained by chance?*) on the held-out data, we permuted targets 10 000 times and then recomputed the test statistic in each iteration. P-values were then defined as the probability of the test statistic under null distribution being larger than the observed test statistic. To compute uncertainty intervals, we used bootstrap, recomputing the test statistic after resampling 10 000 times with replacement and reporting the 2.5 and 97.5 percentiles of the resulting distribution.

Pairwise comparisons between models. For model comparisons, we considered the out-of-sample difference in R^2 or *AUC* between any two models. To obtain a p-value for model comparisons (*could the difference in prediction performance between two given models be explained chance?*) on the held-out data, we permuted the scores predicted by model A and model B for every single prediction 10 000 times and then recomputed the test statistic in each iteration. We omitted all cases for which only predictions from one of the models under comparison was present. P-values were then defined as the probability of the absolute of the test statistic under null distribution being larger than the absolute observed test statistic. The absolute was considered to account for differences in both directions. Uncertainty intervals were obtained from computing the 2.5 and 97.5 percentiles of the bootstrap distribution based on 10 000 iterations. Here, predictions from model A and model B were resampled using identical resampling indices to ensure a meaningful paired difference.

Out-of-sample association between proxy measures and health-related habits

Computation of brain age delta and de-confounding. For association with health-contributing habits (Table 5), we computed the brain age delta as the difference between predicted age and actual age:

$$BrainAge\Delta = Age_{predicted} - Age \quad (1)$$

As age prediction is rarely perfect, the residuals will still contain age-related variance which commonly leads to brain age bias when relating the brain age to an outcome of interest, *e.g.*, sleep duration [102]. To mitigate leakage of age-related information into the statistical models, we employed a de-confounding procedure in line with [103] and [11, eqs. 6-8] consisting in residualizing a measure of interest (*e.g.* sleep duration) with regard to age through multiple regression with quadratic terms for age. To minimize computation on the held-out data, we first trained a model relating the score of interest to age on the validation set to then derive a de-confounding predictor for the held-out generalization data. The resulting de-confounding procedure for variables in the held-out data amounts to computing an age-residualized predictor $measure_{resid}$ from the measure of interest (*e.g.* sleep duration) by applying the following quadratic fit on the validation data:

$$\begin{aligned} measure_{validation} = {} & age_{validation} \times \beta_{val1} + \\ & age^2_{validation} \times \beta_{val2} + \epsilon \end{aligned} \quad (2)$$

The de-confounding predictor was then obtained by evaluating the weights β_{val1} and β_{val2} obtained from Equation 2 on the generalization data:

$$\begin{aligned} deconfounder = {} & age_{generalization} \times \beta_{val1} \\ & + age^2_{generalization} \times \beta_{val2} \end{aligned} \quad (3)$$

We performed this procedure for all target measures, to study associations not driven by the effect of age. For supplementary analyses presented in figure Figure 2 – Figure supplement 3, the same procedure was applied, substituting age for fluid intelligence and neuroticism, respectively.

Health-related habits regression. We then investigated the joint association between proxy measures of interest and health-related habits (Table 5) using multiple linear regression. For simplicity, we combined all brain imaging and all sociodemographics variables (Figure 3, Figure 3 – Figure supplement 1, Figure 3 – Figure supplement 2). The ensuing model can be denoted as

$$\begin{aligned} measure = {} & deconfounder \times \beta_1 + BrainAge\Delta \times \beta_2 \\ & + PredFluidInt \times \beta_3 + PredNeurot \times \beta_4 + \epsilon, \end{aligned} \quad (4)$$

where ~~$outcome_{resid}$~~ *deconfounder* is given by Equation 2. Prior to model fitting, rows with missing inputs were omitted. For comparability, we then applied standard scaling on all outcomes and all predictors.

The parametric bootstrap was a natural choice for uncertainty estimation, as we used standard multiple linear regression which provides a well defined procedure for mathematically quantifying its implied probabilistic model. Computation was carried out using `sim` function from the `arm` package as de-

scribed in [53, Ch.7,pp.142–143]. This procedure can be intuitively regarded as yielding draws from the posterior distribution of the multiple linear regression model under the assumption of a uniform prior. For consistency with previous analyses, we computed 10000 draws.

For supplementary analysis in Figure 2 – Figure supplement 2, the brain-predicted age instead of the delta was used:

$$measure = deconfounder \times \beta_1 + BrainAge \times \beta_2 + PredFluidInt \times \beta_3 + PredNeurot \times \beta_4 + \epsilon, \quad (5)$$

For supplementary analysis in Figure 2 – Figure supplement 3, additional deconfounders were introduced.

$$measure = deconfounder_{age} \times \beta_1 + BrainAge \times \beta_2 + deconfounder_{FI} \times beta_3 + PredFluidInt \times \beta_4 + deconfounder_N + \beta_5 + PredNeurot \times \beta_6 + \epsilon, \quad (6)$$

where $deconfounder_{FI}$ is the deconfounder for fluid intelligence and $deconfounder_N$ the deconfounder for neuroticsm following the procedure described in Equation 2 and Equation 3.

For supplementary analysis in Figure 2 – Figure supplement 4, proxies and targets were analyzed simultaneously.

$$measure = Age \times \beta_1 + BrainAge \times \beta_2 + FluidIntelligence \times beta_3 + PredFluidInt \times \beta_4 + Neuroticism + \beta_5 + PredNeurot \times \beta_6 + \epsilon, \quad (7)$$

Software

Preprocessing and model building were carried out using Python 3.7. The `NiLearn` library was used for processing MRI inputs [95]. We used the *scikit-learn* library for machine learning [104]. For statistical modeling and visualization we used the R-language [105] (version 3.5.3) and its ecosystem: `data.table` for high-performance manipulation of tabular data, `ggplot` [106, 107] for visualization and the `arm` package for parametric bootstrapping [108]. All data analysis code is shared on GitHub: https://github.com/KamalakerDadi/empirical_proxy_measures.

Availability of source code and requirements

- Project name: "empirical_proxy_measures"
- Project home page: e.g. https://github.com/KamalakerDadi/empirical_proxy_measures
- Operating system(s): e.g. Platform independent
- Programming language: e.g. Python and R
- Other requirements: e.g. Python 3.6.8 or higher, R 3.4.3 or higher
- License: BSD-3

Availability of supporting data and materials

The data supporting the results and figures of this article is available in the "empirical_proxy_measures" repository, https://github.com/KamalakerDadi/empirical_proxy_measures. The input data is publicly available via the UK Biobank http://www.ukbiobank.ac.uk.

Declarations

Author's Contributions (alphabetic order)

- **Conceptualization**: BT, DB, DE, GV, JH
- **Data curation**: DB, KD
- **Software**: BT, DE, GV, KD
- **Formal analysis**: DE, GV, KD
- **Supervision**: BT, DE, GV
- **Funding acquisition**: GV, JH
- **Validation**: DE, KD
- **Investigation**: DE, KD
- **Visualization**: DE, GV, KD
- **Methodology**: BT, DE, GV
- **Project administration**: DE, GV
- **Writing – original draft**: DE, KD
- **Writing – review and editing**: DB, BT, DE, GV, JH, KD

Acknowledgements

We would like to thank Dr. Stefania de Vito and Dr. Benjamin de Haas for the critical review and helpful discussion of previous versions of the manuscript. We would like to thank Dr. Julien Dubois and Prof. Ralph Adolphs for helpful discussions in the course of this research project.

References

1. Kapur S, Phillips AG, Insel TR. Why has it taken so long for biological psychiatry to develop clinical tests and what to do about it? Molecular Psychiatry 2012;17(12):1174–1179.
2. Insel T, Cuthbert B, Garvey M, Heinssen R, Pine DS, Quinn K, et al. Research Domain Criteria (RDoC): Toward a New Classification Framework for Research on Mental Disorders. American Journal of Psychiatry 2010;167(7):748–751.
3. Szucs D, Ioannidis JP. Empirical assessment of published effect sizes and power in the recent cognitive neuroscience and psychology literature. PLoS biology 2017;15(3):e2000797.
4. Hozer F, Houenou J. Can neuroimaging disentangle bipolar disorder? Journal of affective disorders 2016;195:199–214.
5. Enkavi AZ, Eisenberg IW, Bissett PG, Mazza GL, MacKinnon DP, Marsch LA, et al. Large-scale analysis of test–retest reliabilities of self-regulation measures. Proceedings of the National Academy of Sciences 2019;116(12):5472–5477.
6. Cronbach LJ, Meehl PE. Construct validity in psychological tests. Psychological Bulletin 1955;52(4):281–302.
7. Borsboom D, Mellenbergh GJ, van Heerden J. The Concept of Validity. Psychological Review 2004;111(4):1061–1071.
8. Eisenberg IW, Bissett PG, Enkavi AZ, Li J, MacKinnon DP, Marsch LA, et al. Uncovering the structure of self-regulation through data-driven ontology discovery. Nature Communications 2019;10(1):1–13.
9. Nave G, Jung WH, Linnér RK, Kable JW, Koellinger PD. Are Bigger Brains Smarter? Evidence From a Large-Scale Preregistered Study:. Psychological Science 2018;.
10. Kievit RA, Fuhrmann D, Borgeest GS, Simpson-Kent IL, Henson RNA. The neural determinants of age-related changes in fluid intelligence: a pre-registered, longitudinal analysis in UK Biobank. Wellcome Open Research 2018;3.
11. Engemann DA, Kozynets O, Sabbagh D, Lemaître G, Varoquaux G, Liem F, et al. Combining magnetoencephalog-

raphy with magnetic resonance imaging enhances learning of surrogate-biomarkers. eLife 2020 may;9:e54055. https://doi.org/10.7554/eLife.54055.
12. Collins R. What makes UK Biobank special? The Lancet 2012;379(9822):1173–1174.
13. Woo CW, Chang LJ, Lindquist MA, Wager TD. Building better biomarkers: brain models in translational neuroimaging. Nature Neuroscience 2017;20(3):365–377.
14. Varoquaux G. Cross-validation failure: Small sample sizes lead to large error bars. NeuroImage 2018;180:68–77.
15. Bzdok D, Meyer-Lindenberg A. Machine learning for precision psychiatry: opportunities and challenges. Biological Psychiatry: Cognitive Neuroscience and Neuroimaging 2018;3(3):223–230.
16. Dosenbach NU, Nardos B, Cohen AL, Fair DA, Power JD, Church JA, et al. Prediction of individual brain maturity using fMRI. Science 2010;329(5997):1358–1361.
17. Cole JH, Leech R, Sharp DJ, Initiative ADN. Prediction of brain age suggests accelerated atrophy after traumatic brain injury. Annals of neurology 2015;77(4):571–581.
18. Cole JH, Ritchie SJ, Bastin ME, Hernández MV, Maniega SM, Royle N, et al. Brain age predicts mortality. Molecular psychiatry 2018;23(5):1385.
19. Smith SM, Elliott LT, Alfaro-Almagro F, McCarthy P, Nichols TE, Douaud G, et al. Brain aging comprises many modes of structural and functional change with distinct genetic and biophysical associations. Elife 2020;9:e52677.
20. Koutsouleris N, Davatzikos C, Borgwardt S, Gaser C, Bottlender R, Frodl T, et al. Accelerated brain aging in schizophrenia and beyond: a neuroanatomical marker of psychiatric disorders. Schizophrenia bulletin 2014;40(5):1140–1153.
21. He T, An L, Feng J, Bzdok D, Holmes AJ, Eickhoff SB, et al. Meta-matching: a simple framework to translate phenotypic predictive models from big to small data. bioRxiv 2020;.
22. Liem F, Varoquaux G, Kynast J, Beyer F, Masouleh SK, Huntenburg JM, et al. Predicting brain-age from multimodal imaging data captures cognitive impairment. NeuroImage 2017;148:179–188.
23. Spiegelhalter D. How old are you, really? Communicating chronic risk through 'effective age'of your body and organs. BMC medical informatics and decision making 2016;16(1):1–6.
24. Smith SM, Vidaurre D, Alfaro-Almagro F, Nichols TE, Miller KL. Estimation of brain age delta from brain imaging. NeuroImage 2019;.
25. Gonneaud J, Baria AT, Binette AP, Gordon BA, Chhatwal JP, Cruchaga C, et al. Functional brain age prediction suggests accelerated aging in preclinical familial Alzheimer's disease, irrespective of fibrillar amyloid-beta pathology. bioRxiv 2020;.
26. Cattell RB, Scheier IH. The meaning and measurement of neuroticism and anxiety. 1961;.
27. Cattell RB. Theory of fluid and crystallized intelligence: A critical experiment. Journal of educational psychology 1963;54(1):1.
28. Shelton JT, Elliott EM, Matthews RA, Hill B, Gouvier W, others. The relationships of working memory, secondary memory, and general fluid intelligence: working memory is special. Journal of Experimental Psychology: Learning, Memory, and Cognition 2010;36(3):813.
29. Keyes KM, Platt J, Kaufman AS, McLaughlin KA. Association of Fluid Intelligence and Psychiatric Disorders in a Population-Representative Sample of US Adolescents. JAMA psychiatry 2017;74(2):179–188.
30. Khandaker GM, Dalman C, Kappelmann N, Stochl J, Dal H, Kosidou K, et al. Association of Childhood Infection With IQ and Adult Nonaffective Psychosis in Swedish Men: A Population-Based Longitudinal Cohort and Co-relative Study. JAMA Psychiatry 2018;75(4):356–362.
31. Costa PT, McCrae RR. Neo Pi-R. Psychological Assessment Resources Odessa, FL; 1992.
32. Eysenck SB, Eysenck HJ, Barrett P. A Revised Version of the Psychoticism Scale. Personality and Individual Differences 1985;6:21–29.
33. Shackman AJ, Tromp DP, Stockbridge MD, Kaplan CM, Tillman RM, Fox AS. Dispositional negativity: An integrative psychological and neurobiological perspective. Psychological bulletin 2016;142(12):1275.
34. Lynn R, Martin T. Gender differences in extraversion, neuroticism, and psychoticism in 37 nations. The Journal of social psychology 1997;137(3):369–373.
35. Hettema JM, Neale MC, Myers JM, Prescott CA, Kendler KS. A population-based twin study of the relationship between neuroticism and internalizing disorders. American journal of Psychiatry 2006;163(5):857–864.
36. Birley AJ, Gillespie NA, Heath AC, Sullivan PF, Boomsma DI, Martin NG. Heritability and nineteen-year stability of long and short EPQ-R Neuroticism scales. Personality and individual differences 2006;40(4):737–747.
37. Pedersen NL, Plomin R, McClearn GE, Friberg L. Neuroticism, extraversion, and related traits in adult twins reared apart and reared together. Journal of personality and social psychology 1988;55(6):950.
38. Lahey BB. Public health significance of neuroticism. American Psychologist 2009;64(4):241.
39. Tyrer P, Reed GM, Crawford MJ. Classification, assessment, prevalence, and effect of personality disorder. The Lancet 2015;385(9969):717–726.
40. Power RA, Pluess M. Heritability estimates of the Big Five personality traits based on common genetic variants. Translational psychiatry 2015;5(7):e604.
41. Vukasović T, Bratko D. Heritability of personality: a meta-analysis of behavior genetic studies. Psychological bulletin 2015;141(4):769.
42. Yarkoni T. Neurobiological substrates of personality: A critical overview. APA handbook of personality and social psychology 2015;4:61–83.
43. Dubois J, Galdi P, Han Y, Paul LK, Adolphs R. Resting-State Functional Brain Connectivity Best Predicts the Personality Dimension of Openness to Experience. Personality Neuroscience 2018;1.
44. Dubois J, Galdi P, Paul LK, Adolphs R. A distributed brain network predicts general intelligence from resting-state human neuroimaging data. Philosophical Transactions of the Royal Society B: Biological Sciences 2018;373(1756):20170284.
45. Quercia D, Kosinski M, Stillwell D, Crowcroft J. Our twitter profiles, our selves: Predicting personality with twitter; 2011. p. 180–185.
46. Youyou W, Kosinski M, Stillwell D. Computer-based personality judgments are more accurate than those made by humans. Proceedings of the National Academy of Sciences 2015;112(4):1036–1040.
47. Horn JL, Donaldson G, Engstrom R. Apprehension, Memory, and Fluid Intelligence Decline in Adulthood. Research on Aging 1981;3(1):33–84. https://doi.org/10.1177/016402758131002.
48. Eysenck HJ. The continuity of abnormal and normal behavior. Psychological Bulletin 1958;55(6):429–432.
49. Perlis RH. Translating biomarkers to clinical practice. Molecular Psychiatry 2011;16(11):1076–1087.
50. Stevens SS, et al. On the theory of scales of measurement

1946;.
51. O'brien RM. A caution regarding rules of thumb for variance inflation factors. Quality & quantity 2007;41(5):673–690.
52. Varoquaux G, Raamana PR, Engemann DA, Hoyos-Idrobo A, Schwartz Y, Thirion B. Assessing and tuning brain decoders: Cross-validation, caveats, and guidelines. NeuroImage 2017;145:166 – 179. http://www.sciencedirect.com/science/article/pii/S105381191630595X, individual Subject Prediction.
53. Gelman A, Hill J. Data analysis using regression and multilevel/hierarchical models. Cambridge university press; 2006.
54. Borsboom D. Measuring the mind: Conceptual issues in contemporary psychometrics. Cambridge University Press; 2005.
55. Terracciano A, Costa Jr PT. Smoking and the Five-Factor Model of personality. Addiction 2004;99(4):472–481.
56. Topiwala A, Allan CL, Valkanova V, Zsoldos E, Filippini N, Sexton C, et al. Moderate alcohol consumption as risk factor for adverse brain outcomes and cognitive decline: longitudinal cohort study. bmj 2017;357:j2353.
57. Wang J, Knol MJ, Tiulpin A, Dubost F, de Bruijne M, Vernooij MW, et al. Gray matter age prediction as a biomarker for risk of dementia. Proceedings of the National Academy of Sciences 2019;116(42):21213–21218.
58. Bzdok D, Engemann D, Thirion B. Inference and Prediction Diverge in Biomedicine. Cell Press Patterns 2020 2020/10/13;https://doi.org/10.1016/j.patter.2020.100119.
59. Cole JH, Poudel RP, Tsagkrasoulis D, Caan MW, Steves C, Spector TD, et al. Predicting brain age with deep learning from raw imaging data results in a reliable and heritable biomarker. NeuroImage 2017;163:115–124.
60. He T, Kong R, Holmes AJ, Sabuncu MR, Eickhoff SB, Bzdok D, et al. Is deep learning better than kernel regression for functional connectivity prediction of fluid intelligence? In: 2018 International Workshop on Pattern Recognition in Neuroimaging (PRNI) IEEE; 2018. p. 1–4.
61. Carretta TR. Pilot candidate selection method. Aviation Psychology and Applied Human Factors 2011;.
62. Carretta TR, Ree MJ. Pilot-candidate selection method: Sources of validity. The International Journal of Aviation Psychology 1994;4(2):103–117.
63. Krapohl E, Rimfeld K, Shakeshaft NG, Trzaskowski M, McMillan A, Pingault JB, et al. The high heritability of educational achievement reflects many genetically influenced traits, not just intelligence. Proceedings of the national academy of sciences 2014;111(42):15273–15278.
64. Colodro-Conde L, Couvy-Duchesne B, Zhu G, Coventry WL, Byrne EM, Gordon S, et al. A direct test of the diathesis–stress model for depression. Molecular psychiatry 2018;23(7):1590–1596.
65. Anatürk M, Kaufmann T, Cole JH, Suri S, Griffanti L, Zsoldos E, et al. Prediction of brain age and cognitive age: Quantifying brain and cognitive maintenance in aging. Human brain mapping 2021;42(6):1626–1640.
66. Maglanoc LA, Kaufmann T, Meer Dvd, Marquand AF, Wolfers T, Jonassen R, et al. Brain Connectome Mapping of Complex Human Traits and Their Polygenic Architecture Using Machine Learning. Biological Psychiatry 2020;87(8):717–726.
67. Khandaker GM, Barnett JH, White IR, Jones PB. A quantitative meta-analysis of population-based studies of premorbid intelligence and schizophrenia. Schizophrenia research 2011;132(2-3):220–227.
68. Corrigan-Curay J, Sacks L, Woodcock J. Real-world evidence and real-world data for evaluating drug safety and effectiveness. Jama 2018;320(9):867–868.
69. Sudlow C, Gallacher J, Allen N, Beral V, Burton P, Danesh J, et al. UK Biobank: An Open Access Resource for Identifying the Causes of a Wide Range of Complex Diseases of Middle and Old Age. PLOS Medicine 2015;12(3):1–10.
70. Miller KL, Alfaro-Almagro F, Bangerter NK, Thomas DL, Yacoub E, Xu J, et al. Multimodal population brain imaging in the UK Biobank prospective epidemiological study. Nature neuroscience 2016;19(11):1523–1536.
71. Schulz MA, Yeo BT, Vogelstein JT, Mourao-Miranada J, Kather JN, Kording K, et al. Different scaling of linear models and deep learning in UKBiobank brain images versus machine-learning datasets. Nature communications 2020;11(1):1–15.
72. Flint C, Cearns M, Opel N, Redlich R, Mehler DM, Emden D, et al. Systematic misestimation of machine learning performance in neuroimaging studies of depression. Neuropsychopharmacology 2021;46(8):1510–1517.
73. IJ H. Statistics versus machine learning. Nature methods 2018;15(4):233.
74. Alfaro-Almagro F, Jenkinson M, Bangerter NK, Andersson JLR, Griffanti L, Douaud G, et al. Image processing and Quality Control for the first 10,000 brain imaging datasets from UK Biobank. NeuroImage 2018;166:400–424.
75. Fry A, Littlejohns TJ, Sudlow C, Doherty N, Adamska L, Sprosen T, et al. Comparison of Sociodemographic and Health-Related Characteristics of UK Biobank Participants With Those of the General Population. American Journal of Epidemiology 2017 06;186(9):1026–1034. https://doi.org/10.1093/aje/kwx246.
76. Little RJA, Rubin DB. Statistical Analysis with Missing Data. New York, NY, USA: John Wiley & Sons, Inc.; 1986.
77. Josse J, Prost N, Scornet E, Varoquaux G. On the consistency of supervised learning with missing values; 2019, https://hal.archives-ouvertes.fr/hal-02024202, working paper or preprint.
78. Lerch JP, van der Kouwe AJ, Raznahan A, Paus T, Johansen-Berg H, Miller KL, et al. Studying neuroanatomy using MRI. Nature neuroscience 2017;20:314 – 326.
79. Ritchie SJ, Dickie DA, Cox SR, Valdes Hernandez MdC, Corley J, Royle NA, et al. Brain volumetric changes and cognitive ageing during the eighth decade of life. Human Brain Mapping 2015;36(12):4910–4925.
80. Cox S, Ritchie S, Fawns-Ritchie C, Tucker-Drob E, Deary I. Structural brain imaging correlates of general intelligence in UK Biobank. Intelligence 2019;76:101376.
81. Thompson PM, Hayashi KM, Dutton RA, Chiang MC, Leow AD, Sowell ER, et al. Tracking Alzheimer's Disease. Annals of the New York Academy of Sciences 2007;1097:183–214.
82. Desikan RS, Ségonne F, Fischl B, Quinn BT, Dickerson BC, Blacker D, et al. An automated labeling system for subdividing the human cerebral cortex on MRI scans into gyral based regions of interest. Neuroimage 2006;31(3):968–980.
83. Diedrichsen J, Balsters JH, Flavell J, Cussans E, Ramnani N. A probabilistic MR atlas of the human cerebellum. NeuroImage 2009;46(1):39 – 46.
84. Behrens TEJ, Woolrich MW, Jenkinson M, Johansen-Berg H, Nunes RG, Clare S, et al. Characterization and propagation of uncertainty in diffusion-weighted MR imaging. Magnetic Resonance in Medicine 2003;50:1077–1088.
85. Conturo TE, Lori NF, Cull TS, Akbudak E, Snyder AZ, Shimony JS, et al. Tracking neuronal fiber pathways in the living human brain. Proceedings of the National Academy of Sciences 1999;96:10422–10427.
86. Saygin ZM, Osher DE, Norton ES, Youssoufian DA, Beach

SD, Feather J, et al. Connectivity precedes function in the development of the visual word form area. Nature neuroscience 2016;19.
87. de Groot M, Vernooij MW, Klein S, Ikram MA, Vos FM, Smith SM, et al. Improving alignment in Tract-based spatial statistics: Evaluation and optimization of image registration. NeuroImage 2013;76:400–411.
88. Biswal B, Yetkin FZ, Haughton VM, Hyde JS. Functional connectivity in the motor cortex of resting human brain using echo-planar mri. Magnetic Resonance in Medicine 1995;34(4):537–541.
89. Greicius MD, Srivastava G, Reiss AL, Menon V. Default-mode network activity distinguishes Alzheimer's disease from healthy aging: evidence from functional MRI. Proceedings of the National Academy of Sciences 2004;101:4637.
90. Ledoit O, Wolf M. Honey, I shrunk the sample covariance matrix. The Journal of Portfolio Management 2004;30(4):110–119.
91. Varoquaux G, Baronnet F, Kleinschmidt A, Fillard P, Thirion B. Detection of brain functional-connectivity difference in post-stroke patients using group-level covariance modeling. Medical image computing and computer-assisted intervention: MICCAI International Conference on Medical Image Computing and Computer-Assisted Intervention 2010;13(Pt 1):200–208.
92. Sabbagh D, Ablin P, Varoquaux G, Gramfort A, Engeman DA. Manifold-regression to predict from MEG/EEG brain signals without source modeling. In: Advances in Neural Information Processing Systems (NeurIPS); 2019. .
93. Dadi K, Rahim M, Abraham A, Chyzhyk D, Milham M, Thirion B, et al. Benchmarking functional connectome-based predictive models for resting-state fMRI. NeuroImage 2019;192:115–134.
94. Pervaiz U, Vidaurre D, Woolrich MW, Smith SM. Optimising network modelling methods for fMRI. NeuroImage 2020;211:116604.
95. Abraham A, Pedregosa F, Eickenberg M, Gervais P, Mueller A, Kossaifi J, et al. Machine learning for neuroimaging with scikit-learn. Frontiers in Neuroinformatics 2014;8.
96. Poldrack RA, Huckins G, Varoquaux G. Establishment of best practices for evidence for prediction: a review. JAMA psychiatry 2020;77(5):534–540.
97. Breiman L. Random Forests. Machine Learning 2001;45(1):5–32.
98. Hastie T, Tibshirani R, Friedman J, Franklin J. The elements of statistical learning: data mining, inference and prediction. The Mathematical Intelligencer 2005;27(2):83–85.
99. Varoquaux G, Raamana PR, Engemann DA, Hoyos-Idrobo A, Schwartz Y, Thirion B. Assessing and tuning brain decoders: Cross-validation, caveats, and guidelines. NeuroImage 2017;145(August 2015):166–179.
100. O'Connor D, Lake EM, Scheinost D, Constable RT. Resample aggregating improves the generalizability of connectome predictive modeling. NeuroImage 2021;236:118044.
101. Gemein LAW, Schirrmeister RT, Chrabąszcz P, Wilson D, Boedecker J, Schulze-Bonhage A, et al. Machine-learning-based diagnostics of EEG pathology. NeuroImage 2020;220:117021. http://www.sciencedirect.com/science/article/pii/S1053811920305073.
102. Le TT, Kuplicki RT, McKinney BA, Yeh HW, Thompson WK, Paulus MP, et al. A Nonlinear Simulation Framework Supports Adjusting for Age When Analyzing BrainAGE. Frontiers in Aging Neuroscience 2018;10:317. https://www.frontiersin.org/article/10.3389/fnagi.2018.00317.
103. Smith SM, Vidaurre D, Alfaro-Almagro F, Nichols TE, Miller KL. Estimation of brain age delta from brain imaging. NeuroImage 2019;200:528 – 539.
104. Pedregosa F, Varoquaux G, Gramfort A, Michel V, Thirion B, Grisel O, et al. Scikit-learn: Machine Learning in Python. J Mach Learn Res 2011 nov;12:2825–2830.
105. R Core Team. R: A Language and Environment for Statistical Computing. R Foundation for Statistical Computing, Vienna, Austria; 2019, https://www.R-project.org/.
106. Wickham H. ggplot2: Elegant Graphics for Data Analysis. Springer-Verlag New York; 2016. http://ggplot2.org.
107. Clarke E, Sherrill-Mix S. ggbeeswarm: Categorical Scatter (Violin Point) Plots; 2017, https://CRAN.R-project.org/package=ggbeeswarm, r package version 0.6.0.
108. Gelman A, Su YS. arm: Data Analysis Using Regression and Multilevel/Hierarchical Models; 2020, https://CRAN.R-project.org/package=arm, r package version 1.11-1.

Supporting Information

Appendix 1: Additional results

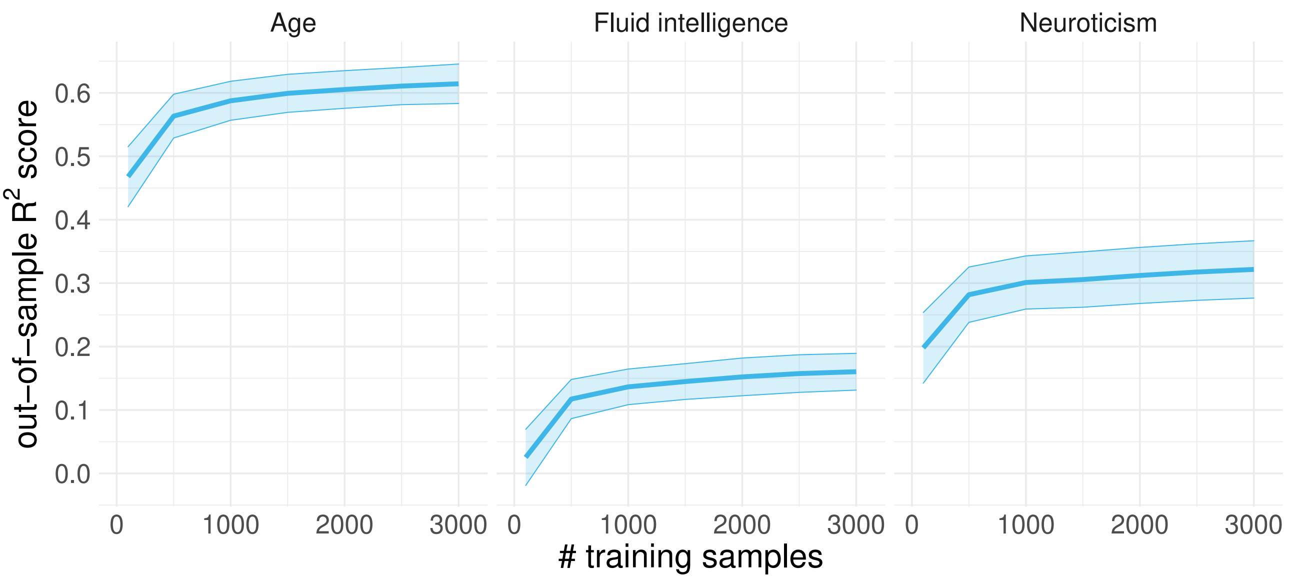

Figure 1 – Figure supplement 1. Learning curves on the random split-half validation used for model building. To facilitate comparisons, we evaluated predictions of age, fluid intelligence and neuroticism from a complete set of socio-demographic variables without brain imaging using the coefficient of determination R^2 metric (y-axis) to compare results obtained from 100 to 3000 training samples (x-axis). The cross-validation (CV) distribution was obtained from 100 Monte Carlo splits. Across targets, performance started to plateau after around 1000 training samples with scores virtually identical to the final model used in subsequent analyses. These benchmarks suggest that inclusion of additional training samples would not have led to substantial improvements in performance.

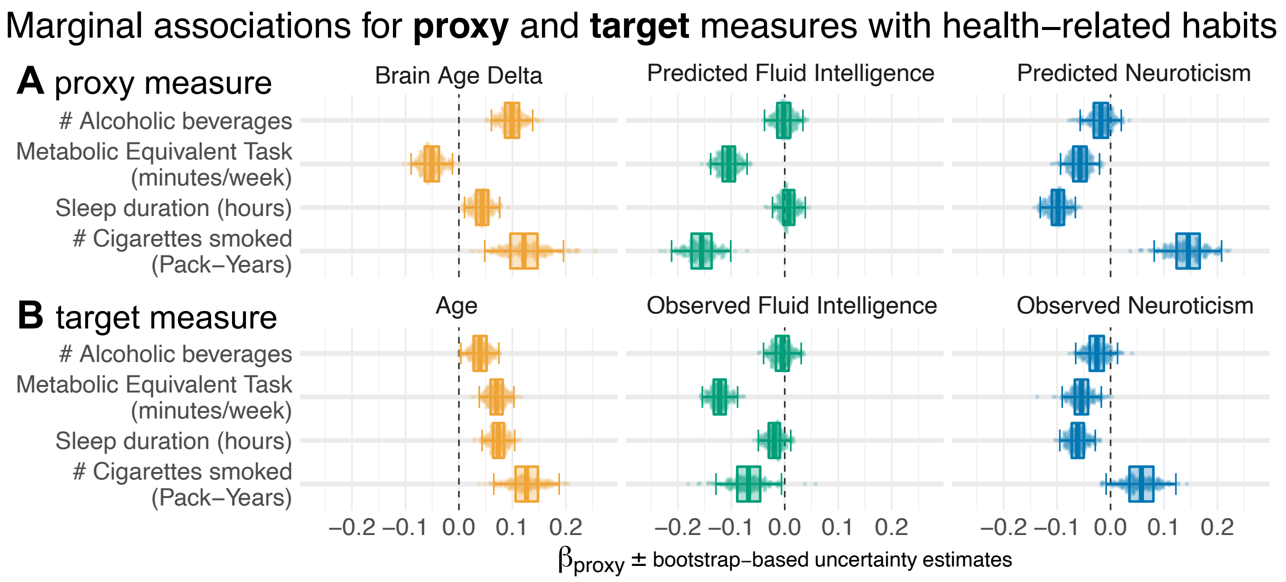

Figure 2 – Figure supplement 1. Marginal associations between proxy measures and health-related habits. Marginal (instead of conditional) estimates using univariate regression. Same visual conventions as in Figure 2 – Figure supplement 1.

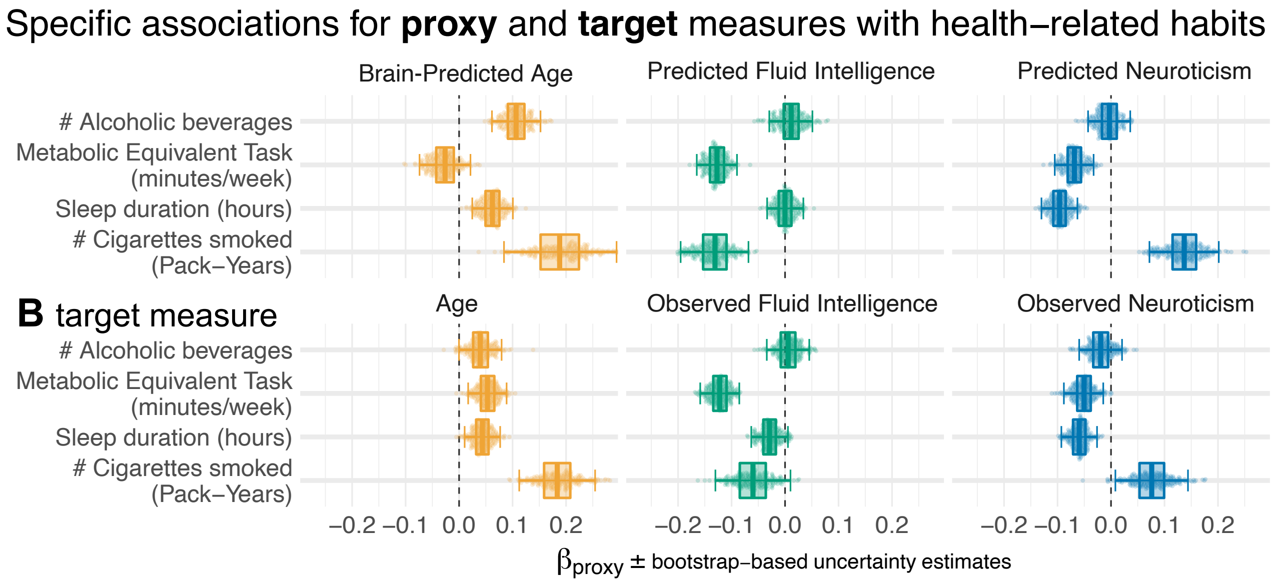

Figure 2 – Figure supplement 2. **Conditional associations between proxy measures and health-related habits without explicit brain age delta**. Conditional estimates using multivariate regression. Instead of the brain age delta, the brain-predicted age is included alongside an age-deconfounder as used in the main analysis. Same visual conventions as in Figure 2.

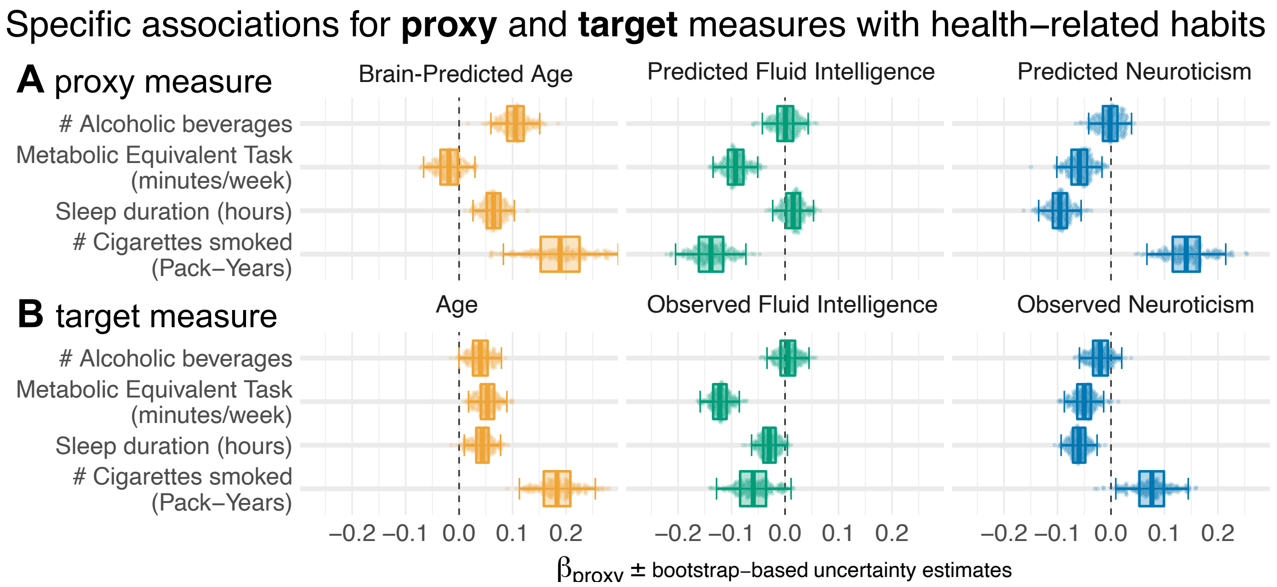

Figure 2 – Figure supplement 3. **Conditional associations between proxy measures and health-related habits with-proxy-specific deconfounding**. Conditional estimates using multivariate regression. Instead of the brain age delta, the brain-predicted age is included alongside an age-deconfounder as used in the main analysis. Moreover, predicted fluid intelligence and neuroticism are deconfounded for the target values at training time, analogous to the brain age predictions. Same visual conventions as in Figure 2.

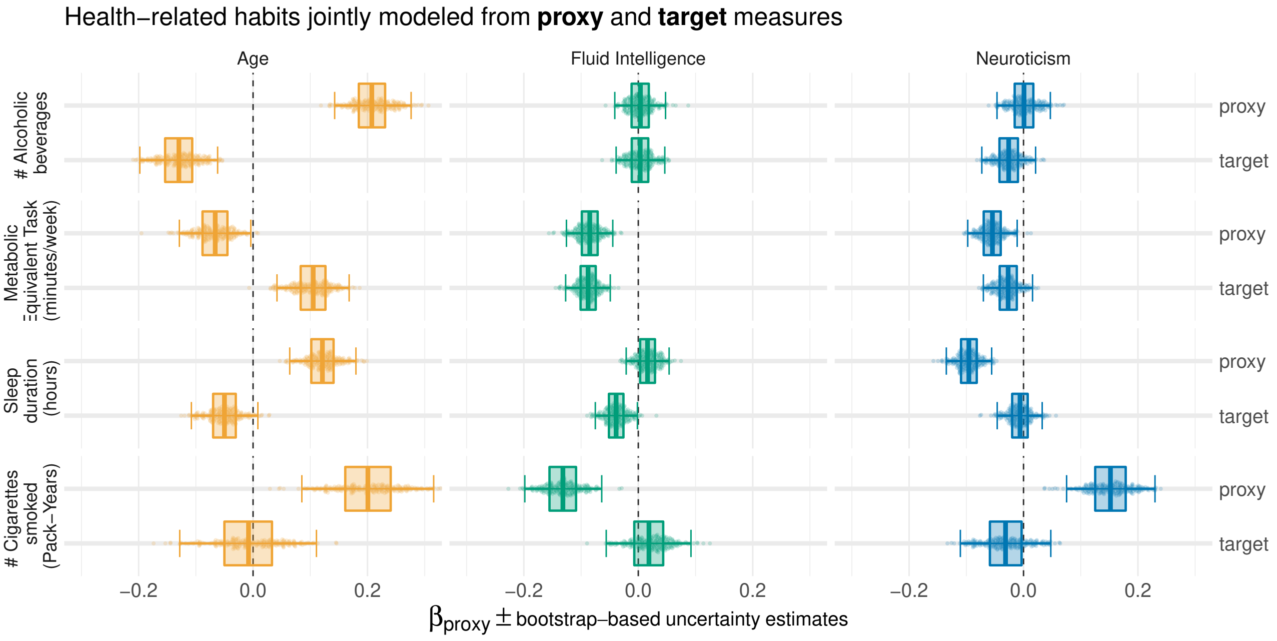

Figure 2 – Figure supplement 4. **Joint modeling of health-related habits from proxy and target measures.** Conditional estimates using multivariate regression. Every health-related habit (double rows) is modeled simultaneously from multiple proxies and targets. Same visual conventions as in Figure 2. Across health-habits, additive effects emerged not only for proxies and targets within the same measure (*e.g.* age) but also across measures (*e.g.* age and fluid intelligence). For illustration, we shall consider two examples. Regarding alcohol consumption, age was the most important measure and opposite conditional effects were observed for the proxy and the target: Across the age range, people with higher brain age tended to drink more and across the brain-age range, older people tended to drink less. For smoking, the proxy measures were the most important variables with clear non-zero coefficients, pointing in different directions across target domains. Holding fluid intelligence and neuroticism constant (targets and proxies), people with higher brain age tended to have been smoking for a longer time. At the same time, those who scored lower on predicted fluid intelligence across the entire range of age, predicted age, measured fluid intelligence, predicted neuroticism and neuroticism, have been smoking for a longer time. Finally, those who scored higher on predicted neuroticism tended to smoke more across the ranges of all other measures.

Table S1. Paired difference between purely sociodemographic and models including brain imaging on held-out data.

Target	sociodemographics	R^2_{diff}	p-value	CI_{low}	CI_{high}
Age	Early Life	0.494	0.0001	0.473	0.515
Age	Education	0.458	0.0001	0.437	0.479
Age	Life style	0.071	0.0001	0.058	0.085
Age	Mood & sentiment	0.294	0.0001	0.272	0.315
Fluid intelligence	Age, Sex	0.048	0.0001	0.040	0.057
Fluid intelligence	Early Life	0.039	0.0001	0.027	0.050
Fluid intelligence	Education	0.018	0.0001	0.010	0.025
Fluid intelligence	Life style	0.030	0.0001	0.020	0.040
Fluid intelligence	Mood & sentiment	0.031	0.0001	0.019	0.043
Neuroticism	Age, Sex	0.001	0.6789	−0.006	0.008
Neuroticism	Early Life	0.010	0.0697	−0.001	0.021
Neuroticism	Education	0.009	0.0817	−0.001	0.020
Neuroticism	Life style	−0.008	0.1750	−0.020	0.004
Neuroticism	Mood & sentiment	−0.030	0.0001	−0.041	−0.018

Table S2. Difference statistics for classification on the held-out set for sociodemographic vs combined approximation.

Target	$AUC_{diff\ observed}$	p-value	CI_{low}	CI_{high}
Age	0.013	0.0008	0.006	0.021
Fluid intelligence	−0.031	0.0001	−0.044	−0.017
Neuroticism	−0.003	0.4818	−0.013	0.006

Table S3. Inferential statistics for joint proxy-target models of health-related habits

	Outcome			
	Alcohol	Activity	Sleep	Smoking
predicted Age	0.208***	−0.066**	0.121***	0.200***
	(0.034)	(0.032)	(0.029)	(0.058)
Age	−0.129***	0.105***	−0.050*	−0.008
	(0.035)	(0.032)	(0.030)	(0.060)
predicted Fluid Intelligence	0.004	−0.085***	0.016	−0.132***
	(0.022)	(0.021)	(0.019)	(0.035)
Fluid Intelligrence	0.003	−0.088***	−0.038**	0.018
	(0.022)	(0.020)	(0.019)	(0.038)
predicted Neuroticism	0.001	−0.054**	−0.095***	0.151***
	(0.024)	(0.022)	(0.020)	(0.040)
Neuroticism	−0.026	−0.027	−0.006	−0.031
	(0.024)	(0.022)	(0.020)	(0.041)
Constant	−0.001	0.018	0.017	−0.052
	(0.019)	(0.018)	(0.017)	(0.034)
Observations	2,687	3,022	3,504	896
R^2	0.016	0.031	0.020	0.071
Adjusted R^2	0.014	0.029	0.018	0.064
Residual Std. Error	1.004 (df = 2680)	0.997 (df = 3015)	0.992 (df = 3497)	0.992 (df = 889)
F Statistic	7.334*** (df = 6; 2680)	15.854*** (df = 6; 3015)	11.733*** (df = 6; 3497)	11.256*** (df = 6; 889)
Note:				*p<0.1; **p<0.05; ***p<0.01

Table S4. Variance Inflation Factors (VIF) for joint proxy-target models of health-related habits

	Alcohol	Activity	Sleep	Smoking
predicted Age	3.063	3.149	3.076	3.000
Age	3.108	3.181	3.123	3.070
predicted Fluid Intelligence	1.259	1.254	1.266	1.254
Fluid Intelligrence	1.220	1.223	1.229	1.229
predicted Neuroticism	1.451	1.457	1.460	1.590
Neuroticism	1.434	1.435	1.439	1.552

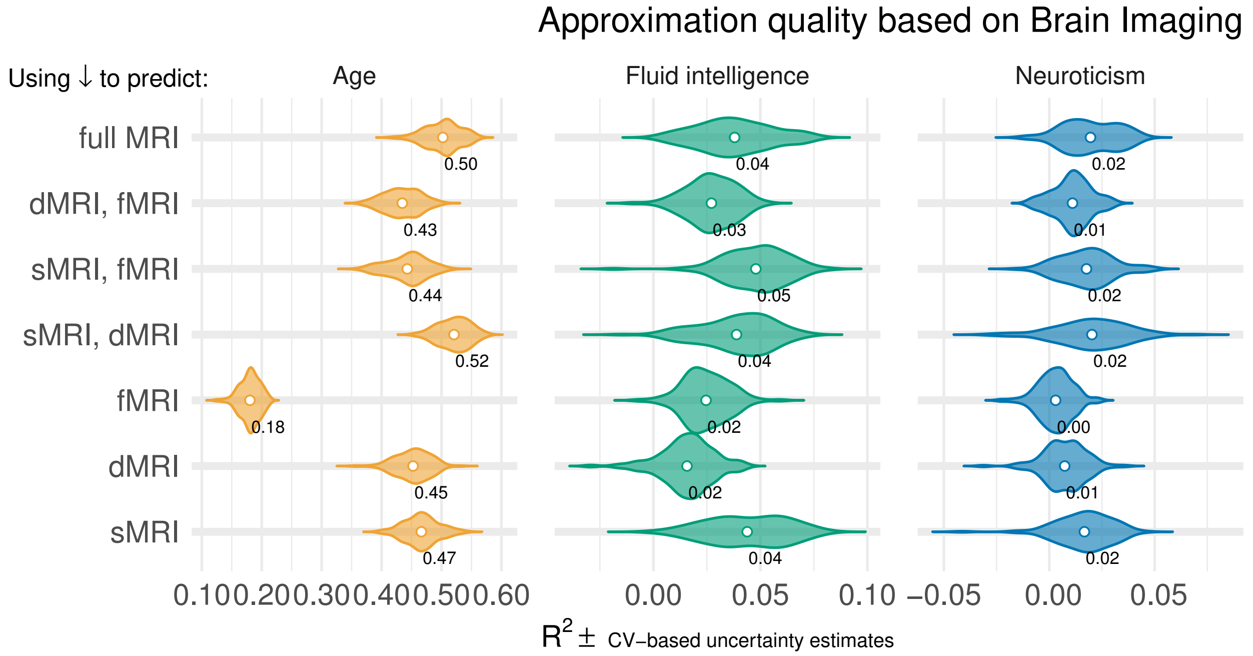

Figure 3 – Figure supplement 1. Prediction of individual differences in proxy measures from MRI. Approximation performance using multiple MR modalities on the validation dataset: sMRI, dMRI, rfMRI and their combinations (see Table 1). Visual conventions as in Figure 3. One can see that prediction of age was markedly stronger than prediction of fluid intelligence or prediction of neuroticism. As a general trend, models based on multiple MRI modalities tended to yield better prediction. For simplicity, we based subsequent analyses on the full model based on all MRI data.

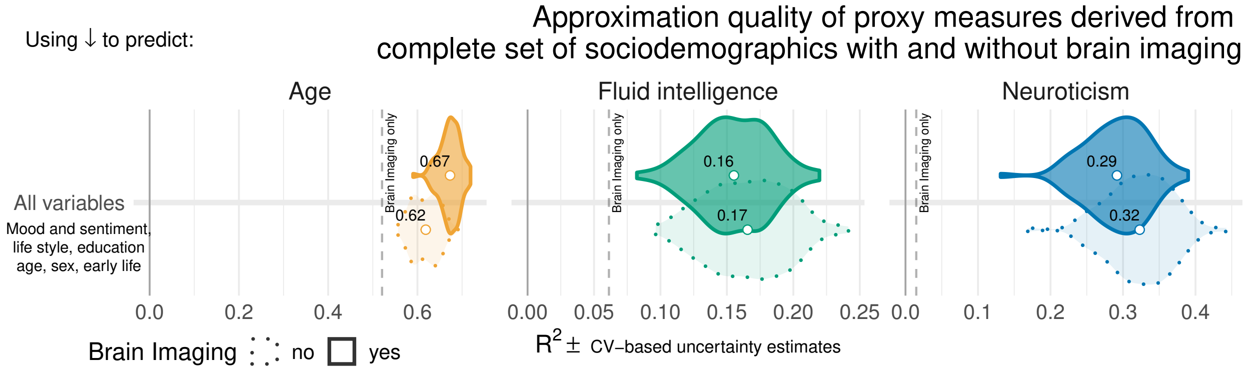

Figure 3 – Figure supplement 2. Approximation performance using all sociodemographic data. Approximation performance using all sociodemographic variables with or without brain imaging included on the validation dataset. Visual conventions as in Figure 3. The performance was highly related to the best performing models within each target Figure 3, *i.e.*, life style for age, education for fluid intelligence and mood & sentiment for neuroticism. This suggests that for each target those specific blocks of predictors were sufficiently explaining the performance. For simplicity, we based subsequent analyses in Figure 4 and Figure 2 on all sociodemographic variables.

Table S5. Regression statistics on the held-out set for purely MRI-based approximation.

Target	$R^2_{observed}$	p-value	CI_{low}	CI_{high}
Age	0.521	1×10^{-4}	0.502	0.538
Fluid intelligence	0.061	1×10^{-4}	0.052	0.070
Neuroticism	0.015	1×10^{-4}	0.005	0.024

Table S6. Classification difference statistics on the held-out set for MRI-based approximation.

Target	$AUC_{observed}$	p-value	CI_{low}	CI_{high}
Neuroticism	0.590	1×10^{-4}	0.566	0.614
Age	0.916	1×10^{-4}	0.905	0.927
Fluid intelligence	0.667	1×10^{-4}	0.643	0.690

Appendix 2: Sociodemographic variables

Table S7. List of variables contained in each block of sociodemographic models: mood & sentiment (MS), Age, Sex (AS), Education (EDU), Early life (EL).

Group	UKBB code	Variables
**Mood & Sentiment**	2040-2.0	Risk taking
	4526-2.0	Happiness
	4537-2.0	Work/job satisfaction
	4548-2.0	Health satisfaction
	4559-2.0	Family relationship satisfaction
	4570-2.0	Friendships satisfaction
	4581-2.0	Financial situation satisfaction
	4598-2.0	Ever depressed for a whole week
	4609-2.0	Longest period of depression
	4620-2.0	Number of depression episodes
	4631-2.0	Ever unenthusiastic/disinterested for a whole week
	4642-2.0	Ever manic/hyper for 2 days
	4653-2.0	Ever highly irritable/argumentative for 2 days
	2050-2.0	Frequency of depressed mood in last 2 weeks
	2060-2.0	Frequency of unenthusiasm / disinterest in last 2 weeks
	2070-2.0	Frequency of tenseness / restlessness in last 2 weeks
	2080-2.0	Frequency of tiredness / lethargy in last 2 weeks
	2090-2.0	Seen doctor (GP) for nerves, anxiety, tension or depression
	2100-1.0	Seen a psychiatrist for nerves, anxiety, tension or depression
	5375-2.0	Longest period of unenthusiasm / disinterest
	5386-2.0	Number of unenthusiastic/disinterested episodes
	5663-2.0	Length of longest manic/irritable episode
	5674-2.0	Severity of manic/irritable episode
	6145-2.0	Illness, injury, bereavement, stress in last 2 years
	6156-2.0	Manic/hyper symptoms
**Age, Sex**	31-0.0	Sex
	34-0.0	Year of birth
	52-0.0	Month of birth
	21022-0.0	Age at recruitment
	21003-2.0	Age when attended assessment centre
**Education**	6138-2.0	Qualifications
	845-2.0	Age completed full time education
**Early life**	1647-2.0	Country of birth (UK/elsewhere)
	1677-2.0	Breastfed as a baby
	1687-2.0	Comparative body size at age 10
	1697-2.0	Comparative height size at age 10
	1707-2.0	Handedness (chirality/laterality)
	1767-2.0	Adopted as a child
	1777-2.0	Part of a multiple birth
	1787-2.0	Maternal smoking around birth
**Lifestyle**	670-2.0	Type of accommodation lived in
	680-2.0	Own or rent accommodation lived in
	6139-2.0	Gas or solid-fuel cooking/heating
	699-2.0	Length of time at current address
	709-2.0	Number in household
	6141-2.0	How are people in household related to participant

Table S7 continued

728-2.0	Number of vehicles in household
738-2.0	Income before tax
796-2.0	Distance between home and job workplace
757-2.0	Time employed in main current job
767-2.0	Length of working week for main job
777-2.0	Freq. of travelling from home to job workplace
6143-2.0	Transport type for commuting to job workplace
6142-2.0	Current employment status
806-2.0	Job involves mainly walking or standing
816-2.0	Job involves heavy manual or physical work
826-2.0	Job involves shift work
3426-2.0	Job involves night shift work
1031-2.0	Freq. of friend/ family visits
6160-2.0	Leisure/social activities
2110-2.0	Able to confide
1239-2.0	Current tobacco smoking
1249-2.0	Past tobacco smoking
1259-2.0	Smoking/smokers in household
1269-2.0	Exposure to tobacco smoke at home
1279-2.0	Exposure to tobacco smoke outside home
2644-2.0	Light smokers, at least 100 smokes in lifetime
2867-2.0	Age started smoking in former smokers
2877-2.0	Type of tobacco previously smoked
2887-2.0	Number of cigarettes previously smoked daily
2897-2.0	Age stopped smoking
2907-2.0	Ever stopped smoking for 6+ months
2926-2.0	Number of unsuccessful stop-smoking attempts
2936-2.0	Likelihood of resuming smoking
3436-2.0	Age started smoking in current smokers
3446-2.0	Type of tobacco currently smoked
3456-2.0	Number of cigarettes currently smoked daily (current cigarette smokers)
3466-2.0	Time from waking to first cigarette
3476-2.0	Difficulty not smoking for 1 day
3486-2.0	Ever tried to stop smoking
3496-2.0	Wants to stop smoking
3506-2.0	Smoking compared to 10 years previous
5959-2.0	Previously smoked cigarettes on most/all days
6157-2.0	Why stopped smoking
6158-2.0	Why reduced smoking

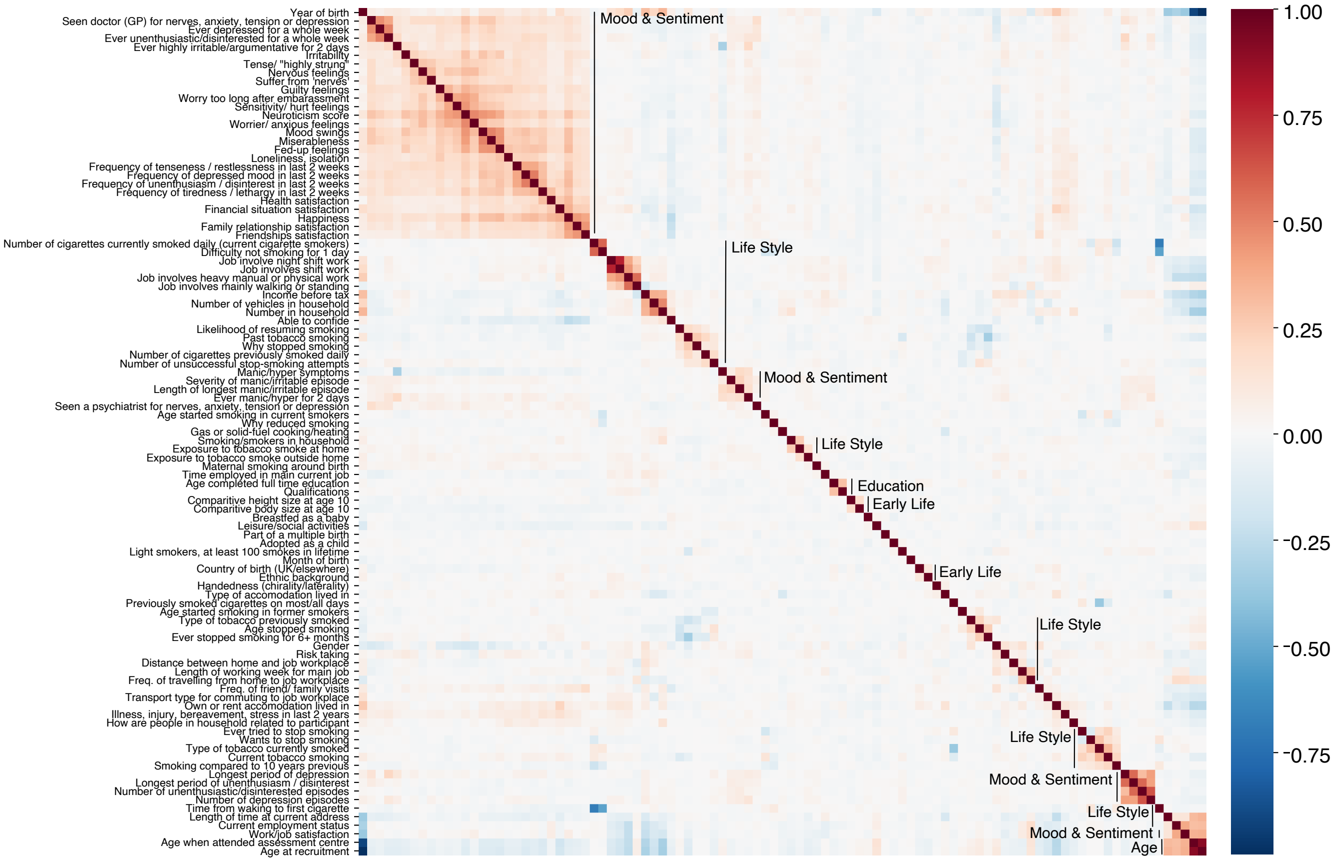

Figure S1. Intercorrelations between sociodemographic inputs. To check the plausibility of the proposed grouping of variables into blocks, we investigated the inter-correlations among the sociodemographic inputs (Table S7). We first applied Yeo-Johnson power transform to the variables yield approximately symmetrical distributions. Then we computed Pearson correlations. One can see that a large majority of variables shows low if any inter-correlations. Strongly inter-correlated blocks emerged, in particular for Mood & Sentiment and Life Style. Note that within the Life Style category many smaller blocks with strong inter-correlation occurred, some of which were obviously related to the circumstance of living such as household or employment status.

Appendix 3: Impact of Measurement Time

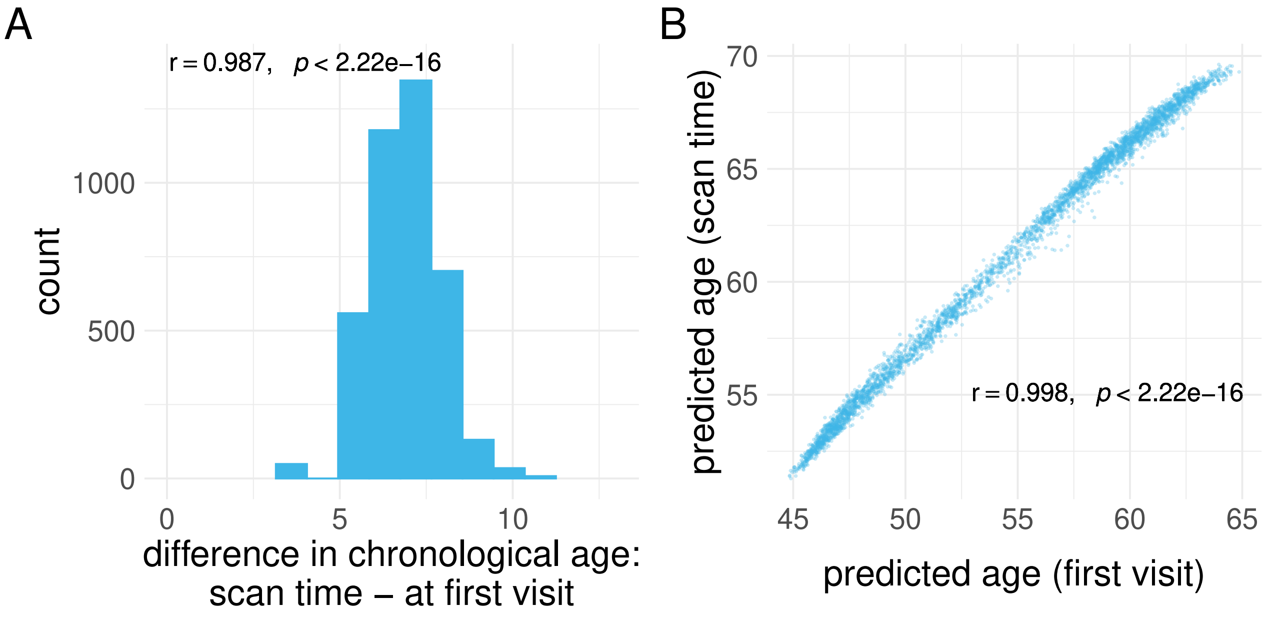

Figure S2. Investigating the age gap between the first visit and the MRI-visit time point. **(A)** Individual gap between age at first visit and MRI-scan time. MRI scans never happened at the first visit, leading to a strictly positive gap greater than five years for most participants. Pearson's correlation coefficient indicates high rank stability, suggesting that, from a statistical perspective, age at first visit and age at scan time are, essentially, interchangeable. **(B)** Direct comparison of individual-specific age predictions from brain images and sociodemographic data. Same model as in the main analysis (Figure 2). The emerging pattern of association summarized by Pearson's correlation coefficient suggests that predictions from models either trained on age at the first visit or at MRI-scan time are equivalent.

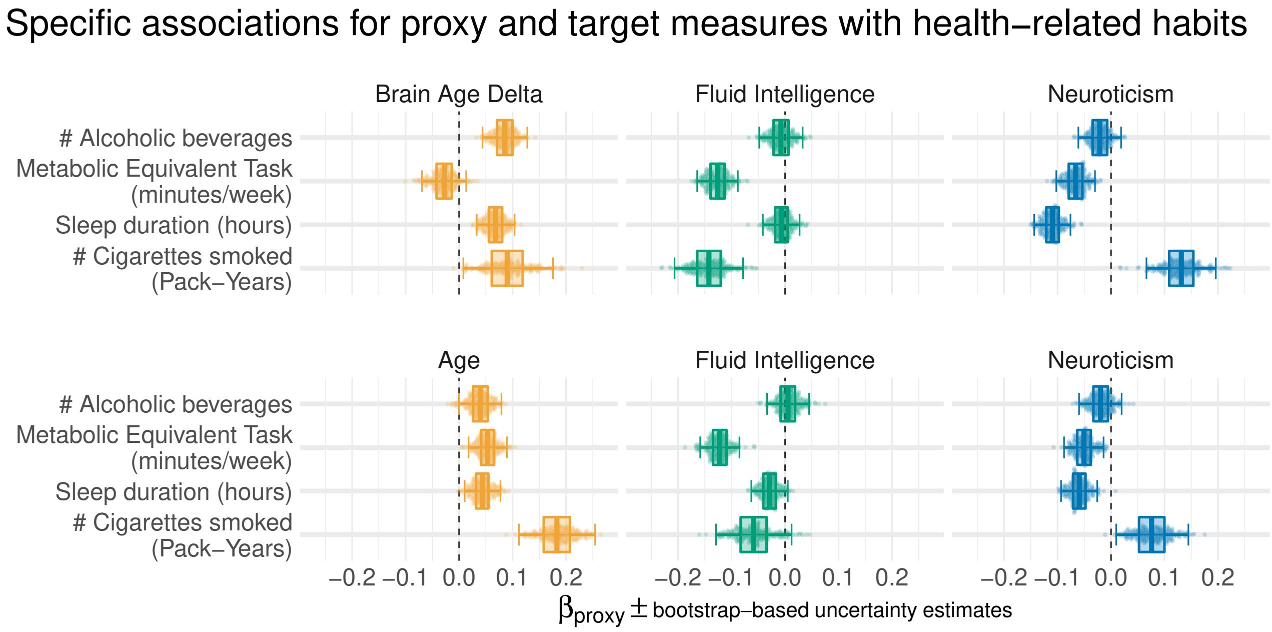

Figure S3. Proxy measures show systematic and complementary out-of-sample associations with health-related habits using age at MRI-scan time. The patterns observed in Figure 2 and global conclusions remain unchanged.
