# Supplementary material for: Population modeling with machine learning can enhance measures of mental health
Source: Gigascience. 2021 Oct 15;10(10):giab071. doi: 10.1093/gigascience/giab071 (PMC8559220; doi:10.1093/gigascience/giab071)
Supplement: giab071_GIGA-D-21-00080_Revision_2 [file giab071_giga-d-21-00080_revision_2.pdf]

GigaScience

Population modeling with machine learning can enhance measures of mental health

--Manuscript Draft--

**Manuscript Number:**	GIGA-D-21-00080R2
**Full Title:**	Population modeling with machine learning can enhance measures of mental health
**Article Type:**	Research
**Funding Information:**	Canada First Research Excellence Fund — Dr. Gaël Varoquaux<br>Canadian Institutes of Health Research (438531) — Dr Danilo Bzdok
**Abstract:**	Background Biological aging is revealed by physical measures, e.g., DNA probes or brain scans. Instead, individual differences in mental function are explained by psychological constructs, e.g., intelligence or neuroticism. These constructs are typically assessed by tailored neuropsychological tests that build on expert judgement and require careful interpretation. Could machine learning on large samples from the general population be used to build proxy measures of these constructs that do not require human intervention?<br>Results Here, we built proxy measures by applying machine learning on multimodal MR images and rich sociodemographic information from the largest biomedical cohort to date: the UK Biobank. Objective model comparisons revealed that all proxies captured the target constructs and were as useful, and sometimes more useful than the original measures for characterizing real-world health behavior (sleep, exercise, tobacco, alcohol consumption). We observed this complementarity of proxy measures and original measures when modeling from brain signals or sociodemographic data, capturing multiple health-related constructs.<br>Conclusions Population modeling with machine learning can derive measures of mental health from brain signals and questionnaire data, which may complement or even substitute for psychometric assessments in clinical populations.
**Corresponding Author:**	Denis-Alexander Engemann<br>Inria Saclay: Inria Centre de Recherche Saclay-Ile-de-France<br>Palaiseau, FRANCE
**Corresponding Author Secondary Information:**	
**Corresponding Author's Institution:**	Inria Saclay: Inria Centre de Recherche Saclay-Ile-de-France
**Corresponding Author's Secondary Institution:**	
**First Author:**	Kamalaker Dadi
**First Author Secondary Information:**	
**Order of Authors:**	Kamalaker Dadi<br>Gaël Varoquaux<br>Josselin Houenou<br>Danilo Bzdok<br>Bertrand Thirion<br>Denis-Alexander Engemann
**Order of Authors Secondary Information:**	
**Response to Reviewers:**	**Please note that a fully formatted and more readable version of the response to the reviewers (including coloured highlights and the new figures and tables in context) is available as PDF among the submitted files.**<br><br>**Note that this text renders to markdown**

Powered by Editorial Manager® and ProduXion Manager® from Aries Systems Corporation

Summary

We would like to thank the editor and the reviewer for the attentive evaluation of our manuscript and the helpful comments. This has allowed us to further refine the manuscript regarding editorial standards and the presentation of our workflow. The key points addressed in this revision are a major update to figure 1 as well as changes in the text that, hopefully, will help the reader to better understand the core ideas of the paper and the methods workflow.

A detailed display of the changes in the manuscript is available in the file `diff-with-r1-submission.pdf` enclosed with the resubmission.

Editorial points

- Please turn URLs mentioned in the text or the footnotes into numbered citations, included in the bibliography. Software homepages, data sets and other URLs or DOIs should all be included in the bibliography and cited from there. please see our instructions for authors for formatting guidance.

- In the data availability section, please explain the access procedure for the UK BioBank data (Is it open or controlled access? For human data, controlled access is fine, but please mention the requirements/procedure to get access).

- Our data curators will prepare an archival snapshot of code and supporting data. Please also include this in the bibliography (including the doi link) and cite it by number from the availability section.

Reply:

We thank the editor for the support regarding editorial standards. In the revised versions, we have addressed all three points.

Reviewer reports:

Reviewer #1:

The substantial revision improved the paper and is appreciated by the reviewer. The details have been enhanced. However, the reviewer still has some **concerns about the basic logic and its presentation of the paper** after reviewing all the comments from other reviewers and the feedback from the author.

Reply:

We thank the reviewer for the positive appreciation of our revision efforts. We hope that the second revision, in response to the thoughtful and constructive criticism provided by the reviewer, helped improve the accessibility issues regarding the basic logic and presentation of the paper.

Reviewer #1

Figure 1 is helpful (BTW, the font is too small and smaller than other figures).

Reply:

We thank the reviewer for this positive feedback and the pointer regarding the font sizes. To address this issue, we have increased the font size of the smallest fonts and scaled up the figure to appear larger inside the manuscript, using the full text width instead of one single column. As a result, all font sizes have been increased.

Reviewer #1:

But if we consider the current approach again, when the machine learning (ML) has

Powered by Editorial Manager® and ProduXion Manager® from Aries Systems Corporation

perfect performance to generate the so called proxy measures, these measures should match exactly each individual's age, fluid-intelligence and neuroticism. What the author claimed about proxy measures providing better assessment to other health related variables **might be simply due to the imperfectness**

Reply:

We thank the reviewer for this opportunity for clarification. The reviewer recognizes an important point about the preconditions for building proxy measures: The predictors from which a proxy measure is modelled should not allow for perfect prediction (which is certainly guaranteed in our context given that the precise data generating mechanisms are unknown and it is reasonable to assume that many important variables remain unobserved). A proxy measure can only bring additional information if, in the first place, there is residual variance in the target that is not explained by the predictors. The entire approach cannot work if proxies and targets are – via perfect prediction – the same.

To make sure this point is not overlooked, in the introduction, we have revised the paragraph in which brain age is introduced. Moreover, we have added a note in the caption figure caption of figure 1.

Changes in the main text:

Yet, by (imperfectly) predicting the age from brain data, machine-learning can capture the relevant signal. Based on a population of brain images, it extracts the _best guess_for the age of a person, indirectly positioning that person within the population.

Related changes in caption of figure 1:

(...) Note that proxy measures can only add to the target measures if they are not identical, _i.e._, if the approximation of the target from the given inputs is imperfect (guaranteed in our context as the exact data generating mechanism is unknown and causally important variables remain unobserved). (...)

Related changes in figure 1 (bold text in panel C):

Machine learning combines various classes of inputs to build (imperfect) proxies for the target measures.

Reviewer #1

The author may need to address this and present the logic of the paper in a clearer way to help the readers understand the main point and results of the paper. In this regard, Figure 1 is incomplete in addressing the full flow of the paper, which is necessary for such a seemingly complex paper in the reviewer's opinion.

Reply:

We thank the reviewer for this excellent recommendation. It is true that because of the substantial revisions, now the concept figure 1 is visibly out of sync with the full story, which indeed may cause confusion or simply make readers miss the main ideas. To present the key ideas of the work with greater clarity to the reader, we have added an outlook on the organization of the paper at the end of the introduction and substantially extended figure 1 to now depict the full workflow of the paper. To generate more attention for the key idea of comparing multiple proxy measures with their respective targets, we have added an explicit illustration of differences between proxies and target measures. To prepare the reader for the core of the paper in which proxies and targets are benchmarked regarding their potential complementarity at statistically explaining health-related behavior, we have included a new panel in figure 1 in which the health behaviors under investigation are illustrated. We have updated the figure caption accordingly.

Changes in the main text (end of introduction):

Powered by Editorial Manager® and ProduXion Manager® from Aries Systems Corporation

	The paper is organized as follows: We first present a summary of the methodology and the workflow of building distinct proxy measures for age, fluid intelligence and neuroticism using machine learning (Figure 1). We then benchmark the proxy and the original target measures against real-world patterns of health-relevant behavior. Subsequently, through systematic model comparisons, we assess the relative contributions of brain imaging and sociodemographic data for prediction performance in the regression and classification settings. The complementarity between the proxy measures is, finally, discussed in the light of statistical considerations, potential data generating mechanisms, and applications for public health and clinical research.<br><br>#### Figure 1 after substantial revisions:<br><br>Please see the PDF version / manuscript for the figure.<br><br>#### Figure 1 caption:<br><br>**Methods workflow: building and evaluating proxy measures.** We combined multiple brain-imaging modalities (**A**) with sociodemographic data (**B**) to approximate health-related biomedical and psychological constructs (**C**), _i.e._, brain age (accessed through prediction of chronological age), cognitive capacity (accessed through a fluid-intelligence test) and the tendency to report negative emotions (accessed through a neuroticism questionnaire). We included the imaging data from the 10,000-subjects release of the UK biobank. Among imaging data (**A**) we considered features related to cortical and subcortical volumes, functional connectivity from rfMRI based on ICA networks, and white-matter molecular tracts from diffusive directions (see Table 1 for an overview about the multiple brain-imaging modalities). We then grouped the sociodemographic data (**B**) into five different blocks of variables related to self-reported mood & sentiment, primary demographics, lifestyle, education, and early-life events (Table 2 lists the number of variables in each block). We systematically compared the approximations of all three targets based on either brain images and sociodemographics in isolation or combined (**C**) to evaluate the relative contribution of these distinct inputs. Note that proxy measures can only add to the target measures if they are not identical, _i.e._, if the approximation of the target from the given inputs is imperfect (guaranteed in our context as the exact data generating mechanism is unknown and causally important variables remain unobserved). Using the full model (brain imaging + sociodemographics), we benchmarked complementarity of the proxy measures and the target measures with regard to real-world patterns of health behavior (**D**), i.e., the number of alcoholic beverages, exercise (metabolic equivalent task), sleep duration and the number of cigarettes smoked. Potentially additive effects between proxies and targets were gauged using multiple linear regression. Models were developed on 50% of the data (randomly drawn) based on random forest regression guided by Monte Carlo cross-validation with 100 splits (see section **Model Development and Generalization Testing**). We assessed generalization and health implications using the other 50% of the data as fully independent out-of-sample evaluations (see section **Statistical Analysis**). Learning curves suggested that this split-half approach provided sufficient data for model construction (Figure 1 – Figure 1 supplement).
**Additional Information:**	
**Question**	**Response**
Are you submitting this manuscript to a special series or article collection?	No
**Experimental design and statistics**<br><br>Full details of the experimental design and statistical methods used should be given in the Methods section, as detailed in our Minimum Standards Reporting Checklist. Information essential to interpreting the	Yes

data presented should be made available in the figure legends.<br><br>Have you included all the information requested in your manuscript?	
**Resources**<br><br>A description of all resources used, including antibodies, cell lines, animals and software tools, with enough information to allow them to be uniquely identified, should be included in the Methods section. Authors are strongly encouraged to cite Research Resource Identifiers (RRIDs) for antibodies, model organisms and tools, where possible.<br><br>Have you included the information requested as detailed in our Minimum Standards Reporting Checklist?	Yes
**Availability of data and materials**<br><br>All datasets and code on which the conclusions of the paper rely must be either included in your submission or deposited in publicly available repositories (where available and ethically appropriate), referencing such data using a unique identifier in the references and in the “Availability of Data and Materials” section of your manuscript.<br><br>Have you have met the above requirement as detailed in our Minimum Standards Reporting Checklist?	Yes

Manuscript

Click here to access/download;Manuscript;manuscript_r2.pdf

GigaScience, 20xx, 1–24

doi: xx.xxxx/xxxx
Manuscript in Preparation
Paper

PAPER

Population modeling with machine learning can enhance measures of mental health

Kamalaker Dadi[1], Gaël Varoquaux[1,2,3], Josselin Houenou[4,5], Danilo Bzdok[1,3,6], Bertrand Thirion[1] and Denis Engemann[1,7,*]

[1]Inria, CEA, Neurospin, Parietal team, Univ. Paris Saclay, 91120 Palaiseau, France and [2]Montréal Neurological Institute, McGill University, Montreal, Canada and [3]Mila - Quebec Artificial Intelligence Institute, Canada and [4]CEA, NeuroSpin, Psychiatry Team, UNIACT Lab, Univ. Paris Saclay and [5]APHP, Mondor University Hospitals, Psychiatry Dept, INSERM U955 Team 15 "Translational Psychiatry", Créteil, France and [6]Department of Biomedical Engineering, Montreal Neurological Institute, Faculty of Medicine, McGill University, Montreal, Canada and [7]Department of Neurology, Max Planck Institute for Human Cognitive and Brain Sciences, Germany

[*] denis.engemann@gmail.com

Abstract

Background Biological aging is revealed by physical measures, *e.g.*, DNA probes or brain scans. Instead, individual differences in mental function are explained by psychological constructs, e.g., intelligence or neuroticism. These constructs are typically assessed by tailored neuropsychological tests that build on expert judgement and require careful interpretation. Could machine learning on large samples from the general population be used to build proxy measures of these constructs that do not require human intervention? **Results** Here, we built proxy measures by applying machine learning on multimodal MR images and rich sociodemographic information from the largest biomedical cohort to date: the UK Biobank. Objective model comparisons revealed that all proxies captured the target constructs and were as useful, and sometimes more useful than the original measures for characterizing real-world health behavior (sleep, exercise, tobacco, alcohol consumption). We observed this complementarity of proxy measures and original measures when modeling from brain signals or sociodemographic data, capturing multiple health-related constructs. **Conclusions** Population modeling with machine learning can derive measures of mental health from brain signals and questionnaire data, which may complement or even substitute for psychometric assessments in clinical populations.

Key words: Mental Health, Proxy Measures, Machine Learning, Sociodemographic Factors, Brain Imaging

Background

Quantitative measures of mental health remain challenging despite substantial efforts [1]. The field has struggled with unstable diagnostic systems [2], small sample sizes [3], and reliance on case-control studies [4]. Perhaps most importantly, mental health cannot be measured the same way diabetes can be assessed through plasma levels of insulin or glucose. Psychological constructs, *e.g.*, intelligence or anxiety, can only be probed indirectly through lengthy expert-built questionnaires or structured examinations by a specialist. Though questionnaires often remain the best accessible option, their capacity to measure a construct is limited [5]. In practice, as full neuropsychological evaluation is not automated process but relies on expert judgement to confront multiple answers and interpret them in the context of the broader picture, such as cul-

Compiled on: August 10, 2021.
Draft manuscript prepared by the author.

Key Points

- We applied machine learning on more than 10.000 individuals from the general population to define empirical approximations of health-related psychological measures that do not require human judgment.
- We found that machine-learning enriched the given psychological measures via approximation from brain and sociodemographic data: Resulting proxy measures related as well or better to real-world health behavior than the original measures.
- Model comparisons showed that sociodemographic information contributed most to characterizing psychological traits beyond aging.

tural background of the participant. While the field of psychometrics has thoroughly studied the validity of psychological constructs and their measure [6, 7, 8], the advent of new biophysical measurements of the brain brings new promises [9, 10, 11]. The growth of biobanks and advances in machine learning open the door to large-scale validation of psychological measures for mental health research [12], and the hope to develop more generalizable models [13]. Yet, to be reliable, machine learning needs large labeled datasets [14]. Its application to learn imaging biomarkers of mental disorders is limited by the availability of large cohorts with high-quality neuropsychiatric diagnosis [15].

By comparison, it is easier to collect data on the general population without information on clinical conditions. For brain health, such data has lead to developing proxy measures that quantify biological aging [16, 17, 18, 11, 19, 20, 21, 22]. One counterintuitive aspect of the methodology is that measures of biological aging can be obtained by focusing on the age of a person, which is known in advance and in itself not interesting. Yet, by (imperfectly) predicting the age from brain data, machine-learning can capture the relevant signal. Based on a population of brain images, it extracts the *best guess* for the age of a person, indirectly positioning that person within the population. Individual-specific prediction errors therefore reflect deviations from what is statistically expected [23]. The brain of a person can look similar to the brains commonly seen in older (or younger) people. The resulting brain-predicted age reflects physical and cognitive impairment in adults [24, 17, 16] and reveals neurodegenerative processes [22, 25]. Can this strategy of biomarker-like proxy measures be extended to other targets beyond the construct of aging? Extrapolating from these successes, we propose to build upon large datasets to extend the collection of health-related *proxy measures*, probing mental traits. For this end, we focused on constructs fundamentally different in terms of content and methodology.

One high-stake target is intelligence, which is measured through socially administered tests and is one of the most extensively studied constructs in psychology. Fluid intelligence refers to the putatively culture-free, heritable and physiological component of intelligence [26, 27] and is a latent construct designed to capture individual differences in cognitive capacity. It has been robustly associated with neuronal maturation and is typically reflected in cognitive-processing speed and working-memory capacity [28]. Applied to psychiatric disorders, it may help characterize psychosis, bipolar disorder, and substance abuse [29, 30].

Neuroticism is a second promising target. As a key representative of the extensively studied Big Five personality inventory, neuroticism has a long-standing tradition in the psychology of individual differences [31, 32]. Neuroticism is measured using self-assessment questionnaires and conceptualized as capturing dispositional negative emotionality including anxiety and depressiveness [33]. It has been inter-culturally validated [26, 34] and population-genetics studies have repeatedly linked neuroticism to shared genes [35, 36, 37]. Neuroticism was shown useful in psychometric screening and supports predicting real-world behavior [38, 39].

Despite strong population-level heritability [40, 41], the link between psychological constructs, brain function and genetics is still being actively researched [42, 33]. Empowered by emerging large-scale datasets, current attempts to predict fluid intelligence or neuroticism from thousands of MRI scans argue in favor of heterogeneity and weakly generalizing effects [43, 44]. This stands in contrast to the remarkable performance obtained when predicting psychometric data from language-based inputs captured by Twitter and Facebook user data [45, 46]. As MRI acquisitions can be difficult to come by in certain populations, the promises of social-media data are appealing. However, such data may lead to measurement and selection biases difficult to control. Instead, background sociodemographic data may provide an easily accessible alternative for contextualizing the heterogeneity of psychological traits [47].

Another challenge is that psychological traits are often measured using arbitrary non-physical units, *e.g.* education degree or monthly income. In fact, society treats individual differences as categorical or continuous, depending on the practical context. While personality has been proposed to span a continuum [48], psychiatrists treat certain people as patients and not others [49]. Therefore, a measure that performs globally poorly at a continuous scale can be sufficient to distinguish subgroups as it may be informative around the boundary region between certain classes, *e.g.*, pilots who should fly and who should not. Choosing the granularity with which to gauge psychological constructs is difficult.

Confronting the promises of population phenotyping with the challenges of measuring psychological traits raises the following questions: 1) Can the success of brain age at characterizing health be extended to other proxy measures directly targeting mental constructs? 2) How well can various constructs related to mental health be approximated from general-purpose inputs not designed to measure specific latent constructs? 3) What is the relative merit of brain imaging and sociodemographics? We tackled these questions by using machine learning to craft *proxy measures* in order to approximate well-characterized *target measures* from brain-imaging and sociodemographic data. We studied age, fluid intelligence, and neuroticism. These targets have been, traditionally, considered as proxies for mental health and are fundamentally different in terms of scope and nature. Our results suggest that, as with brain age, proxy measures can bring value for the study of mental health that goes beyond approximating an available measure.

The paper is organized as follows: We first present a summary of the methodology and the workflow of building distinct proxy measures for age, fluid intelligence and neuroticism using machine learning (Figure 1). We then benchmark the proxy and the original target measures against real-world patterns of health-relevant behavior. Subsequently, through systematic model comparisons, we assess the relative contributions of brain imaging and sociodemographic data for prediction perfor-

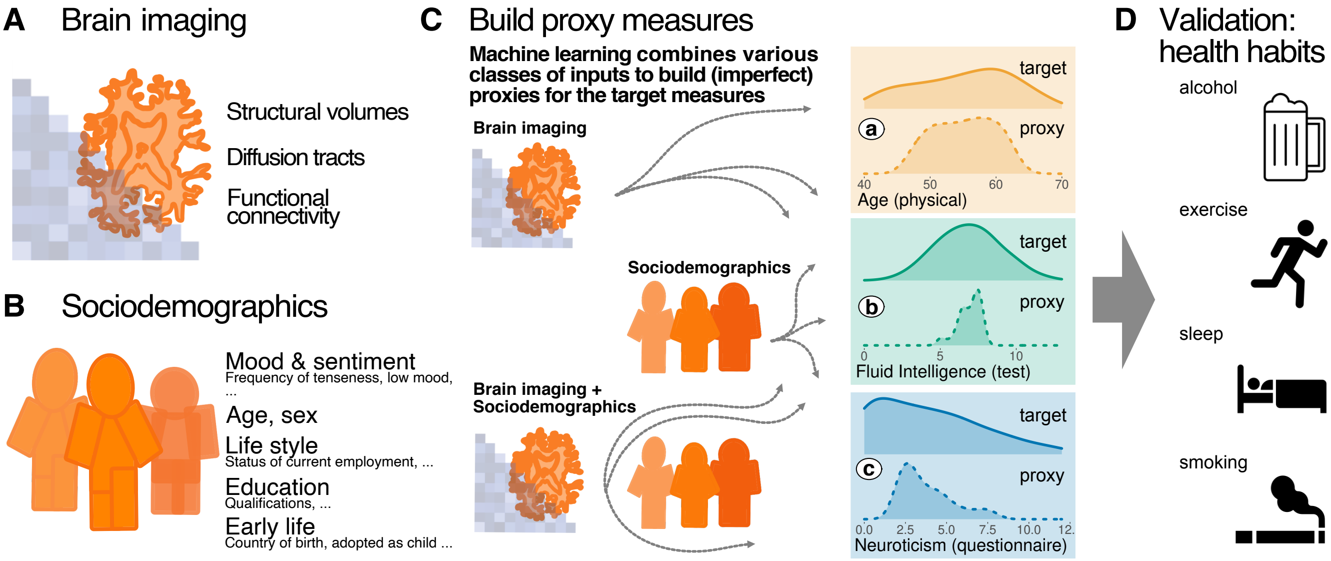

Figure 1. Methods workflow: building and evaluating proxy measures We combined multiple brain-imaging modalities (**A**) with sociodemographic data (**B**) to approximate health-related biomedical and psychological constructs (**C**), *i.e.*, brain age (accessed through prediction of chronological age), cognitive capacity (accessed through a fluid-intelligence test) and the tendency to report negative emotions (accessed through a neuroticism questionnaire). We included the imaging data from the 10 000-subjects release of the UK biobank. Among imaging data (**A**) we considered features related to cortical and subcortical volumes, functional connectivity from rfMRI based on ICA networks, and white-matter molecular tracts from diffusive directions (see Table 1 for an overview about the multiple brain-imaging modalities). We then grouped the sociodemographic data (**B**) into five different blocks of variables related to self-reported mood & sentiment, primary demographics, lifestyle, education, and early-life events (Table 2 lists the number of variables in each block). We systematically compared the approximations of all three targets based on either brain images and sociodemographics in isolation or combined (**C**) to evaluate the relative contribution of these distinct inputs. Note that proxy measures can only add to the target measures if they are not identical, *i.e.*, if the approximation of the target from the given inputs is imperfect (guaranteed in our context as the exact data generating mechanism is unknown and causally important variables remain unobserved). Using the full model (brain imaging + sociodemographics), we benchmarked complementarity of the proxy measures and the target measures with regard to real-world patterns of health behavior (**D**), i.e., the number of alcoholic beverages, exercise (metabolic equivalent task), sleep duration and the number of cigarettes smoked. Potentially additive effects between proxies and targets were gauged using multiple linear regression. Models were developed on 50% of the data (randomly drawn) based on random forest regression guided by Monte Carlo cross-validation with 100 splits (see section Model Development and Generalization Testing). We assessed generalization and health implications using the other 50% of the data as fully independent out-of-sample evaluations (see section Statistical Analysis). Learning curves suggested that this split-half approach provided sufficient data for model construction (Figure 1 – Figure supplement 1).

mance in the regression and classification settings. The complementarity between the proxy measures is, finally, discussed in the light of statistical considerations, potential data generating mechanisms, and applications for public health and clinical research.

Results: validity of proxy measures

Complementing the original measures at characterizing real-life health-related habits

To approximate age, fluid intelligence and neuroticism, we applied random-forest regression on sociodemographic data and brain images. The data was split into *validation data* for model construction (see section Model Development and Generalization Testing) and *generalization data* for statistical inference on out-of-sample predictions with independent data (see section Statistical Analysis). Our findings suggested that some information on psychological constructs can be assembled from general inputs not specifically tailored to measure these constructs, such as brain images and sociodemographic variables. The resulting proxy measures may be regarded as crude approximations of the psychological measures, but they can nonetheless capture essential aspects of the target constructs. To probe the external validity of the proxy measures, we investigated their link with real-world behavior, e.g., sleep, physical exercise, alcohol and tobacco consumption on left-out data. To probe the external validity of the proxy-measures, we investigated their link with real-world behavior, *e.g.*, sleep, physical exercise, alcohol and tobacco consumption on left-out data. To relate such health behaviors to our proxy measures, we modeled them separately as weighted sums of predicted brain-age delta, fluid intelligence and neuroticism using multiple linear regression (section Statistical Analysis). To avoid circularity, we used the out-of-sample predictions for all proxy measures (section Model Development and Generalization Testing).

The estimated regression coefficients (partial correlations), revealed complementary associations between the proxy measures and health-related behavior (Figure 2). Similar patterns arise when considering proxy measures in isolation (Figure 2 – Figure supplement 1). Compared to other proxy measures, elevated brain-age delta was associated with increased alcohol consumption (Figure 2, first row). Levels of physical exercise were consistently associated with all three predicted targets, suggesting additive effects (Figure 2, second row). For fluid intelligence, this result, counter-intuitive from the health standpoint, could imply that higher test scores reveal a more sedentary life style. Increased sleep duration consistently went along with elevated brain age delta, but lower levels of predicted neuroticism (Figure 2, third row). This may seem counter-intuitive, but is conditional on neuroticism showing a negative link with sleep duration. No consistent effect emerged for fluid intelligence. Numbers of cigarettes smoked was independently associated with all predicted targets (Figure 2, last row): Intensified smoking went along with elevated brain age delta and neuroticism but lower fluid intelligence.

The three proxy measures are difficult to compare on an equal footing as a delta was considered for brain age only (the difference between predicted and actual age) and aging-specific deconfounding was applied. The brain-age delta is indeed the standard practice, theoretically justified as age is on

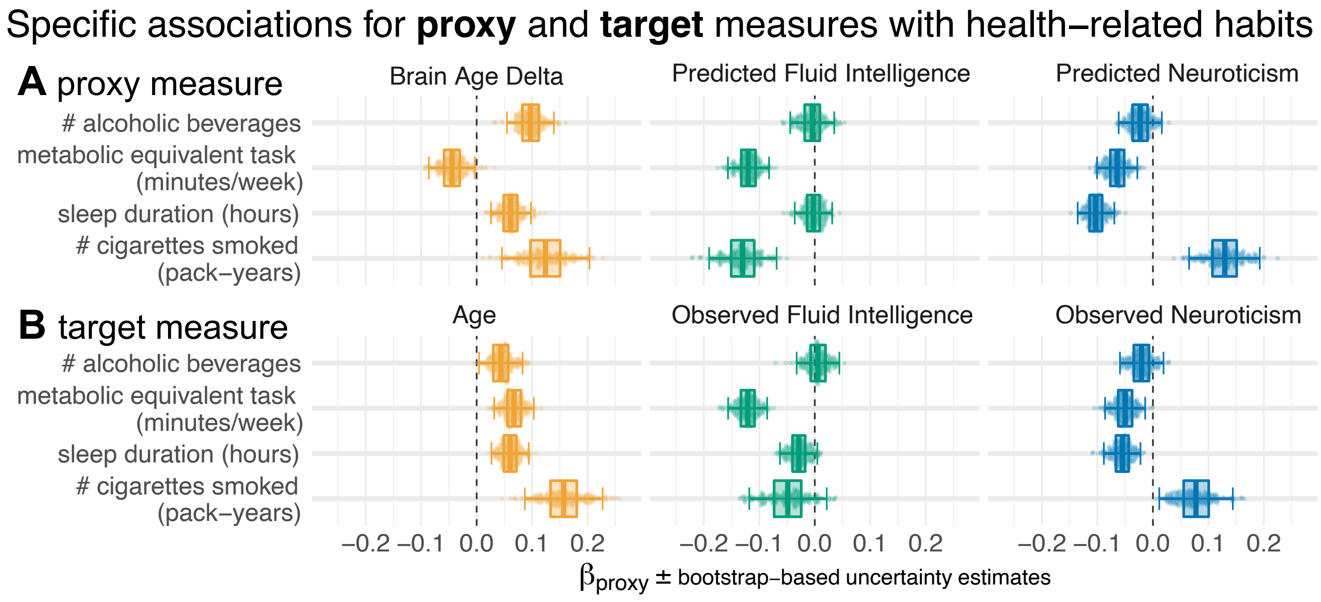

Figure 2. Proxy measures show systematic and complementary out-of-sample associations with health-related habits. We probed the external validity of all three proxy measures (brain age, fluid intelligence, neuroticism) based on a combination of brain images and all sociodemographic factors (see **Figure 1** for details). We investigated their out-of-sample associations with ecological indicators of mental health (sleep duration, time spent with physical exercise, number of alcoholic beverages and cigarettes consumed). To tease apart complementary and redundant effects, we constructed multiple linear regression models on out-of-sample predictions combining all three proxy measures **(A)**. For comparison, we repeated the analysis using the actual target measures **(B)** observed on the held-out data. Regression models are depicted rows-wise. Box plots summarize the uncertainty distribution of target-specific (color) regression coefficients with whiskers indicating two-sided 95% uncertainty intervals (parametric bootstrap). Dots illustrate a random subset of 200 out of 10 000 coefficient draws. The average coefficient estimate is annotated for convenience. At least two distinct patterns emerged: either the health outcome was specifically associated with one proxy measures (brain age delta and number of alcoholic beverages) or multiple measures showed additive associations with the outcome (*e.g.* number of pack years smoked). For target measures **(B)**, associations with health habits were often noisier or less pronounced compared to the target measures **(A)** and even a a change in direction was observed for brain age and metabolic activity. Figure 2 – Figure supplement 1 shows highly similar trends with marginal associations between proxy measures and health-related habits. Our results suggest that the proxy measures capture well health-related habits, potentially better than the original target measures, and in a complementary way across the three measures. The same patterns emerged as brain-predicted age rather than the brain age delta is used as a proxy measure (Figure 2 – Figure supplement 2). As proxy-specific deconfounding is applied, this pattern is preserved (Figure 2 – Figure supplement 3). Modeling of health-related habits jointly from proxy and target measures simultaneously revealed specific complementari. ty between proxy and target measures across multiple domains *i.e.* age, fluid intelligence, neuroticism (Figure 2 – Figure supplement 4).

a metric scale [50] for which the difference between the predicted and the measured value has a clear meaning. Such a difference is less obvious for variables with ordinal scales as implied by psychometric measures. Second, age has a pervasive influence on virtually any biomedical entity, which motivates controlling for its effect on proxy measures. To rule out that differences in proxy measures' associations to health-related behavior are driven by this methodological asymmetry, we repeated the main analysis from Figure 2, first, using the predicted age without computing the delta (Figure 2 – Figure supplement 2) and, second, introducing additional deconfounders for fluid intelligence and neuroticism (Figure 2 – Figure supplement 3). The resulting patterns were virtually unchanged, confirming that interpretations are robust.

A question that remains is whether the proxy measures bring additional value compared to the original target measures they were derived from. These original target measures showed similar associations to health behavior, with the same signs in most cases (Figure 2, B). At the same time, the ensuing patterns were more noisy, suggesting that empirically derived proxy measures yielded enhanced associations with health behavior. This inference may be difficult as differences between targets and proxies were not always easy to pinpoint visually. To implement a more rigorous statistical approach, we built comprehensive models of each respective health-related habit in which we used all proxies (predicted age, predicted fluid intelligence, predicted neuroticism) and all targets (age, fluid intelligence, neuroticism) simultaneously as predictors (Figure 2 – Figure supplement 4). The results show systematic additive effects of proxies and targets across the three target domains and the four health-habits. These trends are well-captured by the hypothesis tests of the respective linear models (Table S3). As targets and proxies may be systematically intercorrelated, multicollinearity may corrupt these inferences. Inspection of variance inflation factors (VIF)— a measure that reveals how well a given predictor can be approximated by a linear combination of the other predictors— argued in favor of low to moderate levels of multicollinearity (Table S4). Indeed, all VIF values fell between 3 and 1, whereas, classically, values above 5 or 10 are considered as thresholds [51] for pathological collinearity. This suggests that the model inferences are statistically sound.

The relative importance of brain and sociodemographic data depends on the target

In a second step, we investigated the relative performance of proxy measures built from brain signals and distinct sociodemographic factors for the three targets: age, fluid intelligence and neuroticism. Among the sociodemographic variables there was one block for each target explaining most of the prediction performance (Figure 3, dotted outlines). Combining all sociodemographic variables did not lead to obvious enhancements (Figure 3 – Figure supplement 2). For age prediction, variables related to current life-style showed by far the highest performance. For fluid intelligence, education performed by far best. For neuroticism, mood & sentiment clearly showed the strongest performance.

Combining MRI and sociodemographics, enhanced age prediction systematically on all four blocks of variables (Figure 3

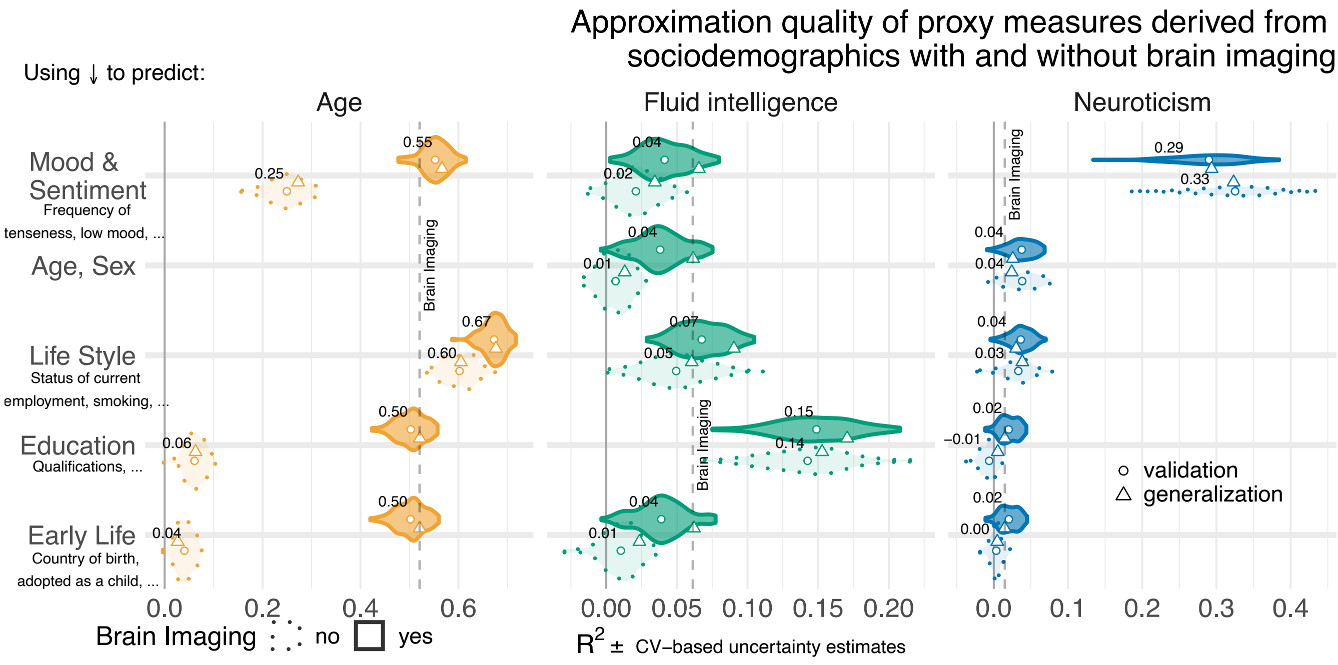

Figure 3. Approximation performance of proxy measures derived from sociodemographic data and MRI. We report the R^2 metric to facilitate comparisons across prediction targets. The cross-validation (CV) distribution (100 Monte Carlo splits) on the validation dataset is depicted by violins. Drawing style indicates whether brain imaging (solid outlines of violins) was included in addition or not (dotted outlines of violins). Dots depict the average performance on the validation data across CV-splits. Pyramids depict the performance of the average prediction (CV-bagging) on held-out generalization datasets. For convenience, the mean performance on the validation set is annotated for each plot. Vertical dotted lines indicate the average performance of the full MRI model. The validation and held-out datasets gave similar picture of approximation performance with no evidence for cross-validation bias [52]. For the averaged out-of-sample predictions, the probability of the observed performance under the null-distribution and the uncertainty of effect sizes were formally probed using permutation tests and bootstrap-based confidence intervals (Table S1). Corresponding statistics for the baseline performance of models solely based on brain imaging (vertical dotted lines) are presented in Table S5. Figure 3 – Figure supplement 1 shows approximation results based on MRI. Figure 3 – Figure supplement 2 presents results based on all sociodemographic factors.

solid outlines, and Table S1). The benefit of brain-imaging features was less marked for prediction of fluid intelligence or neuroticism. With fluid intelligence, brain-imaging data improved the performance statistically significantly for all models, yet, with small effect sizes (Table S1). For neuroticism, no systematic benefit of including brain images alongside sociodemographics emerged (Table S1, bottom row). Nevertheless, brain data was sufficient for statistically significant approximation of the target measures in all three targets (Table S5).

Psychological measures often come without physical scales and units [50]. In practice, clinicians and educators use them with specific thresholds for decision making. To investigate empirically-defined proxy measures beyond continuous regression, we performed binary classification of extreme groups obtained from discretizing the targets using the 33_{rd} and 66_{th} percentiles, following the recommendations by Gelman and Hill 2006 regarding discrete variable encoding strategies. Furthermore, we measured accuracy with the area under the classification accuracy curve (AUC) which is only sensitive to ranking, ignoring the scale of the error. Classification performance visibly exceeded the chance level (AUC > 0.5) for all models (Figure 4) and approached or exceeded levels considered practically useful (AUC > 0.8) [49]. Across proxy measures, models including sociodemographics performed best but the difference between purely sociodemographic and brain-based models was comparably weak, at the order of 0.01-0.02 AUC points (Table S2). Using brain data only led to worse performance, yet, still better than chance as revealed by permutation testing (Table S6).

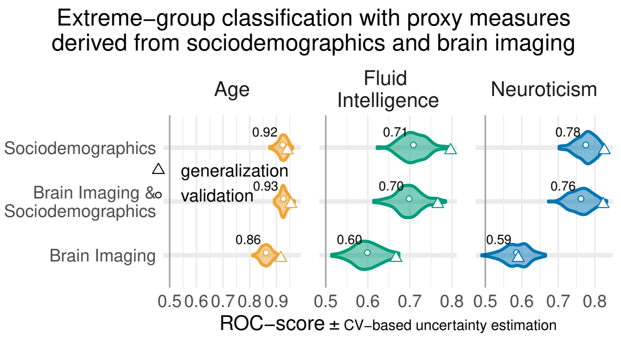

Figure 4. Classification analysis from imaging, sociodemographics and combination of both data. For classification of extreme groups instead of continuous regression, we split the data into low vs high groups based on 33_{rd} and 66_{th} percentiles. Visual conventions follow Figure 3. We report the accuracy in AUC. Models including sociodemographics performed visibly better than models purely based on brain imaging. Differences between brain-imaging and sociodemographics appeared less pronounced as compared to the fully-fledged regression analysis. For the average out-of-sample predictions, the probability of the observed performance under the null-distribution and the uncertainty of effect sizes were formally probed using permutation tests and bootstrap-based confidence intervals (Table S2). Corresponding statistics for the baseline performance of models solely based on brain imaging (vertical dotted lines) are presented in Table S6. Overall, when moving from the more difficult full-scale regression problem to extreme-group classification problem with purely ranking-based scores, the relative differences between brain-based and sociodemographics-based prediction gradually faded away.

Discussion

Guided by machine learning, we empirically derived proxy measures that combine multiple sources of information to capture

extensively validated target measures from psychology. These proxy measures all showed complementary associations with real-world health indicators beyond the original targets. The combination of brain imaging and target-specific sociodemographic inputs often improved approximation performance.

Empirically-derived proxy measures: validity and practical utility

In our study, construct validity [6, 54, 7] of the corresponding proxy measures was supported by the gain in prediction performance brought by specific sociodemographic factors (Figure 3). Association with health-relevant habits added external validity to the proxy measures (Figure 2). The complementary patterns related to traditional construct semantics: High consumption of cigarettes is associated with neuroticism [55], excessive drinking may lead to brain atrophy and cognitive decline [56] – both common correlates of elevated brain age [22, 57].

Can our empirically-derived proxy measures, thus, substitute for specific psychometric instruments? A mental-health professional may still prefer an established routine for clinical assessment, relying on interviews and personality questionnaires with implicit experience-based thresholds. Inclusion of brain imaging may even seem to yield diminishing returns when approximating high-level psychological traits. Yet, it could simply be a matter of time until more effective acquisition protocols will be discovered alongside useful signal representations. Including brain imaging, rather seems a “safe bet” as machine learning is often capable of selecting relevant inputs [11, 58] and costs of MRI-acquisition can be amortized by clinical usage. Empirically-derived proxy measures may open new doors where tailored assessment of latent constructs is not applicable due to lack of specialized mental-health workforce or sheer cost.

Constructs of mental-health can be accessed from general-purpose data

Brain age has served as landmark in this study. It has been arguably the most discussed candidate for a surrogate biomarker in the brain imaging literature [16, 17, 24]. With mean absolute errors around 4 years, up to 67% variance explained, and AUC-scores up to 0.93 in the classification setting, our results compare favorably to the recent brain-age literature within the UK Biobank [19, 59] and in other datasets [22, 11], though we relied on off-the-shelf methods and not custom deep learning methods [60]. Applying the same approach to psychological constructs (fluid intelligence, neuroticism), we found that approximation from brain imaging data or sociodemographic descriptors was generally harder.

It is important to recapitulate that approximation quality on these differently measured targets has a different meaning. Age is measured with meaningful physical units (years) on a ratio scale [50] (Selma is *twice as old* as Bob). Psychometric scores are unit-free, which may provoke ambiguity regarding the level of measurement [54]. Their implied scales may be considered as interval (the *difference between* Bob’s and Selma’s intelligence is -0.1 standard deviations) if not ordinal (Bob’s intelligence was *ranked below* Selma’s) [50]. In day-to-day psychological practice, these scores are often used via practically-defined thresholds, *e.g.* school admission or pilot candidate selection [61, 62]. In the classification setting, all proxy measures approached or exceeded a performance of 0.80 deemed relevant in biomarker development [49], though to be fair, they approximated established psychometric targets (proxy measures themselves) and not a medical condition. Different proxy measures should, thus, be subjected to different standards, depending on the granularity of the implied measurement scale.

A more complete view on how the proxy measures capture mental-health constructs emerges from their associations with real-world behavior (Figure 2). Indeed, the associations with proxy measures (Figure 2 **B**) were less noisy and more consistent then with the target measures (Figure 2 **A**), regardless of their approximation quality. This may seem surprising at first, but the target measures are themselves noisy and of imperfect validity. These measures correspond to traditional tests which, in practice, must be interpreted by an expert, actively confronting their output with broader information on the individual. For instance, IQ scores are typically normalized across age groups. However, extending such a normalization approach to many factors (socio-economic status, culture, gender) poses fundamental high-dimensional statistics challenges. Conversely, using machine learning to assemble proxy measures by mapping the targets to rich sociodemographic and brain data implicitly contextualizes them. In this respect, the resulting measures capture more general signal than the original tests. Here, machine learning could be seen as mimicking the work of a mental health expert who carefully compares psychometric results with other facts known about an individual and its reference population.

The benefits offered by brain data depend on the target construct

All brain-derived approximations were statistically meaningful. Yet, only for age prediction, imaging data by itself led to convincing performance. For fluid intelligence and neuroticism, sociodemographic factors were the most important determinants of prediction success. The best-performing sociodemographic models were based on inputs semantically close to these targets, *i.e.*, education details or mood & sentiment. While those results support construct validity, they may come with a certain risk of circularity. The causal role of those predictors is not necessarily clear as better educational attainment is heritable itself [63] and may reinforce existing cognitive abilities. Similarly, prolonged emotional stress due to life events may exacerbate existing dispositions to experience negative emotions captured by neuroticism [64], traits which commonly help accumulate stressful life events [38]. Nevertheless, for fluid intelligence but not neuroticism, brain imaging added incremental value when combined with various sociodemographic predictors. This may suggest that the cues for neuroticism conveyed by brain imaging were already present in sociodemographic predictors, hinting at common causes. Off note, in the specific context of aging, the empirical distinction between brain age and cognitive age is reflecting a similar intuition [65].

Limitations

Additional constructs and psychometric tools could have been visited. The broader construct of intelligence is often estimated using a general factor model with multiple correlated tests. While this is obviously useful for normative assessments, measures of fluid intelligence can also serve a situational fitness signal [30]. There is a wealth of questionnaires for measuring negative emotionality and neuroticism, specifically. Yet, we could only study the EPQ scale provided by the UK Biobank. A complementary approach would be to estimate latent factors by pooling all non-imaging data semantically related to neuroticism [66]. Here, we considered established target measures “as is”, instead of derivatives.

It terms of mental-health research, this study falls short of

directly testing the clinical relevance of estimated proxy measures. Even in a very large general-population cohort such as the UK Biobank, there are only a few hundred diagnosed cases of mental disorders (ICD-10 mental-health diagnoses from the F chapter) with brain-imaging data available. As a result, we could not directly assess the performance of proxy measures in clinical populations. The low number of diagnosed mental disorders in UK Biobank highlights the practical importance of studying mental health as a continuous, in addition to diagnosed conditions. Indeed, a public health perspective calls for targeting individual differences in health, not only pathology. Psychological constructs such as IQ and neuroticsm are important factors of the epidemiology of psychiatric disorders [38, 30, 29, 67], and accelerated brain aging is associated with various neurological conditions [18, 17, 25]. Yet, few cohorts come with extensive neuropsychological testing. Validated proxies of these constructs open the door to including them in epidemiological studies as secondary outcomes or additional explanatory variables.

Conclusion: Proxy measures may enhance the validity of constructs gauging mental health

In population studies of mental health, individual traits are captured via lengthy assessments, tailored to specific brain and psychological constructs. We have shown that proxy measures built empirically from general-purpose data can capture these constructs and can improve upon traditional measures when studying real-world health patterns. Proxy measures can make psychological constructs available to broader, more ecological studies building on large epidemiological cohorts or real-world evidence. This can make the difference where psychological constructs are central to developing treatment and prevention strategies, but direct measures have not been collected.

Methods

To facilitate reproduction, understanding, and reuse, we have made all data analysis and visualization source code available on Github [68].

Dataset

The United Kingdom Biobank (UKBB) database is to date the most extensive large-scale cohort aimed at studying the determinants of the health outcomes in the general adult population. The UKBB is openly accessible and has extensive data acquired on 500 000 individuals aged 40-70 years covering rich phenotypes, health-related information, brain-imaging and genetic data [12]. Participants were invited for repeated assessments, some of which included MR imaging. For instance, cognitive tests that were administered during an initial assessment were also assessed during the follow-up visits. This has enabled finding for many subjects at least one visit containing all heterogeneous input data needed to develop the proposed proxy measures. The study was conducted using the UKBB Resource Application 23827.

Participants

All participants gave informed consent. The UKBB study was examined and approved by the North West Multi-centre Research Ethics Committee. We considered participants who have responded to cognitive tests, questionnaires, and have access to their primary demographics and brain images [69]. Out of the total size of UKBB populations, we found 11 175 participants who had repeated assessments overlapping with the first brain imaging release [70]. Note that the features (sociodemographic variables) that we included in the analysis are measures that are self-reported during a follow-up imaging visit. The demographics are 51.6% female (5 572) and 48.3% male (5 403), and an age range between 40-70 years (with a mean of 55 years and standard deviation of 7.5 years). The data for model training were selected using a randomized split-half procedure yielding 5 587 individuals. The remaining subjects were set aside as a held-out set for generalization testing (see section Model development and generalization testing). We made sure that the subjects used for model training and generalization testing were strictly non-overlapping.

Learning curves documented that the training split was sufficiently large for constructing stable prediction models Figure 1 – Figure supplement 1 with profiles of performance similar to latest benchmarks on model complexity in the UK Biobank [71]. Moreover, simulations and empirical findings suggest that larger testing sets are more effective at mitigating optimistic performance estimates [72, 52]. Together, this provided a pragmatic solution to the inference-prediction dilemma [58, 73] given the two objectives of the present investigation to obtain reasonably good predictive models, while at the same time performing parameter inference of statistical models developed on the left-out data.

To establish specific comparisons between models based on sociodemographics, brain data or their combinations we exclusively considered the cases for which MRI scans were available. The final sample sizes used for model construction and generalization testing then depended on the availability of MRI: For age and fluid intelligence, our randomized split-half procedure (see section Model development and generalization testing) yielded 4203 cases for model building and 4157 for generalization. For cases with valid neuroticism assessment, fewer brain images were available, which yielded 3550 cases for model building and 3509 for generalization.

Data acquisition

Sociodemographic data (non-imaging) was collected with self-report measures administered through touchscreen questionnaires, complemented by verbal interviews, physical measures, biological sampling and imaging data. MRI data were acquired with the Siemens Skyra 3T using a standard Siemens 32-channel RF receiver head coil [74]. We considered three MR imaging modalities as each of them potentially captures unique neurobiological details: structural MRI (sMRI/T1), resting-state functional MRI (rs-fMRI) and diffusion MRI (dMRI). For technical details about the MR acquisition parameters, please refer to [70]. We used image-derived phenotypes (IDPs) of those distinct brain-imaging modalities, as they provide actionable summaries of the brain measurements and encourage comparability across studies.

Target measures

As our target measures for brain age modeling, we use an individual's age at baseline recruitment (UKBB code "21022-0.0"). Fluid intelligence, was assessed using a cognitive battery designed to measure an individual's capacity to solve novel problems that require logic and abstract reasoning. In the UK Biobank, the fluid intelligence test (UKBB code "20016-2.0") comprises thirteen logic and reasoning questions that were administered via the touchscreen to record a response within two minutes for each question. Therefore, each correct answer is

scored as one point with 13 points in total[1]. Neuroticism (UKBB code "20127-0.0") was measured using a shorter version of the revised Eysenck Personality Questionnaire (EPQ-N) comprised of 12-items [32]. Neuroticism was assessed during Biobank's baseline visit. The summary of the individual's scores ranges from 0 to 12 that assess dispositional tendency to experience negative emotions [2].

In the course of this work, a question that emerged concerned the size of the gap between age at baseline recruitment and MRI-scan time and its potential impact on the analysis. Supplementary checks indicated that the age gap was at least 5 years for most participants. Yet, from a statistical perspective, the two age measures turned out highly interchangeable (Figure S2) and global conclusions remained unchanged (Figure S3).

Sociodemographic data

In this work, we refer to non-imaging variables broadly as sociodemographics excluding the candidate targets fluid intelligence and neuroticism. To approximate latent constructs from sociodemographics, we included 86 non-imaging inputs (Table S7) which are the collection of variables reflecting each participant's demographic and social factors *i.e.*, sex, age, date and month of birth, body mass index, ethnicity, exposures at early life –*e.g.* breast feeding, maternal smoking around birth, adopted as a child– education, lifestyle-related variables –*e.g.* occupation, household family income, household people living at the same place, smoking habits–, and mental-health variables. All these data were self-reported. We then assigned these 86 variables to five groups based on their relationships. Based on our conceptual understanding of the variables, we name assigned them to one out of five groups: **1)** mood & sentiment, **2)** primary demographics as age, sex, **3)** lifestyle, **4)** education, **5)** early life. We then investigated the intercorrelation between all 86 variables to ensure that the proposed grouping is compatible with their empirical correlation structure Figure S1.

The sociodemographic groups had varying amounts of missing data. For *e.g.* the source of missingness is concerned with the participants lifestyle habits such as smoking and mental health issues [77]. To deal with this missingness in the data using imputation [78], we used column-wise replacement of missing information with the median value calculated from the known part of the variable. We subsequently included an indicator for the presence of imputed for down-stream analysis. Such imputation is well suited to predictive models [79].

Image processing to derive phenotypes for machine learning

MRI data preprocessing were carried out by UKBB imaging team. The full technical details are described elsewhere [70, 74]. Below, we describe briefly the custom processing steps that we used on top of the already preprocessed inputs.

Structural MRI

This type of data analysis on T1-weighted brain images are concerned with morphometry of the gray matter areas *i.e.* the quantification of size, volume of brain structures and tissue types and their variations under neuropathologies or behavior [80]. For example, volume changes in gray matter areas over lifetime are associated with: brain aging [81], general intelligence [82] and brain disease [83]. Such volumes are calculated within pre-defined ROIs composed of cortical and sub-cortical structures [84] and cerebellar regions [85]. We included 157 sMRI features consisting of volume of total brain and grey matter along with brain subcortical structures[3]. All these features are pre-extracted by UKBB brain imaging team [70] and are part of data download. We concatenated all inputs alongside custom-built fMRI features for predictive analysis (feature union).

Diffusion weighted MRI

Diffusion MRI enables to identify white matter tracts along principal diffusive direction of water molecules, as well as the connections between different gray matter areas [88, 89]. The study of these local anatomical connections through white matter are relevant to the understanding of neuropathologies and functional organization [90]. We included 432 dMRI skeleton features of FA (fractional anisotropy), MO (tensor mode) and MD (mean diffusivity), ICVF (intra-cellular volume fraction), ISOVF (isotropic volume fraction) and OD (orientation dispersion index) modeled on many brain white matter structures extracted from neuroanatomy[4]. For extensive technical details, please refer to [92]. The skeleton features we included were from category134 shipped by the UKBB brain-imaging team and we used them without modification.

Functional MRI

Resting-state functional MR images capture low-frequency fluctuations in blood oxygenation that can reveal ongoing neuronal interactions in time forming distinct brain networks [93]. Functional connectivity within these brain network can be linked to clinical status [94], to behavior [70], or to psychological traits [44]. We also included resting-state connectivity features based on the time-series extracted from Independent Component Analysis (ICA) with 55 components representing various brain networks extracted on UKBB rfMRI data [70]. These included the default mode network, extended default mode network and cingulo-opercular network, executive control and attention network, visual network, and sensorimotor network. We measured functional connectivity in terms of the between-network covariance. We estimated the covariance matrices using Ledoit-Wolf shrinkage [95]. To account for the fact that covariance matrices live on a particular manifold, *i.e.*, a curved non-Euclidean space, we used the tangent-space embedding to transform the matrices into a Euclidean space [96, 97] following recent recommendations [98, 99]. For predictive modeling, we then vectorized the covariance matrices to 1 485 features by taking the lower triangular part. These steps were performed with `NiLearn` [100].

Comparing predictive models to approximate target measures

Imaging-based models

First, we focused on purely imaging-based models based on exhaustive combinations of the three types of MRI modalities (see Table 1 for an overview). This allowed us to study potential overlap and complementarity between the MRI-modalities. Preliminary analyses revealed that combining all MRI data gave reasonable results with no evident disadvantage over particular

1 A complete overview of the 13 individual fluid intelligence items is provided by the dedicated user manual [75]
2 For a complete list of Neuroticism questionnaires is provided by the dedicated field descriptions and derivation for variables related to bipolar disorder, major depression status and neuroticism score [76]

3 Regional grey matter volumes [86] Subcortical volumes [87]
4 Diffusion-MRI skeleton measurements [91]

Table 1. Imaging-based models.

Index	Name	# variables	# groups
1	brain volumes (sMRI)	157	1
2	white matter (dMRI)	432	1
3	functional connectivity (fMRI)	1485	1
4	sMRI, dMRI	589	2
5	sMRI, fMRI	1642	2
6	dMRI, fMRI	1917	2
7	sMRI, dMRI, fMRI (full MRI)	2074	3

Table 2. Non-imaging baseline models or sociodemographic models based on single group. Variables in each group are described at corresponding section sociodemographic data

Index	Name	# variables
1	Mood & Sentiment (MS)	25
2	Age, Sex (AS)	5
3	Life style (LS)	45
4	Education (EDU)	2
5	Early Life (EL)	9

Table 3. Random forest hyperparameters and tuning with grid search (5 fold cross-validation).

Hyperparameter	Values
Impurity criterion	Mean squared error
Maximum tree depth	5, 10, 20, 40, full depth
Fraction of features for split	1, 5, “log2”, “sqrt”, “complete”
Number of trees	250

combinations of MRI modalities (Figure 3 – Figure supplement 1), hence, for simplicity, we only focused on the full MRI model in subsequent analyses.

Sociodemographic models

We composed predictive models based on non-exhaustive combinations of different types of sociodemographic variables. To investigate the relative importance of each class of sociodemographic inputs, we performed systematic model comparisons. We were particularly interested in studying the relative contributions of early-life factors as compared to factors related to more recent life events such as education as well as factors related to current circumstances such as mood & sentiment and life-style. The resulting models based on distinct groups of predictors are listed in Table 2 (for additional details see Table S7 and Figure S1).

Combined imaging and sociodemographic models

In the next step, we were interested in how brain-related information would interact within each of these sociodemographic models. For example, information such as the age of an individual, or the level of education, may add important contextual information to brain images. We therefore considered an alternative variant for each of the models in Table 2 that included all MRI-related features (2 074 additional features) as described at section image processing to derive phenotypes for machine learning.

Predictive model

Linear models are recommended as default choice in neuroimaging research [98, 101] especially when datasets include fewer than 1000 data points. In this study approximated targets generated by distinct underlying mechanisms based on

Table 4. Number of samples for classification analysis (N).

# groups	Age	Fluid intelligence	Neuroticism
1	1335	1108	1054
2	1200	898	1020

multiple classes of heterogenous input data with several thousands of data points. We hence chose the non-parametric random forest algorithm that can be readily applied on data of different units for non-linear regression and classification [102] with mean squared error as impurity criterion. To improve computation time we fixed tree-depth to 250 trees, a hyper-parameter that is not usually not tuned but set to a generous number as performance plateaus beyond a certain number of trees [103, ch. 15]. Preliminary analyses suggested that additional trees would not have led to substantial improvements in performance. We used nested cross-validation (5-fold grid search) to tune the depth of the trees as well as the number of variables considered for splitting (see Table 3 for a full list of hyper-parameters considered).

Classification analysis. We also performed classification analysis on the continuous targets. Adapting recommendations from Gelman and Hill [53], we performed discrete variable encoding of the targets leading to extreme groups based on the 33_{rd} and 66_{th} percentiles (see Table 4 for the number of classification samples per group). This choice avoids including samples near the average outcome for which the input data may be indistinct. We were particularly interested in understanding whether model performance would increase when moving toward classifying extreme groups. For this analysis, we considered all three types of models (full MRI 2074 features from imaging-based models, all sociodemographics variables, total 86 variables see section, combination of full MRI and all sociodemographics, a total 2160 variables see section (See section Comparing predictive models to approximate target measures). When predicting age, we excluded the age & sex sociodemographic block from all sociodemographic variables which then yielded a total of 81 variables. To assess the performance for classification analysis, we used the area under the curve (AUC) of the receiver operator characteristic (ROC) as an evaluation metric [101].

Model development and generalization testing

Before any empirical work, we generated two random partitions of the data, one validation dataset for model construction and one held-out generalization dataset for studying out-of-sample associations using classical statistical analyses.

For cross-validation, we then subdivided the validation set into 100 training- and testing splits following the Monte Carlo resampling scheme (also referred to as shuffle-split) with 10% of the data used for testing. To compare model performances based on paired tests, we used the same splits across all models. Split-wise testing performance was extracted and carried forward for informal inference using violin plots (Figure 3,Figure 4). For generalization testing, predictions on the held-out data were generated from all 100 models from each cross-validation split.

On the held-out set, unique subject-wise predictions were obtained by averaging across folds and occasional duplicate predictions due to Monte Carlo sampling which could produce multiple predictions per subject[5]. Such strategy is known as

5 We ensured prior to computation that with 100 CV-splits, predictions were available for all subjects.

CV-bagging [104, 105] and can improve both performance and stability of results[6]. The resulting averages were reported as point estimates in Figures 3,4, and 3 – Figure supplement 1 and used as proxy measures in the analysis of health-related behaviors Figure 2.

Statistical analysis

Resampling statistics for model comparisons on the held-out data

To assess the statistical significance of the observed model performance and the differences in performance between the models, we computed resampling statistics of the performance metrics on the held-out generalization data not used for model construction [106]. Once unique subject-wise predictions were obtained on the held-out generalization data by averaging the predictions emanating from each fold of the validation set (cv-bagging), we computed null- and bootstrap-distributions of the observed test statistic on the held-out data, i.e., R^2 score for regression and *AUC* score for classification.

Baseline comparisons. To obtain a p-value for baseline comparisons (*could the prediction performance of a given model be explained by chance?*) on the held-out data, we permuted targets 10 000 times and then recomputed the test statistic in each iteration. P-values were then defined as the probability of the test statistic under null distribution being larger than the observed test statistic. To compute uncertainty intervals, we used bootstrap, recomputing the test statistic after resampling 10 000 times with replacement and reporting the 2.5 and 97.5 percentiles of the resulting distribution.

Pairwise comparisons between models. For model comparisons, we considered the out-of-sample difference in R^2 or *AUC* between any two models. To obtain a p-value for model comparisons (*could the difference in prediction performance between two given models be explained chance?*) on the held-out data, we permuted the scores predicted by model A and model B for every single prediction 10 000 times and then recomputed the test statistic in each iteration. We omitted all cases for which only predictions from one of the models under comparison was present. P-values were then defined as the probability of the absolute of the test statistic under null distribution being larger than the absolute observed test statistic. The absolute was considered to account for differences in both directions. Uncertainty intervals were obtained from computing the 2.5 and 97.5 percentiles of the bootstrap distribution based on 10 000 iterations. Here, predictions from model A and model B were resampled using identical resampling indices to ensure a meaningful paired difference.

Out-of-sample association between proxy measures and health-related habits

Computation of brain age delta and de-confounding. For association with health-contributing habits (Table 5), we computed the brain age delta as the difference between predicted age and actual age:

$$BrainAge\Delta = Age_{predicted} - Age \tag{1}$$

As age prediction is rarely perfect, the residuals will still contain age-related variance which commonly leads to brain age bias when relating the brain age to an outcome of interest, *e.g.*, sleep duration [107]. To mitigate leakage of age-related information into the statistical models, we employed a de-confounding procedure in line with [108] and [11, eqs. 6-8] consisting in residualizing a measure of interest (*e.g.* sleep duration) with regard to age through multiple regression with quadratic terms for age. To minimize computation on the held-out data, we first trained a model relating the score of interest to age on the validation set to then derive a de-confounding predictor for the held-out generalization data. The resulting de-confounding procedure for variables in the held-out data amounts to computing an age-residualized predictor $measure_{resid}$ from the measure of interest (*e.g.* sleep duration) by applying the following quadratic fit on the validation data:

$$\begin{aligned} measure_{validation} = age_{validation} \times \beta_{val1} + \\ age^2_{validation} \times \beta_{val2} + \epsilon \end{aligned} \tag{2}$$

The de-confounding predictor was then obtained by evaluating the weights β_{val1} and β_{val2} obtained from Equation 2 on the generalization data:

$$\begin{aligned} deconfounder = age_{generalization} \times \beta_{val1} \\ + age^2_{generalization} \times \beta_{val2} \end{aligned} \tag{3}$$

We performed this procedure for all target measures, to study associations not driven by the effect of age. For supplementary analyses presented in figure Figure 2 – Figure supplement 3, the same procedure was applied, substituting age for fluid intelligence and neuroticism, respectively.

Health-related habits regression. We then investigated the joint association between proxy measures of interest and health-related habits (Table 5) using multiple linear regression. For simplicity, we combined all brain imaging and all sociodemographics variables (Figure 3, Figure 3 – Figure supplement 1, Figure 3 – Figure supplement 2). The ensuing model can be denoted as

$$\begin{aligned} measure = deconfounder \times \beta_1 + BrainAge\Delta \times \beta_2 \\ + PredFluidInt \times \beta_3 + PredNeurot \times \beta_4 + \epsilon, \end{aligned} \tag{4}$$

where *deconfounder* is given by Equation 2. Prior to model fitting, rows with missing inputs were omitted. For comparability, we then applied standard scaling on all outcomes and all predictors.

The parametric bootstrap was a natural choice for uncertainty estimation, as we used standard multiple linear regression which provides a well defined procedure for mathematically quantifying its implied probabilistic model. Computation was carried out using `sim` function from the `arm` package as described in [53, Ch.7,pp.142-143]. This procedure can be intuitively regarded as yielding draws from the posterior distribution of the multiple linear regression model under the assumption of a uniform prior. For consistency with previous analyses, we computed 10000 draws.

For supplementary analysis in Figure 2 – Figure supplement 2, the brain-predicted age instead of the delta was used:

$$\begin{aligned} measure = deconfounder \times \beta_1 + BrainAge \times \beta_2 + \\ PredFluidInt \times \beta_3 + PredNeurot \times \beta_4 + \epsilon, \end{aligned} \tag{5}$$

For supplementary analysis in Figure 2 – Figure supplement

6 The use of CV-bagging can explain why on figures 3,4, and 3 – Figure supplement 1 the performance was sometimes slightly better on the held-out set compared to the cross-validation on the validation test.

Table 5. Extra health variables used for correlation analysis with subject-specific predicted scores.

Family	eid	Variables
Alcohol*	1568-0.0	Average weekly red wine intake
	1578-0.0	Average weekly champagne plus white wine intake
	1588-0.0	Average weekly beer plus cider intake
	1598-0.0	Average weekly spirits intake
	1608-0.0	Average weekly fortified wine intake
	5364-0.0	Average weekly intake of other alcoholic drinks
Physical activity	22040-0.0	Summed MET minutes per week for all activity
Smoking	20161-0.0	Pack years of smoking
Sleep	1160-0.0	Sleep duration

*We computed a compound drinking score by summing up all variables from the alcohol family

3, additional deconfounders were introduced.

$$measure = deconfounder_{age} \times \beta_1 + BrainAge \times \beta_2 + deconfounder_{FI} \times beta_3 + PredFluidInt \times \beta_4 + deconfounder_N + \beta_5 + PredNeurot \times \beta_6 + \epsilon, \quad (6)$$

where $deconfounder_{FI}$ is the deconfounder for fluid intelligence and $deconfounder_N$ the deconfounder for neuroticsm following the procedure described in Equation 2 and Equation 3.

For supplementary analysis in Figure 2 – Figure supplement 4, proxies and targets were analyzed simultaneously.

$$measure = Age \times \beta_1 + BrainAge \times \beta_2 + FluidIntelligence \times beta_3 + PredFluidInt \times \beta_4 + Neuroticism + \beta_5 + PredNeurot \times \beta_6 + \epsilon, \quad (7)$$

Software

Preprocessing and model building were carried out using Python 3.7. The `NiLearn` library was used for processing MRI inputs [100]. We used the *scikit-learn* library for machine learning [109]. For statistical modeling and visualization we used the R-language [110] (version 3.5.3) and its ecosystem: `data.table` for high-performance manipulation of tabular data, `ggplot` [111, 112] for visualization and the `arm` package for parametric bootstrapping [113]. All data analysis code is shared on GitHub [68].

Availability of source code and requirements

- Project name: "empirical_proxy_measures"
- Project home page: [68]
- Operating system(s): e.g. Platform independent
- Programming language: e.g. Python and R
- Other requirements: e.g. Python 3.6.8 or higher, R 3.4.3 or higher
- License: BSD-3

Availability of supporting data and materials

The data supporting the results and figures of this article is available in the "empirical_proxy_measures" repository [68]. The input data is publicly available (controlled access) via the UK Biobank website [114]. The procedure to apply for access [115] requires registering with the UK Biobank and compiling an application form detailing:

- A summary of the planned research
- The UK Biobank data fields required for the project
- A description of derivatives (data, variables) generated by the project

Declarations

Author's Contributions (alphabetic order)

- **Conceptualization**: BT, DB, DE, GV, JH
- **Data curation**: DB, KD
- **Software**: BT, DE, GV, KD
- **Formal analysis**: DE, GV, KD
- **Supervision**: BT, DE, GV
- **Funding acquisition**: GV, JH
- **Validation**: DE, KD
- **Investigation**: DE, KD
- **Visualization**: DE, GV, KD
- **Methodology**: BT, DE, GV
- **Project administration**: DE, GV
- **Writing - original draft**: DE, KD
- **Writing - review and editing**: DB, BT, DE, GV, JH, KD

Acknowledgements

We would like to thank Dr. Stefania de Vito and Dr. Benjamin de Haas for the critical review and helpful discussion of previous versions of the manuscript. We would like to thank Dr. Julien Dubois and Prof. Ralph Adolphs for helpful discussions in the course of this research project.

References

1. Kapur S, Phillips AG, Insel TR. Why has it taken so long for biological psychiatry to develop clinical tests and what to do about it? Molecular Psychiatry 2012;17(12):1174–1179.
2. Insel T, Cuthbert B, Garvey M, Heinssen R, Pine DS, Quinn K, et al. Research Domain Criteria (RDoC): Toward a New Classification Framework for Research on Mental Disorders. American Journal of Psychiatry 2010;167(7):748–751.
3. Szucs D, Ioannidis JP. Empirical assessment of published effect sizes and power in the recent cognitive neuroscience and psychology literature. PLoS biology 2017;15(3):e2000797.
4. Hozer F, Houenou J. Can neuroimaging disentangle bipolar disorder? Journal of affective disorders 2016;195:199–214.
5. Enkavi AZ, Eisenberg IW, Bissett PG, Mazza GL, MacKinnon DP, Marsch LA, et al. Large-scale analysis of test-retest reliabilities of self-regulation mea-

sures. Proceedings of the National Academy of Sciences 2019;116(12):5472–5477.
6. Cronbach LJ, Meehl PE. Construct validity in psychological tests. Psychological Bulletin 1955;52(4):281–302.
7. Borsboom D, Mellenbergh GJ, van Heerden J. The Concept of Validity. Psychological Review 2004;111(4):1061–1071.
8. Eisenberg IW, Bissett PG, Enkavi AZ, Li J, MacKinnon DP, Marsch LA, et al. Uncovering the structure of self-regulation through data-driven ontology discovery. Nature Communications 2019;10(1):1–13.
9. Nave G, Jung WH, Linnér RK, Kable JW, Koellinger PD. Are Bigger Brains Smarter? Evidence From a Large-Scale Preregistered Study:. Psychological Science 2018;.
10. Kievit RA, Fuhrmann D, Borgeest GS, Simpson-Kent IL, Henson RNA. The neural determinants of age-related changes in fluid intelligence: a pre-registered, longitudinal analysis in UK Biobank. Wellcome Open Research 2018;3.
11. Engemann DA, Kozynets O, Sabbagh D, Lemaître G, Varoquaux G, Liem F, et al. Combining magnetoencephalography with magnetic resonance imaging enhances learning of surrogate-biomarkers. eLife 2020 may;9:e54055. https://doi.org/10.7554/eLife.54055.
12. Collins R. What makes UK Biobank special? The Lancet 2012;379(9822):1173–1174.
13. Woo CW, Chang LJ, Lindquist MA, Wager TD. Building better biomarkers: brain models in translational neuroimaging. Nature Neuroscience 2017;20(3):365–377.
14. Varoquaux G. Cross-validation failure: Small sample sizes lead to large error bars. NeuroImage 2018;180:68–77.
15. Bzdok D, Meyer-Lindenberg A. Machine learning for precision psychiatry: opportunities and challenges. Biological Psychiatry: Cognitive Neuroscience and Neuroimaging 2018;3(3):223–230.
16. Dosenbach NU, Nardos B, Cohen AL, Fair DA, Power JD, Church JA, et al. Prediction of individual brain maturity using fMRI. Science 2010;329(5997):1358–1361.
17. Cole JH, Leech R, Sharp DJ, Initiative ADN. Prediction of brain age suggests accelerated atrophy after traumatic brain injury. Annals of neurology 2015;77(4):571–581.
18. Cole JH, Ritchie SJ, Bastin ME, Hernández MV, Maniega SM, Royle N, et al. Brain age predicts mortality. Molecular psychiatry 2018;23(5):1385.
19. Smith SM, Elliott LT, Alfaro-Almagro F, McCarthy P, Nichols TE, Douaud G, et al. Brain aging comprises many modes of structural and functional change with distinct genetic and biophysical associations. Elife 2020;9:e52677.
20. Koutsouleris N, Davatzikos C, Borgwardt S, Gaser C, Bottlender R, Frodl T, et al. Accelerated brain aging in schizophrenia and beyond: a neuroanatomical marker of psychiatric disorders. Schizophrenia bulletin 2014;40(5):1140–1153.
21. He T, An L, Feng J, Bzdok D, Holmes AJ, Eickhoff SB, et al. Meta-matching: a simple framework to translate phenotypic predictive models from big to small data. bioRxiv 2020;.
22. Liem F, Varoquaux G, Kynast J, Beyer F, Masouleh SK, Huntenburg JM, et al. Predicting brain-age from multimodal imaging data captures cognitive impairment. NeuroImage 2017;148:179–188.
23. Spiegelhalter D. How old are you, really? Communicating chronic risk through 'effective age'of your body and organs. BMC medical informatics and decision making 2016;16(1):1–6.
24. Smith SM, Vidaurre D, Alfaro-Almagro F, Nichols TE, Miller KL. Estimation of brain age delta from brain imaging. NeuroImage 2019;.
25. Gonneaud J, Baria AT, Binette AP, Gordon BA, Chhatwal JP, Cruchaga C, et al. Functional brain age prediction suggests accelerated aging in preclinical familial Alzheimer's disease, irrespective of fibrillar amyloid-beta pathology. bioRxiv 2020;.
26. Cattell RB, Scheier IH. The meaning and measurement of neuroticism and anxiety. 1961;.
27. Cattell RB. Theory of fluid and crystallized intelligence: A critical experiment. Journal of educational psychology 1963;54(1):1.
28. Shelton JT, Elliott EM, Matthews RA, Hill B, Gouvier W, others. The relationships of working memory, secondary memory, and general fluid intelligence: working memory is special. Journal of Experimental Psychology: Learning, Memory, and Cognition 2010;36(3):813.
29. Keyes KM, Platt J, Kaufman AS, McLaughlin KA. Association of Fluid Intelligence and Psychiatric Disorders in a Population-Representative Sample of US Adolescents. JAMA psychiatry 2017;74(2):179–188.
30. Khandaker GM, Dalman C, Kappelmann N, Stochl J, Dal H, Kosidou K, et al. Association of Childhood Infection With IQ and Adult Nonaffective Psychosis in Swedish Men: A Population-Based Longitudinal Cohort and Co-relative Study. JAMA Psychiatry 2018;75(4):356–362.
31. Costa PT, McCrae RR. Neo Pi-R. Psychological Assessment Resources Odessa, FL; 1992.
32. Eysenck SB, Eysenck HJ, Barrett P. A Revised Version of the Psychoticism Scale. Personality and Individual Differences 1985;6:21–29.
33. Shackman AJ, Tromp DP, Stockbridge MD, Kaplan CM, Tillman RM, Fox AS. Dispositional negativity: An integrative psychological and neurobiological perspective. Psychological bulletin 2016;142(12):1275.
34. Lynn R, Martin T. Gender differences in extraversion, neuroticism, and psychoticism in 37 nations. The Journal of social psychology 1997;137(3):369–373.
35. Hettema JM, Neale MC, Myers JM, Prescott CA, Kendler KS. A population-based twin study of the relationship between neuroticism and internalizing disorders. American journal of Psychiatry 2006;163(5):857–864.
36. Birley AJ, Gillespie NA, Heath AC, Sullivan PF, Boomsma DI, Martin NG. Heritability and nineteen-year stability of long and short EPQ-R Neuroticism scales. Personality and individual differences 2006;40(4):737–747.
37. Pedersen NL, Plomin R, McClearn GE, Friberg L. Neuroticism, extraversion, and related traits in adult twins reared apart and reared together. Journal of personality and social psychology 1988;55(6):950.
38. Lahey BB. Public health significance of neuroticism. American Psychologist 2009;64(4):241.
39. Tyrer P, Reed GM, Crawford MJ. Classification, assessment, prevalence, and effect of personality disorder. The Lancet 2015;385(9969):717–726.
40. Power RA, Pluess M. Heritability estimates of the Big Five personality traits based on common genetic variants. Translational psychiatry 2015;5(7):e604.
41. Vukasović T, Bratko D. Heritability of personality: a meta-analysis of behavior genetic studies. Psychological bulletin 2015;141(4):769.
42. Yarkoni T. Neurobiological substrates of personality: A critical overview. APA handbook of personality and social psychology 2015;4:61–83.
43. Dubois J, Galdi P, Han Y, Paul LK, Adolphs R. Resting-State Functional Brain Connectivity Best Predicts the Personality Dimension of Openness to Experience. Personality Neuroscience 2018;1.
44. Dubois J, Galdi P, Paul LK, Adolphs R. A distributed brain network predicts general intelligence from resting-state human neuroimaging data. Philosophical Trans-

actions of the Royal Society B: Biological Sciences 2018;373(1756):20170284.
45. Quercia D, Kosinski M, Stillwell D, Crowcroft J. Our twitter profiles, our selves: Predicting personality with twitter; 2011. p. 180–185.
46. Youyou W, Kosinski M, Stillwell D. Computer-based personality judgments are more accurate than those made by humans. Proceedings of the National Academy of Sciences 2015;112(4):1036–1040.
47. Horn JL, Donaldson G, Engstrom R. Apprehension, Memory, and Fluid Intelligence Decline in Adulthood. Research on Aging 1981;3(1):33–84. https://doi.org/10.1177/016402758131002.
48. Eysenck HJ. The continuity of abnormal and normal behavior. Psychological Bulletin 1958;55(6):429–432.
49. Perlis RH. Translating biomarkers to clinical practice. Molecular Psychiatry 2011;16(11):1076–1087.
50. Stevens SS, et al. On the theory of scales of measurement 1946;.
51. O'brien RM. A caution regarding rules of thumb for variance inflation factors. Quality & quantity 2007;41(5):673–690.
52. Varoquaux G, Raamana PR, Engemann DA, Hoyos-Idrobo A, Schwartz Y, Thirion B. Assessing and tuning brain decoders: Cross-validation, caveats, and guidelines. NeuroImage 2017;145:166 – 179. http://www.sciencedirect.com/science/article/pii/S105381191630595X, individual Subject Prediction.
53. Gelman A, Hill J. Data analysis using regression and multilevel/hierarchical models. Cambridge university press; 2006.
54. Borsboom D. Measuring the mind: Conceptual issues in contemporary psychometrics. Cambridge University Press; 2005.
55. Terracciano A, Costa Jr PT. Smoking and the Five-Factor Model of personality. Addiction 2004;99(4):472–481.
56. Topiwala A, Allan CL, Valkanova V, Zsoldos E, Filippini N, Sexton C, et al. Moderate alcohol consumption as risk factor for adverse brain outcomes and cognitive decline: longitudinal cohort study. bmj 2017;357:j2353.
57. Wang J, Knol MJ, Tiulpin A, Dubost F, de Bruijne M, Vernooij MW, et al. Gray matter age prediction as a biomarker for risk of dementia. Proceedings of the National Academy of Sciences 2019;116(42):21213–21218.
58. Bzdok D, Engemann D, Thirion B. Inference and Prediction Diverge in Biomedicine. Cell Press Patterns 2020 2020/10/13;https://doi.org/10.1016/j.patter.2020.100119.
59. Cole JH, Poudel RP, Tsagkrasoulis D, Caan MW, Steves C, Spector TD, et al. Predicting brain age with deep learning from raw imaging data results in a reliable and heritable biomarker. NeuroImage 2017;163:115–124.
60. He T, Kong R, Holmes AJ, Sabuncu MR, Eickhoff SB, Bzdok D, et al. Is deep learning better than kernel regression for functional connectivity prediction of fluid intelligence? In: 2018 International Workshop on Pattern Recognition in Neuroimaging (PRNI) IEEE; 2018. p. 1–4.
61. Carretta TR. Pilot candidate selection method. Aviation Psychology and Applied Human Factors 2011;.
62. Carretta TR, Ree MJ. Pilot-candidate selection method: Sources of validity. The International Journal of Aviation Psychology 1994;4(2):103–117.
63. Krapohl E, Rimfeld K, Shakeshaft NG, Trzaskowski M, McMillan A, Pingault JB, et al. The high heritability of educational achievement reflects many genetically influenced traits, not just intelligence. Proceedings of the national academy of sciences 2014;111(42):15273–15278.
64. Colodro-Conde L, Couvy-Duchesne B, Zhu G, Coventry WL, Byrne EM, Gordon S, et al. A direct test of the diathesis–stress model for depression. Molecular psychiatry 2018;23(7):1590–1596.
65. Anatürk M, Kaufmann T, Cole JH, Suri S, Griffanti L, Zsoldos E, et al. Prediction of brain age and cognitive age: Quantifying brain and cognitive maintenance in aging. Human brain mapping 2021;42(6):1626–1640.
66. Maglanoc LA, Kaufmann T, Meer Dvd, Marquand AF, Wolfers T, Jonassen R, et al. Brain Connectome Mapping of Complex Human Traits and Their Polygenic Architecture Using Machine Learning. Biological Psychiatry 2020;87(8):717–726.
67. Khandaker GM, Barnett JH, White IR, Jones PB. A quantitative meta-analysis of population-based studies of premorbid intelligence and schizophrenia. Schizophrenia research 2011;132(2-3):220–227.
68. Dadi K, Engemann D, Empirical Proxy Measures; 2021. https://github.com/KamalakerDadi/empirical_proxy_measures.
69. Sudlow C, Gallacher J, Allen N, Beral V, Burton P, Danesh J, et al. UK Biobank: An Open Access Resource for Identifying the Causes of a Wide Range of Complex Diseases of Middle and Old Age. PLOS Medicine 2015;12(3):1–10.
70. Miller KL, Alfaro-Almagro F, Bangerter NK, Thomas DL, Yacoub E, Xu J, et al. Multimodal population brain imaging in the UK Biobank prospective epidemiological study. Nature neuroscience 2016;19(11):1523–1536.
71. Schulz MA, Yeo BT, Vogelstein JT, Mourao-Miranada J, Kather JN, Kording K, et al. Different scaling of linear models and deep learning in UKBiobank brain images versus machine-learning datasets. Nature communications 2020;11(1):1–15.
72. Flint C, Cearns M, Opel N, Redlich R, Mehler DM, Emden D, et al. Systematic misestimation of machine learning performance in neuroimaging studies of depression. Neuropsychopharmacology 2021;46(8):1510–1517.
73. IJ H. Statistics versus machine learning. Nature methods 2018;15(4):233.
74. Alfaro-Almagro F, Jenkinson M, Bangerter NK, Andersson JLR, Griffanti L, Douaud G, et al. Image processing and Quality Control for the first 10,000 brain imaging datasets from UK Biobank. NeuroImage 2018;166:400–424.
75. UK Biobank manual: Touch-screen Fluid intelligence test; 2012. https://biobank.ctsu.ox.ac.uk/crystal/crystal/docs/Fluidintelligence.pdf.
76. UK Biobank manual: Field Descriptions and Derivation for variables related to bipolar disorder, major depression status and neuroticism score; 2013. https://biobank.ctsu.ox.ac.uk/crystal/crystal/docs/MentalStatesDerivation.pdf.
77. Fry A, Littlejohns TJ, Sudlow C, Doherty N, Adamska L, Sprosen T, et al. Comparison of Sociodemographic and Health-Related Characteristics of UK Biobank Participants With Those of the General Population. American Journal of Epidemiology 2017 06;186(9):1026–1034. https://doi.org/10.1093/aje/kwx246.
78. Little RJA, Rubin DB. Statistical Analysis with Missing Data. New York, NY, USA: John Wiley & Sons, Inc.; 1986.
79. Josse J, Prost N, Scornet E, Varoquaux G. On the consistency of supervised learning with missing values; 2019, https://hal.archives-ouvertes.fr/hal-02024202, working paper or preprint.
80. Lerch JP, van der Kouwe AJ, Raznahan A, Paus T, Johansen-Berg H, Miller KL, et al. Studying neuroanatomy using MRI. Nature neuroscience 2017;20:314 – 326.
81. Ritchie SJ, Dickie DA, Cox SR, Valdes Hernandez MdC, Corley J, Royle NA, et al. Brain volumetric changes and cognitive ageing during the eighth decade of life. Human Brain Mapping 2015;36(12):4910–4925.

82. Cox S, Ritchie S, Fawns-Ritchie C, Tucker-Drob E, Deary I. Structural brain imaging correlates of general intelligence in UK Biobank. Intelligence 2019;76:101376.
83. Thompson PM, Hayashi KM, Dutton RA, Chiang MC, Leow AD, Sowell ER, et al. Tracking Alzheimer's Disease. Annals of the New York Academy of Sciences 2007;1097:183–214.
84. Desikan RS, Ségonne F, Fischl B, Quinn BT, Dickerson BC, Blacker D, et al. An automated labeling system for subdividing the human cerebral cortex on MRI scans into gyral based regions of interest. Neuroimage 2006;31(3):968–980.
85. Diedrichsen J, Balsters JH, Flavell J, Cussans E, Ramnani N. A probabilistic MR atlas of the human cerebellum. NeuroImage 2009;46(1):39 – 46.
86. UK Biobank categories: Regional grey matter volumes (FAST);. http://biobank.ctsu.ox.ac.uk/crystal/label.cgi?id=1101.
87. UK Biobank categories: Subcortical volumes (FIRST);. http://biobank.ctsu.ox.ac.uk/crystal/label.cgi?id=1102.
88. Behrens TEJ, Woolrich MW, Jenkinson M, Johansen-Berg H, Nunes RG, Clare S, et al. Characterization and propagation of uncertainty in diffusion-weighted MR imaging. Magnetic Resonance in Medicine 2003;50:1077–1088.
89. Conturo TE, Lori NF, Cull TS, Akbudak E, Snyder AZ, Shimony JS, et al. Tracking neuronal fiber pathways in the living human brain. Proceedings of the National Academy of Sciences 1999;96:10422–10427.
90. Saygin ZM, Osher DE, Norton ES, Youssoufian DA, Beach SD, Feather J, et al. Connectivity precedes function in the development of the visual word form area. Nature neuroscience 2016;19.
91. UK Biobank categories: Diffusion MRI skeleton measurements;. DiffusionMRIskeletonmeasurements.
92. de Groot M, Vernooij MW, Klein S, Ikram MA, Vos FM, Smith SM, et al. Improving alignment in Tract-based spatial statistics: Evaluation and optimization of image registration. NeuroImage 2013;76:400–411.
93. Biswal B, Yetkin FZ, Haughton VM, Hyde JS. Functional connectivity in the motor cortex of resting human brain using echo-planar mri. Magnetic Resonance in Medicine 1995;34(4):537–541.
94. Greicius MD, Srivastava G, Reiss AL, Menon V. Default-mode network activity distinguishes Alzheimer's disease from healthy aging: evidence from functional MRI. Proceedings of the National Academy of Sciences 2004;101:4637.
95. Ledoit O, Wolf M. Honey, I shrunk the sample covariance matrix. The Journal of Portfolio Management 2004;30(4):110–119.
96. Varoquaux G, Baronnet F, Kleinschmidt A, Fillard P, Thirion B. Detection of brain functional-connectivity difference in post-stroke patients using group-level covariance modeling. Medical image computing and computer-assisted intervention: MICCAI International Conference on Medical Image Computing and Computer-Assisted Intervention 2010;13(Pt 1):200–208.
97. Sabbagh D, Ablin P, Varoquaux G, Gramfort A, Engeman DA. Manifold-regression to predict from MEG/EEG brain signals without source modeling. In: Advances in Neural Information Processing Systems (NeurIPS); 2019. .
98. Dadi K, Rahim M, Abraham A, Chyzhyk D, Milham M, Thirion B, et al. Benchmarking functional connectome-based predictive models for resting-state fMRI. NeuroImage 2019;192:115–134.
99. Pervaiz U, Vidaurre D, Woolrich MW, Smith SM. Optimising network modelling methods for fMRI. NeuroImage 2020;211:116604.
100. Abraham A, Pedregosa F, Eickenberg M, Gervais P, Mueller A, Kossaifi J, et al. Machine learning for neuroimaging with scikit-learn. Frontiers in Neuroinformatics 2014;8.
101. Poldrack RA, Huckins G, Varoquaux G. Establishment of best practices for evidence for prediction: a review. JAMA psychiatry 2020;77(5):534–540.
102. Breiman L. Random Forests. Machine Learning 2001;45(1):5–32.
103. Hastie T, Tibshirani R, Friedman J, Franklin J. The elements of statistical learning: data mining, inference and prediction. The Mathematical Intelligencer 2005;27(2):83–85.
104. Varoquaux G, Raamana PR, Engemann DA, Hoyos-Idrobo A, Schwartz Y, Thirion B. Assessing and tuning brain decoders: Cross-validation, caveats, and guidelines. NeuroImage 2017;145(August 2015):166–179.
105. O'Connor D, Lake EM, Scheinost D, Constable RT. Resample aggregating improves the generalizability of connectome predictive modeling. NeuroImage 2021;236:118044.
106. Gemein LAW, Schirrmeister RT, Chrabąszcz P, Wilson D, Boedecker J, Schulze-Bonhage A, et al. Machine-learning-based diagnostics of EEG pathology. NeuroImage 2020;220:117021. http://www.sciencedirect.com/science/article/pii/S1053811920305073.
107. Le TT, Kuplicki RT, McKinney BA, Yeh HW, Thompson WK, Paulus MP, et al. A Nonlinear Simulation Framework Supports Adjusting for Age When Analyzing BrainAGE. Frontiers in Aging Neuroscience 2018;10:317. https://www.frontiersin.org/article/10.3389/fnagi.2018.00317.
108. Smith SM, Vidaurre D, Alfaro-Almagro F, Nichols TE, Miller KL. Estimation of brain age delta from brain imaging. NeuroImage 2019;200:528 – 539.
109. Pedregosa F, Varoquaux G, Gramfort A, Michel V, Thirion B, Grisel O, et al. Scikit-learn: Machine Learning in Python. J Mach Learn Res 2011 nov;12:2825–2830.
110. R Core Team. R: A Language and Environment for Statistical Computing. R Foundation for Statistical Computing, Vienna, Austria; 2019, https://www.R-project.org/.
111. Wickham H. ggplot2: Elegant Graphics for Data Analysis. Springer-Verlag New York; 2016. http://ggplot2.org.
112. Clarke E, Sherrill-Mix S. ggbeeswarm: Categorical Scatter (Violin Point) Plots; 2017, https://CRAN.R-project.org/package=ggbeeswarm, r package version 0.6.0.
113. Gelman A, Su YS. arm: Data Analysis Using Regression and Multilevel/Hierarchical Models; 2020, https://CRAN.R-project.org/package=arm, r package version 1.11-1.
114. UK Biobank website;. http://www.ukbiobank.ac.uk.
115. UK Biobank access procedure;. https://www.ukbiobank.ac.uk/enable-your-research/apply-for-access.

Figure 1

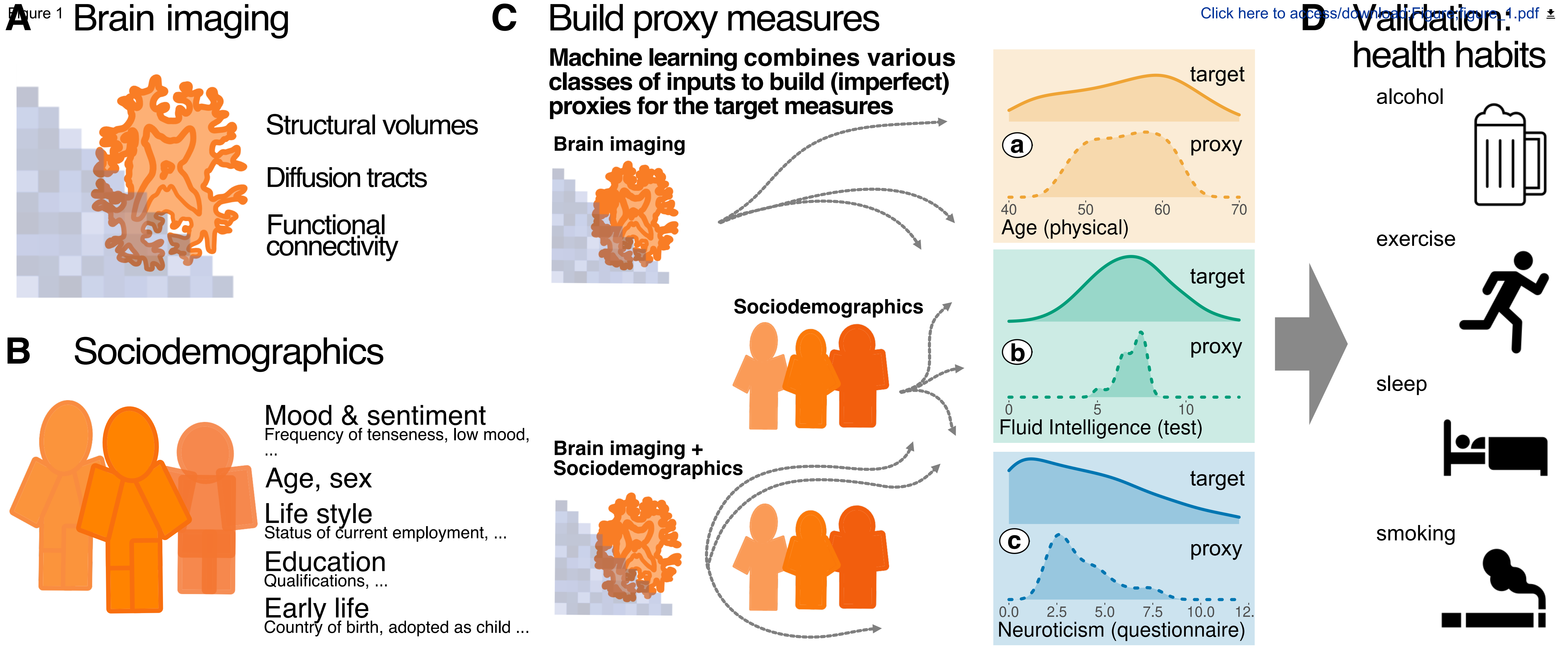

Click here to access/download;Figure;figure_1.pdf

Figure 2

Click here to access/download;Figure;fig_2.pdf

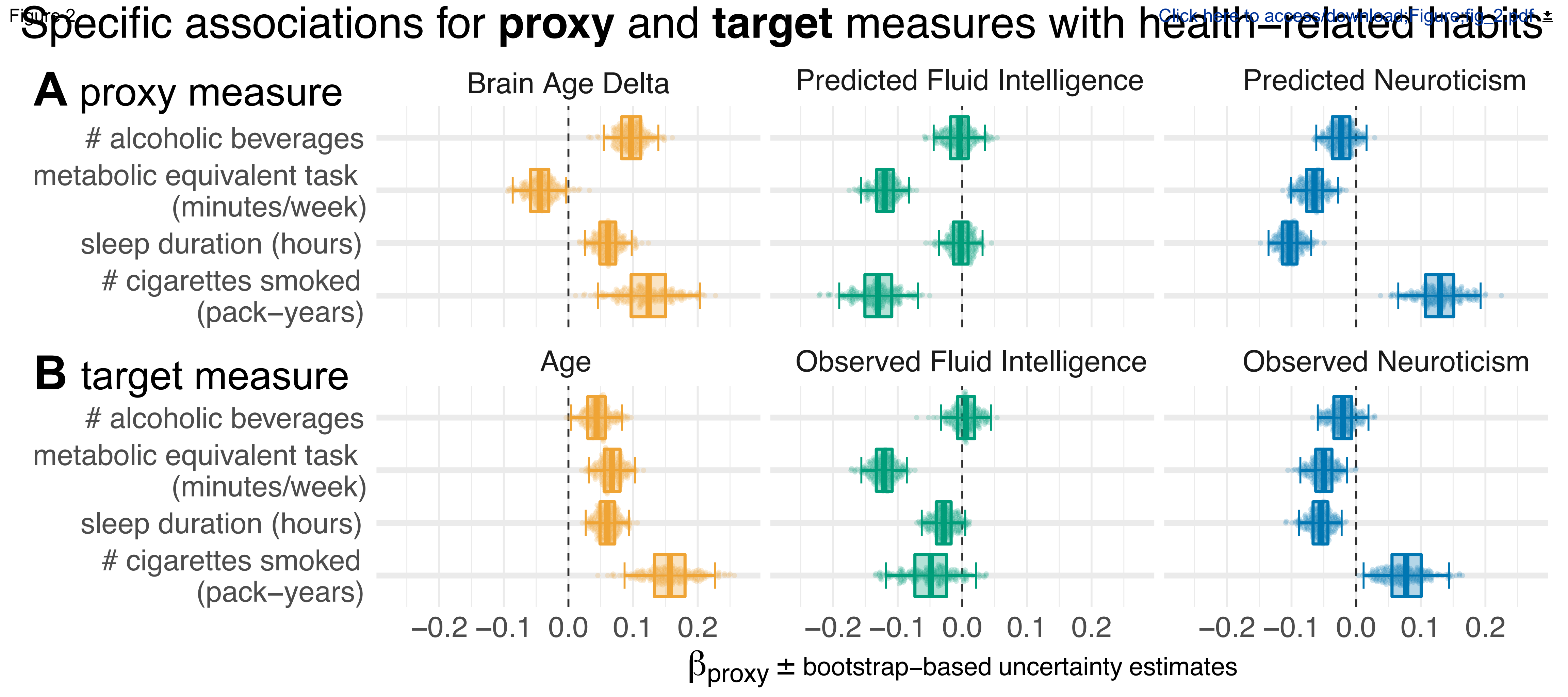

Figure 3

Click here to access/download;Figure;figure_summary_validation.pdf

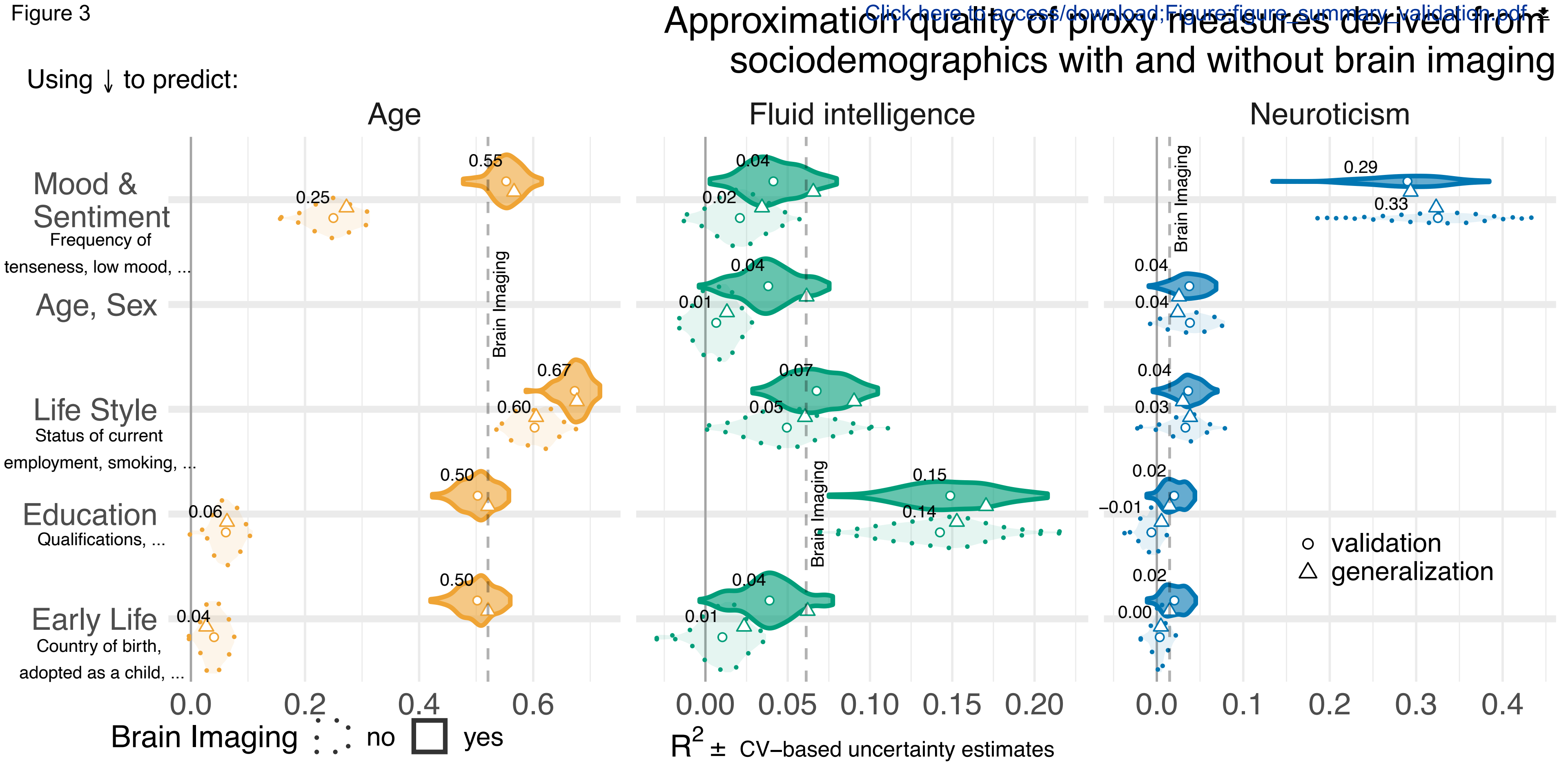

Figure 4

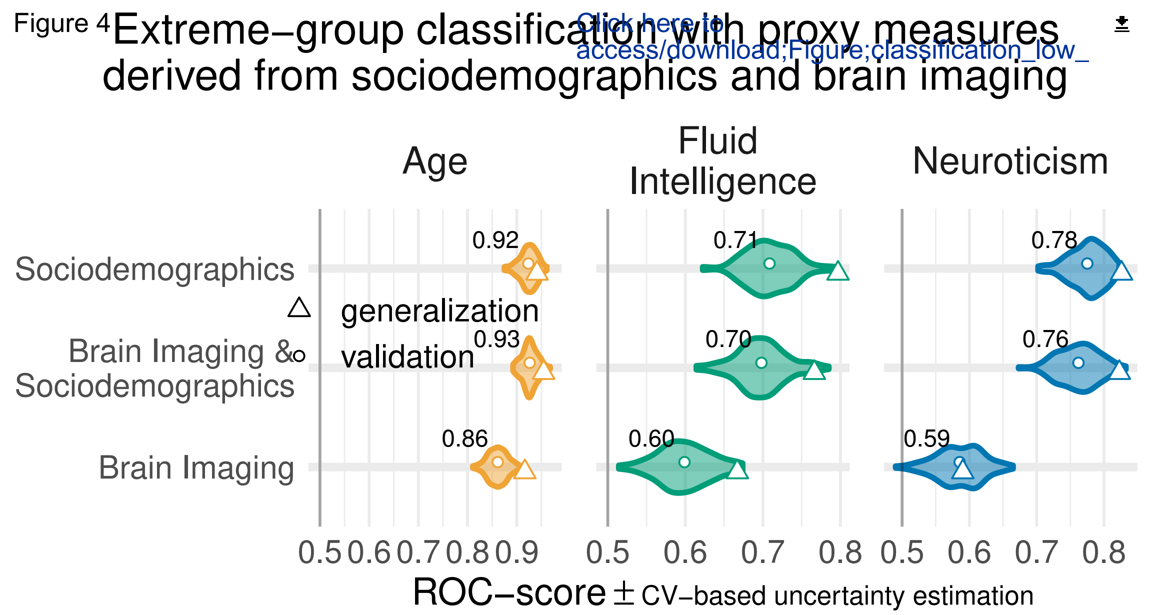

Extreme−group classification with proxy measures derived from sociodemographics and brain imaging
Click here to access/download;Figure;classification_low_
Age
Fluid Intelligence
Neuroticism
Sociodemographics
Brain Imaging & Sociodemographics
Brain Imaging
generalization
validation
0.92
0.93
0.86
0.71
0.70
0.60
0.78
0.76
0.59
0.5 0.6 0.7 0.8 0.9
0.5 0.6 0.7 0.8
0.5 0.6 0.7 0.8
ROC−score ± CV−based uncertainty estimation

Diff with R1 Submission

Click here to access/download
Supplementary Material
diff-with-r1-submission.pdf

Click here to access/download
Supplementary Material
supplement_r2.pdf

Cover Letter

Click here to access/download;Personal Cover;dadi_cover_letter_r2-signed.pdf

Paris, le 10 août 2021

Dr. Denis-Alexander Engemann
research scientist, INRIA-Saclay
www.denis-engemann.de
denis-alexander.engemann@inria.fr

Dear Dr. Zauner, Dear editors of *GigaScience*

Please, find for your consideration the second revision of ourmanuscript entitled "Population modeling with machine learning can enhance measures of mental health".

We would like to thank the editor and the reviewer for the attentive evaluation of our manuscript and the helpful comments. This has allowed us to further refine the manuscript regarding editorial standards and the presentation of our workflow. The key points addressed in this revision are a major update to figure 1 as well as changes in the text that, hopefully, will help the reader to better understand the core ideas of the paper and the methods workflow.

A detailed display of the changes in the manuscript is available in the file 'diff-with-r1-submission.pdf' enclosed with the resubmission.

This work is not under consideration elsewhere and that we believe that GigaScience with its interdisciplinary and innovation-driven culture would be our preferred outlet to disseminate this work.

Sincerely yours,
Kamalaker Dadi, Gaël Varoquaux, Josselin Houenou, Danilo Bzdok, Bertrand Thirion & Denis A. Engemann

RESEARCH CENTRE
SACLAY ÎLE-DE-FRANCE
1 Rue Honoré
d'Estienne d'Orves
91120 Palaiseau
Phone: +33 (0)1 72 92 59 00
Fax: +33 (0)1 74 85 42 42

www.inria.fr

Click here to access/download;Personal Cover;reply-to-the-reviewer.pdf

Summary

We would like to thank the editor and the reviewer for the attentive evaluation of our manuscript and the helpful comments. This has allowed us to further refine the manuscript regarding editorial standards and the presentation of our workflow. The key points addressed in this revision are a major update to figure 1 as well as changes in the text that, hopefully, will help the reader to better understand the core ideas of the paper and the methods workflow.
A detailed display of the changes in the manuscript is available in the file `diff-with-r1-submission.pdf` enclosed with the resubmission.

Editorial points

- Please turn URLs mentioned in the text or the footnotes into numbered citations, included in the bibliography. Software homepages, data sets and other URLs or DOIs should all be included in the bibliography and cited from there. please see our instructions for authors for formatting guidance.

- In the "data availability" section, please explain the access procedure for the UK BioBank data (Is it open or controlled access? For human data, controlled access is fine, but please mention the requirements/procedure to get access).

- Our data curators will prepare an archival snapshot of code and supporting data. Please also include this in the bibliography (including the doi link) and cite it by number from the "availability" section.

We thank the editor for the support regarding editorial standards. In the revised versions, we have addressed all three points.

Reviewer reports:
Reviewer #1: The substantial revision improved the paper and is appreciated by the reviewer. The details have been enhanced. However, the reviewer still has some **concerns about the basic logic and its presentation of the paper** after reviewing all the comments from other reviewers and the feedback from the author.

We thank the reviewer for the positive appreciation of our revision efforts. We hope that the second revision, in response to the thoughtful and constructive criticism provided by the reviewer, helped improve the accessibility issues regarding the basic logic and presentation of the paper.

Figure 1 is helpful (BTW, the font is too small and smaller than other figures).

We thank the reviewer for this positive feedback and the pointer regarding the font sizes. To address this issue, we have increased the font size of the smallest fonts and scaled up the

figure to appear larger inside the manuscript, using the full text width instead of one single column. As a result, all font sizes have been increased.

But if we consider the current approach again, when the machine learning (ML) has perfect performance to generate the so called "proxy measures", these measures should match exactly each individual's age, fluid-intelligence and neuroticism. What the author claimed about proxy measures providing better assessment to other health related variables **might be simply due to the imperfectness**

We thank the reviewer for this opportunity for clarification. The reviewer recognizes an important point about the preconditions for building proxy measures: The predictors from which a proxy measure is modelled should not allow for perfect prediction (which is certainly guaranteed in our context given that the precise data generating mechanisms are unknown and it is reasonable to assume that many important variables remain unobserved). A proxy measure can only bring additional information if, in the first place, there is residual variance in the target that is not explained by the predictors. The entire approach cannot work if proxies and targets are – via perfect prediction – the same.
To make sure this point is not overlooked, in the introduction, we have revised the paragraph in which brain age is introduced. Moreover, we have added a note in the caption figure caption of figure 1.

Changes in the main text:

> Yet, by (imperfectly) predicting the age from brain data, machine-learning can capture the relevant signal. Based on a population of brain images, it extracts the *best guess* for the age of a person, indirectly positioning that person within the population.

Related changes in caption of figure 1:

> Note that proxy measures can only add to the target measures if they are not identical, *i.e.*, if the approximation of the target from the given inputs is imperfect (guaranteed in our context as the exact data generating mechanism is unknown and causally important variables remain unobserved).

Related changes in figure 1 (bold text in panel C):

> Machine learning combines various classes of inputs to build (imperfect) proxies for the target measures.

The author may need to address this and present the logic of the paper in a clearer way to help the readers understand the main point and results of the paper. In this regard, Figure 1 is incomplete in addressing the full flow of the paper, which is necessary for such a seemingly complex paper in the reviewer's opinion.

We thank the reviewer for this excellent recommendation. It is true that because of the substantial revisions, now the concept figure 1 is visibly out of sync with the full story, which indeed may cause confusion or simply make readers miss the main ideas. To present the key ideas of the work with greater clarity to the reader, we have added an outlook on the organization of the paper at the end of the introduction and substantially extended figure 1 to now depict the full workflow of the paper. To generate more attention for the key idea of comparing multiple proxy measures with their respective targets, we have added an explicit illustration of differences between proxies and target measures. To prepare the reader for the core of the paper in which proxies and targets are benchmarked regarding their potential complementarity at statistically explaining health-related behavior, we have included a new panel in figure 1 in which the health behaviors under investigation are illustrated. We have updated the figure caption accordingly.

Changes in the main text (end of introduction):

> The paper is organized as follows: We first present a summary of the methodology and the workflow of building distinct proxy measures for age, fluid intelligence and neuroticism using machine learning (Figure 1). We then benchmark the proxy and the original target measures against real-world patterns of health-relevant behavior. Subsequently, through systematic model comparisons, we assess the relative contributions of brain imaging and sociodemographic data for prediction performance in the regression and classification settings. The complementarity between the proxy measures is, finally, discussed in the light of statistical considerations, potential data generating mechanisms, and applications for public health and clinical research.

Figure 1 after substantial revisions:

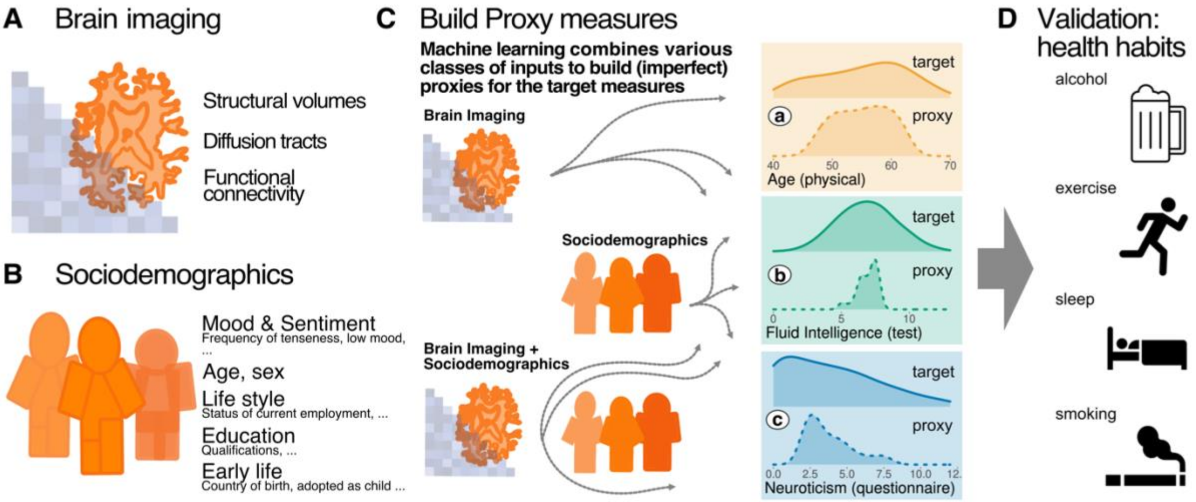

Figure 1 caption:

> **Methods workflow: building and evaluating proxy measures.** We combined multiple brain-imaging modalities (**A**) with sociodemographic data (**B**) to approximate health-

related biomedical and psychological constructs (**C**), *i.e.*, brain age (accessed through prediction of chronological age), cognitive capacity (accessed through a fluid-intelligence test) and the tendency to report negative emotions (accessed through a neuroticism questionnaire). We included the imaging data from the 10,000-subjects release of the UK biobank. Among imaging data (**A**) we considered features related to cortical and subcortical volumes, functional connectivity from rfMRI based on ICA networks, and white-matter molecular tracts from diffusive directions (see Table 1 for an overview about the multiple brain-imaging modalities).

We then grouped the sociodemographic data (**B**) into five different blocks of variables related to self-reported mood & sentiment, primary demographics, lifestyle, education, and early-life events (Table 2 lists the number of variables in each block).

We systematically compared the approximations of all three targets based on either brain images and sociodemographics in isolation or combined (**C**) to evaluate the relative contribution of these distinct inputs. Note that proxy measures can only add to the target measures if they are not identical, *i.e.*, if the approximation of the target from the given inputs is imperfect (guaranteed in our context as the exact data generating mechanism is unknown and causally important variables remain unobserved). Using the full model (brain imaging + sociodemographics), we benchmarked complementarity of the proxy measures and the target measures with regard to real-world patterns of health behavior (**D**), i.e., the number of alcoholic beverages, exercise (metabolic equivalent task), sleep duration and the number of cigarettes smoked. Potentially additive effects between proxies and targets were gauged using multiple linear regression. Models were developed on 50% of the data (randomly drawn) based on random forest regression guided by Monte Carlo cross-validation with 100 splits (see section **Model Development and Generalization Testing**). We assessed generalization and health implications using the other 50% of the data as fully independent out-of-sample evaluations (see section **Statistical Analysis**). Learning curves suggested that this split-half approach provided sufficient data for model construction (Figure 1 – Figure 1 supplement).
