# Supplementary material for: Population modeling with machine learning can enhance measures of mental health
Source: Gigascience. 2021 Oct 15;10(10):giab071. doi: 10.1093/gigascience/giab071 (PMC8559220; doi:10.1093/gigascience/giab071)
Supplement: giab071_Supplemental_File [file giab071_supplemental_file.pdf]

Supporting Information

Appendix 1: Additional results

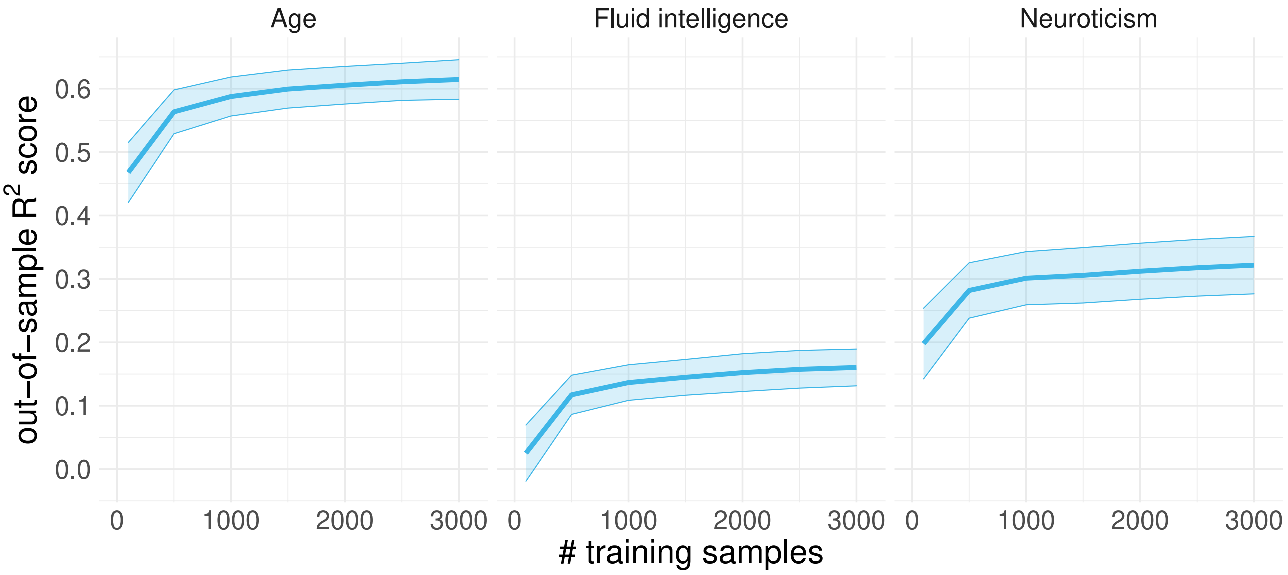

Figure 1 – Figure supplement 1. Learning curves on the random split-half validation used for model building. To facilitate comparisons, we evaluated predictions of age, fluid intelligence and neuroticism from a complete set of socio-demographic variables without brain imaging using the coefficient of determination R^2 metric (y-axis) to compare results obtained from 100 to 3000 training samples (x-axis). The cross-validation (CV) distribution was obtained from 100 Monte Carlo splits. Across targets, performance started to plateau after around 1000 training samples with scores virtually identical to the final model used in subsequent analyses. These benchmarks suggest that inclusion of additional training samples would not have led to substantial improvements in performance.

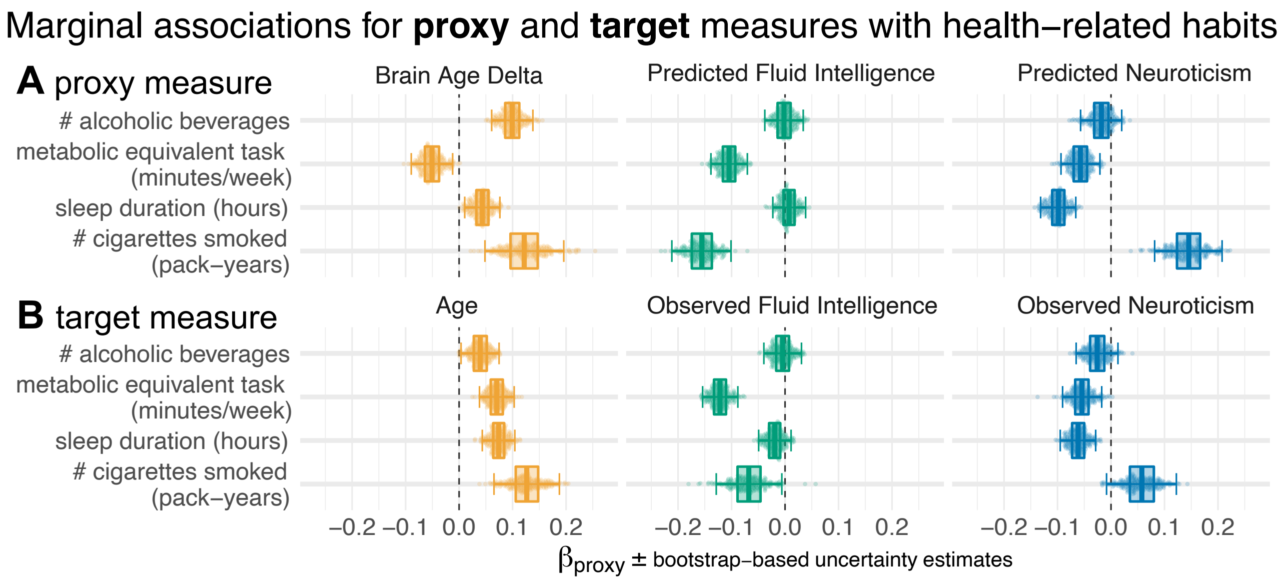

Figure 2 – Figure supplement 1. Marginal associations between proxy measures and health-related habits. Marginal (instead of conditional) estimates using univariate regression. Same visual conventions as in Figure 2.

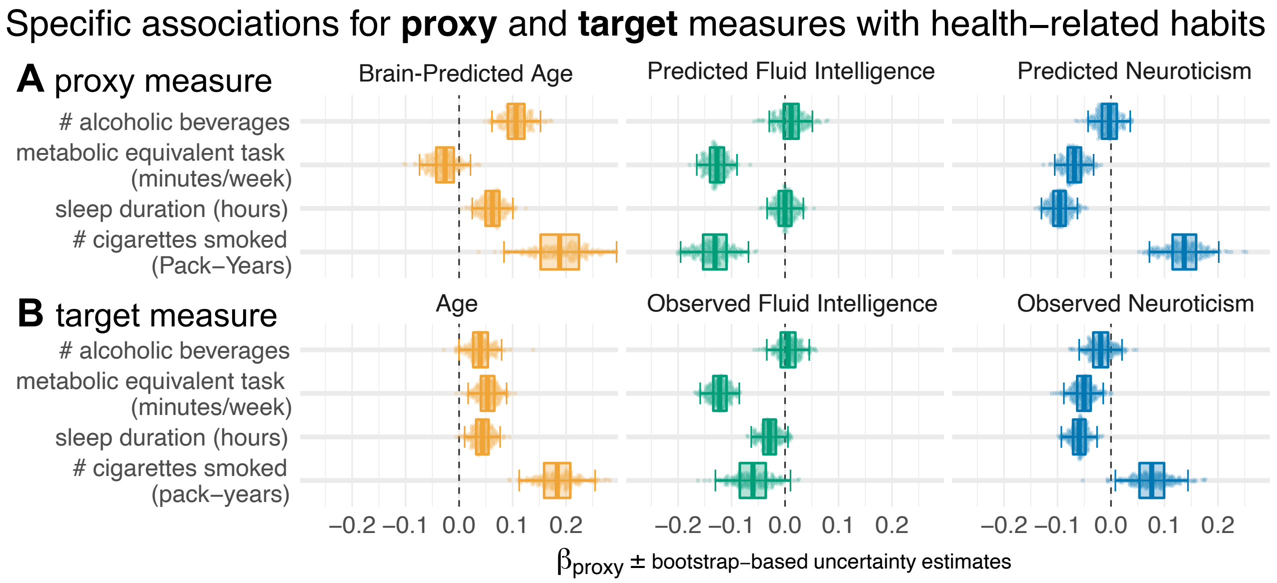

Figure 2 – Figure supplement 2. Conditional associations between proxy measures and health-related habits without explicit brain age delta. Conditional estimates using multivariate regression. Instead of the brain age delta, the brain-predicted age is included alongside an age-deconfounder as used in the main analysis. Same visual conventions as in Figure 2.

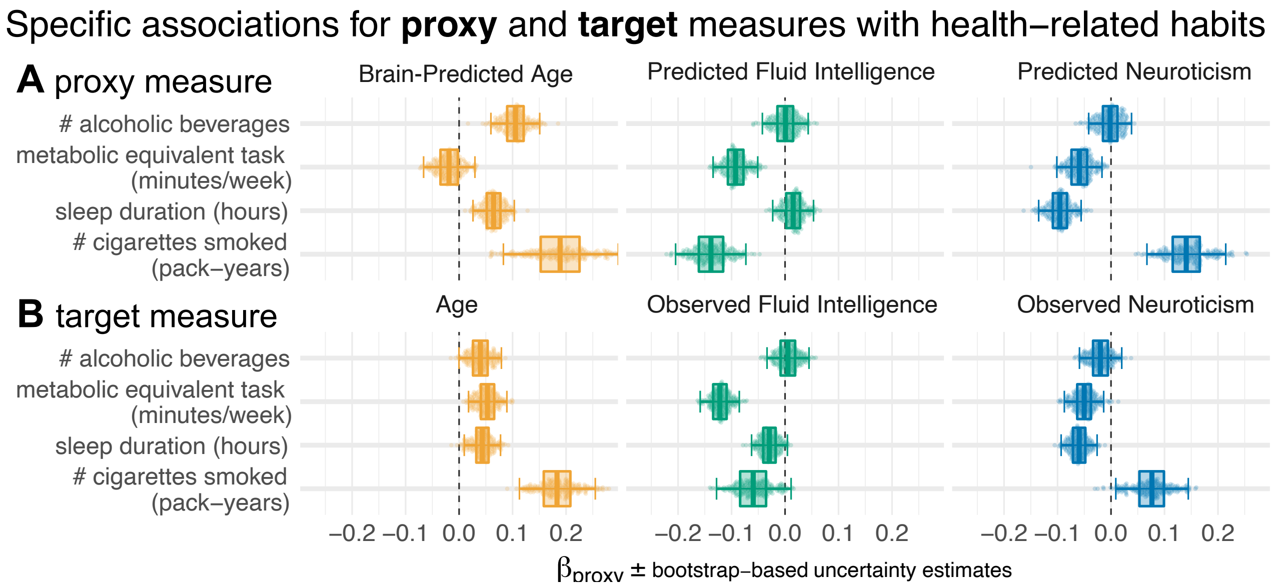

Figure 2 – Figure supplement 3. Conditional associations between proxy measures and health-related habits with-proxy-specific deconfounding. Conditional estimates using multivariate regression. Instead of the brain age delta, the brain-predicted age is included alongside an age-deconfounder as used in the main analysis. Moreover, predicted fluid intelligence and neuroticism are deconfounded for the target values at training time, analogous to the brain age predictions. Same visual conventions as in Figure 2.

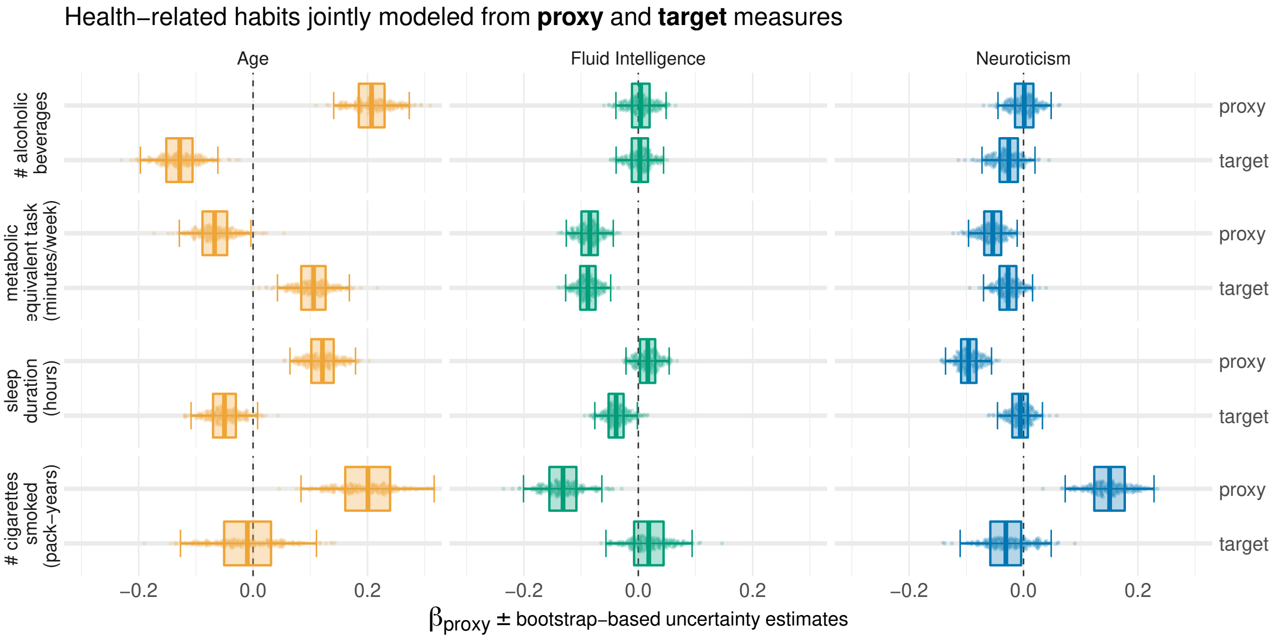

Figure 2 – Figure supplement 4. Joint modeling of health-related habits from proxy and target measures. Conditional estimates using multivariate regression. Every health-related habit (double rows) is modeled simultaneously from multiple proxies and targets. Same visual conventions as in Figure 2. Across health-habits, additive effects emerged not only for proxies and targets within the same measure (*e.g.* age) but also across measures (*e.g.* age and fluid intelligence). For illustration, we shall consider two examples. Regarding alcohol consumption, age was the most important measure and opposite conditional effects were observed for the proxy and the target: Across the age range, people with higher brain age tended to drink more and across the brain-age range, older people tended to drink less. For smoking, the proxy measures were the most important variables with clear non-zero coefficients, pointing in different directions across target domains. Holding fluid intelligence and neuroticism constant (targets and proxies), people with higher brain age tended to have been smoking for a longer time. At the same time, those who scored lower on predicted fluid intelligence across the entire range of age, predicted age, measured fluid intelligence, predicted neuroticism and neuroticism, have been smoking for a longer time. Finally, those who scored higher on predicted neuroticism tended to smoke more across the ranges of all other measures.

Table S1. Paired difference between purely sociodemographic and models including brain imaging on held-out data.

Target	sociodemographics	R^2_{diff}	p-value	CI_{low}	CI_{high}
Age	Early Life	0.494	0.0001	0.473	0.515
Age	Education	0.458	0.0001	0.437	0.479
Age	Life style	0.071	0.0001	0.058	0.085
Age	Mood & sentiment	0.294	0.0001	0.272	0.315
Fluid intelligence	Age, Sex	0.048	0.0001	0.040	0.057
Fluid intelligence	Early Life	0.039	0.0001	0.027	0.050
Fluid intelligence	Education	0.018	0.0001	0.010	0.025
Fluid intelligence	Life style	0.030	0.0001	0.020	0.040
Fluid intelligence	Mood & sentiment	0.031	0.0001	0.019	0.043
Neuroticism	Age, Sex	0.001	0.6789	−0.006	0.008
Neuroticism	Early Life	0.010	0.0697	−0.001	0.021
Neuroticism	Education	0.009	0.0817	−0.001	0.020
Neuroticism	Life style	−0.008	0.1750	−0.020	0.004
Neuroticism	Mood & sentiment	−0.030	0.0001	−0.041	−0.018

Table S2. Difference statistics for classification on the held-out set for sociodemographic vs combined approximation.

Target	$AUC_{diff\ observed}$	p-value	CI_{low}	CI_{high}
Age	0.013	0.0008	0.006	0.021
Fluid intelligence	−0.031	0.0001	−0.044	−0.017
Neuroticism	−0.003	0.4818	−0.013	0.006

Table S3. Inferential statistics for joint proxy-target models of health-related habits

	Outcome			
	Alcohol	Activity	Sleep	Smoking
predicted Age	0.208^{***}	-0.066^{**}	0.121^{***}	0.200^{***}
	(0.034)	(0.032)	(0.029)	(0.058)
Age	-0.129^{***}	0.105^{***}	-0.050^{*}	−0.008
	(0.035)	(0.032)	(0.030)	(0.060)
predicted Fluid Intelligence	0.004	-0.085^{***}	0.016	-0.132^{***}
	(0.022)	(0.021)	(0.019)	(0.035)
Fluid Intelligrence	0.003	-0.088^{***}	-0.038^{**}	0.018
	(0.022)	(0.020)	(0.019)	(0.038)
predicted Neuroticism	0.001	-0.054^{**}	-0.095^{***}	0.151^{***}
	(0.024)	(0.022)	(0.020)	(0.040)
Neuroticism	−0.026	−0.027	−0.006	−0.031
	(0.024)	(0.022)	(0.020)	(0.041)
Constant	−0.001	0.018	0.017	−0.052
	(0.019)	(0.018)	(0.017)	(0.034)
Observations	2,687	3,022	3,504	896
R^2	0.016	0.031	0.020	0.071
Adjusted R^2	0.014	0.029	0.018	0.064
Residual Std. Error	1.004 (df = 2680)	0.997 (df = 3015)	0.992 (df = 3497)	0.992 (df = 889)
F Statistic	7.334^{***} (df = 6; 2680)	15.854^{***} (df = 6; 3015)	11.733^{***} (df = 6; 3497)	11.256^{***} (df = 6; 889)
Note:				*p<0.1; **p<0.05; ***p<0.01

Table S4. Variance Inflation Factors (VIF) for joint proxy-target models of health-related habits

	Alcohol	Activity	Sleep	Smoking
predicted Age	3.063	3.149	3.076	3.000
Age	3.108	3.181	3.123	3.070
predicted Fluid Intelligence	1.259	1.254	1.266	1.254
Fluid Intelligrence	1.220	1.223	1.229	1.229
predicted Neuroticism	1.451	1.457	1.460	1.590
Neuroticism	1.434	1.435	1.439	1.552

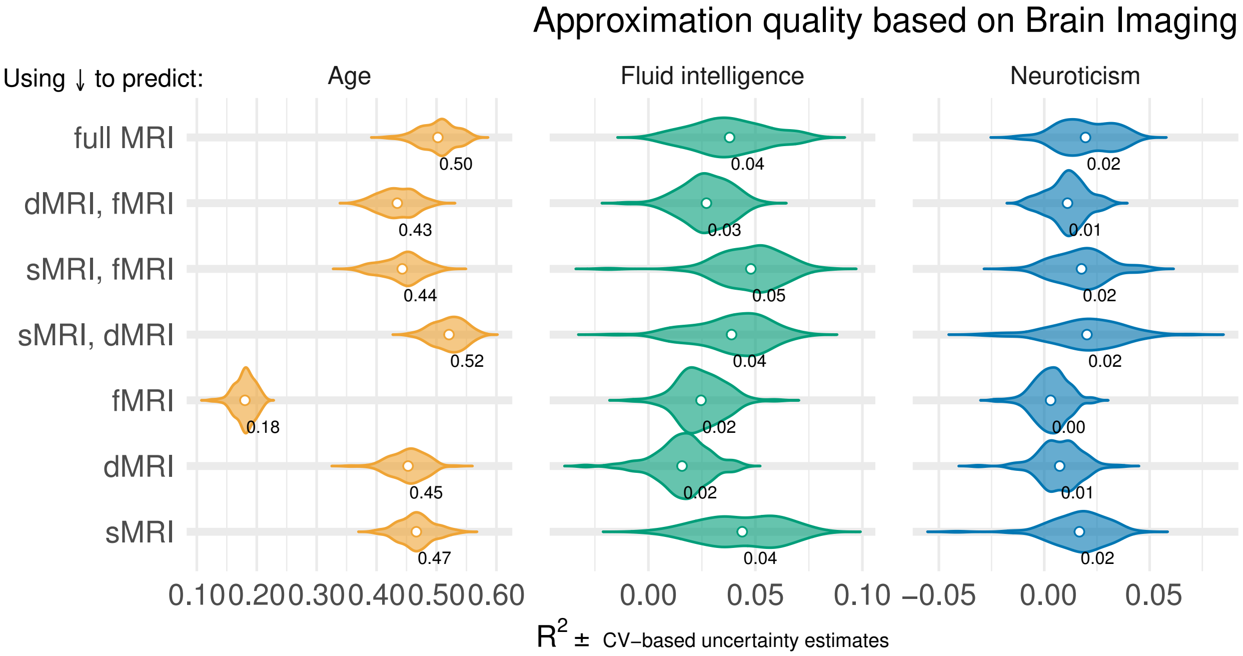

Figure 3 – Figure supplement 1. Prediction of individual differences in proxy measures from MRI. Approximation performance using multiple MR modalities on the validation dataset: sMRI, dMRI, rfMRI and their combinations (see Table 1). Visual conventions as in Figure 3. One can see that prediction of age was markedly stronger than prediction of fluid intelligence or prediction of neuroticism. As a general trend, models based on multiple MRI modalities tended to yield better prediction. For simplicity, we based subsequent analyses on the full model based on all MRI data.

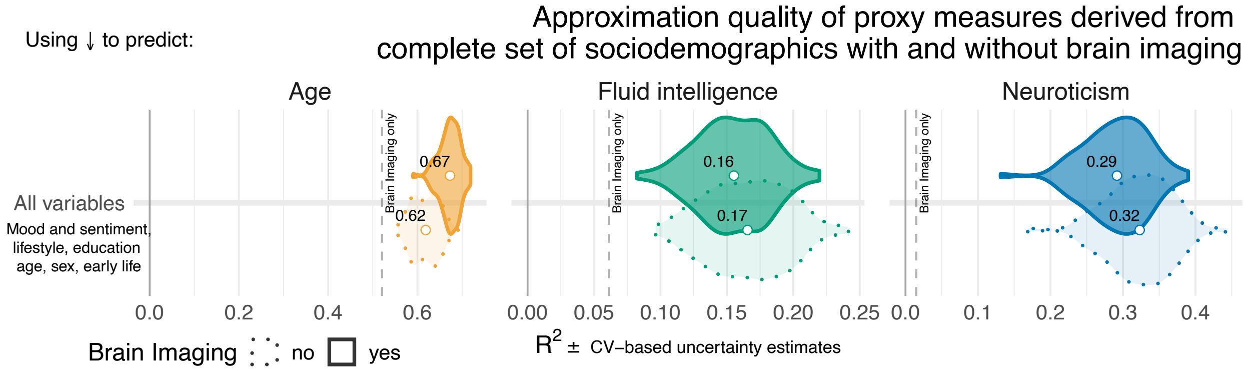

Figure 3 – Figure supplement 2. Approximation performance using all sociodemographic data. Approximation performance using all sociodemographic variables with or without brain imaging included on the validation dataset. Visual conventions as in Figure 3. The performance was highly related to the best performing models within each target Figure 3, *i.e.*, life style for age, education for fluid intelligence and mood & sentiment for neuroticism. This suggests that for each target those specific blocks of predictors were sufficiently explaining the performance. For simplicity, we based subsequent analyses on all sociodemographic variables in Figure 2, Figure 3 and Figure 4.

Table S5. Regression statistics on the held-out set for purely MRI-based approximation.

Target	$R^2_{observed}$	p-value	CI_{low}	CI_{high}
Age	0.521	1×10^{-4}	0.502	0.538
Fluid intelligence	0.061	1×10^{-4}	0.052	0.070
Neuroticism	0.015	1×10^{-4}	0.005	0.024

Table S6. Classification difference statistics on the held-out set for MRI-based approximation.

Target	$AUC_{observed}$	p-value	CI_{low}	CI_{high}
Neuroticism	0.590	1×10^{-4}	0.566	0.614
Age	0.916	1×10^{-4}	0.905	0.927
Fluid intelligence	0.667	1×10^{-4}	0.643	0.690

Appendix 2: Sociodemographic variables

Table S7. List of variables contained in each block of sociodemographic models: mood & sentiment (MS), Age, Sex (AS), Education (EDU), Early life (EL).

Group	UKBB code	Variables
**Mood & Sentiment**	2040-2.0	Risk taking
	4526-2.0	Happiness
	4537-2.0	Work/job satisfaction
	4548-2.0	Health satisfaction
	4559-2.0	Family relationship satisfaction
	4570-2.0	Friendships satisfaction
	4581-2.0	Financial situation satisfaction
	4598-2.0	Ever depressed for a whole week
	4609-2.0	Longest period of depression
	4620-2.0	Number of depression episodes
	4631-2.0	Ever unenthusiastic/disinterested for a whole week
	4642-2.0	Ever manic/hyper for 2 days
	4653-2.0	Ever highly irritable/argumentative for 2 days
	2050-2.0	Frequency of depressed mood in last 2 weeks
	2060-2.0	Frequency of unenthusiasm / disinterest in last 2 weeks
	2070-2.0	Frequency of tenseness / restlessness in last 2 weeks
	2080-2.0	Frequency of tiredness / lethargy in last 2 weeks
	2090-2.0	Seen doctor (GP) for nerves, anxiety, tension or depression
	2100-1.0	Seen a psychiatrist for nerves, anxiety, tension or depression
	5375-2.0	Longest period of unenthusiasm / disinterest
	5386-2.0	Number of unenthusiastic/disinterested episodes
	5663-2.0	Length of longest manic/irritable episode
	5674-2.0	Severity of manic/irritable episode
	6145-2.0	Illness, injury, bereavement, stress in last 2 years
	6156-2.0	Manic/hyper symptoms
**Age, Sex**	31-0.0	Sex
	34-0.0	Year of birth
	52-0.0	Month of birth
	21022-0.0	Age at recruitment
	21003-2.0	Age when attended assessment centre
**Education**	6138-2.0	Qualifications
	845-2.0	Age completed full time education
	1647-2.0	Country of birth (UK/elsewhere)
	1677-2.0	Breastfed as a baby
	1687-2.0	Comparative body size at age 10
	1697-2.0	Comparative height size at age 10
**Early life**	1707-2.0	Handedness (chirality/laterality)
	1767-2.0	Adopted as a child
	1777-2.0	Part of a multiple birth
	1787-2.0	Maternal smoking around birth
**Lifestyle**	670-2.0	Type of accommodation lived in
	680-2.0	Own or rent accommodation lived in
	6139-2.0	Gas or solid-fuel cooking/heating
	699-2.0	Length of time at current address
	709-2.0	Number in household
	6141-2.0	How are people in household related to participant

Table S7 continued

728-2.0	Number of vehicles in household
738-2.0	Income before tax
796-2.0	Distance between home and job workplace
757-2.0	Time employed in main current job
767-2.0	Length of working week for main job
777-2.0	Freq. of travelling from home to job workplace
6143-2.0	Transport type for commuting to job workplace
6142-2.0	Current employment status
806-2.0	Job involves mainly walking or standing
816-2.0	Job involves heavy manual or physical work
826-2.0	Job involves shift work
3426-2.0	Job involves night shift work
1031-2.0	Freq. of friend/ family visits
6160-2.0	Leisure/social activities
2110-2.0	Able to confide
1239-2.0	Current tobacco smoking
1249-2.0	Past tobacco smoking
1259-2.0	Smoking/smokers in household
1269-2.0	Exposure to tobacco smoke at home
1279-2.0	Exposure to tobacco smoke outside home
2644-2.0	Light smokers, at least 100 smokes in lifetime
2867-2.0	Age started smoking in former smokers
2877-2.0	Type of tobacco previously smoked
2887-2.0	Number of cigarettes previously smoked daily
2897-2.0	Age stopped smoking
2907-2.0	Ever stopped smoking for 6+ months
2926-2.0	Number of unsuccessful stop-smoking attempts
2936-2.0	Likelihood of resuming smoking
3436-2.0	Age started smoking in current smokers
3446-2.0	Type of tobacco currently smoked
3456-2.0	Number of cigarettes currently smoked daily (current cigarette smokers)
3466-2.0	Time from waking to first cigarette
3476-2.0	Difficulty not smoking for 1 day
3486-2.0	Ever tried to stop smoking
3496-2.0	Wants to stop smoking
3506-2.0	Smoking compared to 10 years previous
5959-2.0	Previously smoked cigarettes on most/all days
6157-2.0	Why stopped smoking
6158-2.0	Why reduced smoking

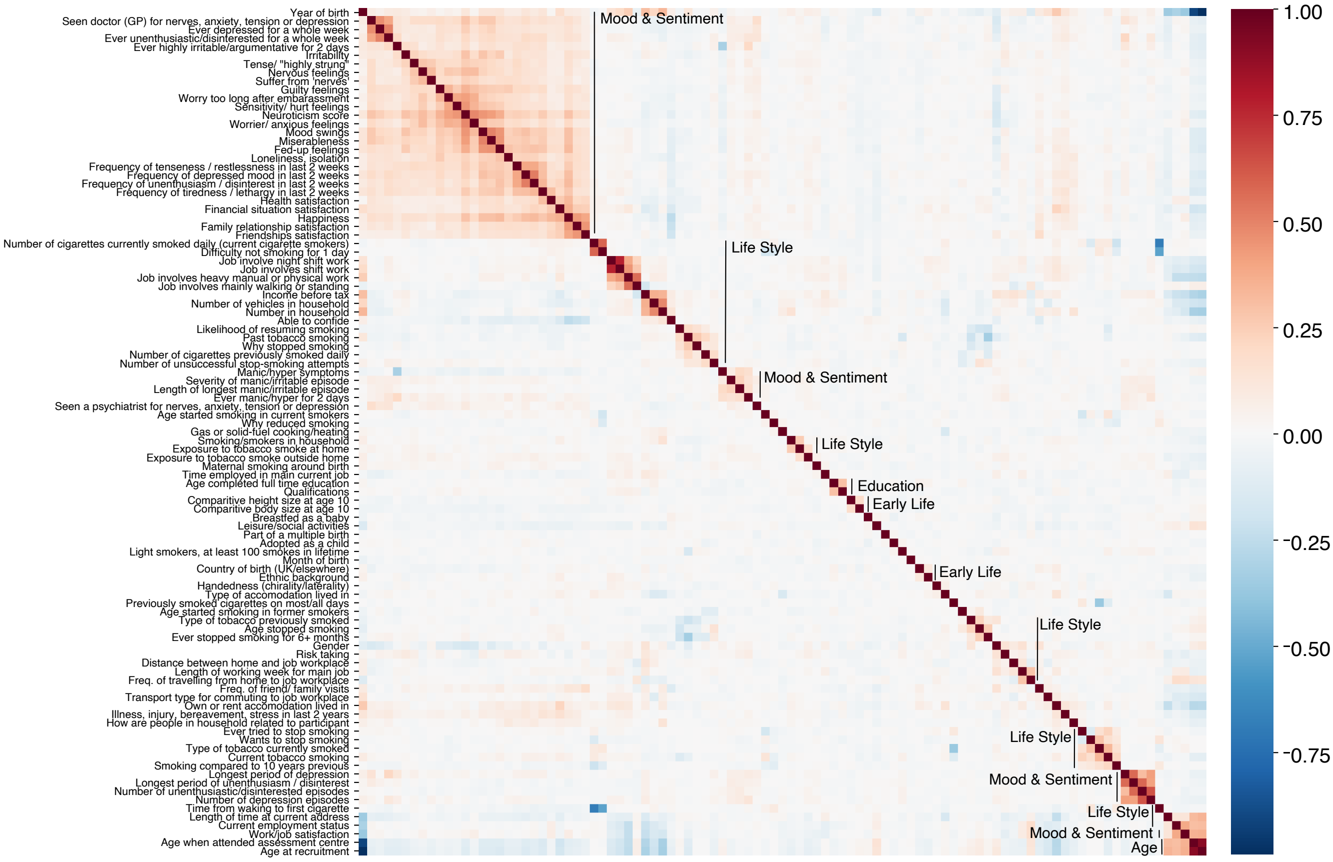

Figure S1. Intercorrelations between sociodemographic inputs. To check the plausibility of the proposed grouping of variables into blocks, we investigated the inter-correlations among the sociodemographic inputs (Table S7). We first applied Yeo-Johnson power transform to the variables yield approximately symmetrical distributions. Then we computed Pearson correlations. One can see that a large majority of variables shows low if any inter-correlations. Strongly inter-correlated blocks emerged, in particular for Mood & Sentiment and Life Style. Note that within the Life Style category many smaller blocks with strong inter-correlation occurred, some of which were obviously related to the circumstance of living such as household or employment status.

Appendix 3: Impact of Measurement Time

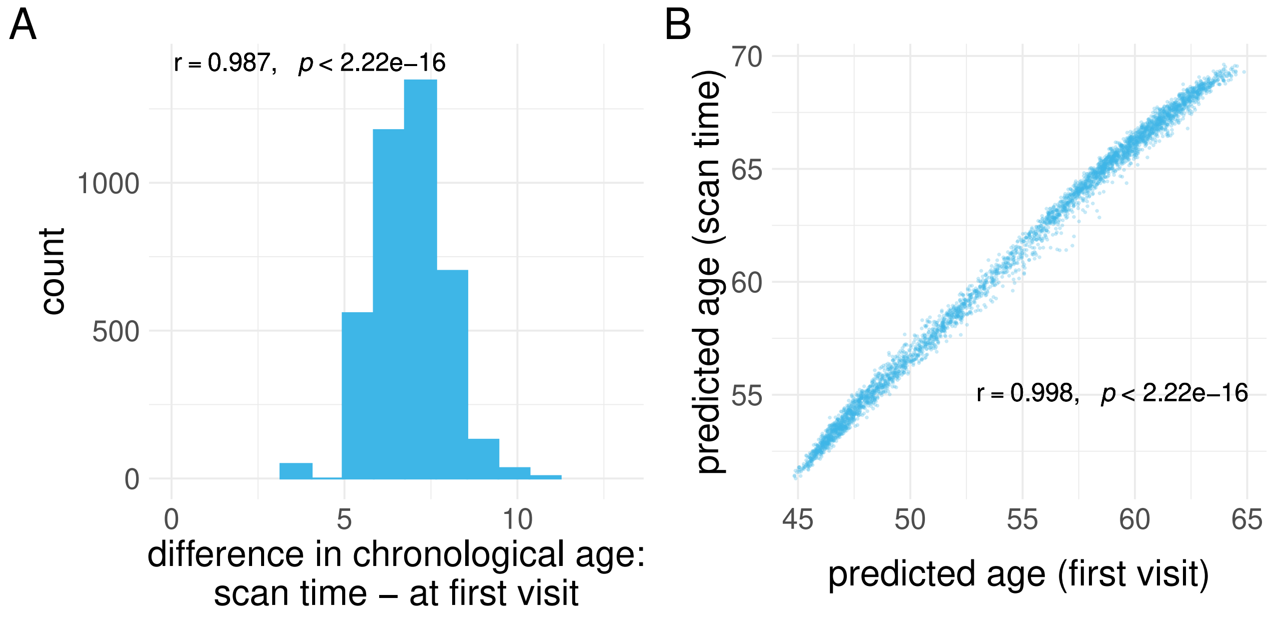

Figure S2. Investigating the age gap between the first visit and the MRI-visit time point. **(A)** Individual gap between age at first visit and MRI-scan time. MRI scans never happened at the first visit, leading to a strictly positive gap greater than five years for most participants. Pearson's correlation coefficient indicates high rank stability, suggesting that, from a statistical perspective, age at first visit and age at scan time are, essentially, interchangeable. **(B)** Direct comparison of individual-specific age predictions from brain images and sociodemographic data. Same model as in the main analysis (Figure 2). The emerging pattern of association summarized by Pearson's correlation coefficient suggests that predictions from models either trained on age at the first visit or at MRI-scan time are equivalent.

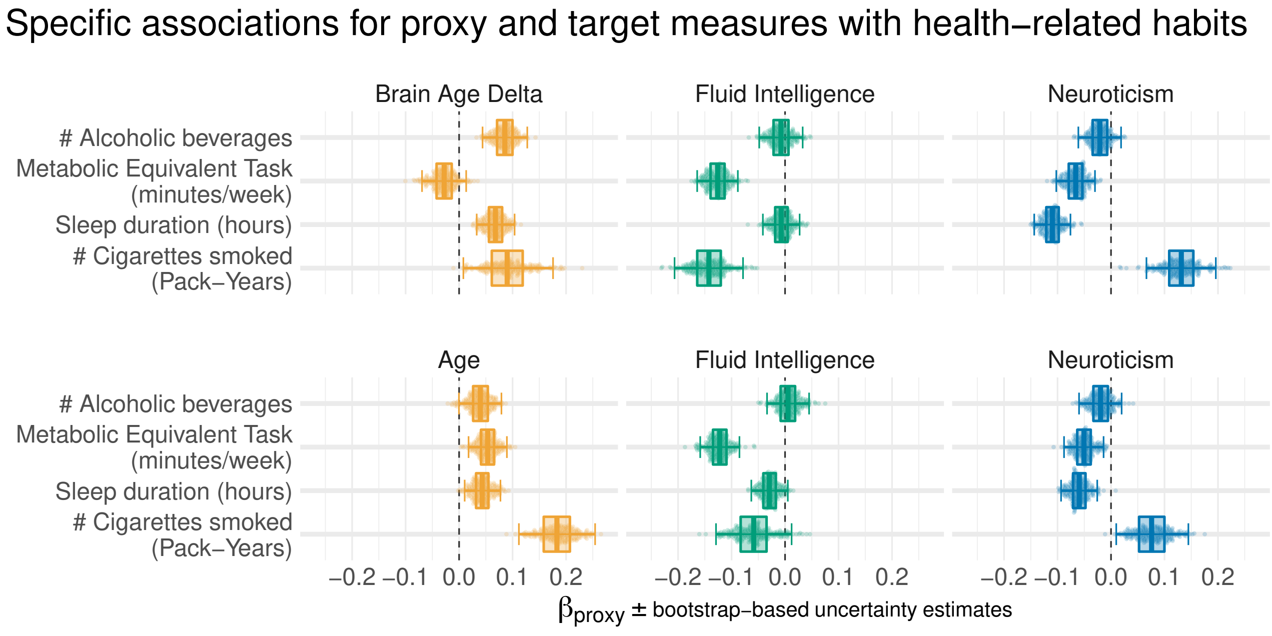

Figure S3. Proxy measures show systematic and complementary out-of-sample associations with health-related habits using age at MRI-scan time. The patterns observed in Figure 2 and global conclusions remain unchanged.
