# Supplementary material for: Population modeling with machine learning can enhance measures of mental health
Source: Gigascience. 2021 Oct 15;10(10):giab071. doi: 10.1093/gigascience/giab071 (PMC8559220; doi:10.1093/gigascience/giab071)
Supplement: giab071_Response_to_Reviewer_Comments_Original_Submission [file giab071_response_to_reviewer_comments_original_submission.pdf]

# Author's Response To Reviewer Comments

Close

## Summary

**Please note that a fully formatted and more readable version of the response to the reviewers (including coloured highlights and the new figures and tables in context) is available as PDF among the submitted files.**

**Please note that we could not fully control the order in which the files were included by the submission system**

We would like to thank the editor and the reviewers for the thoughtful feedback and constructive criticism on our work. We have taken the revisions as an important opportunity to improve our manuscript and would like to highlight two points that both reviewers and the editor have emphasized:

1. both reviewers had concerns regarding the far-reaching conclusion: the **findings suggested that psychological constructs can be approximated from brain images and sociodemographic variables.** Considering these concerns, we added methodological and conceptual clarifications, discussed our findings more carefully and revised the general conclusions to express greater nuance.

1. both reviewers also pointed out that the phenotype **age / brain age** is different from the other measurements. We conducted multiple control analysis to investigate this point and clarify the differences, both, conceptually and statistically. The wealth of results and related discussion points are fully reported in the revised manuscript.

This required substantial reanalysis demanding several weeks of computation time and led us to including 6 new supplementary figures and 2 new supplementary tables. A detailed display of the changes in the manuscript is available in the file `diff-with-original-submission.pdf` enclosed with the resubmission.

_Please note that, where appropriate, replies are organized by bundles of related concerns, sometimes involving concerns raised by both reviewers._

## Reviewer 1

#### R1.1

The manuscript describes an application of Machine Learning (ML) models for the quantification of psychological constructs, e.g., fluid intelligence and neuroticism, using multi-model MRI data from a large population cohort, the UK biobank data. They show that the proxy measures of these psychological constructs are more useful compared to the original constructs for characterizing health behaviors. Overall, the manuscript is well written. The research questions are clearly stated and are of practical importance. However, the reviewer has following concerns.

**Reply to R1.1:** We thank the reviewer for the positive appreciation of our work.

#### R1.2

Major Concerns:

1) In page 3 (left, lines 3-6 of the main text), the author claims that Our findings suggested that psychological constructs can be approximated from brain images and sociodemographic variables - inputs not tailored to specifically measure these constructs.. The reviewer has concerns about this claim. Although Figure 3 shows the models performance in predicting age, fluid intelligence and neuroticism using neuroimaging data and different areas of sociodemographic data, the performance of the models

in predicting the psychological constructs, fluid intelligences and neuroticism, may not be good enough to support such a claim.

**Reply to R1.2** : We thank the reviewer for pointing out the potential ambiguity of the wording. An approximation can be understood as an almost perfect prediction but also as a crude guess. In fact, we did not intend to suggest that the approximations are perfect. We have now rephrased the above sentence accordingly and, moreover, carefully edited other parts of the main text to help avoid this misunderstanding.

**Changes**

In abstract (instead of advantage):

We observed this complementarity of proxy measures and original measures when modeling from brain signals or sociodemographic data, capturing multiple health-related constructs.

In results section title (instead of outperforming):

Complementing the original measures at characterizing real-life health-related habits

In results section:

Our findings suggested that some information on psychological constructs can be assembled from general inputs not specifically tailored to measure these constructs, such as brain images and sociodemographic variables. The resulting proxy measures may be regarded as crude approximations of the psychological measures, but they can nonetheless capture essential aspects of the target constructs. To probe the external validity of the proxy-measures, we investigated their link with real-world behavior, e.g., sleep, physical exercise, alcohol and tobacco consumption on left-out data.

#### R1.3

2) In Figure 2, the proxy measure and original measure show similar associations with the health phenotypes for fluid intelligence (center plot) and neuroticism (right plot), but not for the brain age delta. The main reason seems to be when doing the association analysis, the measures of the health phenotypes are de-confounded for their dependence for age (In the subsection Out-of-sample association between proxy measures and health-related habits of the statistical analysis section). However, it seems the same procedure is not applied for the association analysis of fluid intelligence and neuroticism. The estimated brain age or brain age gap depends on the age. Thus, we need to either correct the brain age or brain age gap for its dependence on the age, or de-confounded the health phenotypes dependence on age. If the author wants to derive the proxy measure of the psychological construct in the same as the brain age (or biological age), same procedure should be used to correct the proxy measures dependence on the original measure.

AND

#### R1.4

3) Based on Figure 2, the author claims that the proxy measures have enhanced association with health behavior compared to the original measures. If we only focus on the central and right part of the Figure 2, the difference is not that obvious. We do not know if the difference is significant or not. A better approach maybe is that correct the predicted fluid intelligence and predicted fluid intelligence for their dependence on the original measures or de-confounded the original measures effects on the health behaviors.

**Reply to R1.3-R1.4:** We thank the reviewer for giving us the opportunity for clarifying our conceptual view and substantially extending the analysis in response to these thoughtful concerns. Note that concerns **R2.2-2.4** by reviewer 2 were highly related. Therefore, the following reply is intended to address the same issue raised by both reviewers **.**

First of all, we believe that the three measures under study are fundamentally distinct and lead to different statistical behaviors, which is the reason for which we included them in this study. Age is a metric measure, related to physical units on which intervals of the same size express the same distance.

A delta can thus be a meaningful summary, which, in the case of brain age, has a straight-forward interpretation. For the other measures, ordinal or categorical scales apply; the delta is less convincing as a proxy measure metric here and may have no meaning.

Rather, in our view, what is important is that the predicted measure contains information -- in the widest sense -- beyond the actual measure. Therefore, the popular brain age delta is not necessary here. A multivariate analysis linking both chronological age and predicted age to the outcome of interest can capture the same information (see new figure-2 supplements 2-4). On the other hand, when inspecting the other proxy measures, we found it important to use an age deconfounder, to confirm that effects were not driven by age, as age shows pervasive correlations with nearly any biobehavioral entity. This de-confounding is important to interpret results as indeed driven by the construct of interest, but associations hold without it or when adding specific deconfounders to all proxies, as we show in new analyses below (see new figure-2 supplements 3-4).

These considerations explain why we have initially processed the proxies in different ways (age-decorrelation for all proxies, delta for brain age only). We understand, however, that rational arguments alone may feel unsatisfying from a readers perspective. We have taken the opportunity to extend our analysis. To probe the robustness of our analysis to these different options we have run several checks which we have included in the supplements (described below).

While implementing these extra checks, we realized that in figure 2B, we had kept the age-deconfounder active when modeling the link between age and the health descriptors, which was not ideal as it may destroy the effect. We have corrected this accordingly and removed the age-deconfounder subsequently from the target measure analyses (see corrected figure 2 below).

New figure 2 after removing the age deconfounder for the target measures (lower panel).

(See formatted reply to the reviewers among submitted files)

**New** Supplementary analyses.

**Figure 2 supplement 2:** We ran the same analysis for figure 2 using the brain-predicted age as such, not the delta as the proxy measure. Outputs are virtually identical.

**Figure 2 supplement 3:** We then added de-confounders for fluid intelligence and neuroticism and repeated the analysis for figure 2. Outputs were highly similar.

**Figure 2 supplement 4:** To provide a more rigorous comparison between proxies and targets as stimulated in **R1.4** and assess statistical significance of possible differences between proxies and targets, we have composed a big regression model including both the proxies and the targets as predictors. Across health outcomes, we found two frequent scenarios: 1) the proxies were found statistically significant, not the actual measures, suggesting that the proxies show improved SNR over the actual measures. 2) proxies and actual measures were found statistically significant, pointing at true additive effects. The overall picture depended on the health outcome.

**Table S3:** Detailed statistical inferences for the comprehensive proxy-target models are listed in this new table.

**Table S4** : In this context, the intrinsic link between proxies and targets may imply multicollinearity. Too much multicollinearity could potentially render the statistical testing procedure invalid. To test for multicollinearity we computed variance inflation factors (VIF) across the different models, which intuitively quantify how well each predictor can be approximated by a linear combination of the other predictors. The VIF is defined as 1 / (1 - R^2_i), where R^2_i is the coefficient of determination for the model that approximates the predictor i from all remaining predictors. A VIFs 1-5 counts as low to moderate (see extensive review and discussion in Obrien 2007, _Quality & Quantity_ 41, 673-690). The observed VIFs were mostly around 1. The highest VIF was found for age and brain age with values around 3, which suggests that age and brain age are linked to the other proxy measures. This is implicitly justifying our previous intuition of keeping age-deconfounders for the other proxy measures. More importantly, these control analyses suggest that the significance tests are not impacted by multicollinearity, which is also in line with reasonable standard errors produced by the big models from S3.

Based on these new analyses, we have updated the results section and have highlighted the theoretical considerations regarding asymmetries between the proxy measures.

**Changes**

In the introduction, the asymmetry has been emphasized:

Extrapolating from these successes, we propose to build upon large datasets to extend the collection of health-related proxy measures, probing mental traits.For this end, we focused on constructs fundamentally different in terms of content and methodology.

In the main text, result section, a novel paragraph has been added to address the conceptual difference of the measures and consequences for the analysis:

The three proxy measures are difficult to compare on an equal footing as a delta was considered for brain age only (the difference between predicted and actual age) and aging- specific deconfounding was applied. The brain-age delta is indeed the standard practice, theoretically justified as age is on a metric scale [50] for which the difference between the predicted and the measured value has a clear meaning. Such a difference is less obvious for variables with ordinal scales as implied by psychometric measures. Second, age has a pervasive influence on virtually any biomedical entity, which motivates controlling for its effect on proxy measures. To rule out that differences in proxy measures associations to health-related behavior are driven by this methodological asymmetry, we repeated the main analysis from Figure 2, first, using the predicted age without computing the delta (Figure 2 – Figure supplement 2) and, second, introducing additional deconfounders for fluid intelligence and neuroticism (Figure 2 – Figure supplement 3). The resulting patterns were virtually unchanged, confirming that interpretations are robust.

In the main text, result section, a paragraph on big-picture statistical differences between proxies and targets has been generously expanded:

A question that remains is whether the proxy measures bring additional value compared to the original target measures they were derived from. These original target measures showed similar associations to health behavior, with the same signs in most cases (Figure 2, B). At the same time, the ensuing patterns were more noisy, suggesting that empirically derived proxy measures yielded enhanced associations with health behavior. This inference may be difficult as differences between targets and proxies were not always easy to pinpoint visually. To implement a more rigorous statistical approach, we built comprehensive models of each respective health-related habit in which we used all proxies (predicted age, predicted fluid intelligence, predicted neuroticism) and all targets (age, fluid intelligence, neuroticism) simultaneously as predictors (Figure 2 – Figure supplement 4). The results show systematic additive effects of proxies and targets across the three target domains and the four health-habits. These trends are well-captured by the hypothesis tests of the respective linear models (Table S3). As targets and proxies may be systematically intercorrelated, multicollinearity may corrupt these inferences. Inspection of variance inflation factors (VIF)— a measure that reveals how well a given predictor can be approximated by a linear combi- nation of the other predictors— argued in favor of low to moderate levels of multicollinearity (Table S4). Indeed, all VIF values fell between 3 and 1, whereas, classically, values above 5 or 10 are considered as thresholds [51] for pathological collinearity. This suggests that the model inferences are statistically sound.

In the main text, method section, health-related habits regression, a passage has been added describing the additional models (see blue highlight in screenshot).

(See formatted reply to the reviewers among submitted files)

The following items have been added to the supplement:

(See formatted reply to the reviewers among submitted files)

**Figure 2 supplement 2:** Conditional associations between proxy measures and health-related habits without explicit brain age delta. Conditional estimates using multivariate regression. Instead of the brain age delta, the brain-predicted age is included alongside an age-deconfounder as used in the main analysis. Same visual conventions as in Figure 2.

(See formatted reply to the reviewers among submitted files)

**Figure 2 supplement 3** Conditional associations between proxy measures and health-related habits with-proxy-specific deconfounding. Conditional estimates using multivariate regression. Instead of the brain age delta, the brain-predicted age is included alongside an age-deconfounder as used in the main analysis. Moreover, predicted fluid intelligence and neuroticism are deconfounded for the target values at training time, analogous to the brain age predictions. Same visual conventions as in Figure 2.

(See formatted reply to the reviewers among submitted files)

**Figure 2 supplement 4** Joint modeling of health-related habits from proxy and target measures. Conditional estimates using multivariate regression. Every health-related habit (double rows) is modeled simultaneously from multiple proxies and targets. Same visual conventions as in Figure 2. Across health-habits, additive effects emerged not only for proxies and targets within the same measure (e.g. age) but also across measures (e.g. age and fluid intelligence).

For illustration, we shall consider two examples. Regarding alcohol consumption, age was the most important measure and opposite conditional effects were observed for the proxy and the target: Across the age range, people with higher brain age tended to drink more and across the brain-age range, older people tended to drink less. For smoking, the proxy measures were the most important variables with clear non-zero coefficients, pointing in different directions across target domains. Holding fluid intelligence and neuroticism constant (targets and proxies), people with higher brain age tended to have been smoking for a longer time. At the same time, those who scored lower on predicted fluid intelligence across the entire range of age, predicted age, measured fluid intelligence, predicted neuroticism and neuroticism, have been smoking for a longer time. Finally, those who scored higher on predicted neuroticism tended to smoke more across the ranges of all other measures.

**Supplementary table 3**

(See formatted reply to the reviewers among submitted files)

**Supplementary table 4**

(See formatted reply to the reviewers among submitted files)

Minor concerns:

#### R1.5

1) In page 1 (two lines before reference 15), it seems that to learn is mis-spelled into tolearn.

**Reply R1.5:** We thank the reviewer for having found this typo. We have corrected it.

#### R1.6

2) The author stated that there are repeated measures for subjects in UK biobank data. How the author tackles this issue in their data preprocessing? Using the last one or the first one or something else?

**Reply R1.6** We thank reviewers for having raised this concern whose clarification may, indeed, help the reader.

Out of 500 000 participants, only a fraction has visited for repeated assessments. For instance, 10 000 participants were included in the first brain imaging release (Miller K et al. Nat. Neuroscience 2016; Thomas J. Littlejohns et al. Nat. Communications 2020). The visits are categorized as: initial assessment visit, repeated initial assessment, imaging visit and soon. These visits are coded as 0, 1, 2 and soon. In this paper, we relied mostly on assessments overlapping with imaging visit _i.e._ variables coded as 2.0 as can be seen from Table S5, _Appendix_ 2.

Some variables used in the analysis display code 0, _e.g._ for target variables like age (21022-0.0), neuroticism (20127-0.0) or external validity variables. The reason for this is that these variables were not assessed during the follow up visits. We, therefore, relied on primary visit information.

Moreover, we explicitly checked that there is no overlap of the subjects between the validation and the generalization set.

**Changes** To clarify this point, we extended the subsection _Participants_.

(...) we found 11175 participants who had repeated assessments overlapping with the first brain imaging release. Note that the features (sociodemographic variables) that we included in the analysis are measures that are self-reported during a follow-up imaging visit. (...) We made sure that the subjects used for model construction and generalization were strictly non-overlapping.

#### R1.7

3) The selection 5,587 out of all the 10,975 subjects for the modeling, while the left part is for the out-of-sample association analysis. The selection seems arbitrary. Can the author also show a learning curve, in which x is the sample size and y is the models performance, to justify their choice is enough to train an accurate ML model?

**R1.7** We thank reviewers for allowing us to clarify how we composed the training dataset.

First, we selected a percentage of training samples based on the random split half method from the scikit-learn package. As sample size differed across three targets, e.g., fewer brain images available for neuroticism prediction (see second paragraph at the subsection _Participants_) testing the out-of-sample associations on 50% of gathered samples was a pragmatic approach for obtaining sufficient data for model training and subsequent statistical inference, two complementary and distinct objectives in this investigation. Moreover, this is in line with evidence arguing in favor of larger test sets for mitigating optimism bias in estimation of out-of-sample performance (Flint et al 2021, Nat. Neuropsychopharmacology, Varoquaux et al. 2017, NeuroImage).

To explore the implications of our training and testing splits, as suggested in, we computed learning curves (displayed below). Across all targets, the results suggest that generalization performance started saturating around 1000 training samples, well below the final training samples. Adding more samples should not substantially improve generalization performance.

**Changes** To clarify this point, we have included a new figure as a supplement to the concept figure 1 and extended the method section on participants.

In the main text, methods, section Participants:

The demographics are $51.6\%$ female (5\,572) and $48.3\%$ male (5\,403) and an age range between 40-70 years (with a mean of 55 years and standard deviation of 7.5 years). The data for model training were selected using a randomized split-half procedure yielding 5\,587 individuals. The remaining subjects were set aside as a held-out set for generalization testing (see section _Model development and generalization testing_). We made sure that the subjects used for model training and generalization testing were strictly non-overlapping. Learning curves documented that the training split was sufficiently large for constructing stable prediction models (Figure 1 - supplement 1) with profiles of performance similar to latest benchmarks on model complexity in the UK Biobank (Schulz et al 2020). Moreover, simulations and empirical findings suggest that larger testing sets are more effective at mitigating optimistic performance estimates (Flint et al 2021, Varoquaux et al. 2017). Together, this provided a pragmatic solution to the inference-prediction dilemma (Bzdok et al 2020; Bzdok et al., 2018) given the two objectives of the present investigation to obtain reasonably good predictive models, while at the same time performing parameter inference of statistical models developed on the left-out data.

(See formatted reply to the reviewers among submitted files)

**Figure 1 supplement 1** Learning curves on the random split-half validation used for model building. To facilitate comparisons, we evaluated predictions of age, fluid intelligence and neuroticism from a complete set of socio-demographic variables without brain imaging using the coefficient of determination $R^2$ metric (y-axis) to compare results obtained from 100 to 3000 training samples (x-axis). The cross-validation (CV) distribution was obtained from 100 Monte Carlo splits. Across targets, performance started to plateau after around 1000 training samples with scores virtually identical to the final model used in subsequent analyses. These benchmarks suggest that inclusion of additional training samples would not have led to substantial improvements in performance.

#### R1.8

4) In the first paragraph of the Methods section, there are duplications.

Reply **R1.8:** We thank the reviewer for having pointed out the duplications. We have found and removed them.

#### R1.9

5) In the subsection of Data acquisition part, under the target measures paragraph, the age at the baseline recruitment is used as the outcome. However, in general, there is a gap between the age at baseline and the age when the MRI images were acquired. Does this matter for the data analysis in this manuscript.

**Reply R1.9:** We thank the reviewer for this thoughtful remark as we had not considered this issue in the first place. To investigate this age gap, we computed a distribution of the individual differences between the age at recruitment and the age at MRI-scan time. As expected, the difference was strictly positive, meaning that the MRI scan was always acquired after the first visit. The individual differences were highly rank-stable, suggesting that the age variables at recruitment and scan-time were equivalent from a statistical perspective. We subsequently fitted the prediction models from the main analysis using age at the time of the MRI-scan and directly compared the individual-specific predictions. As hypothesized, they were virtually identical. We have now made this point explicit in the main text and added a supplementary figure.

**Changes**

In methods, data acquisition, target measures:

In the course of this work, a question that emerged concerned the size of the gap between age at baseline recruitment and MRI-scan time and its potential impact on the analysis. Supplementary checks indicated that the age gap was at least 5 years for most participants. Yet, from a statistical perspective, the two age measures turned out highly interchangeable (Figure S2) and global conclusions remained unchanged (Figure S3).

(See formatted reply to the reviewers among submitted files)

**Figure S2** Investigating the age gap between the first visit and the MRI-visit time point. **(A)** Individual gap between age at first visit and MRI-scan time. MRI scans never happened at the first visit, leading to a strictly positive gap greater than five years for most participants. Pearsons correlation coefficient indicates high rank stability, suggesting that, from a statistical perspective, age at first visit and age at scan time are, essentially, interchangeable. **(B)** Direct comparison of individual-specific age predictions from brain images and sociodemographic data. Same model as in the main analysis (Figure 2). The emerging pattern of association summarized by Pearsons correlation coefficient suggests that predictions from models either trained on age at the first visit or at MRI-scan time are equivalent.

(See formatted reply to the reviewers among submitted files)

**Figure S3** Proxy measures show systematic and complementary out-of-sample associations with health-related habits using age at MRI-scan time. The patterns observed in Figure 2 and global conclusions remain unchanged.

#### R1.10

6) For the classification analysis (paragraph Classification analysis in the subsection of Comparing predictive models to approximate target measures, and the paragraph above the Discussion section), the thresholds selected to discretize the outcome variables are kind of arbitrary.

**Reply R1.10:** The division into groups follows the practical recommendations by Gelman and Hill 2007 for dichotomizing variables. The idea is to avoid having too many cases near the median boundary (where differences should be very subtle). We have now explained the idea and referenced Gelman and Hills book.

**Changes**

In results, the relative importance of brain and sociodemographic data depends on the target:

To investigate empirically-defined proxy measures beyond continuous regression, we performed binary classification of extreme groups obtained from discretizing the targets using the 33rd and 66th percentiles, following the recommendations by Gelman and Hill (2006) regarding discrete variable encoding strategies.

In method section, predictive model, classification analysis:

We also performed classification analysis on the continuous targets. Adapting recommendations from Gelman and Hill 2005, we performed discrete variable encoding of the targets leading to extreme groups based on the 33rd and 66th percentiles (see

Table 4) for the number of classification samples per group). This choice avoids including samples near the average outcome for which the input data may be indistinct.

## Reviewer 2

All in all, this is a scientifically interesting study, but I think the presentation could be improved, by more clearly stating the aims of it, and by giving more insight in certain aspects of the proxy modeling.

We thank the reviewer for the positive appreciation of our work and the constructive criticism. In response to this concern, we have carefully edited the main text, extended the discussion and taken efforts to make our theoretical standpoint more explicit.

#### R2.1

This manuscript reports on the results of a study that can be split into two parts. For this, it should be noted that the authors consider three categories of quantities. The first category are the input data, or predictors: (a) variables derived from MRI scans and (b) rich sociodemographic variables. The second category, or target variables, as the authors call them, include: (a) age, (b) fluid intelligence and (c) neuroticism. In the first part of the study, using machine learning, predictive models are built to predict the target variables from the input variables. The resulting predictions are called proxy measures. For the second stage, a third category of variables is included, the real world health behaviours, such as alcohol use and physical activity. The authors now set out to predict these measures of behaviour based on the measures of the second category, either the real ones or the proxies. Thus, the question is, can alcohol use be better predicted by neuroticism determined from a questionnaire, or by the neuroticism proxy derived from MRI and sociodemographics? The main results are presented in Figure 2, and the conclusion made by the authors is that the proxies perform better than the real measures.The authors carry out additional analyses, including the study of the relative importance of MRI and sociodemographics. The authors suggest that these proxies may have clinical use in the future.

At first sight it may seem surprising that proxies perform better then the real measure in capturing the associations, but, as the authors mention, the real measures suffer from (measurement) noise and non-objectivity. However, the proxies are biased (in the sense of being to simple) and are thus less capable of modeling the (true) individual variation. I would have expected a more in depth discussion about this.

**Reply to R2.1:** We thank the reviewer for sharing this thoughtful impression with us. We now realize that the current wording of the paper may have led to the impression that we see the proxies as a drop-in-replacement for the actual targets (see also **R1.2** and **R1.4).** Instead, we propose that the proxies can be flexibly used to complement the original measures or act as replacements when the original measures are not available. Of note, in the context of machine learning, biased models often lead to better predictions as it can reduce uncertainty as in the classical bias/variance tradeoff. We have now extended the main text to make our perspective clearer to the reader and avoid this kind of misunderstanding and extended the discussion to better explain the potential mechanism by which proxy measures function.

**Changes**

In the discussion:

A more complete view on how the proxy measures capture mental-health constructs emerges from their associations with real-world behavior (Figure 2). Indeed, the associations with proxy measures (Figure 2 A) were less noisy and more consistent than with the target measures (Figure 2 B), regardless of their approximation quality. This may seem surprising at first, but the target measures are themselves noisy and of imperfect validity. These measures correspond to traditional tests which, in practice, must be interpreted by an expert, actively confronting their output with broader information on the individual. For instance, IQ scores are typically normalized across age groups. However, extending such a normalization approach to many factors (socio-economic status, culture, gender) poses fundamental high-dimensional statistics challenges. Conversely, using machine learning to assemble proxy measures by mapping the targets to rich sociodemographic and brain data implicitly contextualizes them. In this respect, the resulting measures capture more general signal than the original tests. Here, machine learning could be seen as mimicking the work of a mental health expert who carefully compares psychometric results with other facts known about an individual and its reference population.

Conclusion:

In population studies of mental health, individual traits are captured via lengthy assessments, tailored to specific brain and psychological constructs. We have shown that proxy measures built empirically from general-purpose data can capture these constructs and can improve upon traditional measures when studying real-world health patterns. Proxy measures can make psychological constructs available to broader, more ecological studies building on large epidemiological cohorts or real-world evidence. This can make the difference where psychological constructs are central to developing treatment and prevention strategies, but direct measures have not been collected.

**Related changes reported above in response to R 1.2**

In abstract (instead of advantage):

We observed this complementarity of proxy measures and original measures when modeling from brain signals or sociodemographic data, capturing multiple health-related constructs.

In results section title (instead of outperforming):

Complementing the original measures at characterizing real-life health-related habits

In results section:

Our findings suggested that some information on psychological constructs can be assembled from general inputs not specifically tailored to measure these constructs, such as brain images and sociodemographic variables. The resulting proxy measures may be regarded as crude approximations of the psychological measures, but they can nonetheless capture essential aspects of the target constructs. To probe the external validity of the proxy-measures, we investigated their link with real-world behavior, e.g., sleep, physical exercise, alcohol and tobacco consumption on left-out data.

#### R2.2

Apart from this, there is an asymmetry in the way age is treated as compared to the other two target variables, intelligence and neuroticism. Age is a very hard measure, without any measurement error, and independent of the brain.

AND

#### R2.3

The other two targets, intelligence and neuroticism, are softer measures, and directly related to the brain. How does this influence the analyses and the results?

AND

#### R2.4

Indeed, not predicted age is used as proxy, but brain age delta. I would have liked to see more explanation and discussion about this.

Reply to **R2.2-2.4:** We thank the reviewer for sharing these thoughtful concerns. The reviewer is absolutely right that we have treated the target variables differently, as they are fundamentally different in terms of data generating mechanisms and measurement scale. The difference between target and proxy, e.g., the delta, can make sense where one deals with physical units (e.g. years for time) but may make less sense for ordinal or categorical variables (the effective measurement scale for intelligence and neuroticism is less clear; it is only clear that there are no physical units).

These points have also been brought up by reviewer 1 and that the reply, the edits on the manuscript and the additional analyses in response **R1.3-1.4** were explicitly designed to also address concerns **R2.2-2.4**.

Moreover, we have revised the main text to make this asymmetry even more explicit, borrowing the proposed terminology of hard and soft measures. Finally, we have presented evidence in the main text that the proposed framework is flexible enough to handle these intrinsic differences. It turned out that very similar results are obtained even when the three measures are not treated asymmetrically but identically.

**New** Supplementary analyses.

**Figure 2 supplement 2:** We ran the same analysis for figure 2 using the brain-predicted age as such, not the delta as the proxy measure. Outputs are virtually identical.

**Figure 2 supplement 3:** We then added de-confounders for fluid intelligence and neuroticism and repeated the analysis for figure 2. Outputs were highly similar.

**Figure 2 supplement 4:** To provide a more rigorous comparison between proxies and targets as stimulated in **R1.4** and assess statistical significance of possible differences between proxies and targets, we have composed a big regression model including both the proxies and the targets as predictors. Across health outcomes, we found two frequent scenarios: 1) the proxies were found statistically significant, not the actual measures, suggesting that the proxies show improved SNR over the actual measures. 2) proxies and actual measures were found statistically significant, pointing at true additive effects. The overall picture depended on the health outcome.

**Table S3:** Detailed statistical inferences for the comprehensive proxy-target models are listed in this new table.

**Table S4** : In this context, the intrinsic link between proxies and targets may imply multicollinearity. Too much multicollinearity could potentially render the statistical testing procedure invalid. To test for multicollinearity we computed variance inflation factors (VIF) across the different models, which intuitively quantify how well each predictor can be approximated by a linear combination of the other predictors. The VIF is defined as 1 / (1 - R^2_i), where R^2_i is the coefficient of determination for the model that approximates the predictor i from all remaining predictors. A VIFs 1-5 counts as low to moderate (see extensive review and discussion in Obrien 2007, _Quality & Quantity_ 41, 673-690). The observed VIFs were mostly around 1. The highest VIF was found for age and brain age with values around 3, which suggests that age and brain age are linked to the other proxy measures. This is implicitly justifying our previous intuition of keeping age-deconfounders for the other proxy measures. More importantly, these control analyses suggest that the significance tests are not impacted by multicollinearity, which is also in line with reasonable standard errors produced by the big models from S3.

Based on these new analyses, we have updated the results section and have highlighted the theoretical considerations regarding asymmetries between the proxy measures.

**Related changes reported above in response to reviewer 1**

In the interest of a concise reply, only related changes to the text are reprinted below. For the related figures and tables, please consider the reply to points **R1.3-1.4** above.

In the introduction, the asymmetry has been emphasized:

Extrapolating from these successes, we propose to build upon large datasets to extend the collection of health-related proxy measures, probing mental traits.For this end, we focused on constructs fundamentally different in terms of content and methodology.

In the main text, result section, a novel paragraph has been added to address the conceptual asymmetry of the measures and consequences for the analysis:

The three proxy measures are difficult to compare on an equal footing as a delta was considered for brain age only (the difference between predicted and actual age) and aging-specific deconfounding was applied. The brain-age delta is in- deed the standard practice, theoretically justified as age is on a metric scale [50] for which the difference between the predicted and the measured value has a clear meaning. Such a difference is less obvious for variables with ordinal scales as im- plied by psychometric measures. Second, age has a pervasive influence on virtually any biomedical entity, which motivates controlling for its effect on proxy measures. To rule out that differences in proxy measures associations to health-related behavior are driven by this methodological asymmetry, we repeated the main analysis from Figure 2, first, using the predicted age without computing the delta (Figure 2 – Figure supplement 2) and, second, introducing additional deconfounders for fluid intelligence and neuroticism (Figure 2 – Figure supplement 3). The resulting patterns were virtually unchanged, confirming that our interpretations are robust.

In the main text, result section, a paragraph on big-picture statistical differences between proxies and targets has been generously expanded:

A question that remains is whether the proxy measures bring additional value compared to the original target measures they were derived from. These original target measures showed similar associations to health behavior, with the same signs in most cases (Figure 2, B). At the same time, the ensuing patterns were more noisy, suggesting that empirically derived proxy measures yielded enhanced associations with health behavior. This inference may be difficult as differences between targets and proxies were not always easy to pinpoint visually. To implement a more rigorous statistical approach, we built comprehensive models of each respective health-related habit in which we used all proxies (predicted age, predicted fluid intelligence, predicted neuroticism) and all targets (age, fluid intelligence, neuroticism) simultaneously as predictors (Figure 2 – Figure supplement 4). The results show systematic additive effects of proxies and targets across the three target domains and the four health-habits. These trends are well-captured by the hypothesis tests of the respective linear models (Table S3). As targets and proxies may be systematically intercorrelated, multicollinearity may corrupt these inferences. Inspection of variance inflation factors (VIF)— a measure that reveals how well a given predictor can be approximated by a linear combination of the other predictors— argued in favor of low to moderate levels of multicollinearity (Table S4). Indeed, all VIF values fell between 3 and 1, whereas, classically, values above 5 or 10 are considered as thresholds [51] for pathological collinearity. This suggests that the model inferences are statistically sound.

#### R2.5

Finally, the suggested clinical use of the proxies is not supported well enough in my opinion. Maybe the authors could add more this discussion to this point as well.

We thank the reviewer for this suggestion. We absolutely agree with this impression. Unfortunately, relevant clinical data have not been available in the UK-Biobank. At the same time, the focus here is a public health perspective targeting individual differences in health, not pathology. We believe that, beyond the exact measures studied here, the framework, theory and methods proposed in this work can be readily applied with other measures and applied in the clinical setting, which is something that needs to be done in forthcoming studies. We have extended the discussion acknowledging this limitation in the dedicated section.

**Changes**

In limitations:

In terms of mental-health research, this study falls short of directly testing the clinical relevance of estimated proxy measures. Even in a very large general-population cohort such as the UK Biobank,

there are only a few hundred diagnosed cases of mental disorders (ICD-10 mental-health diagnoses from the F chapter) with brain-imaging data available. As a result, we could not directly assess the performance of proxy measures in clinical populations.

The low number of diagnosed mental disorders in UK Biobank highlights the practical importance of studying mental health as a continuous, in addition to diagnosed conditions. Indeed, a public health perspective calls for targeting individual differences in health, not only pathology. Psychological constructs such as IQ and neuroticism are important factors of the epidemiology of psychiatric disorders [38, 30, 29, 67], and accelerated brain aging is associated with various neurological conditions [18, 17, 25]. Yet, few cohorts come with extensive neuropsychological testing. Validated proxies of these constructs open the door to including them in epidemiological studies as secondary outcomes or additional explanatory variables.

**Related changes reported above in earlier response to related concerns by reviewer 1**

In the discussion:

A more complete view on how the proxy measures capture mental-health constructs emerges from their associations with real-world behavior (Figure 2). Indeed, the associations with proxy measures (Figure 2 B) were less noisy and more consistent then with the target measures (Figure 2 A), regardless of their approximation quality. This may seem surprising at first, but the target measures are themselves noisy and of imperfect validity. These measures correspond to traditional tests which, in practice, must be interpreted by an expert, actively confronting their output with broader information on the individual. For instance, IQ scores are typically normalized across age groups. Extending such normalization approach to many factors (socio-economic status, culture, gender) poses however fundamental challenges of high-dimensional statistics. Conversely, using machine learning to assemble proxy measures by mapping the targets to rich sociodemographic and brain data implicitly contextualizes them. In this respect, the resulting measure captures more general signal than the original tests. Here, machine learning could be seen as mimicking the work of a mental health expert who carefully compares psychometric results with other facts known about an individual and its reference population.

Conclusion:

In population studies of mental health, individual traits are captured via lengthy assessments, tailored to specific brain and psychological constructs. We have shown that proxy measures built empirically from general-purpose data can capture these constructs and can improve upon traditional measures when studying real-world health patterns. Proxy measures can make psychological constructs available to broader more ecological studies building on large epidemiological cohorts or real-world evidence. This can make the difference where psychological constructs are central to developing treatment and prevention strategies, but direct measures have not been collected.

Close
