# Supplementary material for: Population modeling with machine learning can enhance measures of mental health
Source: Gigascience. 2021 Oct 15;10(10):giab071. doi: 10.1093/gigascience/giab071 (PMC8559220; doi:10.1093/gigascience/giab071)
Supplement: giab071_Response_to_Reviewer_Comments_Revision_1 [file giab071_response_to_reviewer_comments_revision_1.pdf]

Author's Response To Reviewer Comments

Close

Please note that a fully formatted and more readable version of the response to the reviewers (including coloured highlights and the new figures and tables in context) is available as PDF among the submitted files.

Note that this text renders to markdown

Summary

We would like to thank the editor and the reviewer for the attentive evaluation of our manuscript and the helpful comments. This has allowed us to further refine the manuscript regarding editorial standards and the presentation of our workflow. The key points addressed in this revision are a major update to figure 1 as well as changes in the text that, hopefully, will help the reader to better understand the core ideas of the paper and the methods workflow.

A detailed display of the changes in the manuscript is available in the file `diff-with-r1-submission.pdf` enclosed with the resubmission.

Editorial points

- Please turn URLs mentioned in the text or the footnotes into numbered citations, included in the bibliography. Software homepages, data sets and other URLs or DOIs should all be included in the bibliography and cited from there. please see our instructions for authors for formatting guidance.

- In the data availability section, please explain the access procedure for the UK BioBank data (Is it open or controlled access? For human data, controlled access is fine, but please mention the requirements/procedure to get access).

- Our data curators will prepare an archival snapshot of code and supporting data. Please also include this in the bibliography (including the doi link) and cite it by number from the availability section.

Reply:

We thank the editor for the support regarding editorial standards. In the revised versions, we have addressed all three points.

Reviewer reports:

Reviewer #1:

The substantial revision improved the paper and is appreciated by the reviewer. The details have been enhanced. However, the reviewer still has some **concerns about the basic logic and its presentation of the paper** after reviewing all the comments from other reviewers and the feedback from the author.

Reply:

We thank the reviewer for the positive appreciation of our revision efforts. We hope that the second revision, in response to the thoughtful and constructive criticism provided by the reviewer, helped improve the accessibility issues regarding the basic logic and presentation of the paper.

Reviewer #1

Figure 1 is helpful (BTW, the font is too small and smaller than other figures).

Reply:

We thank the reviewer for this positive feedback and the pointer regarding the font sizes. To address this issue, we have increased the font size of the smallest fonts and scaled up the figure to appear larger inside the manuscript, using the full text width instead of one single column. As a result, all font sizes have been increased.

Reviewer #1:

But if we consider the current approach again, when the machine learning (ML) has perfect performance to generate the so called proxy measures, these measures should match exactly each individual's age, fluid-intelligence and neuroticism. What the author claimed about proxy measures providing better assessment to other health related variables **might be simply due to the imperfectness**

Reply:

We thank the reviewer for this opportunity for clarification. The reviewer recognizes an important point about the preconditions for building proxy measures: The predictors from which a proxy measure is modelled should not allow for perfect prediction (which is certainly guaranteed in our context given that the precise data generating mechanisms are unknown and it is reasonable to assume that many important variables remain unobserved). A proxy measure can only bring additional information if, in the first place, there is residual variance in the target that is not explained by the predictors. The entire approach cannot work if proxies and targets are – via perfect prediction – the same.

To make sure this point is not overlooked, in the introduction, we have revised the paragraph in which brain age is introduced. Moreover, we have added a note in the caption figure caption of figure 1.

Changes in the main text:

Yet, by (imperfectly) predicting the age from brain data, machine-learning can capture the relevant signal. Based on a population of brain images, it extracts the _best guess_for the age of a person, indirectly positioning that person within the population.

Related changes in caption of figure 1:

(...) Note that proxy measures can only add to the target measures if they are not identical, _i.e._, if the approximation of the target from the given inputs is imperfect (guaranteed in our context as the exact data generating mechanism is unknown and causally important variables remain unobserved). (...)

Related changes in figure 1 (bold text in panel C):

Machine learning combines various classes of inputs to build (imperfect) proxies for the target measures.

Reviewer #1

The author may need to address this and present the logic of the paper in a clearer way to help the readers understand the main point and results of the paper. In this regard, Figure 1 is incomplete in addressing the full flow of the paper, which is necessary for such a seemingly complex paper in the reviewer's opinion.

Reply:

We thank the reviewer for this excellent recommendation. It is true that because of the substantial revisions, now the concept figure 1 is visibly out of sync with the full story, which indeed may cause confusion or simply make readers miss the main ideas. To present the key ideas of the work with greater clarity to the reader, we have added an outlook on the organization of the paper at the end of the introduction and substantially extended figure 1 to now depict the full workflow of the paper. To generate more attention for the key idea of comparing multiple proxy measures with their respective targets, we have added an explicit illustration of differences between proxies and target measures. To

prepare the reader for the core of the paper in which proxies and targets are benchmarked regarding their potential complementarity at statistically explaining health-related behavior, we have included a new panel in figure 1 in which the health behaviors under investigation are illustrated. We have updated the figure caption accordingly.

Changes in the main text (end of introduction):

The paper is organized as follows: We first present a summary of the methodology and the workflow of building distinct proxy measures for age, fluid intelligence and neuroticism using machine learning (Figure 1). We then benchmark the proxy and the original target measures against real-world patterns of health-relevant behavior. Subsequently, through systematic model comparisons, we assess the relative contributions of brain imaging and sociodemographic data for prediction performance in the regression and classification settings. The complementarity between the proxy measures is, finally, discussed in the light of statistical considerations, potential data generating mechanisms, and applications for public health and clinical research.

Figure 1 after substantial revisions:

Please see the PDF version / manuscript for the figure.

Figure 1 caption:

Methods workflow: building and evaluating proxy measures. We combined multiple brain-imaging modalities (**A**) with sociodemographic data (**B**) to approximate health-related biomedical and psychological constructs (**C**), _i.e._, brain age (accessed through prediction of chronological age), cognitive capacity (accessed through a fluid-intelligence test) and the tendency to report negative emotions (accessed through a neuroticism questionnaire). We included the imaging data from the 10,000-subjects release of the UK biobank. Among imaging data (**A**) we considered features related to cortical and subcortical volumes, functional connectivity from rfMRI based on ICA networks, and white-matter molecular tracts from diffusive directions (see Table 1 for an overview about the multiple brain-imaging modalities). We then grouped the sociodemographic data (**B**) into five different blocks of variables related to self-reported mood & sentiment, primary demographics, lifestyle, education, and early-life events (Table 2 lists the number of variables in each block). We systematically compared the approximations of all three targets based on either brain images and sociodemographics in isolation or combined (**C**) to evaluate the relative contribution of these distinct inputs. Note that proxy measures can only add to the target measures if they are not identical, _i.e._, if the approximation of the target from the given inputs is imperfect (guaranteed in our context as the exact data generating mechanism is unknown and causally important variables remain unobserved). Using the full model (brain imaging + sociodemographics), we benchmarked complementarity of the proxy measures and the target measures with regard to real-world patterns of health behavior (**D**), i.e., the number of alcoholic beverages, exercise (metabolic equivalent task), sleep duration and the number of cigarettes smoked. Potentially additive effects between proxies and targets were gauged using multiple linear regression. Models were developed on 50% of the data (randomly drawn) based on random forest regression guided by Monte Carlo cross-validation with 100 splits (see section **Model Development and Generalization Testing**). We assessed generalization and health implications using the other 50% of the data as fully independent out-of-sample evaluations (see section **Statistical Analysis**). Learning curves suggested that this split-half approach provided sufficient data for model construction (Figure 1 – Figure 1 supplement).

Close
