# Supplementary material for: Population modeling with machine learning can enhance measures of mental health
Source: Gigascience. 2021 Oct 15;10(10):giab071. doi: 10.1093/gigascience/giab071 (PMC8559220; doi:10.1093/gigascience/giab071)
Supplement: giab071_Response_to_Reviewer_Comments_Revision_2 [file giab071_response_to_reviewer_comments_revision_2.pdf]

# Author's Response To Reviewer Comments

Close

Dear Hans, I have updated the data access section and included a diff file (PDF) for your convenience.
Best wishes,
Denis

Close
