# Supplementary material for: Population modeling with machine learning can enhance measures of mental health
Source: Gigascience. 2021 Oct 15;10(10):giab071. doi: 10.1093/gigascience/giab071 (PMC8559220; doi:10.1093/gigascience/giab071)
Supplement: giab071_Reviewer_1_Report_Original_Submission — Bo Cao -- 4/24/2021 Reviewed [file giab071_reviewer_1_report_original_submission.pdf]

Reviewer Report

Title: Population modeling with machine learning can enhance measures of mental health

Version: Original Submission **Date:** 4/24/2021

Reviewer name: Bo Cao

Reviewer Comments to Author:

The manuscript describes an application of Machine Learning (ML) models for the quantification of psychological constructs, e.g. fluid intelligence and neuroticism, using multi-mode MRI data from a large population cohort, the UK biobank data. They show that the proxy measures of these psychological constructs are more useful compared to the original constructs for characterizing health behaviors. Overall, the manuscript is well written. The research questions are clearly stated and are of practical importance. However, the reviewer has following concerns.

Major Concerns:

1) In page 3 (left, lines 3-6 of the main text), the author claims that "Our findings suggested that psychological constructs can be approximated from brain images and sociodemographic variables - inputs not tailored to specifically measure these constructs.". The reviewer has concerns about this claim. Although Figure 3 shows the model's performance in predicting age, fluid intelligence and neuroticism using neuroimaging data and different areas of sociodemographic data, the performance of the models in predicting the psychological constructs, fluid intelligences and neuroticism, may not be good enough to support such a claim.

2) In Figure 2, the proxy measure and original measure show similar associations with the health phenotypes for fluid intelligence (center plot) and neuroticism (right plot), but not for the brain age delta. The main reason seems to be when doing the association analysis, the measures of the health phenotypes are de-confounded for their dependence for age (In the subsection "Out-of-sample association between proxy measures and health-related habits" of the "statistical analysis" section). However, it seems the same procedure is not applied for the association analysis of fluid intelligence and neuroticism. The estimated brain age or brain age gap depends on the age. Thus, we need to either correct the brain age or brain age gap for its dependence on the age, or de-confounded the health phenotype's dependence on age. If the author wants to derive the proxy measure of the psychological construct in the same as the brain age (or biological age), same procedure should be used to correct the proxy measure's dependence on the original measure.

3) Based on Figure 2, the author claims that the proxy measures have enhanced association with health behavior compared to the original measures. If we only focus on the central and right part of the Figure 2, the difference is not that obvious. We do not know if the difference is significant or not. A better approach maybe is that correct the predicted fluid intelligence and predicted fluid intelligence for their dependence on the original measures or de-confounded the original measures' effects on the health behaviors.

Minor concerns:

1) In page 1 (two lines before reference 15), it seems that "to learn" is mis-spelled into "tolearn".

2) The author stated that there are repeated measures for subjects in UK biobank data. How the author tackles this issue in their data preprocessing? Using the last one or the first one or something else?
3) The selection 5,587 out of all the 10,975 subjects for the modeling, while the left part is for the out-of-sample association analysis. The selection seems arbitrary. Can the author also show a learning curve, in which x is the sample size and y is the model's performance, to justify their choice is enough to train an accurate ML model?
4) In the first paragraph of the "Methods" section, there are duplications.
5) In the subsection of "Data acquisition" part, under the "target measures" paragraph, the age at the baseline recruitment is used as the outcome. However, in general, there is a gap between the age at baseline and the age when the MRI images were acquired. Does this matter for the data analysis in this manuscript.
6) For the classification analysis (paragraph "Classification analysis" in the subsection of "Comparing predictive models to approximate target measures", and the paragraph above the "Discussion" section), the thresholds selected to discretize the outcome variables are kind of arbitrary.

Methods

Are the methods appropriate to the aims of the study, are they well described, and are necessary controls included? Choose an item.

Conclusions

Are the conclusions adequately supported by the data shown? Choose an item.

Reporting Standards

Does the manuscript adhere to the journal's guidelines on minimum standards of reporting? Choose an item.

Choose an item.

Statistics

Are you able to assess all statistics in the manuscript, including the appropriateness of statistical tests used? Choose an item.

Quality of Written English

Please indicate the quality of language in the manuscript: Choose an item.

Declaration of Competing Interests

Please complete a declaration of competing interests, considering the following questions:

- Have you in the past five years received reimbursements, fees, funding, or salary from an organisation that may in any way gain or lose financially from the publication of this manuscript, either now or in the future?
- Do you hold any stocks or shares in an organisation that may in any way gain or lose financially from the publication of this manuscript, either now or in the future?
- Do you hold or are you currently applying for any patents relating to the content of the manuscript?
- Have you received reimbursements, fees, funding, or salary from an organization that holds or has applied for patents relating to the content of the manuscript?
- Do you have any other financial competing interests?
- Do you have any non-financial competing interests in relation to this paper?

If you can answer no to all of the above, write 'I declare that I have no competing interests' below. If your reply is yes to any, please give details below.

I declare that I have no competing interests.

I agree to the open peer review policy of the journal. I understand that my name will be included on my report to the authors and, if the manuscript is accepted for publication, my named report including any attachments I upload will be posted on the website along with the authors' responses. I agree for my report to be made available under an Open Access Creative Commons CC-BY license (http://creativecommons.org/licenses/by/4.0/). I understand that any comments which I do not wish to be included in my named report can be included as confidential comments to the editors, which will not be published.

Choose an item.

To further support our reviewers, we have joined with Publons, where you can gain additional credit to further highlight your hard work (see: https://publons.com/journal/530/gigascience). On publication of this paper, your review will be automatically added to Publons, you can then choose whether or not to claim your Publons credit. I understand this statement.

Yes Choose an item.
