# Supplementary material for: Population modeling with machine learning can enhance measures of mental health
Source: Gigascience. 2021 Oct 15;10(10):giab071. doi: 10.1093/gigascience/giab071 (PMC8559220; doi:10.1093/gigascience/giab071)
Supplement: giab071_Reviewer_2_Report_Original_Submission — Hugo Schnack -- 4/27/2021 Reviewed [file giab071_reviewer_2_report_original_submission.pdf]

Reviewer Report

Title: Population modeling with machine learning can enhance measures of mental health

Version: Original Submission **Date:** 4/27/2021

Reviewer name: Hugo Schnack

Reviewer Comments to Author:

This manuscript reports on the results of a study that can be split into two parts. For this, it should be noted that the authors consider three categories of quantities. The first category are the input data, or 'predictors': (a) variables derived from MRI scans and (b) rich sociodemographic variables. The second category, or 'target variables', as the authors call them, include: (a) age, (b) fluid intelligence and (c) neuroticism. In the first part of the study, using machine learning, predictive models are built to predict the target variables from the input variables. The resulting predictions are called 'proxy measures'. For the second stage, a third category of variables is included, the 'real world health behaviours', such as alcohol use and physical activity. The authors now set out to predict these measures of behaviour based on the measures of the second category, either the 'real ones' or the 'proxies'. Thus, the question is, can alcohol use be better predicted by neuroticism determined from a questionnaire, or by the neuroticism proxy derived from MRI and sociodemographics? The main results are presented in Figure 2, and the conclusion made by the authors is that the proxies perform better than the real measures.The authors carry out additional analyses, including the study of the relative importance of MRI and sociodemographics. The authors suggest that these proxies may have clinical use in the future.
At first sight it may seem surprising that proxies perform better then the real measure in capturing the associations, but, as the authors mention, the real measures suffer from (measurement) noise and non-objectivity. However, the proxies are biased (in the sense of being to simple) and are thus less capable of modeling the (true) individual variation. I would have expected a more in depth discussion about this. Apart from this, there is an asymmetry in the way age is treated as compared to the other two target variables, intelligence and neuroticism. Age is a very hard measure, without any measurement error, and independent of the brain. The other two targets, intelligence and neuroticism, are softer measures, and directly related to the brain. How does this influence the analyses and the results? Indeed, not 'predicted age' is used as proxy, but 'brain age delta'. I would have liked to see more explanation and discussion about this. Finally, the suggested clinical use of the proxies is not supported well enough in my opinion. Maybe the authors could add more this discussion to this point as well. All in all, this is a scientifically interesting study, but I think the presentation could be improved, by more clearly stating the aims of it, and by giving more insight in certain aspects of the 'proxy modeling'.

Methods

Are the methods appropriate to the aims of the study, are they well described, and are necessary controls included? Choose an item.

Conclusions

Are the conclusions adequately supported by the data shown? Choose an item.

Reporting Standards

Does the manuscript adhere to the journal's guidelines on minimum standards of reporting? Choose an item.

Choose an item.

Statistics

Are you able to assess all statistics in the manuscript, including the appropriateness of statistical tests used? Choose an item.

Quality of Written English

Please indicate the quality of language in the manuscript: Choose an item.

Declaration of Competing Interests

Please complete a declaration of competing interests, considering the following questions:

- Have you in the past five years received reimbursements, fees, funding, or salary from an organisation that may in any way gain or lose financially from the publication of this manuscript, either now or in the future?
- Do you hold any stocks or shares in an organisation that may in any way gain or lose financially from the publication of this manuscript, either now or in the future?
- Do you hold or are you currently applying for any patents relating to the content of the manuscript?
- Have you received reimbursements, fees, funding, or salary from an organization that holds or has applied for patents relating to the content of the manuscript?
- Do you have any other financial competing interests?
- Do you have any non-financial competing interests in relation to this paper?

If you can answer no to all of the above, write 'I declare that I have no competing interests' below. If your reply is yes to any, please give details below.

I declare that I have no competing interests

I agree to the open peer review policy of the journal. I understand that my name will be included on my report to the authors and, if the manuscript is accepted for publication, my named report including any attachments I upload will be posted on the website along with the authors' responses. I agree for my report to be made available under an Open Access Creative Commons CC-BY license (http://creativecommons.org/licenses/by/4.0/). I understand that any comments which I do not wish to

be included in my named report can be included as confidential comments to the editors, which will not be published.

Choose an item.

To further support our reviewers, we have joined with Publons, where you can gain additional credit to further highlight your hard work (see: https://publons.com/journal/530/gigascience). On publication of this paper, your review will be automatically added to Publons, you can then choose whether or not to claim your Publons credit. I understand this statement.

Yes Choose an item.
